# Origin and Use of Hydroxyl Group Tolerance in Cationic Molybdenum Imido Alkylidene N‐Heterocyclic Carbene Catalysts

**DOI:** 10.1002/anie.201913322

**Published:** 2019-12-10

**Authors:** Roman Schowner, Iris Elser, Mathis Benedikter, Mohasin Momin, Wolfgang Frey, Tanja Schneck, Laura Stöhr, Michael R. Buchmeiser

**Affiliations:** ^1^ Institut für Polymerchemie Universität Stuttgart Pfaffenwaldring 55 70569 Stuttgart Germany; ^2^ Institut für Organische Chemie Universität Stuttgart Pfaffenwaldring 55 70569 Stuttgart Germany

**Keywords:** alcohols, molybdenum, N-heterocyclic carbenes, olefin metathesis

## Abstract

The origin of hydroxyl group tolerance in neutral and especially cationic molybdenum imido alkylidene N‐heterocyclic carbene (NHC) complexes has been investigated. A wide range of catalysts was prepared and tested. Most cationic complexes can be handled in air without difficulty and display an unprecedented stability towards water and alcohols. NHC complexes were successfully used with substrates containing the hydroxyl functionality in acyclic diene metathesis polymerization, homo‐, cross and ring‐opening cross metathesis reactions. The catalysts remain active even in 2‐PrOH and are applicable in ring‐opening metathesis polymerization and alkene homometathesis using alcohols as solvent. The use of weakly basic bidentate, hemilabile anionic ligands such as triflate or pentafluorobenzoate and weakly basic aromatic imido ligands in combination with a sterically demanding 1,3‐dimesitylimidazol‐2‐ylidene NHC ligand was found essential for reactive and yet robust catalysts.

## Graphical abstract

**Sometimes less is more**: reduction of electron density in cationic Mo imido alkylidene N‐heterocyclic carbene complexes substantially enhances tolerance to hydroxyl groups in olefin metathesis reactions including acyclic diene metathesis polymerization, cross‐metathesis and ring‐opening cross metathesis.

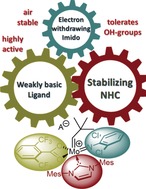

## Introduction

Olefin metathesis catalysts based on high‐oxidation state molybdenum or tungsten alkylidene species, usually referred to as “Schrock catalysts”, have been under development for decades and are a cornerstone for a vast number of regio‐ and stereoselective transformations today.^1^ However, their inherent oxophilicity and sensitivity to air and moisture limits the user‐friendliness that competing ruthenium alkylidenes can offer in many cases. Several attempts have been made to circumvent this challenging situation. By reversible chelation of the active catalysts by bipyridine or phenanthroline, Fürstner et al. showed that Mo^VI^ alkylidenes could indeed be converted into air‐stable, storable but unreactive pre‐catalysts, which can be reactivated by the addition of zinc salts to regain their original reactivity.^2^ A different approach was the formulation of a catalyst/paraffin mixture that displays significantly lowered sensitivity to air and offers the possibility of handling these catalysts without a glovebox.^3^ These admittedly elegant solutions, however, still do not address the challenge of dealing with protic groups in substrates or with traces of water, since the active species is structurally not changed and thus remains very sensitive. Therefore, costly purification and drying protocols still must be followed, even for unfunctionalized substrates or solvents. First studies on neutral, pentacoordinate Mo imido alkylidene NHC complexes revealed their remarkable stability towards functional groups including alcohols, carboxylic acids, aldehydes or amines,^4^ which in general deactivate Schrock‐type catalysts by protonation or via Wittig‐type reactions with the alkylidene ligand.^5^ So far, this enhanced functional group tolerance has only been exploited in the preparation of polymers by ring opening metathesis polymerization (ROMP) or the cyclopolymerization of α,ω‐diynes by *neutral*, *pentacoordinate* molybdenum imido alkylidene NHC bistriflate species.

## Results and Discussion

**Figure 1:**
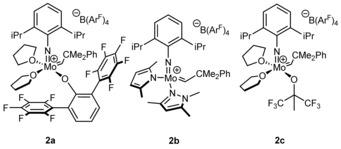
Cationic Schrock‐type catalysts **2 a**–**c** prepared for this study. B(Ar^F^)_4_=tetrakis[3,5‐bis(trifluoromethyl)phenyl]borate.

We were interested in the question, whether the reported tolerance towards hydroxyl groups and general reactivity would be retained under more harsh conditions and employed the previously reported4a neutral pentacoordinate complex Mo(*N*‐2,6‐Me_2_‐C_6_H_3_)(CHCMe_2_Ph)(IMesH_2_)(OTf)_2_ (**1**, IMesH_2_=1,3‐dimesitylimidazolin‐2‐ylidene, OTf=CF_3_SO_3_) in the acyclic diene metathesis (ADMET) polymerization of 6‐hydroxy‐1,10‐undecadiene (**M1**)^6^ and diphenylbis(pent‐4‐en‐1‐yl)silane (**M2**) at 80 °C and 20 mbar,^7^ resulting in the formation of high molecular weight poly‐**M1** (*M_n_*=11 000 g mol^−1^, *Đ*=2.0, 16 % *trans*) and poly‐**M2** (*M_n_*=433 000 g mol^−1^, *Đ*=1.8, 77 % *trans*), both in 95 % isolated yield. Encouraged by these results, we set out to explore whether the potentially more active cationic catalysts also display enhanced stability in the presence of protic groups. This seemed not unlikely since the pentacoordinate complexes have been identified as precursors to the actual olefin metathesis‐active four coordinate cationic catalysts.4a We particularly tried to identify the structural motifs responsible for their unique properties. Although NHC‐free cationic molybdenum alkylidene complexes have successfully been prepared over a decade ago,^8^ their moderate stability and low reactivity led to their abandonment quickly after. We started our investigations by revisiting these compounds in form of **2 a**–**c** (Figure 1) bearing electron‐withdrawing terphenoxide and alkoxide ligands or a basic pyrrolide. Complex **2 b** features a strongly electron‐donating, nucleophilic pyrazole, which stabilizes the cationic metal center considerably more than (previously reported) coordinated THF or lutidine.^9^ Even though these compounds are stable in dry solvents (**2 b** at least for two weeks in CD_2_Cl_2_ at room temperature), they immediately react with air, moisture and 2‐PrOH to unknown species that no longer contain an alkylidene ligand.

**Figure 2:**
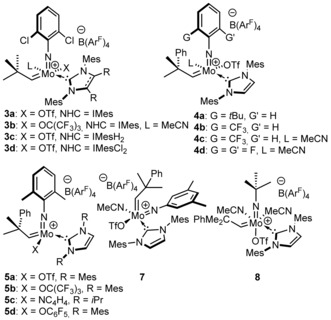
Cationic molybdenum imido alkylidene NHC catalysts. IMes=1,3‐dimesitylimidazol‐2‐ylidene, IMesCl_2_=1,3‐dimesityl‐4,5‐dichloroimidazol‐2‐ylidene, NC_4_H_4_=pyrrolide, 5‐*i*Pr=1,3‐di*iso*propylimidazol‐2‐ylidene.

We already have shown that cationic molybdenum imido alkylidene NHC complexes are highly active catalysts in various olefin metathesis reactions.4b, 10 Here we prepared a wide range of complexes with the general formula [Mo(*N*R′)(CHCMe_2_R)(NHC)(X)][B(Ar^F^)_4_] (Figure 2, X=anionic ligand). The emphasis was placed on monotriflate species since they exhibit high reactivity and are prepared in a straightforward manner from corresponding bistriflate complexes by reaction with Na(BAr^F^)_4_ and elimination of NaOTf (usually in >90 % isolated yield).

**Figure 3:**
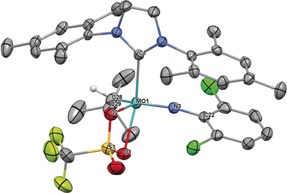
Single crystal X‐ray structure of [Mo(*N*‐2,6‐Cl_2_‐C_6_H_3_)(CHCMe_3_)(IMes)(OTf)][B(Ar^F^)_4_], **3 a**. Relevant bond lengths [pm] and angles [°]: Mo–N3 173.4, Mo–C28 186.7, Mo–O2 217.9, Mo–C1 218.2, Mo–O1 226.9, Mo–S1 280.2; N3‐Mo‐C28 104.81, N3‐Mo‐O2 136.15, C28‐Mo‐O2 115.56, N3‐Mo‐C1 99.87, C28‐Mo‐C1 101.72, O2‐Mo‐C1 88.45, N3‐Mo‐O1 98.45, C28‐Mo‐O1 94.12, O2‐Mo‐O1 63.52, C1‐Mo‐O1 151.75, C22‐N3‐Mo 160.5, C29‐C28‐Mo 144.3. One CH_2_Cl_2_ molecule, the anion and hydrogens have been omitted for clarity. Thermal ellipsoids are set at a 50 % probability level.^30^

Subsequent salt metathesis of the remaining triflate provides access to other cationic monoalkoxide catalysts in high yield, usually in a one‐pot reaction from the bistriflate precursor. We employed various imido ligands, including the previously not reported 2,6‐difluorophenylimido ligand in **4 d** (Scheme S1, Supporting Information). Additional variations comprised different NHC ligands and the presence of coordinating acetonitrile. Coordination of polar molecules to cationic monotriflate species can easily be probed by ^19^F NMR spectroscopy, since cationic metal centers are coordinated in a η^2^‐fashion by the triflate ligand in case no other polar molecule is present. This results in chemical shifts of *δ*≈−73.5 ppm in the ^19^F NMR spectrum. The single crystal X‐ray structure of **3 a** (Figure 3) confirms this binding situation. **3 a** crystallizes in the monoclinic space group *P*2_1_/*n* with *a*=1892.45(17) pm, *b*=1653.39(15) pm, *c*=2273.5(2) pm, *α*=*γ*=90°, *β*=94.994(3)° (*Z*=4). The geometry at the metal is distorted square pyramidal (*τ*_5_=0.26)^11^ with the alkylidene in *syn* orientation forming the apex (Mo−C28=186.7 pm). Notably, the triflate ligand is bound unsymmetrically, as evidenced by the Mo−O1 bond being weaker compared to Mo−O2 (226.9 vs. 217.9 pm). This effect is likely caused by the NHC, which is coordinated fairly *trans* to O1 of the triflate. However, in solution, broad signals for both, the triflate and alkylidene ligand in ^1^H and ^19^F NMR spectra imply a degree of fluctuation in the triflate‐metal bonding.

**Figure 4:**
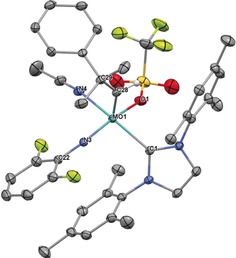
Single crystal X‐ray structure of [Mo(*N*‐2,6‐F_2_‐C_6_H_3_)(CHCMe_2_Ph)(IMes)(OTf)(MeCN)][B(Ar^F^)_4_], **4 d**. Relevant bond lengths [pm] and angles [°]: Mo–N3 173.1, Mo–C28 187.1, Mo–O1 208.9, Mo–N4 217.2, Mo–C1 218.3; N3‐Mo‐C28 102.03, N3‐Mo‐O1 151.93, C28‐Mo‐O1 105.03, N3‐Mo‐C1 97.06, C28‐Mo‐C1 102.72, N4‐Mo‐C1 160.29, N3‐Mo‐O1 151.93, C28‐Mo‐O1 105.03, N4‐Mo‐O1 81.88, C1‐Mo‐O1 84.21, C22‐N3‐Mo 166.38, C29‐C28‐Mo 143.56. The anion and hydrogens have been omitted for clarity. Thermal ellipsoids are set at a 50 % probability level.^30^

Other potential neutral ligands, like acetonitrile, coordinate *trans* to the NHC and consequently break up η^2^ coordination of the triflate resulting in chemical shifts of *δ*≈−75 ppm and below. This can be observed in the single crystal X‐ray structure of **4 d** (Figure 4). Coordination of acetonitrile *trans* to the NHC is also observed with monodentate ligands, as is seen in the structures of **5 d** and its acetonitrile adduct (Figure S128,129, Supporting Information). **4 d** crystallizes in the triclinic space group *P‐1* with *a*=1279.17(5) pm, *b*=1815.54(7) pm, *c*=1860.54(8) pm, *α*=64.059(2)°, *β*=70.333(2)°, *γ*=73.654(2)° (*Z*=2). The molybdenum center has a slightly distorted square pyramidal coordination sphere (*τ*_5_=0.14) with the alkylidene in the apex. Here the Mo−O(triflate) bond is considerably shorter (208.9 pm) compared to the one in **3 a** (217.9 pm) with the triflate being *trans* to the imido ligand.

In contrast to **2 a**–**c**, most cationic NHC‐stabilized complexes shown here do not react readily with protic groups. NMR measurements (Figure S90–92) suggest that complex **3 a** is coordinated by 2‐PrOH as indicated by a shift of the triflate resonance in the ^19^F NMR from *δ*=−73.7 ppm (η^2^‐bound) to −74.8 ppm (η^1^‐bound) and a shift of the alkylidene proton in the ^1^H NMR from *δ*=12.59 to 13.02 ppm. The broadening of the signals in the presence of 0.5 equiv 2‐PrOH indicates that coordination is highly reversible. Likewise, if 2 equiv of 2‐PrOH are present, several broad ^1^H NMR signals between *δ*=3.5 and 4.7 ppm suggest that 2‐PrOH molecules exchange readily.

**Figure 5:**
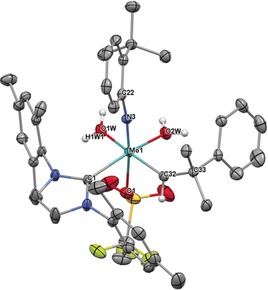
Single crystal X‐ray structure of [Mo(*N*‐2‐*t*Bu‐C_6_H_4_)(CHCMe_2_Ph)(IMes)(OTf)(H_2_O)_2_][B(Ar^F^)_4_], **4 a⋅H_2_O**. Relevant bond lengths [pm] and angles [°]: Mo–N3 173.2, Mo–C32 191.8, Mo–O2W 219.2, Mo–C1 224.1, Mo–O1 226.8, Mo–O1W 232.11; N3‐Mo‐C32 99.47, N3‐Mo‐O2W 99.39, C32‐Mo‐O2W 88.23, N3‐Mo‐C1 95.05, C32‐Mo‐C1 96.37, O2W‐Mo‐C1 163.92, N3‐Mo‐O1 172.82, C32‐Mo‐O1 87.69, O2W‐Mo‐O1 81.21, C1‐Mo‐O1 83.58, N3‐Mo‐O1W 99.06, C32‐Mo‐O1W 154.92, O2W‐Mo‐O1W 72.15, C1‐Mo‐O1W 98.69, O1‐Mo‐O1W 74.25, C33‐C32‐Mo 142.38, C22‐N3‐Mo 172.02. One CH_2_Cl_2_ molecule, anion and hydrogens have been omitted for clarity. Thermal ellipsoids are set at a 50 % probability level.^30^

Recently, we have shown that tailored six‐coordinate neutral Mo‐imido alkylidene NHC catalysts can serve as latent pre‐catalysts in the polymerization of dicyclopentadiene (DCPD) and are stable to air for at least 12 h.^12^ Here, we tested the air stability of our cationic complexes for a minimum of 12 hours. While those complexes that contained monodentate or basic ligands decomposed during that time, the monotriflate complexes did not. However, the monotriflate complexes proved to be hygroscopic, absorbing varying amounts of water (Figure S93–115). Thus, complexes **3 a** and **3 c** contained approx. 2–3 equiv of water after 12 h. Accordingly, new alkylidene species, which we attribute to the corresponding water‐coordinated complexes, are observed. In addition, the appearance of imidazolium signals (≈10 %, relative to the anion as judged by NMR) and free triflate implied slow decomposition. Similar observations were made for **5 a** (≈10 % imidazolium). On the other hand, complex **3 d**, which features the less basic, air stable 1,3‐dimesityl‐4,5‐dichloroimidazol‐2‐ylidene (IMesCl_2_) NHC ligand,^13^ did not show any signs of protonation. In almost the same manner, **4 c** and complexes **7** and **8**, containing acetonitrile, did not decompose. Notably, complex **8** lost one acetonitrile in favor of water. **4 d** with the 2,6‐difluorophenylimido ligand remained virtually unchanged after 12 h. **4 a** also did not show any decomposition even after 24 hours in air. The solid material contained ≈0.5 equiv of water after this time. In solution, the broad ^1^H and ^19^F NMR signals for the triflate and the alkylidene ligand imply a substantial degree of fluctuation in the triflate‐metal bonding. We attribute this to the reversibility of water coordination. To finally prove the inherent stability of these catalysts against moisture, we were able to crystallize **4 a** as the *dihydrate* complex (Figure 5). **4 a⋅H_2_O** crystallizes in the triclinic space group *P‐1* with *a*=1544.78(14) pm, *b*=1631.48(15) pm, *c*=1722.14(16) pm, *α*=96.014(5)°, *β*=110.634(5)°, *γ*=97.988° (*Z*=2). The molybdenum center adopts an octahedral coordination sphere with the triflate being *trans* to the imido ligand. The Mo−NHC bond is comparably long (224.1 pm). The water molecules coordinate *trans* to the NHC and the alkylidene ligand. With the NHC causing the stronger *trans*‐effect, the Mo−O(water) bond is significantly longer than the Mo−O(water) bond *trans* to the alkylidene (232 vs. 219 pm).

**Scheme 1:**
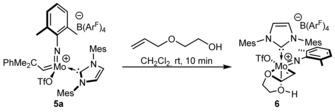
Formation of [Mo(*N*‐2,6‐Me_2_‐C_6_H_4_)(CHCH_2_O(CH_2_)_2_OH) (IMes)(OTf)][B(Ar^F^)_4_] (**6**).

In the solid state, the more tightly bound water molecule shows a weak interaction between one of its hydrogens and one of the oxygens of the triflate ligand and is consequently slightly rotated around the C32‐Mo‐O1W axis. The distance of the hydrogen atom tilted towards the triflate ligand and the oxygen is 263.1 pm and thus markedly shorter than the other H(water)−O(triflate) distances, which are around 330 pm for the second water molecule (*trans* to the NHC). To learn about the stability of cationic monotriflate NHC complexes towards hydroxyl groups in the presence of double bonds, **5 a** was reacted with 1.5 equiv 2‐allyloxyethanol (Scheme 1). After a few minutes the stable, crystalline alkylidene complex [Mo(*N*‐2,6‐Me_2_‐C_6_H_3_)(CHCH_2_O(CH_2_)_2_OH)(IMes)(OTf)][B(Ar^F^)_4_] **6** formed quantitatively.

**Figure 6:**
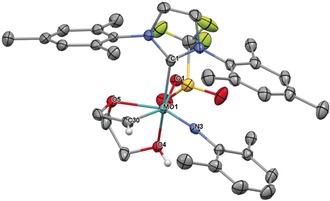
Single crystal X‐ray structure of **6**. Selected bond lengths [pm] and angles [°]: Mo–N3 171.8, Mo–C30 192.2, Mo–O4 217.7, Mo–O1 218.9, Mo–C1 220.5, Mo–O5 229.3; N3‐Mo‐C30 98.63, N3‐Mo‐O4 94.36, C30‐Mo‐O4 97.11, N3‐Mo‐O1 117.38, C30‐Mo‐O1 143.97, O4‐Mo‐O1 79.88, N3‐Mo‐C1 96.24, C30‐Mo‐C1 95.16, O4‐Mo‐C1 162.38, O1‐Mo‐C1 82.75, N3‐Mo‐O5 157.22, C30‐Mo‐O5 64.87, O4‐Mo‐O5 73.49, O1‐Mo‐O5 80.10, C1‐Mo‐O5 100.64, C22‐N3‐Mo 168.47. The anion, hydrogens (except H on C30 and O4) and one Pr_2_O molecule coordinating to the hydrogen on O4 have been omitted for clarity. Thermal ellipsoids are set at a 50 % probability level.^30^

In **6**, the hydroxyl group at the alkylidene ligand is coordinated to molybdenum (Figure 6). This implies that the metallacyclobutanes of these complexes are stable towards hydroxyl groups, which makes productive metathesis possible. **6** crystallizes in the triclinic space group *P* with *a*=1333.10(6) pm, *b*=1734.49(7) pm, *c*=1819.79(9) pm, *α*=78.865(2)°, *β*=88.052(2)°, *γ*=88.528(2)° (*Z*=2). The allyl ether oxygen forms a four‐membered ring by coordinating *trans* to the imido ligand. The hydroxyl group is *trans* to the NHC. Both in the solid state and in solution the alkylidene ligand is in the *anti*‐configuration (Mo−C30=192 pm) (*δ*=13.38 ppm, ^1^ *J*_CH_=167 Hz, CDCl_3_).

**Scheme 2:**
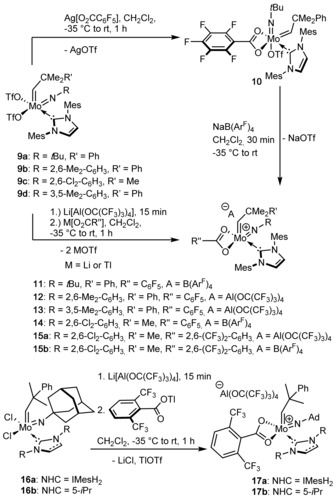
Synthesis of cationic monocarboxylate species **11**–**15** from bistriflates **9 a**–**d** 12, 15 via mixed monotriflate/monocarboxylate complex **10** or in situ generation of cationic monotriflate species and subsequent salt metathesis. Synthesis of cationic monocarboxylate complexes **17 a**,**b** from dichloride NHC alkylidenes **16 a**,**b**.^16^ Ad=adamantyl.

Next, we were interested whether the stability of cationic molybdenum imido alkylidene NHC monotriflate species could be further enhanced by introducing other bidentate ligands. We therefore prepared a series of carboxylate‐ligated cationic complexes, that were expected to fit our requirements (Scheme 2). Previously, dicarboxylate‐based molybdenum olefin metathesis catalysts have successfully been prepared and employed in the regioselective cyclopolymerization of α,ω‐diynes.^14^ However, because of their saturated coordination sphere, these compounds were rather poor catalysts for other metathesis reactions. Until now, monocarboxylate species of Schrock‐type catalysts have not been reported. However, the monotriflate‐monocarboxylate species Mo(*N*‐*t*Bu)(CHCMe_2_Ph)(IMes)(O_2_CC_6_F_5_)(OTf) **10** can readily be prepared from **9 a** 12 (Scheme 2). Subsequent transformation into the cationic monocarboxylate species **11** is facile and high‐yielding employing NaB(Ar^F^)_4_.

**Figure 7:**
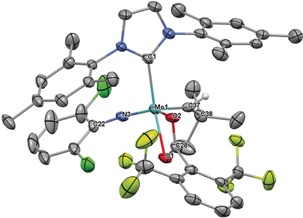
Single crystal X‐ray structure of [Mo(*N*‐2,6‐Cl_2_‐C_6_H_3_)(CHCMe_3_)(IMes)(2,6‐(CF_3_)_2_‐C_6_H_3_)][B(Ar^F^)_4_], **15 b**. Selected bond lengths [pm] and angles [°]: Mo–N3 172.5, Mo–C37 184.1, Mo–O2 218.6, Mo–C1 218.9, Mo–O1 219.1, Mo–C28 255.3; N3‐Mo‐C37 103.4, N3‐Mo‐O2 142.33, C37‐Mo‐O2 109.69, N3‐Mo‐C1 101.58, C37‐Mo‐C1 101.41, O2‐Mo‐C1 89.29, N3‐Mo‐O1 100.69, C37‐Mo‐O1 95.22, O2‐Mo‐O1 59.42, C1‐Mo‐O1 148.17, C22‐N3‐Mo 165.1, C38‐C37‐Mo=145.9. Thermal ellipsoids are set at a 50 % probability level. The B(Ar^F^)_4_ anion and hydrogens except for H on C37 have been omitted for clarity.^30^

The synthesis of cationic carboxylates can be accomplished in an even more straightforward manner in a one‐pot reaction of the bistriflate or dichloride precursors with NaB(Ar^F^)_4_ or LiAl(OC(CF_3_)_3_)_4_, respectively,^17^ followed by salt metathesis of the triflate or chloride, usually in ≈90 % yield. It is interesting to note, that we did not observe bridged dimers as reported for neutral carboxylate species with small ligands,^18^ probably due to the repulsion of the cationic metal centers. The single crystal X‐ray structure of complex **15 b** confirms the monomeric structure (Figure 7). Solution NMR experiments and the solid‐state structure suggest a significantly more tightly bound carboxylate ligand, compared to the triflate in **3 a**. This results in sharp resonances in both the ^1^H and ^19^F NMR spectra and an almost symmetrically bound carboxylate ligand (Mo−O1=219.1 pm, Mo−O2=218.6 pm) and a Mo−C28 (carboxylate carbon) distance of 255.3 pm in the solid state (compared to Mo−S1=280.2 pm, in **3 a**). The overall structure is a slightly distorted square pyramid (*τ*_5_=0.1) with the alkylidene ligand forming the apex (Mo−C37=184.1 pm) and one of the carboxylate oxygens roughly *trans* to the NHC.

As anticipated and evidenced by the structure of **15 b**, the carboxylate complexes exhibit remarkable air stability. **11**, **13**, **15 a**, **17 a** and **17 b** were left open to air for 5 days. After this time only **17 b** with the small 1,3‐di*iso*propylimidazol‐2‐ylidene ligand decomposed. All other complexes did not show any sign of decomposition and remained virtually unchanged. Also, no water absorption was observed (Figure S116–119). This shows that, beside electronic stabilization, steric protection by the NHC ligand seems to play a crucial role in the prevention of decomposition in air. In addition to that, more tightly bound bidentate ligands are a key factor for stability.

**Scheme 3:**
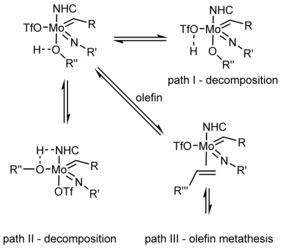
Proposed initial decomposition reactions competing with olefin metathesis.

Next, we subjected our catalysts to several benchmark olefin metathesis reactions with substrates containing the alcohol functionality. Table 1 contains the corresponding maximum turnover numbers (TON_max_) for catalysts **3**–**8** and the Schrock‐type catalysts Mo(*N*‐2,6‐Me_2_‐C_6_H_3_)(CHCMe_2_Ph)(OC(CF_3_)_3_)_2_ **SF9** and **2 a**–**c**, which were employed for comparison. Several important observations were made: 1) Electron‐withdrawing imido ligands increase productivity. 2) X‐type ligands with lower p*K*_a_ values for the corresponding acid, increase productivity. 3) In X‐type ligands with comparable p*K*_a_ values, higher steric demand is more favorable (**5 b** vs. **5 d**). 4) The IMes ligand seems to be the best choice, however, it is questionable that only its donor strength is the decisive measure. 5) Coordinated acetonitrile does not play a significant role in reactivity. Results for the benchmark reactions for carboxylate substituted catalysts are summarized in Table 2. We essentially observed the same trends as in Table 1. Catalysts with electron‐withdrawing imido ligands outperform more basic imido ligands. Pentafluorobenzoate is favored over the slightly more basic 2,6‐bis(trifluoromethyl)benzoate but is not favored over the triflate ligand in terms of reactivity. These findings are counterintuitive since one might anticipate that substitution of a cationic metal alkylidene with electron‐withdrawing substituents would further enhance the electrophilic character and thus lead to more active but also more unstable catalysts. However, while electrophilicity is indeed enhanced, the propensity of the catalysts to decompose in the presence of protic functional groups is reduced. Our data suggest that the predominant decomposition pathway is initiated by the coordination of the protic group to the metal center and subsequent proton transfers. Coordination always occurs *trans* to the NHC. This is supported by the single crystal X‐ray structures reported here and several other already reported crystal structures of similar compounds,4c recently published mechanistic studies from our group^19^ and mechanistic studies for MAP (monoalkoxide pyrrolide) type Schrock‐catalysts, which indicate that coordination occurs preferably *trans* to the strongest σ‐donor ligand.^20^ Hence, the NHC dictates the coordination of molecules to the metal center. This results in a maximum distance between the NHC and the protic groups as compared to *cis* coordination, which in turn makes protonation of the NHC less probable. Recently, we reported on oxo‐bridged dimers that formed in the reaction of a Mo‐imido alkylidene NHC complex containing a basic aryloxide with water.^16^ Moreover, it was shown that protonation of Mo(*N*‐Ad)(CHCMe_2_Ph)(NHC)(pyrrolide)(aryloxide) selectively eliminates pyrrole.^21^ Also, other examples in which pyrrole was eliminated from metal imido alkylidene NHC bispyrrolide complexes or cationic metal imido alkylidene NHC monopyrrolide complexes were reported.^15^ All this evidence indicates that X‐type ligands are the first and preferred target for proton transfer. Notably, so far we have not observed direct protonation of the alkylidene‐ or the imido ligand, which theoretically would result in amido alkylidene or imido alkyl species. Indeed, the imido alkylidene motif is remarkably stable considering the fact that it is generated via protonation of a bisimido species, often with excess acid or alcohols.^22^ Amido alkylidyne complexes, however, seem to be close in energy and form reversibly from imido alkylidenes by formal proton transfer.^16^, ^23^ Data published by Schrock et al. also suggest that the alkylidene ligand is protonated preferably by strong acids in case two alkyl ligands (strong σ donors) are present, but not in the presence of one alkyl and one alkoxide ligand.^24^ In the same report, it was surmised that Mo alkylidenes might be stable in alcohols under certain conditions. Therefore, we propose that if the most probable decomposition pathways are inhibited by ligands with low basicity, by steric hindrance and cationic metal centers (no bimolecular decomposition), olefin coordination and subsequent olefin metathesis is possible (Scheme 3).

**Table 1:** Maximum turnover numbers (TON_max_) for the homometathesis of hydroxyl‐substituted substrates with cationic NHC‐type catalysts **3**–**8** and the Schrock‐type catalysts **SF9** and **2 a**–**c**.

	**3 a**	**3 b**	**3 c**	**3 d**	**4 a**	**4 b**	**4 c**	**4 d**	**5 a**	**5 b**	**5 d**	**7**	**8**	**SF9**	**2 a**	**2 b**	**2 c**
4‐penten‐1‐ol^[a]^	4000 (1100^[c]^)	2900	3300	3000	3000	3300	3300	4000	2100	1100	600	1900	2000	100	0	0	0
5‐hexene‐1‐ol^[a]^	2300 (500^[c]^)	500	500	150	100	1200	1500	2300	2000	100	0	1600	0	0	0	0	0
7‐octene‐1‐ol^[a]^	3200 (300^[c]^)	200	0	0	0	0	0	0	0	0	0	0	0	0	0	0	0
2‐allylphenol^[b]^	3800 (0^[c]^)	900	3300	3800	0	400	600	2900	100	0	0	1300	0	0	0	0	0

[a] CH_2_Cl_2_, 2 h, room temperature, 3 m in substrate, cat:substrate 1:4000, internal standard for GC‐MS dodecane. [b] Internal standard for GC‐MS: cyclooctane. [c] Reaction in 2‐PrOH.

**Table 2:** Maximum turnover numbers (TON_max_) for the homometathesis of hydroxyl‐substituted substrates with cationic carboxylate NHC catalysts **11**–**17**.

	**11**	**12**	**13**	**14**	**15 a**	**17 a**
4‐penten‐1‐ol^[a]^	2500	700	1500	3100	2800	1500
5‐hexene‐1‐ol^[a]^	0	0	200	750	550	0
7‐octene‐1‐ol^[a]^	0	0	0	200	150	0
2‐allylphenol^[b]^	0	50	500	3300	2300	0

[a] CH_2_Cl_2_, 2 h, room temperature, 3 m in substrate, cat:substrate 1:4000, internal standard for GC‐MS dodecane. [b] Internal standard for GC‐MS: cyclooctane.

The use of ligands with low basicity, e.g., of perfluorinated alkoxides, triflate and carboxylates, impedes proton transfer from the alcohol. Notably, while coordination of an alcohol to the cationic metal center will certainly increase its acidity, p*K*_a_ is still considered a useful tool for choosing potentially suitable ligands. This low‐basicity concept is also in line with reports by Hohloch et al. on the protolytic stability of Mo‐imido NHC complexes.^25^ We chose triflate (p*K*_a,HOTf_(H_2_O)=−12),^26^ nonafluoro‐*tert*‐butoxide (p*K*_a,HOR_(H_2_O)=5.4, R=C(CF_3_)_3_)^26^ and pentafluorobenzoate (p*K*_a,HOOCR_(H_2_O)=1.48, R=C_6_F_5_)^27^ as the most promising X‐type ligands. In view of its excellent σ‐donating properties (TEP=2050.5 cm^−1^), we also decided to employ 1,3‐dimesitylimidazol‐2‐ylidene as the NHC‐type ligand.^28^ The ability to delocalize the cationic charge on molybdenum over its aromatic system led us to believe that it would engage in strong metal carbene bonds and provide more productive catalysts. However, while IMes‐based **3 a** proved to be the most productive in our benchmark reactions, IMesCl_2_‐based **3 d** (TEP=2052.6 cm^−1^)^29^ was significantly more stable in air, which probably is a consequence of the lower basicity of the NHC. Then again, IMes‐based **4 d** with fluorinated imido ligand was robust and highly productive, which shows that careful consideration of all ligands is crucial. It became evident that the imido ligand has a huge impact on productivity (Table 1 and 2). Catalysts containing the more basic alkyl‐substituted imido ligands (**8**, **11**, **17 a**) displayed decreased reactivity compared to complexes with aromatic imido ligands. This was also observed earlier.^12^, ^15^, ^21^ To demonstrate that this is a reactivity issue, we mixed catalyst **11** with 10 equiv 10‐undecenoic acid and monitored the reaction by ^1^H NMR (Figure S122). Even after 80 minutes, most of the catalyst was unchanged, clearly showing that proton transfer to either ligand was very slow. After 8 hours, all catalyst initiated as indicated by the formation of CH=CH_2_CMe_2_Ph, but only little metathesis product was formed before decomposition took place (formation of the imidazolium species was verified by NMR). We therefore conclude that even basic imido ligands are not protonated readily in metal imido alkylidene NHC complexes, but that electron‐withdrawing imido ligands increase reactivity by increasing electrophilicity. Additionally, the formation of stable chelates as in **6** is a factor that lowers productivity. When comparing the triflate‐ to the nonafluoro‐*tert*‐butoxide or the carboxylate ligands in complexes **3 a**, **3 b**, **14**, and **15 a**, respectively, the triflate ligand outperforms the other in accordance with its weaker basicity. This is understandable when revisiting the crystal structure of **4 a⋅H_2_O** (Figure 5), where a weak interaction between one of the hydrogen atoms of water and a triflate ligand shows the way to X‐type ligand protonation. On top, in stark contrast to catalysts bearing weakly basic ligands, [Mo(*N*‐2,6‐Me_2_‐C_6_H_3_)(CHCMe_2_Ph)(NC_4_H_4_)(5‐*i*Pr)][B(Ar^F^)_4_] (**5 c**),^15^ with a basic pyrrolide ligand (p*K*_a,pyrrole_(H_2_O)=16.5) exhibited no productivity in any of the investigated reactions. Instead, when **5 c** was reacted with 5 equiv of 4‐penten‐1‐ol, the formation of pyrrole was immediately observed in the ^1^H NMR spectrum, accompanied by the emerging of a new alkylidene signal (*δ*=14.67 ppm, t, ^3^ *J*_HH_=5.7 Hz, CD_2_Cl_2_). We tentatively assign the signal to [Mo(*N*‐2,6‐Me_2_‐C_6_H_3_)(CH(CH_2_)_3_O)(5‐*i*Pr)][B(Ar^F^)_4_], in which the neophylidene ligand was replaced by substrate; the alkylidene proton therefore couples to the adjacent methylene (Figure S120, 121). The results in Table 1 and Table 2 not only demonstrate that the correct ligand combination is decisive for catalytic activity, but also that the choice of catalyst is crucial for a given substrate.

Overall, **3 a** with the favorable triflate and the electron‐withdrawing *N*‐2,6‐Cl_2_‐C_6_H_3_ imido ligand clearly stands out from all other investigated catalysts in terms of reactivity. We therefore used **3 a** in additional reactions with hydroxyl‐substituted substrates (Table 3) as in the ring‐opening cross metathesis (ROCM) of 2‐*endo*,3‐*endo*‐norborn‐5‐ene‐2,3‐dimethanol with allyltrimethylsilane (65 % isolated yield) and 1‐hexene (76 % isolated yield) at a catalyst loading of 1 mol % with respect to the NBE derivative. For both cross partners, the formation of two products was observed, formed by either one or two cross metathesis events. In both cases approximately equal amounts of double‐substituted product and mono‐substituted product were observed. **3 a** was also employed in the ROMP of 2‐*exo*‐norborn‐5‐enemethanol (87 % yield) and 2‐*exo*,3‐*exo*‐norborn‐5‐ene‐2,3‐dimethanol (68 % yield) in the polar protic solvent 2‐PrOH. Finally, we successfully employed **3 a** in cross metathesis reactions of 4‐penten‐1‐ol, 5‐hexen‐1‐ol, 7‐octen‐1‐ol and 2‐allylphenol in 2‐PrOH and still observed activity, although somewhat reduced compared to the TON in CH_2_Cl_2_ (Table 1, values in brackets). This is nonetheless remarkable since the olefinic substrate competes here in coordination with a large excess of alcohol.

**Table 3:** HM, RCM, ROCM and ROMP with catalyst **3 a**.

Method	Alcohol		TON_max_^[a]^	Yield [%]
HM	oleyl alcohol		1600	
RCM	1,6‐heptadiene‐4‐ol		1400	
ROMP in 2‐PrOH^[b]^	2‐*exo*‐norborn‐5‐enemethanol			87
	2‐*exo*,3‐*exo*‐norborn‐5‐enedimethanol			68

ROCM with 2‐*endo*,3‐*endo*‐norborn‐5‐enedimethanol^[c]^				
cross partner	conversion [%]^[d]^	yield [%]^[e]^	double‐subst. [%]	mono‐subst. [%]
1‐hexene	100	76	54	46
allyltrimethylsilane	100	65	50	50

[a] Room temperature, CH_2_Cl_2_, 4 h, 3 m in substrate, catalyst: substrate=1:4000. [b] Room temperature, 4 h, 2‐PrOH, cat.:substrate=1:200; polymers were insoluble in common organic solvents. [c] Room temperature, 2 h, cat:norborn‐5‐ene‐2,3‐dimethanol:olefin=1:100:1000, 0.3 m (substrate) in CHCl_3_, internal standard: dodecane, ethylene release. [d] Conversion of norborn‐5‐ene‐2,3‐dimethanol determined by GC‐MS. [e] Isolated yields, *cis*‐/*trans*‐mixture.

## Conclusion

In summary, we extended the concept of functional group‐tolerant Mo‐imido alkylidene NHC complexes. We have shown that these highly active catalysts clearly stand out from all known high oxidation state molybdenum and tungsten olefin metathesis catalysts in terms of (air) stability and in this regard rival ruthenium based alkylidene complexes. The applicability in HM, RCM, ROCM, ADMET polymerization and ROMP reactions with hydroxyl functionalized olefins even more narrows the gap between Mo‐ and competing Ru‐catalysts. We successfully demonstrated a correlation between ligand basicity and hydroxyl group tolerance in olefin metathesis employing cationic molybdenum imido alkylidene NHC complexes. Work on extending this unique reactivity to (cationic) W imido and W oxo alkylidene NHC complexes is under way.

## Conflict of interest

The authors declare no conflict of interest.

## Supporting information

As a service to our authors and readers, this journal provides supporting information supplied by the authors. Such materials are peer reviewed and may be re‐organized for online delivery, but are not copy‐edited or typeset. Technical support issues arising from supporting information (other than missing files) should be addressed to the authors.

Supplementary
