# Supplementary material for: Origin and Use of Hydroxyl Group Tolerance in Cationic Molybdenum Imido Alkylidene N‐Heterocyclic Carbene Catalysts
Source: Angew Chem Int Ed Engl. 2019 Dec 10;59(2):951–8. doi: 10.1002/anie.201913322 (PMC6972570; doi:10.1002/anie.201913322)
Supplement: Supplementary file 1 — Supplementary [file ANIE-59-951-s001.pdf]

Angewandte Chemie
Eine Zeitschrift der Gesellschaft Deutscher Chemiker

Supporting Information

Origin and Use of Hydroxyl Group Tolerance in Cationic Molybdenum Imido Alkylidene N-Heterocyclic Carbene Catalysts

*Roman Schowner, Iris Elser, Mathis Benedikter, Mohasin Momin, Wolfgang Frey, Tanja Schneck, Laura Stöhr, and Michael R. Buchmeiser**

anie_201913322_sm_miscellaneous_information.pdf

Table of Contents

General Information 3
Experimental Procedures 3
Synthesis of Organometallic Complexes 4
NMR Spectra of Organometallic Complexes 12
NMR Spectra of Organic Compounds 51
NMR Spectra of Stability Experiments 57
Single Crystal X-Ray Analyses 74
High Resolution Mass Spectra (ESI) 81
References 81

General Information

All reactions were performed under the exclusion of air and moisture by standard Schlenk techniques unless noted otherwise. Reactions involving metal complexes were performed in a nitrogen-filled glove box (MBraun Labmaster 130). Glassware was stored at 120 °C overnight and cooled in an evacuated antechamber. ^1H and ^{13}C NMR spectra were recorded on a Bruker Avance III 400 spectrometer at 400 and 100 MHz, respectively. Chemical shifts are reported in ppm from tetramethylsilane with the solvent resonance resulting from residual solvent protons ($CDCl_3$: 7.26 ppm, CD_2Cl_2 5.32 ppm) as reference.[1] Data are reported as follows: chemical shift, multiplicity (s = singlet, d = doublet, t = triplet, q = quartet, quint = quintet, sept = septet, br = broad, m = multiplet), coupling constants (Hz) and integration. Elemental analyses were carried out at the Institute of Inorganic Chemistry, University of Stuttgart, Germany. High-resolution mass spectra were recorded at the Institute of Organic Chemistry, University of Stuttgart, Germany. CH_2Cl_2, diethyl ether, toluene and pentane were dried by using an MBraun SPS-800 solvent purification system with alumina drying columns and stored over 4 Å Linde type molecular sieves. THF, benzene and DME were distilled from Na prior to use and stored over 4 Å Linde type molecular sieves and Selexsorb®. Deuterated solvents were filtered over activated alumina and stored over 4 Å Linde type molecular sieves inside the glove box. Reagents prepared according to the literature: 6-hydroxy-1,10-undecadiene,[2] DFTOH,[3] IMes,[4] $IMesCl_2$,[4] 1,3,5-trimethylpyrazole (TMP),[5] $NaB(Ar^F)_4$,[6] silver pentafluorobenzoate,[7] $LiAl(OC(CF_3)_3)_4$.[8] $LiOC(CF_3)_3$, $LiO_2C(C_6F_5)$ were prepared by treating the corresponding alcohol or acid with *n*-BuLi (in hexane) in diethyl ether at -35 °C. $TlO_2C(2,6\text{-}(CF_3)_2\text{-}C_6H_3)$ was prepared by treating the corresponding acid with 1 equiv. Thallium ethoxide in diethyl ether at -35 °C. 2-Allyloxyethanol (98%, Sigma Aldrich), 2-PrOH (99%, Acros), 4-penten-1-ol (99%, Sigma Aldrich), 5-hexen-1-ol (99%, TCI), 7-octene-1-ol (99%, TCI), oleyl alcohol (99+%, Alfa Aesar), 1,6-heptadiene-4-ol (97%, Acros), 2-*exo*-norborn-5-enemethanol (99+%, Sigma Aldrich), 2-*exo*-3-*exo*-norborn-5-enedimethanol (97%, Sigma Aldrich), 2-*endo*-3-*endo*-norborn-5-enedimethanol (98%, Sigma Aldrich), 1-hexene (99%, Acros), allyltrimethylsilane (99%, Acros), dodecane (99%, Acros) and cyclooctane (99%, Sigma Aldrich) were used as received. The following metal complexes were published earlier and prepared according to the literature procedures: Compounds **1**,[9] **2c**,[10] **4a**,[11] **4b**,[11] **5c**,[12] **5d**,[13] **7**,[11] **8**,[11] **9a**,[14] **9b**,[15] **9c**,[15] **9d**,[16] and **SF9**[17].

Experimental Procedures

General Procedure HM, CM: A catalyst stock solution was prepared (15 mg/mL in CH_2Cl_2 or 2-PrOH). The appropriate amount of substrate (cat: substrate = 1:4000, approximately 30-80 mg) and internal standard (2-4 drops, dodecane or cyclooctane) were dissolved in the appropriate amount of solvent (3 M solution of substrate in CH_2Cl_2 or 2-PrOH). A sample for GC-MS was withdrawn. Then, the catalyst solution (15 mg/mL, 1 equiv.) was added and the vials were closed with a cap which was pierced with a cannula for ethylene removal. The reaction mixtures were stirred at room temperature for two hours and were then analyzed via GC-MS.

General Procedure ROMP: The NBE-derivative (200 equiv., approx. 70 mg) was dissolved in 2-PrOH (0.3 mL) and **3** (1 equiv.) was dissolved in 2-PrOH (0.3 mL). The catalyst solution was added to the NBE-derivative solution and the mixture was stirred at room temperature for four hours. Then, the suspension was dropped into *n*-pentane and the resulting precipitated colorless polymer was separated via centrifuge and dried *in vacuo* overnight. All polymers were insoluble and were therefore not be subjected to further analysis.

General Procedure ADMET:
The monomers **M1** and **M2** (100 equiv., approx. 40-70 mg) used for the ADMET polymerization were pre-dried and distilled under N_2 atmosphere. Catalyst **1** was added (1 equiv.) at room temperature. The ADMET polymerization was carried out at a temperature of 80 °C in bulk for four days. By applying a high vacuum (20 mbar), volatile by-products (ethylene) were removed and the reaction equilibrium directed to the side of the products (polymers). After the indicated time period, the polymerization was terminated by precipitation in methanol. The suspension was centrifuged and decanted. The resulting polymer was dried *in vacuo*.
*Poly(***M1***):* **^{1}H NMR** (400 MHz, 100 °C, C2D2Cl4) δ = 5.41 (br s, 2H, CH), 3.55 (br s, 1H, CHOH), 1.99 (br s, 4H, CH2), 1.59 - 1.24 (m, 9H, CH2, OH). **^{13}C NMR** (101 MHz, 100 °C, $C_2D_2Cl_4$) δ = 130.7, 72.0, 37.3, 32.7, 25.8.
*Poly(***M2***):* **^{1}H NMR** (400 MHz, $CDCl_3$) δ = 7.53 - 7.46 (m, 4H, ArH), 7.45 - 7.30 (m, 6H, ArH), 5.82 - 5.72 (m, 2H, CH), 2.13 - 1.96 (m, 4H, CH_2), 1.63 - 0.78 (br s, 8H, CH_2). **^{13}C NMR** (101 MHz, $CDCl_3$) δ = 138.8, 135.0, 129.3, 127.9, 114.9, 37.8, 26.8, 23.4, 12.3.

General Procedure ROCM: A catalyst stock solution was prepared (15 mg/mL in CH_2Cl_2). The appropriate amount of 2-*endo*,3-*endo*-norborn-5-ene-2,3-dimethanol (100 equiv., approximately 80 mg), cross partner (1000 equiv.) and internal standard (2-4 drops, dodecane) were dissolved in the appropriate amount of $CHCl_3$ (0.3 M solution of 2-*endo*, 3-*endo*-norborn-5-ene-2,3-dimethanol). A sample for GC-MS was withdrawn. Then, the catalyst solution (15 mg/mL, 1 equiv.) was added and the vials were closed with a cap which was pierced with a cannula for ethylene removal. The reaction mixtures were stirred at room temperature for 2 hours and were then analyzed via GC-MS. The solutions were filtered over silica and purified via semi-preparative HPLC (ethyl acetate : heptane 1:10) to afford the pure mono- or di-substituted products (mixtures of *cis* and *trans*).
(3,5-bis(3-(trimethylsilyl)prop-1-en-1-yl)cyclopentane-1,2-diyl)dimethanol: **^{1}H NMR** (400 MHz, $CDCl_3$) δ = 5.45 - 5.23 (m, 4H), 3.71 (br s, 4H), 2.67 (m, 2H), 2.44 (br s, 2H), 2.35 (m, 2H), 1.98 (m, 2H), 1.41 (m, 4H), 1.29 (m, 1H), -0.01 (s, 18H). **^{13}C NMR** (100 MHz, $CDCl_3$) δ = 130.4, 127.3, 61.9, 48.3, 44.2, 38.4, 23.0, -1.8.

(3-(3-(trimethylsilyl)prop-1-en-1-yl)-5-vinylcyclopentane-1,2-diyl)dimethanol: **^{1}H NMR** (400 MHz, $CDCl_3$) δ = 5.87 (m, 1H), 5.44 (m, 1H), 5.25 (m, 1H), 3.71 (m, 4H), 2.72 (m, 2H), 2.59 (s, 2H), 2.39 (m, 2H), 2.00 (m, 2H), 1.44-1.29 (m, 4H), -0.01 (s, 9H). **^{13}C NMR** (100 MHz, $CDCl_3$) δ = 142.3, 131.9, 129.3, 116.9, 63.6, 63.4, 50.1, 49.8, 46.7, 45.8, 39.1, 24.7, -0.01.

(3,5-bis(hex-1-en-1-yl)cyclopentane-1,2-diyl)dimethanol: **^{1}H NMR** (400 MHz, $CDCl_3$) δ = 5.48 - 5.38 (m, 4H),3.71 - 3.65 (m, 4H), 2.72 - 2.65 (m, 2H), 2.48 (s, 2H), 2.40 - 2.33 (m, 2H), 2.02 - 1.95 (m, 5H), 1.38 - 1.31 (m, 9H), 0.89 (t, $^3J_{HH}$ = 7.1 Hz, 6H). **^{13}C NMR** (100 MHz, $CDCl_3$) δ = 131.8, 131.5, 61.8, 48.2, 43.8, 37.6, 32.4, 31.9, 22.4, 14.1.

(3-(hex-1-en-1-yl)-5-vinylcyclopentane-1,2-diyl)dimethanol: **^{1}H NMR** (400 MHz, $CDCl_3$) δ = 5.91 - 5.83 (m, 1H), 5.49 - 5.39 (m, 2H), 5.06 - 4.99 (m, 2H), 3.74 - 3.65 (m, 4H), 2.75 - 2.69 (m, 2H), 2.53 (s, 2H), 2.46 - 2.35 (m, 2H), 2.05 - 1.97 (m, 3H), 1.44 - 1.28 (m, 6H), 0.80 (t, $^3J_{HH}$ = 7.1 Hz). **^{13}C NMR** (100 MHz, $CDCl_3$) δ = 140.5, 131.7, 115.1, 61.7, 61.6, 48.2, 48.0, 44.9, 43.7, 37.0, 32.4, 31.9, 22.4, 14.11, 1.17.

Synthesis of Organometallic Complexes

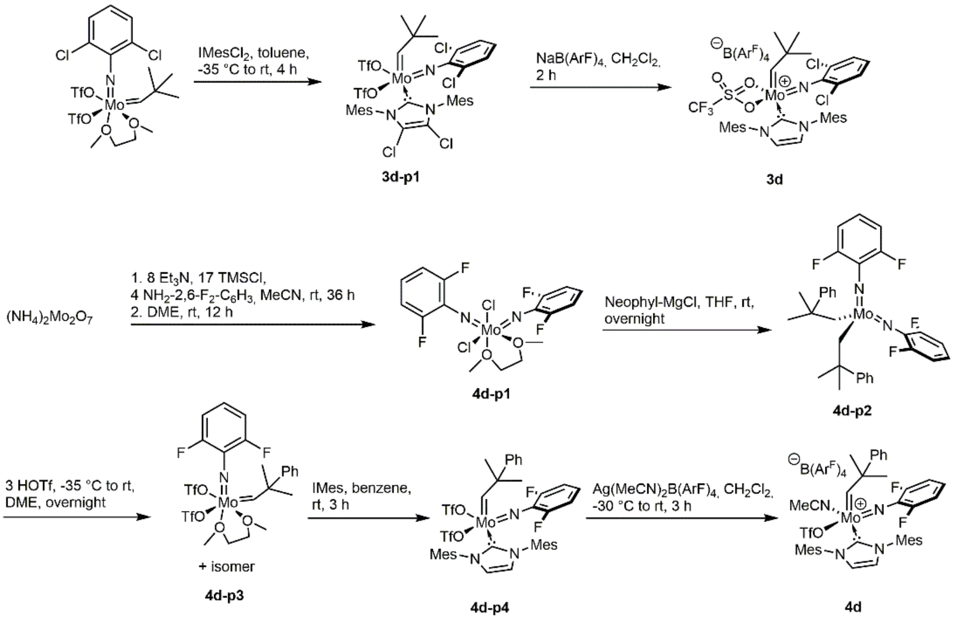

Scheme S1. Synthesis of **3d** and **4d** from previously not reported precursors.

[Mo(*N*-2,6-*i*Pr-C_6H_3)($CHCMe_2Ph$)(DFTO)($THF)_2$][B($Ar^F)_4$], **2a**

[Mo(*N*-2,6-*i*Pr-C_6H_3)($CHCMe_2Ph$)(Me_2pyr)($THF)_2$][B($Ar^F)_4$][10] (71.1 mg, 0.0472 mmol, 1 equiv) was dissolved in 3 mL THF and cooled to -35 °C. To the cold solution solid DFTOH (20.1 mg, 0.0472 mmol, 1 equiv) was added as a solid. The red solution turned yellow and was stirred at room temperature for 30 min. All volatiles were removed *in vacuo*. The dark residue was washed with 5 mL of pentane. The crude product was recrystallized from 2 mL of a 1:1 mixture of diethyl ether and pentane at -35 °C. The product precipitated as a crystalline yellow solid (61 mg, 73%). **^{1}H NMR** (400 MHz, CD_2Cl_2) δ = 13.09 (s, $^1J_{CH}$ = 118.4 Hz, 1H, Mo=CH), 7.72 (br m, 8H, *o*-Ar-B($Ar^F)_4$), 7.56 (br s, 4H, *p*-Ar-B($Ar^F)_4$), 7.45 (br m, 1H, Ar), 7.40 (m, 3H, Ar), 7.31 (m, 2H, Ar), 7.23 (m, 3H, Ar), 7.15 (m, 2H, Ar), 3.62 (m, 4H, THF), 3.48 (m, 4H, THF), 3.09 (sept, $^3J_{HH}$ = 6.8 Hz, 2H, CH-*i*Pr), 1.82 (m, 8H, THF), 1.76 (s, 6H, C<u>Me$_2$</u>Ph), 1.15 (d, $^3J_{HH}$ = 6.8 Hz, 12H, CH_3-*i*Pr). **^{13}C NMR** (101 MHz, CD_2Cl_2) δ = 298.9 (Mo=CH), 162.3 (q, $^1J_{CB}$ = 49.8 Hz, *ipso*-Ar-B($Ar^F)_4$), 160.4 (*ipso*-Ar-DFTO), 151.9 (*ipso*-Ar-Imido), 146.9 (*ipso*-Ar-CMe_2Ph), 144.9 (*o*-Ar-Imido), 135.4 (br m, *o*-Ar-B($Ar^F)_4$), 134.1 (*ipso*-C_6F_5), 130.9 (Ar-DFTO), 129.9 (Ar-DFTO), 129.4 (qq, $^2J_{CF}$ = 30.9 Hz, $^3J_{CB}$ = 2.5 Hz, *m*-Ar-B($Ar^F)_4$), 128.7 (Ar-Imido), 125.8 (Ar-CMe_2Ph), 125.13 (q, $^1J_{CF}$ = 272.4 Hz, CF_3-B($Ar^F)_4$), 125.08 (Ar-CMe_2Ph), 122.3 (*o*-Ar-DFTO), 118.0 (sept, $^3J_{CF}$ = 3.9 Hz, *p*-Ar-B($Ar^F)_4$), 80.8 (OCH_2-THF), 57.7 (<u>C</u>Me$_2$Ph), 30.3 (C<u>Me$_2$</u>Ph), 28.9 (CH-*i*Pr), 26.3 (CH_2-THF), 24.7 (CH_3-*i*Pr), Resonances of fluorinated aromatic carbons were not observed. **^{19}F NMR** (376 MHz, CD_2Cl_2) δ = -62.90 (br s, 24F, B($Ar^F)_4$), -138.40 (d, J_{FF} = 25.2 Hz, 2F, DFTO), -138.92 (d, J_{FF} = 25.2 Hz, 2F, DFTO), -151.36 (m, 1F, *p*-F-DFTO), -153.14 (m, 1F, *p*-F-DFTO), -159.61 (m, 2F, DFTO), -160.78 (m, 2F, DFTO). **Elemental analysis** (%) calcd. for $C_{80}H_{60}BF_{34}MoNO_3$: C, 52.33; H, 3.29; N, 0.76. Found: C, 52.29; H, 3.41; N, 0.84.

[Mo(*N*-2,6-*i*Pr-C_6H_3)($CHCMe_2Ph$)(Me_2Pyr)(TMP)][B($Ar^F)_4$], **2b**

[Mo(*N*-2,6-*i*Pr-C_6H_3)($CHCMe_2Ph$)(Me_2pyr)($THF)_2$][B($Ar^F)_4$][10] (65 mg, 0.043 mmol, 1 equiv) was dissolved in 3 mL CH_2Cl_2. At room temperature 1,3,5-trimethylpyrazole (24 mg, 0.22 mmol, 5 equiv) was added as a solid and the mixture was stirred for 15 mins. The color changed from dark red to yellow. All volatiles were removed *in vacuo* and the dark yellow residue was washed with 10 mL pentane to obtain a yellow solid (61 mg, 96%). The product can be recrystallized from diethyl ether/pentane. **^{1}H NMR** (400 MHz, $CDCl_3$) δ = 14.08 (br, s, 1H, Mo=CH), 7.73 (br m, 8H, *o*-Ar-B($Ar^F)_4$), 7.55 (br s, 4H, *p*-Ar-B($Ar^F)_4$), 7.38 – 7.28 (m, 5H, Ar), 7.27 – 7.22 (m, 1H, Ar), 7.18 (d, $^3J_{HH}$ = 7.7 Hz, 2H, *m*-Ar-Imido), 6.24 (br s, 1H, CH-pyr), 6.19 (br s, 1H, CH-TMP), 5.80 (br s, 1H, CH-pyr), 3.88 –

2.75 (br, 5H, overlapping CH-*i*Pr and *N*-Me-TMP signals), 2.61 – 2.03 (br, 9H, overlapping Me-pyr and Me-TMP signals), 1.75 (br s, 6H, CMe2Ph), 1.59 (s, 3H, Me-TMP), 1.20 (br d, 12H, $^3J_{HH}$ = 6.9 Hz, CH_3-*i*Pr). **^{13}C NMR** (101 MHz, $CDCl_3$) δ = 333.6 (br s, Mo=CH), 162.1 (q, $^1J_{CB}$ = 49.7 Hz, *ipso*-Ar-B(Ar^F)4), 153.7, 151.3, 148.9, 146.9, 145.2, 142.7, 135.0 (br m, *o*-Ar-B(Ar^F)4), 129.9, 129.1, 128.9 (qq, $^2J_{CF}$ = 30.9 Hz, $^3J_{CB}$ = 2.5 Hz, *m*-Ar-B(Ar^F)4), 126.4, 124.8 (q, $^1J_{CF}$ = 272.3 Hz, CF_3-B(Ar^F)4), 124.6, 117.6 (sept, $^3J_{CF}$ = 3.9 Hz, *p*-Ar-B(Ar^F)4), 110.5, 104.7, 104.0, 61.4, 53.6 (CMe_2Ph), 37.3 (N-Me-TMP), 30.4 (CMe_2Ph), 30.1 (CMe_2Ph), 28.3, 25.0, 23.5, 18.2, 17.6, 15.0 (Me-pyr), 12.7 (Me-TMP). **^{19}F NMR** (376 MHz, $CDCl_3$) δ = -62.34 (br s, 24F, B(Ar^F)4). **Elemental analysis** (%) calcd. for $C_{66}H_{59}BF_{24}MoN_4$: C, 53.89; H, 4.04; N, 3.81. Found: C, 53.62; H, 4.17; N, 3.91. **HR-MS** ESI calcd. for $C_{34}H_{47}MoN_4^+$: 609.2849. Found: 609.2848.

[Mo(*N*-2,6-Cl_2-C_6H_3)($CHCMe_3$)(IMes)(OTf)][B(Ar^F)4], **3a**

Mo(*N*-2,6-Cl_2-C_6H_3)($CHCMe_3$)(IMes)($OTf)_2$[15] (120.4 mg, 0.130 mmol, 1 equiv) was dissolved in 5 mL CH_2Cl_2. At room temperature solid NaB(Ar^F)4 (114.9 mg, 0.130 mmol, 1 equiv) was added. The mixture was stirred for 30 min. Subsequently all solids were filtered off and the filtrate was reduced to dryness. The oily product was dissolved in 1 mL CH_2Cl_2 and filtered once more. The CH_2Cl_2 was removed *in vacuo* and the oily product was triturated with pentane until a yellow solid formed. Yield: 184 mg, 87%. **^{1}H NMR** (400 MHz, $CDCl_3$) δ = 12.59 (br s, 1H, Mo=CH), 7.70 (br m, 8H, *o*-Ar-B(Ar^F)4), 7.51 (br s, 4H, *p*-Ar-B(Ar^F)4), 7.38 (s, 2H, CH=CH-NHC), 7.21 (m, 3H, Ar), 6.98 (s, 2H, *m*-Ar-Mes), 6.82 (s, 2H, *m*-Ar-Mes), 2.22 (s, 6H, Me-Mes), 2.02 (s, 12H, Me-Mes), 1.08 (s, 9H, *t*Bu). **^{13}C NMR** (101 MHz, $CDCl_3$) δ = 328.9 (Mo=CH), 181.8 (NCN-NHC), 161.9 (q, $^1J_{CB}$ = 49.8 Hz, *ipso*-Ar-B(Ar^F)4), 149.4 (*ipso*-Ar-Imido), 142.1 (*ipso*-Ar-Mes), 135.5 (Ar-Mes), 135.0 (br m, *o*-Ar-B(Ar^F)4), 134.6 (Ar-Mes), 133.7 (*o*-Ar-Imido), 131.8 (Ar-Imido), 130.7 (*m*-Ar-Mes), 129.9 (*m*-Ar-Mes), 129.1 (qq, $^2J_{CF}$ = 30.9 Hz, $^3J_{CB}$ = 2.5 Hz, *m*-Ar-B(Ar^F)4), 128.6 (Ar-Imido), 126.09 (CH=CH-NHC), 124.7 (q, $^1J_{CF}$ = 272.5 Hz, CF_3-B(Ar^F)4), 117.6 (sept, $^3J_{CF}$ = 3.8 Hz, *p*-Ar-B(Ar^F)4), 51.9 (CMe_3), 30.1 (CMe_3), 21.1 (*p*-Me-Mes), 18.2 (*o*-Me-Mes), 17.9 (*o*-Me-Mes). **^{19}F NMR** (376 MHz, $CDCl_3$) δ = -62.46 (s, 24F, B(Ar^F)4), -73.63 (s, 3F, OTf). **Elemental analysis** (%) calcd. for $C_{65}H_{49}BCl_2F_{27}MoN_3O_3S$: C, 47.52; H, 3.01; N, 2.56. Found: C, 47.48; H, 3.23; N, 2.73.

[Mo(*N*-2,6-Cl_2-C_6H_3)($CHCMe_3$)(IMes)(OC($CF_3)_3$)(MeCN)][B(Ar^F)4], **3b**

A solution of [Mo(*N*-2,6-Cl_2-C_6H_3)($CHCMe_3$)(IMes)(OTf)][B(Ar^F)4] **3a** (100 mg, 0.061 mmol, 1 equiv.) in in 2 mL CH_2Cl_2 was quickly added to a solution of LiOC($CF_3)_3$ (14.7 mg, 0.061 mmol, 1 equiv.) in 2 mL of CH_2Cl_2 and a few drops of acetonitrile. The resulting solution was stirred overnight, and the solvent was removed *in vacuo*. The residue was triturated with pentane to give a yellow suspension. The pentane was decanted, and the residue dried *in vacuo*. CH_2Cl_2 was added and the resulting suspension was filtered through a pad of celite. The solvent was removed, and the residue was triturated with pentane, yielding the product as a yellow solid. Yield: 85 mg (81 %). **^{1}H NMR** (400 MHz, $CDCl_3$) δ = 13.52 (s, $^1J_{CH}$ = 120.2 Hz, Mo=CH), 7.70 (br m, 8H, *o*-Ar-B(Ar^F)4), 7.52 (br s, 4H, *p*-Ar-B(Ar^F)4), 7.19 (d, $^3J_{HH}$ = 8.1 Hz, 2H, *m*-Ar-Imido), 7.15 (s, 2H, CH=CH-NHC), 7.07 (t, $^3J_{HH}$ = 8.1 Hz, 1H, *p*-Ar-Imido), 6.99 (br s, 2H, *m*-Ar-Mes), 6.63 (br s, 2H, *m*-Ar-Mes), 2.26 (s, 6H, Me-Mes), 2.05 (s, 6H, Me-Mes), 1.99 (br s. 3H, MeCN), 1.96 (br s, 6H, Me-Mes), 0.88 (s, 9H, *t*Bu). **^{13}C NMR** (101 MHz, $CDCl_3$) δ = 332.0 (Mo=CH), 183.2 (NCN-NHC), 161.6 (q, $^1J_{CB}$ = 49.9 Hz, *ipso*-Ar-B(Ar^F)4), 148.8 (*ipso*-Ar-Imido), 141.4 (*ipso*-Ar-Mes), 135.6 (Ar-Mes), 135.4 (Ar-Mes), 134.9 (br m, *o*-Ar-B(Ar^F)4), 134.8 (*o*-Ar-Imido), 130.5 (*m*-Ar-Mes), 129.4 (Ar-Imido), 129.1 (qq, $^2J_{CF}$ = 31.5 Hz, $^3J_{CB}$ = 2.9 Hz, *m*-Ar-B(Ar^F)4), 128.5 (Ar-Imido), 125.7 (CH=CH-NHC), 124.7 (q, $^1J_{CF}$ = 272.5 Hz, CF_3-B(Ar^F)4), 121.2 (q, $^1J_{CF}$ = 291.6 Hz, CF_3-OC($CF_3)_3$), 117.6 (sept, $^3J_{CF}$ = 3.6 Hz, *p*-Ar-B(Ar^F)4), 85.4 (m, C($CF_3)_3$), 50.9 (CMe_3), 29.7 (CMe_3), 21.0 (*p*-Me-Mes), 18.1 (*o*-Me-Mes), 18.0 (*o*-Me-Mes), 2.3 (MeCN). **^{19}F NMR** (376 MHz, $CDCl_3$) δ = -62.43 (s, 24F, B(Ar^F)4), -71.61 (s, 9F, OC($CF_3)_3$). **Elemental analysis** (%) calcd. for $C_{70}H_{52}BCl_2F_{33}MoN_4O$: C, 47.51; H, 2.96; N, 3.17. Found: C, 47.71; H, 3.19; N, 3.20.

[Mo(*N*-2,6-Cl_2-C_6H_3)($CHCMe_3$)($IMesH_2$)(OTf)][B(Ar^F)4], **3c**

At room temperature solid NaB(Ar^F)4 (73.8 mg, 0.083 mmol) was added to a solution of Mo(*N*-2,6-Cl_2-C_6H_3)($CHCMe_3$)($IMesH_2$)($OTf)_2$[15] (77.5 mg, 0.083 mmol, 1 equiv.) in 6 mL of CH_2Cl_2. The vial was rinsed with an additional 4 mL of CH_2Cl_2 and the resulting suspension was stirred for five hours at room temperature. It was then filtered through a pad of celite and the solvent was evaporated *in vacuo*. The oily residue was triturated with pentane, which led to the formation of a yellow solid. The solid was filtered off and washed with pentane to yield the product as a yellow solid. Yield: 124 mg (91%). **^{1}H NMR** (400 MHz, $CDCl_3$) δ = 12.72 (s, $^1J_{CH}$ = 118.1 Hz, Mo=CH), 7.70 (br m, 8H, *o*-Ar-B(Ar^F)4), 7.51 (br s, 4H, *p*-Ar-B(Ar^F)4), 7.28 (m, 2H, *m*-Ar-Imido), 7.21 (m, 1H, *p*-Ar-Imido), 6.90 (br s, 2H, *m*-Ar-Mes), 6.79 (br s, 2H, *m*-Ar-Mes), 4.14 (m, 4H, CH_2-NHC), 2.27 (s, 12H, Me-Mes), 2.16 (s, 6H, Me-Mes), 1.08 (s, 9H, *t*Bu). **^{13}C NMR** (101 MHz, $CDCl_3$) δ = 336.5 (Mo=CH), 207.0 (NCN-NHC), 161.6 (q, $^1J_{CB}$ = 49.9 Hz, *ipso*-Ar-B(Ar^F)4), 148.6 (*ipso*-Ar-Imido), 140.5 (*ipso*-Ar-Mes), 136.3 (Ar-Mes), 135.8 (Ar-Mes), 134.9 (br m, *o*-Ar-B(Ar^F)4), 132.4 (*o*-Ar-Imido), 131.5 (*m*-Ar-Mes), 131.1 (*m*-Ar-Mes), 129.8 (Ar-Imido), 129.0 (qq, $^2J_{CF}$ = 31.5 Hz, $^3J_{CB}$ = 2.9 Hz, *m*-Ar-B(Ar^F)4), 128.3 (Ar-Imido), 124.7 (q, $^1J_{CF}$ = 272.5 Hz, CF_3-B(Ar^F)4), 119.1 (q, $^1J_{CF}$ = 319.0 Hz, CF_3-OTf), 117.7 (sept, $^3J_{CF}$ = 3.6 Hz, *p*-Ar-B(Ar^F)4), 52.0 (CH_2-NHC), 51.4 (CMe_3), 29.7 (CMe_3), 20.9 (*p*-Me-Mes), 18.5 (*o*-Me-Mes), 18.3 (*o*-Me-Mes). **^{19}F NMR** (376 MHz, $CDCl_3$) δ = -62.42 (s, 24F, B(Ar^F)4), -73.78 (s, 3F, OTf). **Elemental analysis** (%) calcd. for $C_{65}H_{51}BCl_2F_{27}MoN_3O_3S$: C, 47.47; H, 3.13; N, 2.55. Found: C, 47.73; H, 3.37; N, 2.60.

Mo(*N*-2,6-Cl_2-C_6H_3)($CHCMe_3$)($IMesCl_2$)($OTf)_2$, **3d-p1**

A cold (-35 °C) solution of $IMesCl_2$ (261 mg, 0.70 mmol) in 8 mL of toluene was added to a cold (-35 °C) solution of Mo(*N*-2,6-Cl_2-C_6H_3)($CHCMe_3$)(DME)($OTf)_2$[18] (500 mg, 0.70 mmol, 1 equiv.) in 25 mL of toluene. The mixture was stirred at room temperature for four hours during which time a yellow solid precipitated. The suspension was filtered and the solid was washed with 2 mL of toluene and 2 mL of pentane to yield the product as a yellow solid. Yield: 455 mg (65%). **^{1}H NMR** (400 MHz, CD_2Cl_2) δ = 12.95 (s, $^1J_{CH}$ = 120.0 Hz, Mo=CH), 7.27 (br m, 1H, *p*-Ar-Imido), 7.14 (br m, 2H, *m*-Ar-Imido), 7.01 (br s, 2H, *m*-Ar-Mes), 6.73 (br s, 2H, *m*-Ar-Mes), 2.24 (br s, 6H, Me-Mes), 2.11 (br s, 6H, Me-Mes), 2.09 (br s, 6H, Me-Mes), 1.14 (s, 9H, *t*Bu). **^{13}C NMR** (101 MHz, CD_2Cl_2) δ = 329.5

(Mo=CH), 185.5 (NCN-NHC), 149.6 (*ipso*-Ar-Imido), 142.0 (*ipso*-Ar-Mes), 141.9 (*ipso*-Ar-Mes), 136.9 (*o*-Ar-Mes), 136.6 (*o*-Ar-Mes), 132.7 (*p*-Ar-Mes), 131.0 (*m*-Ar-Mes), 130.6 (Ar-Imido), 129.5 (Ar-Imido), 128.3 (*m*-Ar-Mes), 127.9 (Ar-Imido), 122.1 (CCl-NHC), 119.63 (q, $^1J_{CF}$ = 319.5 Hz, CF_3-OTf), 119.55 (q, $^1J_{CF}$ = 318.5 Hz, CF_3-OTf), 50.7 (<u>C</u>Me3), 31.3 (C<u>Me3</u>), 21.4 (*p*-Me-Mes), 18.9 (*o*-Me-Mes), 18.9 (*o*-Me-Mes). **^{19}F NMR** (376 MHz, CD_2Cl_2) δ = -75.05 (q, J_{FF} = 3.7 Hz, 3F, OTf), -76.58 (q, J_{FF} = 3.8 Hz, 3F, OTf). **Elemental analysis** (%) calcd. for $C_{34}H_{35}Cl_4F_6MoN_3O_6S_2$: C, 40.94; H, 3.54; N, 4.21. Found: C, 40.99; H, 3.58; N, 4.22.

[Mo(*N*-2,6-Cl_2-C_6H_3)($CHCMe_3$)(IMesCl2)(OTf)][B(Ar^F)4], **3d**
A solution of Mo(*N*-2,6-Cl_2-C_6H_3)($CHCMe_3$)(IMesCl2)$(OTf)_2$ **3d-p1** (200 mg, 0.20 mmol) in 4 mL of CH_2Cl_2 was added to a suspension of NaB(Ar^F)4 (177 mg, 0.20 mmol, 1 equiv.) in 2 mL of CH_2Cl_2. The suspension was stirred at room temperature for two hours and filtered through a pad of celite. The solution was concentrated to 2 mL *in vacuo* and pentane was added. Upon storing the solution at -35 °C overnight, yellow crystals of the product formed. Yield: 293 mg (85%). **^{1}H NMR** (400 MHz, $CDCl_3$) δ = 12.68 (s, $^1J_{CH}$ = 119.0 Hz, Mo=CH), 7.74 (br m, 8H, *o*-Ar-B(Ar^F)4), 7.54 (br s, 4H, *p*-Ar-B(Ar^F)4), 7.26 (m, 2H, *m*-Ar-Imido), 7.20 (m, 1H, *p*-Ar-Imido), 7.03 (br s, 2H, *m*-Ar-Mes), 6.88 (br s, 2H, *m*-Ar-Mes), 2.23 (br s, 6H, Me-Mes), 2.05 (br s, 12H, Me-Mes), 1.10 (s, 9H, *t*Bu). **^{13}C NMR** (101 MHz, $CDCl_3$) δ = 331.4 (Mo=CH), 181.4 (NCN-NHC), 161.9 (q, $^1J_{CB}$ = 49.9 Hz, *ipso*-Ar-B(Ar^F)4), 149.5 (*ipso*-Ar-Imido), 143.2 (*ipso*-Ar-Mes), 136.2 (*o*-Ar-Mes), 135.2 (*o*-Ar-Mes), 135.0 (br m, *o*-Ar-B(Ar^F)4), 132.2 (*p*-Ar-Mes), 131.1 (Ar-Imido), 131.0 (*m*-Ar-Mes), 130.2 (Ar-Imido), 129.1 (qq, $^2J_{CF}$ = 31.5 Hz, $^3J_{CB}$ = 2.9 Hz, *m*-Ar-B(Ar^F)4), 128.6 (Ar-Imido), 124.7 (q, $^1J_{CF}$ = 272.5 Hz, CF_3-B(Ar^F)4), 122.9 (CCl-NHC), 119.0 (q, $^1J_{CF}$ = 320.2 Hz, OTf), 117.6 (sept, $^3J_{CF}$ = 3.6 Hz, *p*-Ar-B(Ar^F)4), 52.2 (<u>C</u>Me3), 30.1 (C<u>Me3</u>), 21.2 (*p*-Me-Mes), 18.3 (*o*-Me-Mes), 17.9 (*o*-Me-Mes). **^{19}F NMR** (376 MHz, $CDCl_3$) δ = -62.41 (s, 24F, B(Ar^F)4), -73.69 (s, 3F, OTf). **Elemental analysis** (%) calcd. for $C_{65}H_{47}BCl_4F_{27}MoN_3O_3S$: C, 45.61; H, 2.77; N, 2.45. Found: C, 45.53; H, 2.85; N, 2.51.

[Mo(*N*-2-*t*Bu-C_6H_4)($CHCMe_2Ph$)(IMes)(OTf)$(H_2O)_2$][B(Ar^F)4], **4a*H2O**
Complex **4a** was reported previously.[11] Crystallization of the dihydrate succeeded via a slightly altered procedure by employing non predried NaB(Ar^F)4 which contains approx. 2.5 equiv. of water.[6] A cold solution (-35 °C) of NaB(Ar^F)4 (28.8 mg, 0.031 mmol, 1 equiv.) in diethyl ether (1 mL) was slowly added to a cold solution of Mo(*N*-2-*t*Bu-C_6H_4)(IMes)($CHCMe_2Ph$)$(OTf)_2$[13] (30 mg, 0.031 mmol, 1 equiv.) in CH_2Cl_2 (1 mL). The solution was stirred at room temperature for three hours and the solvent was evaporated. The residue was dissolved in CH_2Cl_2 and filtered through a pad of celite. The product crystallized from a 1:1 mixture of CH_2Cl_2 and *n*-pentane (0.5 mL) at -35 °C over the course of a few days as yellow crystals.

[Mo(*N*-2-CF_3-C_6H_4)($CHCMe_2Ph$)(IMes)(OTf)(MeCN)][B(Ar^F)4], **4c**
A solution of Mo(*N*-2-CF_3-C_6H_4)($CHCMe_2Ph$)(IMes)$(OTf)_2$[11] (100 mg, 0.10 mmol) in 2 mL of CH_2Cl_2 was added to a suspension of NaB(Ar^F)4 (90 mg, 0.10 mmol, 1 equiv.) in 2 mL CH_2Cl_2 containing three drops of acetonitrile. The mixture was stirred for two hours at room temperature and the solvent was removed *in vacuo*. The residue was triturated with *n*-pentane which led to the formation of a yellow solid. The pentane was decanted and the solid was dried *in vacuo*. The residue was extracted with CH_2Cl_2 and filtered through pad of celite. *n*-Pentane was added to the solution and upon storage of the solution at -35 °C overnight, a yellow solid precipitated. The suspension was filtered and the solid washed with pentane to yield the product as a yellow solid. Yield: 90 mg (52 %). **^{1}H NMR** (400 MHz, $CDCl_3$) δ = 13.28 (s, $^1J_{CH}$ = 123.0 Hz, Mo=CH), 7.72 (br m, 8H, *o*-Ar-B(Ar^F)4), 7.52 (br s, 4H, *p*-Ar-B(Ar^F)4), 7.48 (m, 2H, *m*-Ar-Imido), 7.43 (m, 1H, *p*-Ar-Imido), 7.32 (m, 1H, *o*-Ar-Imido), 7.23 (s, 2H, CH=CH-NHC), 7.21 (m, 2H, Ar-CMe_2Ph), 7.14 (m, 1H, *p*-Ar-CMe_2Ph), 7.04 (m, 2H, Ar-CMe_2Ph), 6.97 (br s, 2H, *m*-Ar-Mes), 6.66 (br s, 2H, *m*-Ar-Mes), 2.23 (s, 6H, Me-Mes), 2.08 (s, 6H, Me-Mes), 1.92 (s, 6H, Me-Mes), 1.80 (s, 3H, Me), 1.79 (s, 3H, Me), 1.01 (s, 3H, C<u>Me2</u>Ph). **^{13}C NMR** (101 MHz, $CDCl_3$) δ = 328.0 (Mo=CH), 182.2 (NCN-NHC), 161.6 (q, $^1J_{CB}$ = 49.9 Hz, *ipso*-Ar-B(Ar^F)4), 149.4 (*ipso*-Ar-Imido), 143.0 (*ipso*-Ar-CMe_2Ph), 141.5 (*ipso*-Ar-Mes), 135.3 (*o*-Ar-Mes), 134.9 (br m, *o*-Ar-B(Ar^F)4), 134.5 (*o*-Ar-Mes), 134.2 (*p*-Ar-Mes), 132.3 (Ar-Imido), 131.0 (Ar-Imido), 130.6 (*m*-Ar-Mes), 130.2 (*o*-Ar-Imido), 129.8 (*m*-Ar-Mes), 129.5 (Ar-Imido), 129.1 (qq, $^2J_{CF}$ = 31.5 Hz, $^3J_{CB}$ = 2.9 Hz, *m*-Ar-B(Ar^F)4), 128.8 (Ar-Imido), 127.3 (Ar-CMe_2Ph), 126.2 (CH=CH-NHC), 125.7 (Ar-CMe_2Ph), 124.7 (q, $^1J_{CF}$ = 272.5 Hz, CF_3-B(Ar^F)4), 119.3 (q, $^1J_{CF}$ = 319.3 Hz, OTf), 117.6 (sept, $^3J_{CF}$ = 3.6 Hz, *p*-Ar-B(Ar^F)4), 58.0 (<u>C</u>Me2Ph), 29.1 (C<u>Me2</u>Ph), 28.7 (C<u>Me2</u>Ph), 20.9 (*p*-Me-Mes), 18.1 (*o*-Me-Mes), 18.0 (*o*-Me-Mes), 2.3 (MeCN). **^{19}F NMR** (376 MHz, $CDCl_3$) δ = -59.24 (s, 3F, CF_3-Imido), -62.41 (s, 24F, B(Ar^F)4), -75.62 (s, 3F, OTf). **Elemental analysis** (%) calcd. for $C_{73}H_{55}BF_{30}MoN_4O_3S$: C, 50.25; H, 3.18; N, 3.21. Found: C, 50.22; H, 3.14; N, 3.27.

Mo(*N*-2,6-F_2-C_6H_3)$_2$(Cl)$_2$(DME), **4d-p1**
Ammonium dimolybdate (1.00 g, 2.94 mmol, 1 equiv.) was suspended in 30 mL acetonitrile. Triethylamine (2.38 g, 23.52 mmol, 8 equiv.), chlorotrimethysilane (5.45 g, 50.17 mmol, 17 equiv.) and 2,6-difluoroaniline (1.52 g, 11.77 mmol, 4 equiv.) were added sequentially under stirring. The reaction mixture was stirred at room temperature for 36 h to afford a dark red solution with some precipitate. The solvent was removed *in vacuo* and the remaining solid material was stirred with a minimum amount of 1,2-dimethoxyethane (DME) for 12 hours. Subsequently the solvent was removed *in vacuo* and co-evaporated with *n*-pentane several times. The resulting black-red solid was extracted with a Soxhlet apparatus using DME as a solvent. The DME was removed *in vacuo* and a minimum amount of dichloromethane was added to the obtained black-red solid and stirred for one hour. From this mixture a crystalline red solid was filtered off. The product was washed with cold dichloromethane and recrystallized from a mixture of DCM/diethyl ether to get a crystalline bright orange solid (0.72 g, 47% yield). **^{1}H NMR** (400 MHz, CD_2Cl_2) δ = 7.11 (m, 2H, *p*-Ar-Imido), 6.92 (m, 4H, *m*-Ar-Imido), 4.01 (s, 4H, CH_2-DME), 3.99 (s, 6H, CH_3-DME). **^{13}C NMR** (101 MHz, CD_2Cl_2) δ = 158.3 (d, $^1J_{CF}$ = 256.6 Hz, *o*-Ar-Imido), 135.5 (m, *ipso*-Ar-Imido), 129.2 (m, *p*-Ar-Imido), 112.0 (m, *m*-Ar-Imido), 72.0 (CH_2-DME), 64.7 (CH_3-DME). **^{19}F NMR** (376 MHz, CD_2Cl_2) δ = -117.37 (m, 4F, Imido). **Elemental analysis** (%) calcd. for $C_{16}H_{16}F_4MoN_2O_2$: C, 37.60; H, 3.16; N, 5.48. Found: C, 37.48; H, 3.45; N, 5.59.

Mo(*N*-2,6-F_2-C_6H_3)$_2$(CH_2CMe_2Ph)$_2$, **4d-p2**

$Mo(N\text{-}2,6\text{-}F_2\text{-}C_6H_3)_2Cl_2(DME)$ **4d-p1** (0.131 g, 0.25 mmol, 1 equiv.) was dissolved in 15 mL THF and cooled to -30 °C. Neophylmagnesium chloride (1.21 M in diethyl ether, 0.4 mL, 0.50 mmol, 2 equiv.) was added dropwise to the stirring solution. The reaction mixture was stirred overnight at room temperature and filtered through celite. The solvent was removed *in vacuo* and the red-orange solid was recrystallized from pentane (0.14 g, 92% yield). **^{1}H NMR** (400 MHz, CD_2Cl_2) δ = 7.38 (m, 4H, *o*-Ar-CMe_2Ph) 7.22 (m, 4H, *m*-Ar-CMe_2Ph), 7.08 (m, 2H, *p*-Ar-CMe_2Ph), 6.97 (m, 2H, *p*-Ar-Imido), 6.82 (m, 4H, *m*-Ar-Imido), 1.97 (s, 4H, CH_2), 1.40 (s, 12H, Me). **^{13}C NMR** (101 MHz, CD_2Cl_2) δ = 156.6 (d, $^1J_{CF}$ = 252.1 Hz, *o*-Ar-Imido), 151.2 (*ipso*-Ar-CMe_2Ph), 135.2 (m, *ipso*-Ar-Imido), 128.7 (Ar-CMe_2Ph), 126.3 (Ar-CMe_2Ph), 126.3 (Ar-CMe_2Ph), 125.1 (m, *p*-Ar-Imido), 111.4 (m, *m*-Ar-Imido), 83.4 (CH_2), 41.2 ($\underline{C}Me_2Ph$), 32.2 ($C\underline{Me_2}Ph$). **^{19}F NMR** (376 MHz, CD_2Cl_2) δ = -119.52 (m, 4F, Imido). **Elemental analysis** (%) calcd. for $C_{32}H_{32}F_4MoN_2$: C, 62.34; H, 5.23; N, 4.54. Found: C, 62.37; H, 5.39; N, 4.44.

$Mo(N\text{-}2,6\text{-}F_2\text{-}C_6H_3)(CHCMe_2Ph)(OTf)_2DME$, **4d-p3**

$Mo(N\text{-}2,6\text{-}F_2C_6H_3)_2(CH_2CMe_2Ph)_2$ **4d-p2** (0.140 g, 0.23 mmol, 1 equiv.) was dissolved in 20 mL 1,2-dimethoxyethane (DME) and cooled to -30 °C. The chilled triflic acid (0.10 g, 0.69 mmol) and 1 mL of 1,2-dimethoxyethane was mixed and cooled to -30 °C. After one hour, the cooled triflic acid solution was added dropwise to the solution of educt. The reaction mixture was stirred overnight and DME was evaporated *in vacuo*. Benzene was added to the residue and the suspension was stirred for 15 minutes. Subsequently the precipitate was removed by filtration through celite. The benzene was removed *in vacuo* and diethyl ether was added. The yellow precipitate was filtered and washed with cold diethyl ether. The product was recrystallized from a mixture of CH_2Cl_2/Et_2O/pentane to obtain a yellow crystalline solid (0.11 g, 64% yield). The product consists of two isomers in a 1:0.5 ratio, as can be seen in NMR spectra. We assign the two isomers to complexes with *cis* and *trans* coordinated triflates. **^{1}H NMR** (400 MHz, CD_2Cl_2) δ = 14.74 (Mo=CH), 13.89 (Mo=CH), 7.47, 7.30, 7.19, 7.08, 6.98, 6.87, 6.82, 4.19, 4.11, 4.00, 3.88, 3.67, 3.60, 3.56, 1.83, 1.62, 1.50. **^{13}C NMR** (101 MHz, CD_2Cl_2) δ = 335.5 (d, *J* = 32.8 Hz, Mo=CH), 327.1 (d, *J* = 11.7 Hz, Mo=CH), 161.3 (dd, *J* = 260.9, 2.4 Hz, *o*-Ar-Imido), 160.9 (dd, *J* = 257.9, 2.6 Hz, *o*-Ar-Imido), 148.5 (*ipso*-Ar-CMe_2Ph), 147.0 (*ipso*-Ar-CMe_2Ph), 133.3 (t, *J* = 16.6 Hz, *ipso*-Ar-Imido), 131.3 (t, *J* = 9.6 Hz, *ipso*-Ar-Imido), 131.0 (t, *J* = 9.7 Hz, *ipso*-Ar-Imido), 129.0 (Ar-CMe_2Ph), 128.5 (Ar-CMe_2Ph), 127.7 (Ar-CMe_2Ph), 127.31 (Ar-CMe_2Ph), 127.30 (Ar-CMe_2Ph), 126.9 (Ar-CMe_2Ph), 120.1 (q, $^1J_{CF}$ = 317.6, OTf), 119.8 (qd, $^1J_{CF}$ = 317.7, 5.6 Hz, OTf), 112.7 (m, *m*-Ar-Imido), 112.4 (m, *m*-Ar-Imido), 79.0 (DME), 77.1 (DME), 75.0 (DME), 70.9 (DME), 65.7 (DME), 63.2 (DME), 61.7 (DME), 58.8 ($\underline{C}Me_2Ph$), 58.8 ($\underline{C}Me_2Ph$), 29.9 ($C\underline{Me_2}Ph$), 29.6 ($C\underline{Me_2}Ph$), 29.2 ($C\underline{Me_2}Ph$). **^{19}F NMR** (376 MHz, CD_2Cl_2) δ = -77.31 (OTf), -77.35 (OTf), -78.24 (OTf), -111.85 (F-Imido), -114.36 (F-Imido). **Elemental analysis** (%) calcd. for $C_{22}H_{25}F_8MoNO_8S_2$: C, 35.54; H, 3.39; N, 1.88. Found: C, 35.50; H, 3.56; N, 1.90.

$Mo(N\text{-}2,6\text{-}F_2\text{-}C_6H_3)(CHCMe_2Ph)(IMes)(OTf)_2$, **4d-p4**

$Mo(N\text{-}2,6\text{-}F_2\text{-}C_6H_3)(CHCMe_2Ph)(OTf)_2(DME)$ **4d-p3** (0.080 g, 0.11 mmol, 1 equiv.) was dissolved in 5 mL benzene and a solution of 1,3-bis(2,4,6-trimethylphenyl)imidazol-2-ylidene (0.033 g, 0.11 mmol, 1 equiv.) in 1 mL benzene was added dropwise. The reaction mixture was stirred for 3 h at room temperature. The solvent was removed *in vacuo*. The yellow solid was recrystallized from a mixture of dichloromethane and pentane to obtain a yellow crystalline solid (0.070 g, 67% yield). **^{1}H NMR** (400 MHz, CD_2Cl_2) δ = 13.32 (s, $^1J_{CH}$ = 123.4 Hz, 1H, Mo=CH), 7.26 (m, 3H, Ar), 7.22 (m, 1H, Ar), 7.21 (s, 2H, CH=CH-NHC), 7.09 (m, 2H, Ar), 6.83 (m, 2H, Ar), 6.73 (br s, 2H, *m*-Ar-Mes), 6.63 (br s, 2H, *m*-Ar-Mes), 2.15 (s, 6H, Me-Mes), 2.03 (s, 6H, Me-Mes), 2.00 (s, 6H, Me-Mes), 1.71 (s, 3H, $C\underline{Me_2}Ph$), 1.69 (s, 3H, $C\underline{Me_2}Ph$). **^{13}C NMR** (101 MHz, CD_2Cl_2) δ = 320.4 (Mo=CH), 184.0 (NCN-NHC), 160.2 (d, $^1J_{CF}$ = 258.8 Hz, *o*-Ar-Imido), 148.1 (*ipso*-Ar-CMe_2Ph), 141.0 (*ipso*-Ar-Mes), 136.0 (Ar-Mes), 135.3 (Ar-Mes), 135.1 (Ar-Mes), 134.0 (m, Ar-Imido), 131.2 (m, Ar-Imido), 130.4 (*m*-Ar-Mes), 129.7 (*m*-Ar-Mes), 128.8 (Ar-CMe_2Ph), 127.0 (Ar-CMe_2Ph), 125.98 (CH=CH-NHC), 125.96 (Ar-CMe_2Ph), 119.9 5 (q, $^1J_{CF}$ = 318.8 Hz, OTf), 111.8 (dd, J_{CF} = 19.9, 3.1 Hz, *m*-Ar-Imido), 56.5 ($\underline{C}Me_2Ph$), 35.1 ($C\underline{Me_2}Ph$), 27.9 ($C\underline{Me_2}Ph$), 21.4 (*p*-Me-Mes), 18.49 (*o*-Me-Mes), 18.44 (*o*-Me-Mes). **^{19}F NMR** (376 MHz, CD_2Cl_2) δ = -75.77 (br s, 3F, OTf), -77.02 (br s, 3F, OTf), -109.53 (br s, 2F, Imido). **Elemental analysis** (%) calcd. for $C_{39}H_{39}F_8MoN_3O_6S_2$: C, 48.91; H, 4.10; N, 4.39. Found: C, 48.65; H, 4.21; N, 4.41.

$[Mo(N\text{-}2,6\text{-}F_2\text{-}C_6H_3)(CHCMe_2Ph)(IMes)(OTf)(MeCN)][B(Ar^F)_4]$, **4d**

$Mo(N\text{-}2,6\text{-}F_2\text{-}C_6H_3)(CHCMe_2Ph)(OTf)_2(IMes)$ **4d-p4** (0.050 g, 0.052 mmol. 1 equiv.) was dissolved in 4 mL dichloromethane and cooled to -30 °C for about 30 min. Then $Ag(CH_3CN)_2B(Ar^F)_4$ (0.057 mg, 0.052 mmol, 1 equiv.) was added in small portions as a solid and the reaction mixture was stirred for 2 h at room temperature. The reaction mixture was filtered through a pad of celite and CH_2Cl_2 was removed *in vacuo*. The residue was co-evaporated with pentane to yield a yellow solid. The product was recrystallized from a mixture of CH_2Cl_2/Et_2O/pentane to obtain a crystalline yellow solid (0.080 g, 89% yield). **^{1}H NMR** (400 MHz, CD_2Cl_2) δ = 13.26 (s, $^1J_{CH}$ = 124.0 Hz, 1H, Mo=CH), 7.73 (br m, 8H, *o*-Ar-B(Ar^F)$_4$), 7.56 (br s, 4H, *p*-Ar-B(Ar^F)$_4$), 7.33 (s, 2H, CH=CH-NHC), 7.30 (m, 1H, Ar), 7.22 (m, 2H, Ar), 7.15 (m, 1H, Ar), 7.07 (m, 2H, Ar), 6.95 (s, 2H, *m*-Ar-Mes), 6.88 (br m, 2H, Ar), 7.00 – 6.35 (br s, 2H, *m*-Ar-Mes), 2.22 (s, 6H, Me-Mes), 2.05 (br s, 12H, Me-Mes), 1.87 (s, 3H, Me), 1.78 (s, 3H, Me), 1.10 (s, 3H, CMe_2Ph). **^{13}C NMR** (101 MHz, CD_2Cl_2) δ = 328.6 (Mo=CH), 182.8 (NCN-NHC), 162.1 (q, $^1J_{CB}$ = 50.0 Hz, *ipso*-Ar-B(Ar^F)$_4$), 144.2 (*ipso*-Ar-CMe_2Ph), 141.5 (*ipso*-Ar-Mes), 135.9 (Ar-Mes), 135.2 (br m, *o*-Ar-B(Ar^F)$_4$), 134.8 (Ar-Mes), 134.4 (Ar-Mes), 132.7 (m, Ar-Imido), 130.6 (*m*-Ar-Mes), 130.1 (Ar-Imido), 129.8 (br, Ar-Imido), 129.2 (qq, $^2J_{CF}$ = 31.2 Hz, $^3J_{CB}$ = 2.9 Hz, *m*-Ar-B(Ar^F)$_4$), 129.0 (Ar-CMe_2Ph), 127.5 (Ar-CMe_2Ph), 126.4 (CH=CH-NHC), 125.8 (Ar-CMe_2Ph), 125.0 (q, $^1J_{CF}$ = 272.4 Hz, CF_3-B(Ar^F)$_4$), 119.5 (q, $^1J_{CF}$ = 318.5 Hz, OTf), 117.9 (sept, $^3J_{CF}$ = 3.6 Hz, *p*-Ar-B(Ar^F)$_4$), 112.3 (dd, *J* = 19.5, 3.1 Hz, *m*-Ar-Imido), 56.5 ($\underline{C}Me_2Ph$), 28.5 ($C\underline{Me_2}Ph$), 27.8 ($C\underline{Me_2}Ph$), 21.3 (*p*-Me-Mes), 18.1 (*o*-Me-Mes), 3.0 (MeCN). **^{19}F NMR** (376 MHz, CD_2Cl_2) δ = -62.76 (s, 24F, B(Ar^F)$_4$), -76.28 (s, 3F, OTf), -111.14 (br s, 2F, Imido). **Elemental analysis** (%) calcd. for $C_{72}H_{54}BF_{29}MoN_4O_3S$: C, 50.48; H, 3.18; N, 3.27. Found: C, 50.52; H, 3.23; N, 3.32.

$[Mo(N\text{-}2,6\text{-}Me_2\text{-}C_6H_3)(CHCMe_2Ph)(IMes)(OTf)][B(Ar^F)_4]$, **5a**

Mo(*N*-2,6-Me_2-C_6H_3)($CHCMe_2Ph$)(IMes)$(OTf)_2$[15] (70.7 mg, 0.0744 mmol, 1 equiv) was dissolved in 5 mL CH_2Cl_2. At room temperature solid NaB$(Ar^F)_4$ (66 mg, 0.0744 mmol, 1 equiv) was added. The mixture was stirred for 30 min. Subsequently all solids were filtered off over celite and the filtrate was reduced to dryness. The oily product was dissolved in 1 mL CH_2Cl_2 and filtered once more. The CH_2Cl_2 was removed *in vacuo* and the oily product was triturated with pentane until a yellow solid formed. Yield: 103 mg, 83%.^{1}**H NMR** (400 MHz, $CDCl_3$) δ = 13.03 (s, 1H, $^1J_{CH}$ = 120.7 Hz, Mo=CH), 7.70 (br m, 8H, *o*-Ar-B$(Ar^F)_4$), 7.51 (br s, 4H, *p*-Ar-B$(Ar^F)_4$), 7.34 (s, 2H, CH=CH-NHC), 7.07 (m, 3H, Ar), 7.00 (m, 2H, Ar), 6.95 (m, 2H, Ar), 6.87 (br s, 2H, Ar-Mes), 6.78 (m, 3H, Ar), 2.31 (s, 6H, Me-Mes), 2.08 (br s, 3H, Me-Imido), 2.02 (s, 6H, Me-Mes), 1.85 (br s, 9H, Me-Mes overlapping with Me-Imido), 1.10 (s, 3H, C$\underline{Me_2}$Ph), 0.98 (s, 3H, C$\underline{Me_2}$Ph). ^{13}**C NMR** (101 MHz, $CDCl_3$) δ = 321.5 (Mo=CH), 179.9 (NCN-NHC), 161.9 (q, $^1J_{CB}$ = 50.0 Hz, *ipso*-Ar-B$(Ar^F)_4$), 154.3 (*ipso*-Ar-Imido), 145.5 (*ipso*-Ar-CMe_2Ph), 142.4 (*ipso*-Ar-Mes), 135.4 (Ar-Mes), 135.3 (Ar-Mes), 135.0 (br m, *o*-Ar-B$(Ar^F)_4$), 132.7 (br, Ar-Imido), 131.1 (Ar-Imido), 130.8 (*m*-Ar-Mes), 130.1 (*m*-Ar-Mes), 129.1 (qq, $^2J_{CF}$ = 31.1 Hz, $^3J_{CB}$ = 2.8 Hz, *m*-Ar-B$(Ar^F)_4$), 128.3 (Ar-CMe_2Ph), 128.2 (br, Ar-Imido), 127.2 (Ar-CMe_2Ph), 126.3 (CH=CH-NHC), 126.2 (Ar-CMe_2Ph), 124.5 (q, $^1J_{CF}$ = 272.4 Hz, CF_3-B$(Ar^F)_4$), 119.5 (q, $^1J_{CF}$ = 320.1 Hz, OTf), 117.6 (sept, $^3J_{CF}$ = 3.6 Hz, *p*-Ar-B$(Ar^F)_4$), 58.1 ($\underline{C}Me_2Ph$), 30.5 (C$\underline{Me_2}$Ph), 26.2 (C$\underline{Me_2}$Ph), 21.1 (*p*-Me-Mes), 19.5 (Me-Imido), 17.9 (*o*-Me-Mes), 17.4 (*o*-Me-Mes). ^{19}**F NMR** (376 MHz, $CDCl_3$) δ = -62.42 (s, 24F, B$(Ar^F)_4$), -73.22 (s, 3F, OTf). **Elemental analysis** (%) calcd. for $C_{65}H_{49}BCl_2F_{27}MoN_3O_3S$: C, 51.97; H, 3.45; N, 2.53. Found: C, 51.94; H, 3.68; N, 2.46.

[Mo(*N*-2,6-Me_2-C_6H_3)($CHCMe_2Ph$)(IMes)($OC(CF_3)_3$)][B$(Ar^F)_4$], **5b**

[Mo(*N*-2,6-Me_2-C_6H_3)($CHCMe_2Ph$)(OTf)(IMes)][B$(Ar^F)_4$] **5a** (377.4 mg, 0.2 mmol, 1 equiv.) was dissolved in CH_2Cl_2 (7 mL). Li($OC(CF_3)_3$) (54.9 mg, 0.2 mmol, 1 equiv.) was dissolved in a mixture of CH_2Cl_2 (1 mL) and acetonitrile (1 mL) and both mixtures were cooled to -35 °C. Then, the lithium alkoxide solution was slowly added to the metal complex solution. The mixture was stirred at room temperature for 3 hours, upon which the solution turned cloudy. All volatiles were removed and the residue was triturated with CH_2Cl_2 (~12 mL), resulting in a yellow suspension. LiOTf was removed by filtration over Celite. The filtrate was evaporated to dryness. For crystallization, the residue was dissolved in CH_2Cl_2 (2 mL) and diethyl ether (4 mL). Then, pentane (~10 mL) was added until the solution started getting cloudy upon addition of more pentane. Crystals of **5b** were obtained from this solution at -35 °C after 12 hours. Yield: 77% (305 mg). ^{1}**H NMR** (400 MHz, CD_2Cl_2) δ = 12.69 (s, 1H, Mo=CH), 7.72 (br m, 8H, *o*-Ar-B$(Ar^F)_4$), 7.56 (br s, 4H, *p*-Ar-B$(Ar^F)_4$), 7.47 (s, 2H, CH=CH-NHC), 7.12 (t, $^3J_{HH}$ = 7.6 Hz, 1H, *p*-Ar-Imido), 7.09 (s, 2H, *m*-Ar-Mes), 7.03 – 6.98 (m, 4H, Ar-$CMe_2\underline{Ph}$), 6.95 (d, $^3J_{HH}$ = 7.6 Hz, 2H, *m*-Ar-Imido), 6.85 (br m, 3H, overlapping signals of *p*-Ar-$CMe_2\underline{Ph}$ and *m*-Ar-Mes), 2.33 (s, 6H, Me-Mes), 2.07 (s, 6H, Me-Mes), 1.91 (s, 6H, Me-Mes), 1.91 (br s, 6H, Me-Imido), 1.24 (s, 3H, C$\underline{Me_2}$Ph), 0.95 (s, 3H, C$\underline{Me_2}$Ph). ^{13}**C NMR** (101 MHz, CD_2Cl_2) δ = 315.6 (Mo=CH), 180.10 (NCN-NHC), 162.3 (q, $^1J_{CB}$ = 50.1 Hz, *ipso*-Ar-B$(Ar^F)_4$), 156.1 (*ipso*-Ar-Imido), 146.5 (*ipso*-Ar-CMe_2Ph), 142.5 (*ipso*-Ar-Mes), 135.5 (Ar-Mes), 135.4 (br m, *o*-Ar-B$(Ar^F)_4$), 134.2 (Ar-Mes), 131.2 (Ar-Imido), 131.0 (Ar-Imido), 130.3 (*m*-Ar-Mes), 129.4 (q, $^2J_{CF}$ = 31.2 Hz, *m*-Ar-B$(Ar^F)_4$), 128.7 (Ar-Imido), 128.6 (Ar-CMe_2Ph), 127.5 (Ar-CMe_2Ph), 127.2 (Ar-CMe_2Ph), 126.3 (CH=CH-NHC), 125.2 (q, $^1J_{CF}$ = 272.5 Hz, CF_3-B$(Ar^F)_4$), 121.1 (q, $^1J_{CF}$ = 292.9 Hz, OC($\underline{CF_3}$)$_3$), 118.0 (br m, *p*-Ar-B$(Ar^F)_4$), 84.7 (m, $\underline{C}(CF_3)_3$), 57.4 ($\underline{C}Me_2Ph$), 31.3 (C$\underline{Me_2}$Ph), 27.9 (C$\underline{Me_2}$Ph), 21.3 (*p*-Me-Mes), 19.7 (Me-Imido), 18.1 (*o*-Me-Mes). ^{19}**F NMR** (367 MHz, CD_2Cl_2) δ = -62.9 (s, 24F, B$(Ar^F)_4$), -73.3 (s, 9F, $OC(CF_3)_3$). **Elemental analysis** (%) calcd. for $C_{75}H_{57}BF_{33}MoN_3O$: C, 51.48; H, 3.28; N, 2.40. Found: C, 51.48; H, 3.57; N, 2.71.

[Mo(*N*-2,6-Me_2-C_6H_3)($CHCH_2OCH_2CH_2OH$)(IMes)(OTf)][B$(Ar^F)_4$], **6**

[Mo(*N*-2,6-Me_2-C_6H_3)($CHCMe_2Ph$)(IMes)(OTf)][B$(Ar^F)_4$] **5a** (33 mg, 0.0198 mmol, 1 equiv) was dissolved in 2 mL CH_2Cl_2. At room temperature 2-allyloxyethanol (3 mg, 0.036 mL, 0.0297 mmol, 1.5 equiv) was added via syringe. The mixture was stirred for 5 min. All volatiles were removed *in vacuo* to obtain a yellow oily solid. This solid was washed with pentane (3x 2 mL) and then recrystallized from 1 mL dipropyl ether. A yellow crystalline solid precipitated in the course of a few days at -35 °C (25 mg, 69%) which analyzed as the propyl ether adduct. NMR Data is given for a sample which was not recrystallized. ^{1}**H NMR** (400 MHz, $CDCl_3$) δ = 13.38 (dd, $^3J_{HH}$ = 4.2, 3.1 Hz, $^1J_{CH}$ = 167 Hz, 1H, Mo=CH), 7.95 (dd, *J* = 8.1, 3.8 Hz, 1H), 7.70 (br m, 8H, *o*-Ar-B$(Ar^F)_4$), 7.51 (br s, 4H, *p*-Ar-B$(Ar^F)_4$), 7.16 (s, 2H, CH=CH-NHC), 7.16 (t, $^3J_{HH}$ = 7.6 Hz, 1H, *p*-Ar-Imido), 6.99 (br s, 2H, *m*-Ar-Mes) overlapping with (m, 2H, *m*-Ar-Imido), 6.67 (br s, 2H, *m*-Ar-Mes), 5.13 (dd, *J* = 20.5, 4.4 Hz, 1H), 4.30 – 4.10 (m, overlapping 3H), 3.82 (dd, *J* = 13.0, 3.2 Hz, 1H), 3.67 (m, 1H), 2.7 – 1.6 (br s, 6H, Me-Imido), 2.27 (s, 6H, Me-Mes), 1.98 (s, 6H, Me-Mes), 1.86 (s, 6H, Me-Mes), Imido-methyl groups are broad and overlap with signals between 2.70 and 1.6 ppm. ^{13}**C NMR** (101 MHz, $CDCl_3$) δ = 309.5 (Mo=CH), 180.4 (NCN-NHC), 161.7 (q, $^1J_{CB}$ = 49.8 Hz, *ipso*-Ar-B$(Ar^F)_4$), 155.2 (*ipso*-Ar-Imido), 141.5 (*ipso*-Ar-Mes), 140.3 (br, *o*-Ar-Imido), 135.7 (Ar-Mes), 135.0 (br m, *o*-Ar-B$(Ar^F)_4$), 131.3 (*p*-Ar-Imido), 130.0 (*m*-Ar-Mes), 129.9 (*m*-Ar-Mes), 129.0 (qq, $^2J_{CF}$ = 31.4 Hz, $^3J_{CB}$ = 2.8 Hz, *m*-Ar-B$(Ar^F)_4$), 128.11 (br, *m*-Ar-Imido), 125.97 (CH=CH-NHC), 124.7 (q, $^1J_{CF}$ = 272.5 Hz, CF_3-B$(Ar^F)_4$), 117.6 (sept, $^3J_{CF}$ = 3.6 Hz, *p*-Ar-B$(Ar^F)_4$), 82.3 (CH_2), 71.5 (CH_2), 63.6 (CH_2), 21.1 (*p*-Me-Mes), 19.5 (Me-Imido), 17.8 (*o*-Me-Mes), 17.5 (*o*-Me-Mes). ^{19}**F NMR** (376 MHz, $CDCl_3$) δ = -62.39 (s, 24F, B$(Ar^F)_4$), -76.70 (s, 3F, OTf). **Elemental analysis** (%) calcd. for $C_{65}H_{51}BF_{27}MoN_3O_5S{\cdot}2Pr_2O$: C, 51.35; H, 4.48; N, 2.30. Found: C, 51.21; H, 4.49; N, 2.45.

Mo(*N*-*t*Bu)($CHCMe_2Ph$)(IMes)(OTf)($C_6F_5CO_2$), **10**

Mo(*N*-*t*Bu)($CHCMe_2Ph$)(IMes)$(OTf)_2$[14] (111 mg, 0.123 mmol, 1 equiv) was dissolved in 5 mL CH_2Cl_2 and cooled to -35 °C. To the cold solution solid silver pentafluorobenzoate (40 mg, 0.123 mmol, 1 equiv) was added. The mixture was stirred for one hour in the dark. All solids were filtered off and the filtrate was reduced to dryness. The oily residue was redissolved in 1 mL CH_2Cl_2 and filtered once more. All volatiles were removed *in vacuo*. The residue was triturated with 5 mL pentane until a pale yellow solid precipitated. Yield: 82 mg, 69%. ^{1}**H NMR** (400 MHz, $CDCl_3$) δ = 13.59 (s, $^1J_{CH}$ = 123.11 Hz, 1H, Mo=CH), 7.18 (m, 2H, Ar-CMe_2Ph), 7.15 – 7.00 (m, 7H, overlapping ArH), 6.82 (br s, 2H, *m*-Ar-Mes), 2.35 (s, 6H, Me-Mes), 2.18 (br s, 6H, Me-Mes), 1.89 (br s, 6H, Me-Mes), 1.66 (s, 3H, C$\underline{Me_2}$Ph), 1.64 (s, 3H, C$\underline{Me_2}$Ph), 1.10 (s, 9H, *t*Bu). ^{13}**C NMR** (101 MHz, $CDCl_3$) δ = 317.2 (Mo=CH), 188.4 (NCN-NHC), 174.4 (CO_2-Carboxylate), 149.4 (*ipso*-Ar-CMe_2Ph), 140.0 (*ipso*-Ar-Mes), 130.0 (br, *m*-Ar-Mes), 129.5 (br, *m*-Ar-Mes), 128.2 (CH=CH-NHC),

126.09 (Ar-CMe$_2$Ph), 126.05 (*p*-Ar-CMe$_2$Ph), 125.0 (Ar-CMe$_2$Ph), 119.9 (q, $^1J_{CF}$ = 317.1 Hz, CF$_3$-OTf), 76.4 (*N*-<u>C</u>Me$_3$), 53.00 (<u>C</u>Me$_2$Ph), 33.0 (C<u>Me$_2$</u>Ph), 29.7 (*N*-C<u>Me$_3$</u>), 29.7 (C<u>Me$_2$</u>Ph), 21.2 (*p*-Me-Mes), 18.5 (*o*-Me-Mes), 18.3 (*o*-Me-Mes), Aromatic resonances of the carboxylate- and IMes ligand were broad and only partially observed. **^{19}F NMR** (376 MHz, CDCl$_3$) δ = -77.38 (s, 3F, OTf), -136.57 (m, 2F, C$_6$F$_5$), -149.92 (m, 1F, *p*-C$_6$F$_5$), -162.69 (m, 2F, C$_6$F$_5$). **Elemental analysis** (%) calcd. for $C_{43}H_{45}F_8MoN_3O_5S$: C, 53.58; H, 4.71; N, 4.36. Found: C, 53.52; H, 4.71; N, 4.48.

[Mo(*N*-*t*Bu)(CHCMe$_2$Ph)(IMes)(C$_6$F$_5$CO$_2$)][B(Ar^F)$_4$], **11**

Mo(*N*-*t*Bu)(CHCMe$_2$Ph)(IMes)(OTf)(C$_6$F$_5$CO$_2$) **10** (53 mg, 0.055 mmol, 1 equiv) was dissolved in 3 mL CH$_2$Cl$_2$. At room temperature solid NaB(Ar^F)$_4$ (48.7 mg, 0.055 mmol, 1 equiv) was added. The mixture was stirred for 30 min. Subsequently all solids were filtered off and the filtrate was reduced to dryness. The oily product was dissolved in 1 mL CH$_2$Cl$_2$ and filtered once more. The CH$_2$Cl$_2$ was removed *in vacuo* and the dark oily product (pure by NMR) was triturated with pentane until a pale yellow solid formed. Yield: 86 mg, 93%. **^{1}H NMR** (400 MHz, CDCl$_3$) δ = 13.06 (s, $^1J_{CH}$ = 118.55 Hz, 1H, Mo=CH), 7.71 (br m, 8H, *o*-Ar-B(Ar^F)$_4$), 7.51 (br s, 4H, *p*-Ar-B(Ar^F)$_4$), 7.25 (m, 5H, overlapping resonances of Ph, solvent and CH=CH-NHC), 7.12 (m, 2H, Ar-CMe$_2$Ph), 7.06 (br s, 2H, *m*-Ar-Mes), 6.86 (br s, 2H, *m*-Ar-Mes), 2.28 (s, 6H, Me-Mes), 2.11 (s, 6H, Me-Mes), 1.90 (s, 6H, Me-Mes), 1.45 (s, 3H; C<u>Me$_2$</u>Ph), 1.40 (s, 3H, C<u>Me$_2$</u>Ph), 0.87 (*N*-*t*Bu). **^{13}C NMR** (101 MHz, CDCl$_3$) δ = 315.4 (Mo=CH), 182.2 (NCN-NHC), 180.0 (CO$_2$-carboxylate), 162.1 (q, $^1J_{CB}$ = 49.8 Hz, *ipso*-Ar-B(Ar^F)$_4$), 148.5 (br m, carboxylate), 147.2 (*ipso*-Ar-CMe$_2$Ph), 146.9 (br m, carboxylate), 145.8 (br m, carboxylate), 144.3 (br m, carboxylate), 141.9 (*ipso*-Ar-Mes), 139.5 (br m, carboxylate), 136.9 (br m, carboxylate), 135.5 (Ar-Mes), 135.0 (br m, *o*-Ar-B(Ar^F)$_4$), 134.8 (Ar-Mes), 134.5 (Ar-Mes), 130.7 (*m*-Ar-Mes), 130.5 (*m*-Ar-Mes), 129.0 (qq, $^2J_{CF}$ = 31.5 Hz, $^3J_{CB}$ = 2.7 Hz, *m*-Ar-B(Ar^F)$_4$), 128.7 (Ar-CMe$_2$Ph), 127.2 (*p*-Ar-CMe$_2$Ph), 126.3 (CH=CH-NHC), 126.01 (Ar-CMe$_2$Ph), 124.7 (q, $^1J_{CF}$ = 272.5 Hz, CF$_3$-B(Ar^F)$_4$), 117.6 (sept, $^3J_{CF}$ = 3.8 Hz, *p*-Ar-B(Ar^F)$_4$), 105.9 (br m, carboxylate), 79.1 (*N*-<u>C</u>Me$_3$), 55.3 (<u>C</u>Me$_2$Ph), 32.5 (C<u>Me$_2$</u>Ph), 30.0 (*N*-C<u>Me$_3$</u>), 29.5 (C<u>Me$_2$</u>Ph), 21.1 (*p*-Me-Mes), 17.7 (*o*-Me-Mes). **^{19}F NMR** (376 MHz, CDCl$_3$) δ = -62.46 (s, 24F, B(Ar^F)$_4$), -134.69 (m, 2F, C$_6$F$_5$), -141.37 (m, 1F, *p*-C$_6$F$_5$), -158.72 (m, 2F, C$_6$F$_5$). **Elemental analysis** (%) calcd. for $C_{74}H_{57}BF_{29}MoN_3O_2$: C, 52.97; H, 3.42; N, 2.50. Found: C, 53.05; H, 3.56; N, 2.63.

[Mo(*N*-2,6-Me$_2$-C$_6$H$_3$)(CHCMe$_2$Ph)(IMes)(C$_6$F$_5$CO$_2$)][Al(OC(CF$_3$)$_3$)$_4$], **12**

Mo(*N*-2,6-Me$_2$-C$_6$H$_3$)(CHCMe$_2$Ph)(IMes)(OTf)$_2$[15] (53 mg, 0.0558 mmol, 1 equiv) was dissolved in 3 mL CH$_2$Cl$_2$ and cooled to -35 °C. To this solution solid LiAl(OC(CF$_3$)$_3$)$_4$ (54.3 mg, 0.0558 mmol, 1 equiv) was added and the solution was stirred for 15 min at room temperature. All solids were filtered off and solid Lithium pentafluorobenzoate (15.8 mg, 0.0725 mmol, 1.3 equiv) was added to the filtrate. The mixture was stirred for one hour. Subsequently the reaction mixture was filtered and reduced to dryness. The residue was triturated with pentane until a yellow solid precipitated. The product can be recrystallized from chloroform. Yield: 82 mg, 80%. **^{1}H NMR** (400 MHz, CD$_2$Cl$_2$) δ = 12.99 (s, $^1J_{CH}$ = 121.1 Hz, 1H, Mo=CH), 7.44 (s, 2H, CH=CH-NHC), 7.11 (t, $^3J_{HH}$ = 7.6 Hz, 1H, *p*-Ar-Imido), 7.04 (s, 2H, *m*-Ar-Mes), 7.03 (m, 4H, Ar-CMe$_2$Ph), 6.96 (d, $^3J_{HH}$ = 7.6 Hz, 2H, *m*-Ar-Imido), 6.90 (m, 1H, Ar-CMe$_2$Ph), 6.80 (br s, 2H, *m*-Ar-Mes), 2.29 (s, 6H, Me-Mes), 2.25 (br s, 3H, Me-Imido), 2.07 (s, 6H, Me-Mes), 1.98 (s, 6H, Me-Mes), 1.95 (br s, 3H, Me-Imido), 1.39 (s, 3H, C<u>Me$_2$</u>Ph), 1.04 (s, 3H, C<u>Me$_2$</u>Ph). **^{13}C NMR** (101 MHz, CD$_2$Cl$_2$) δ = 312.1 (Mo=CH), 182.7 (N<u>C</u>N-NHC), 179.2 (CO$_2$-carboxylate), 154.4 (*ipso*-Ar-Imido), 148.9 (br m, Carboxylate), 147.2 (*ipso*-Ar-CMe$_2$Ph), 146.4 (br m, carboxylate), 144.6 (br m, carboxylate), 142.3 (*ipso*-Ar-Mes), 140.0 (br m, carboxylate), 137.4 (br m, carboxylate),136.1 (Ar-Mes), 135.6 (Ar-Mes), 133.9 (*o*-Ar-Imido), 130.6 (*m*-Ar-Mes), 130.43 (*p*-Ar-Imido), 130.39 (*m*-Ar-Mes), 128.7 (CH=CH-NHC), 128.4 (*m*-Ar-Imido), 127.2 (*p*-Ar-CMe$_2$Ph), 126.4 (Ar-CMe$_2$Ph), 126.3 (Ar-CMe$_2$Ph), 121.8 (q, $^1J_{CF}$ = 292.9 Hz, OC(<u>C</u>F$_3$)$_3$), 105.9 (br m, carboxylate), 79.5 (br, O<u>C</u>(CF$_3$)$_3$), 57.0 (<u>C</u>Me$_2$Ph), 29.1 (C<u>Me$_2$</u>Ph), 29.0 (C<u>Me$_2$</u>Ph), 21.3 (*p*-Me-Mes), 19.8 (Me-Imido), 18.04 (*o*-Me-Mes), 17.95 (*o*-Me-Mes). **^{19}F NMR** (376 MHz, CD$_2$Cl$_2$) δ = -75.75 (s, 36F, CF$_3$), -135.61 (m, 2F, C$_6$F$_5$), -142.79 (m, 1F, *p*-C$_6$F$_5$), -159.83 (m, 2F, C$_6$F$_5$). **Elemental analysis** (%) calcd. for $C_{62}H_{46}AlF_{41}MoN_3O_6$: C, 40.67; H, 2.53; N, 2.30. Found: C, 40.69; H, 2.48; N, 2.39.

[Mo(*N*-3,5-Me$_2$-C$_6$H$_3$)(CHCMe$_2$Ph)(IMes)(C$_6$F$_5$CO$_2$)][Al(OC(CF$_3$)$_3$)$_4$], **13**

Mo(*N*-3,5-Me$_2$-C$_6$H$_3$)(CHCMe$_2$Ph)(IMes)(OTf)$_2$[16] (120 mg, 0.126 mmol, 1 equiv) was dissolved in 6 mL CH$_2$Cl$_2$ and cooled to -35 °C. To this solution solid LiAl(OC(CF$_3$)$_3$)$_4$ (123 mg, 0.126 mmol, 1 equiv) was added and the solution was stirred for 15 min at room temperature. All solids were filtered off and solid lithium pentafluorobenzoate (35.8 mg, 0.164 mmol, 1.3 equiv) was added to the filtrate. The mixture was stirred for one hour. Subsequently the reaction mixture was filtered and reduced to dryness. The residue was triturated with pentane until a yellow solid precipitated. The product can be recrystallized from chloroform. Yield: 192 mg, 83%. **^{1}H NMR** (400 MHz, CD$_2$Cl$_2$) δ = 13.01 (s, $^1J_{CH}$ = 122.3 Hz, 1H, Mo=CH), 7.40 (s, 2H, CH=CH-NHC), 7.20 (m, 3H, Ar-CMe$_2$Ph), 7.10 (m, 2H, Ar-CMe$_2$Ph), 7.01 (br s, 1H, *p*-Ar-Imido), 6.87 (br s, 2H, *m*-Ar-Mes), 6.78 (br s, 2H, *m*-Ar-Mes), 6.49 (br s, 2H, *o*-Ar-Imido), 2.27 (s, 6H, Me), 2.20 (s, 6H, Me), 2.08 (s, 6H, Me), 1.99 (s, 6H, Me), 1.58 (s, 3H, C<u>Me$_2$</u>Ph), 1.37 (s, 3H, C<u>Me$_2$</u>Ph). **^{13}C NMR** (101 MHz, CD$_2$Cl$_2$) δ = 313.5 (Mo=CH), 183.6 (NCN-NHC), 180.5 (CO$_2$-carboxylate), 154.3 (*ipso*-Ar-Imido), 149.1 (br m, carboxylate), 146.7 (*ipso*-Ar-CMe$_2$Ph), 146.5 (br m, carboxylate), 141.8 (*ipso*-Ar-Mes), 139.9 (br m, carboxylate), 138.9 (Ar-Imido), 137.5 (br m, carboxylate), 135.9 (Ar-Mes), 134.7 (Ar-Mes), 134.6 (Ar-Mes), 133.2 (Ar-Imido), 130.5 (*m*-Ar-Mes), 130.5 (*m*-Ar-Mes), 129.1 (Ar-Imido), 127.5 (Ar-CMe$_2$Ph), 126.7 (Ar-CMe$_2$Ph), 126.2 (CH=CH-NHC), 126.1 (Ar-CMe$_2$Ph), 121.8 (q, J_{CF} = 291.1 Hz, CF$_3$), 56.7 (<u>C</u>Me$_2$Ph), 33.0 (C<u>Me$_2$</u>Ph), 28.9 (C<u>Me$_2$</u>Ph), 21.3 (*p*-Me-Mes), 21.3 (Me-Imido), 18.1 (*o*-Me-Mes), 18.0 (*o*-Me-Mes). **^{19}F NMR** (376 MHz, CD$_2$Cl$_2$) δ = -75.75 (s, 36F, CF$_3$), -134.88 (m, 2F, C$_6$F$_5$), -142.29 (m, 1F, *p*-C$_6$F$_5$), -159.79 (m, 2F, C$_6$F$_5$). **Elemental analysis** (%) calcd. for $C_{62}H_{45}AlF_{41}MoN_3O_6$: C, 40.69; H, 2.48; N, 2.30. Found: C, 40.41; H, 2.50; N, 2.39.

[Mo(*N*-2,6-Cl$_2$-C$_6$H$_3$)(CHCMe$_3$)(IMes)(C$_6$F$_5$CO$_2$)][B(Ar^F)$_4$], **14**

A solution of [Mo(*N*-2,6-Cl$_2$-C$_6$H$_3$)(CHCMe$_3$)(IMes)(OTf)][B(Ar^F)$_4$] **5a** (120 mg, 0.073 mmol, 1 equiv.) in 2 mL of CH$_2$Cl$_2$ was added to a suspension of LiO$_2$CC$_6$F$_5$ (17.5 mg, 0.08 mmol, 1.1 equiv.) in 1 mL of CH$_2$Cl$_2$ and the resulting suspension was stirred at room temperature for three hours. The suspension was filterted through a pad of celite and the solvent was removed *in vacuo*. The residue

was triturated with pentane to yield a yellow solid. The pentane was decanted and the solid was dried *in vacuo*. The compound was recrystallized from a mixture of CH_2Cl_2, diethylether and pentane to yield the product as yellow crystals. Yield: 71 mg (57 %). **^{1}H NMR** (400 MHz, $CDCl_3$) δ = 12.75 (s, $^1J_{CH}$ = 118.9 Hz, 1H, Mo=CH), 7.70 (br m, 8H, *o*-Ar-B(Ar^F)$_4$), 7.50 (br m, 4H, *p*-Ar-B(Ar^F)$_4$), 7.34 (s, 2H, CH=CH-NHC), 7.23 (m, 2H, *m*-Ar-Imido), 7.15 (m, 2H, *m*-Ar-Imido), 6.91 (br s, 2H, *m*-Ar-Mes), 6.81 (br s, 2H, *m*-Ar-Mes), 2.17 (s, 6H, Me-Mes), 2.08 (s, 6H, Me-Mes), 2.00 (s, 6H, Me-Mes), 1.14 (s, 9H, *t*Bu). **^{13}C NMR** (101 MHz, $CDCl_3$) δ = 321.2 (Mo=CH), 183.7 (NCN-NHC), 180.0 (CO_2), 161.9 (q, $^1J_{CB}$ = 49.8 Hz, *ipso*-Ar-B(Ar^F)$_4$), 148.8 (*ipso*-Ar-Imido), 148.7 (br m, carboxylate), 146.0 (br m, carboxylate), 141.5 (*ipso*-Ar-Mes), 139.5 (br m, carboxylate), 136.9 (br m, carboxylate), 135.7 (Ar-Mes), 134.9 (br m, *o*-Ar-B(Ar^F)$_4$), 134.4 (Ar-Mes), 134.2 (Ar-Mes), 130.6 (Ar-Imido), 130.4 (*m*-Ar-Mes), 129.9 (*m*-Ar-Mes), 129.0 (qq, $^2J_{CF}$ = 31.5 Hz, $^3J_{CB}$ = 2.7 Hz, *m*-Ar-B(Ar^F)$_4$), 128.3 (Ar-Imido), 125.6 (CH=CH-NHC), 124.7 (q, $^1J_{CF}$ = 272.5 Hz, CF_3-B(Ar^F)$_4$), 117.6 (sept, $^3J_{CF}$ = 3.8 Hz, *p*-Ar-B(Ar^F)$_4$), 105.1 (br m, carboxylate), 50.3 (<u>C</u>Me$_3$), 30.6 (C<u>Me</u>$_3$), 21.2 (*p*-Me-Mes), 18.1 (*o*-Me-Mes), 17.9 (*o*-Me-Mes). **^{19}F NMR** (376 MHz, $CDCl_3$) δ = -62.45 (s, 24F, B(Ar^F)$_4$), -134.52 (m, 2F, C_6F_5), -140.55 (m, 1F, *p*-C_6F_5), -158.35 (m, 2F, C_6F_5). **Elemental analysis** (%) calcd. for $C_{71}H_{49}BCl_2F_{29}MoN_3O_2$: C, 50.02; H, 2.90; N, 2.46. Found: C, 50.08; H, 2.97; N, 2.48.

[Mo(*N*-2,6-Cl_2-C_6H_3)($CHCMe_3$)(IMes)(2,6-(CF_3)-$C_6H_3CO_2$)][Al(OC(CF_3)$_3$)$_4$], **15a**

Mo(*N*-2,6-Cl_2-C_6H_3)($CHCMe_3$)(IMes)(OTf)$_2$[15] (49 mg, 0.0525 mmol, 1 equiv) was dissolved in 3 mL CH_2Cl_2 and cooled to -35 °C. To this solution solid LiAl(OC(CF_3)$_3$)$_4$ (51 mg, 0.0525 mmol, 1 equiv) was added and the solution was stirred for 15 min at room temperature. All solids were filtered off and solid Tl(2,6-(CF_3)-$C_6H_3CO_2$) (26.5 mg, 0.0576 mmol, 1 equiv) was added to the filtrate. The mixture was stirred under the exclusion of light for 30 min. Subsequently the reaction mixture was filtered and reduced to dryness. The residue was triturated with *n*-pentane until a yellow solid precipitated. The product can be recrystallized from chloroform. Yield: 89 mg, 92%. **^{1}H NMR** (400 MHz, CD_2Cl_2) δ = 12.81 (s, $^1J_{CH}$ = 119 Hz, 1H, Mo=CH), 8.03 (d, $^3J_{HH}$ = 8.1 Hz, 2H, Ar) , 7.88 (t, $^1J_{HH}$ = 8.0 Hz, 1H, *p*-Ar), 7.43 (s, 2H, CH=CH-NHC), 7.33 (dd, *J* = 8.0, 0.9 Hz, 2H, Ar), 7.24 (dd, *J* = 9.0, 7.0 Hz, 1H, Ar) , 7.06 (s, 2H, *m*-Ar-Mes), 6.82 (s, 2H, *m*-Ar-Mes), 2.28 (s, 6H, Me-Mes), 2.06 (s, 6H, Me-Mes), 2.04 (s, 6H, Me-Mes), 1.09 (s, 9H, *t*Bu). **^{13}C NMR** (101 MHz, CD_2Cl_2) δ = 321.5 (Mo=CH), 186.3 (NHC-NCN), 183.7 (CO_2), 149.9 (*ipso*-Ar-Imido), 142.0 (*ipso*-Ar-Mes), 136.1 (Ar-Mes), 135.3 (Ar-Mes), 134.7 (Ar-Mes), 132.7 (Ar), 131.1 (Ar), 131.0 (br m, *m*-Ar-Carboxylate), 131.0 (*m*-Ar-Mes), 130.1 (Ar-Imido), 129.1 (q, $^2J_{CF}$ = 32.8 Hz, br m, *o*-Ar-Carboxylate), 128.7 (Ar-Imido), 126.1 (CH=CH-NHC), 123.21 (q, $^1J_{CF}$ = 274.3 Hz, CF_3-Carboxylate), 121.80 (q, $^1J_{CF}$ = 291.9 Hz, OC(<u>C</u>F$_3$)$_3$), 51.0 (<u>C</u>Me$_3$), 30.3 (C<u>Me</u>$_3$), 21.4 (*p*-Me-Mes), 18.4 (*o*-Me-Mes), 18.2 (*o*-Me-Mes). **^{19}F NMR** (376 MHz, CD_2Cl_2) δ = -59.15 (s, 6F, CF_3-carboxylate), -75.75 (s, 36F, OC(CF_3)$_3$). **Elemental analysis** (%) calcd. for $C_{57}H_{41}AlCl_2F_{42}MoN_3O_6$: C, 36.89; H, 2.23; N, 2.26. Found: C, 36.77; H, 2.19; N, 2.39.

[Mo(*N*-2,6-Cl_2-C_6H_3)($CHCMe_3$)(IMes)(2,6-(CF_3)-$C_6H_3CO_2$)][B(Ar^F)$_4$], **15b**

Mo(*N*-2,6-Cl_2-C_6H_3)($CHCMe_3$)(IMes)(OTf)$_2$[15] (103 mg, 0.111 mmol, 1 equiv) was dissolved in 7 mL CH_2Cl_2 and cooled to -35 °C. To this solution solid NaB(Ar^F)$_4$ (98.3 mg, 0.111 mmol, 1 equiv) was added and the solution was stirred for 15 min at room temperature. All solids were filtered off and solid Tl(2,6-(CF_3)-$C_6H_3CO_2$) (56.3 mg, 0.122 mmol, 1.1 equiv) was added to the filtrate. The mixture was stirred under the exclusion of light for 30 min. Subsequently the reaction mixture was filtered and reduced to dryness. The residue was triturated with pentane until a yellow solid precipitated. Yield: 181 mg, 93%. **^{1}H NMR** (400 MHz, CD_2Cl_2) δ = 12.80 (s, $^1J_{CH}$ = 120.9 Hz, 1H, Mo=CH), 8.02 (d, $^3J_{HH}$ = 8.0 Hz, 2H, *m*-Ar-Imido), 7.85 (t, $^3J_{HH}$ = 7.6 Hz, 1H, *p*-Ar-Imido), 7.73 (br m, 8H, *o*-Ar-B(Ar^F)$_4$), 7.56 (br s, 8H, *p*-Ar-B(Ar^F)$_4$), 7.43 (s, 2H, CH=CH-NHC), 7.32 (d, $^4J_{HF}$ = 1.2 Hz, 1H, *m*-Ar-carboxylate), 7.30 (d, $^4J_{HF}$ = 1.1 Hz, 1H, *m*-Ar-carboxylate), 7.23 (dd, $^3J_{HH}$ = 9.0, 7.1 Hz, 1H, *p*-Ar-carboxylate), 7.05 (br s, 2H, *m*-Ar-Mes), 6.81 (br s, 2H, *m*-Ar-Mes), 2.27 (s, 6H, Me-Mes), 2.06 (s, 6H, Me-Mes), 2.04 (s, 6H, Me-Mes), 1.08 (s, 9H, *t*Bu). **^{13}C NMR** (101 MHz, CD_2Cl_2) δ = 321.5 (Mo=CH), 186.2 (NCN-NHC), 183.7 (CO_2), 162.6 (q, $^1J_{CB}$ = 49.9 Hz, *ipso*-Ar-B(Ar^F)$_4$), 149.9 (*ipso*-Ar-Imido), 141.9 (*ipso*-Ar-Mes), 136.1 (Ar-Mes), 135.4 (br m, *o*-Ar-B(Ar^F)$_4$), 135.2 (Ar-Mes), 134.7 (Ar-Mes), 132.7 (Ar), 131.1 (Ar), 131.0 (*m*-Ar-Mes), 130.1 (Ar-Imido), 129.4 (qq, $^2J_{CF}$ = 31.7 Hz, $^3J_{CB}$ = 2.8 Hz, *m*-Ar-B(Ar^F)$_4$), 129.1 (q, $^2J_{CF}$ = 32.8 Hz, <u>C</u>-CF_3-carboxylate), 128.7 (Ar-Imido), 126.1 (CH=CH-NHC), 125.1 (q, $^1J_{CF}$ = 272.4 Hz, CF_3-B(Ar^F)$_4$),), 123.2 (q, $^1J_{CF}$ = 272.5 Hz, CF_3-Carboxylate), 118.0 (sept, $^3J_{CF}$ = 3.8 Hz, *p*-Ar-B(Ar^F)$_4$), 51.0 (<u>C</u>Me$_3$), 30.3 (C<u>Me</u>$_3$), 21.4 (*p*-Me-Mes), 18.4 (*o*-Me-Mes), 18.2 (*o*-Me-Mes). **^{19}F NMR** (376 MHz, CD_2Cl_2) δ = -59.14 (s, 6F, CF_3-Carboxylate), -62.87 (s, 24F, B(Ar^F)$_4$). **Elemental analysis** (%) calcd. for $C_{80}H_{60}BF_{30}MoN_3O_2$: C, 54.22; H, 3.14; N, 2.37. Found: C, 54.06; H, 3.21; N, 2.51.

[Mo(*N*-Ad)($CHCMe_2Ph$)($IMesH_2$)(2,6-(CF_3)-$C_6H_3CO_2$)][Al(OC(CF_3)$_3$)$_4$], **17a**

Mo(N-Ad)($CHCMe_2Ph$)($IMesH_2$)(Cl)$_2$[19] (67 mg, 0.0887 mmol, 1 equiv) was dissolved in 3 mL CH_2Cl_2 and cooled to -35 °C. To this solution solid LiAl(OC(CF_3)$_3$)$_4$ (86.5 mg, 0.0887 mmol, 1 equiv) was added and the solution was stirred for 15 min at room temperature. All solids were filtered off and solid Tl(2,6-(CF_3)-$C_6H_3CO_2$) (41 mg, 0.0887 mmol, 1 equiv) was added to the filtrate. The mixture was stirred under the exclusion of light for one hour. Subsequently, the reaction mixture was filtered and reduced to dryness. The residue was triturated with *n*-pentane until a pale yellow solid precipitated. The product can be recrystallized from chloroform. Yield: 153 mg, 90%. **^{1}H NMR** (400 MHz, $CDCl_3$) δ = 13.09 (s, $^1J_{CH}$ = 118 Hz, 1H; Mo=CH), 8.01 (d, $^3J_{HH}$ = 8.1 Hz, 2H; *m*-Ar), 7.85 (t, $^3J_{HH}$ = 8.0 Hz, 1H; *p*-Ar), 7.34 (m, 2H; *m*-Ar), 7.28 (m, 1H; *p*-Ar), 7.17 (m, 2H; *o*-Ar), 7.03 (s, 2H; *m*-Ar-Mes), 6.83 (s, 2H; *m*-Ar-Mes), 4.07 (m, 2H, CH_2-NHC), 3.95 (m, 2H, CH_2-NHC), 2.32 (s, 6H; Me-Mes), 2.29 (s, 6H; Me-Mes), 2.14 (s, 6H, Me-Mes), 1.85 (br m, 3H; Ad), 1.65 (s, 3H; C<u>Me</u>$_2$Ph), 1.53 (s, 3H; C<u>Me</u>$_2$Ph), 1.45 (br m, 3H; Ad), 1.34 (br m, 6H; Ad), 1.24 (br m, 3H; Ad). **^{13}C NMR** (101 MHz, $CDCl_3$) δ = 321.4 (Mo=CH), 207.6 (NCN-NHC), 187.0 (CO_2), 147.4 (*ipso*-Ar-CMe_2Ph), 140.8 (*ipso*-Ar-Mes), 136.6 (Ar-Mes), 135.5 (Ar-Mes), 134.5 (Ar-Mes), 132.1 (*p*-Ar-carboxylate), 131.0 (*m*-Ar-Mes), 130.8 (*m*-Ar-Mes), 130.4 (q, $^3J_{CF}$ = 4.3 Hz, *m*-Ar-carboxylate), 128.8 (Ar-CMe_2Ph), 128.6 (q, $^2J_{CF}$ = 32.8 Hz, *o*-Ar-carboxylate), 127.2 (Ar-CMe_2Ph), 126.4 (Ar-CMe_2Ph), 122.8 (q, $^1J_{CF}$ = 274.6 Hz, CF_3-carboxylate), 121.4 (q, $^1J_{CF}$ = 292.8 Hz, OC(<u>C</u>F$_3$)$_3$), 82.0 (N-Ad), 55.7 (<u>C</u>Me$_2$Ph), 52.2 (CH_2-NHC), 42.1 (CH_2-Ad), 35.0 (CH_2-Ad), 32.8 (CH-Ad), 29.5 (CH-Ad), 29.3 (C<u>Me</u>$_2$Ph), 21.1 (*p*-Me-Mes), 17.95 (*o*-Me-Mes), 17.9 (*o*-Me-Mes). **^{19}F NMR** (376 MHz, $CDCl_3$) δ = -

59.22 (s, 6F; Ar-CF_3), -75.48 (s, 36F; $OC(CF_3)_3$). **Elemental analysis** (%) calcd. for $C_{66}H_{56}AlF_{42}MoN_3O_6$: C, 41.55; H, 2.96; N, 2.20. Found: C, 41.56; H, 2.97; N, 2.34.

[Mo(*N*-Ad)($CHCMe_2Ph$)(5-*i*Pr)(2,6-(CF_3)-$C_6H_3CO_2$)][Al($OC(CF_3)_3$)$_4$], **17b**

Mo(N-Ad)($CHCMe_2Ph$)(5-*i*Pr)$(Cl)_2$[19] (58 mg, 0.096 mmol, 1 equiv) was dissolved in 3 mL CH_2Cl_2 and cooled to -35 °C. To this solution solid LiAl($OC(CF_3)_3$)$_4$ (93.6 mg, 0.096 mmol, 1 equiv) was added and the solution was stirred for 15 min at room temperature. All solids were filtered off and solid Tl(2,6-(CF_3)-$C_6H_3CO_2$) (47 mg, 0.1 mmol, 1.05 equiv) was added to the filtrate. The mixture was stirred under the exclusion of light for one hour. Subsequently the reaction mixture was filtered and reduced to dryness. The residue was triturated with *n*-pentane until a pale yellow solid precipitated. The product can be recrystallized from chloroform. Yield: 142 mg, 84%.**^{1}H NMR** (400 MHz, CD_2Cl_2) δ = 13.80 (s, $^1J_{CH}$ = 116.4 Hz, 1H, Mo=CH), 8.05 (d, $^3J_{HH}$ = 8.0 Hz, 2H, *m*-Ar), 7.89 (t, $^3J_{HH}$ = 8.0 Hz, 1H, *p*-Ar), 7.41 (s, 2H, Ar), 7.39 (s, 2H, Ar), 7.30 (m, 1H, Ar), 7.28 (s, 2H, Ar), 4.21 (sept, $^3J_{HH}$ = 6.6 Hz, 2H, CH-*i*Pr), 2.14 (br m, 9H, Ad), 2.09 (s, 3H, C<u>Me</u>$_2$Ph), 1.70 (br m, 6H, Ad), 1.67 (s, 3H, C<u>Me</u>$_2$Ph), 1.33 (d, $^3J_{HH}$ = 6.6 Hz, 6H, CH_3-*i*Pr), 1.27 (d, $^3J_{HH}$ = 6.6 Hz, 6H, CH_3-*i*Pr). **^{13}C NMR** (101 MHz, CD_2Cl_2) δ = 308.7 (Mo=CH), 188.5 (NCN-NHC), 175.5 (CO_2), 146.0 (*ipso*-CMe_2Ph), 132.7 (*p*-Ar-carboxylate), 131.0 (q, $^3J_{CF}$ = 4.1 Hz, *m*-Ar-carboxylate), 129.7 (Ar-CMe_2Ph), 128.8 (q, $^2J_{CF}$ = 32.8 Hz, *o*-Ar-carboxylate), 128.0 (Ar-CMe_2Ph), 126.2 (Ar-CMe_2Ph), 123.5 (q, $^1J_{CF}$ = 274.4 Hz, CF_3-carboxylate), 121.8 (q, $^1J_{CF}$ = 292.4 Hz, OC(<u>C</u>F$_3$)$_3$), 120.6 (CH=CH-NHC), 80.1 (N-Ad), 55.1 (CH-*i*Pr), 54.6 (<u>C</u>Me$_2$Ph), 44.7 (CH_2-Ad), 35.8 (CH_2-Ad), 31.9 (CH-Ad), 30.0 (C<u>Me</u>$_2$Ph), 29.6 (C<u>Me</u>$_2$Ph), 23.9 (CH_3-*i*Pr), 23.7 (CH_3-*i*Pr). **^{19}F NMR** (376 MHz, CD_2Cl_2) δ = -59.53 (s, 6F, CF_3-carboxylate), -75.75 (s, 36F, $OC(CF_3)_3$). **Elemental analysis** (%) calcd. for $C_{54}H_{46}AlF_{42}MoN_3O_6 \cdot CHCl_3$: C, 35.25; H, 2.58; N, 2.24. Found: C, 35.17; H, 2.56; N, 2.35.

NMR Spectra of Organometallic Complexes

^{1}H NMR (400 MHz, CD_2Cl_2) δ = 13.09, 7.72, 7.56, 7.45, 7.40, 7.31, 7.23, 7.15, 3.62, 3.48, 3.09, 1.82, 1.76, 1.15.

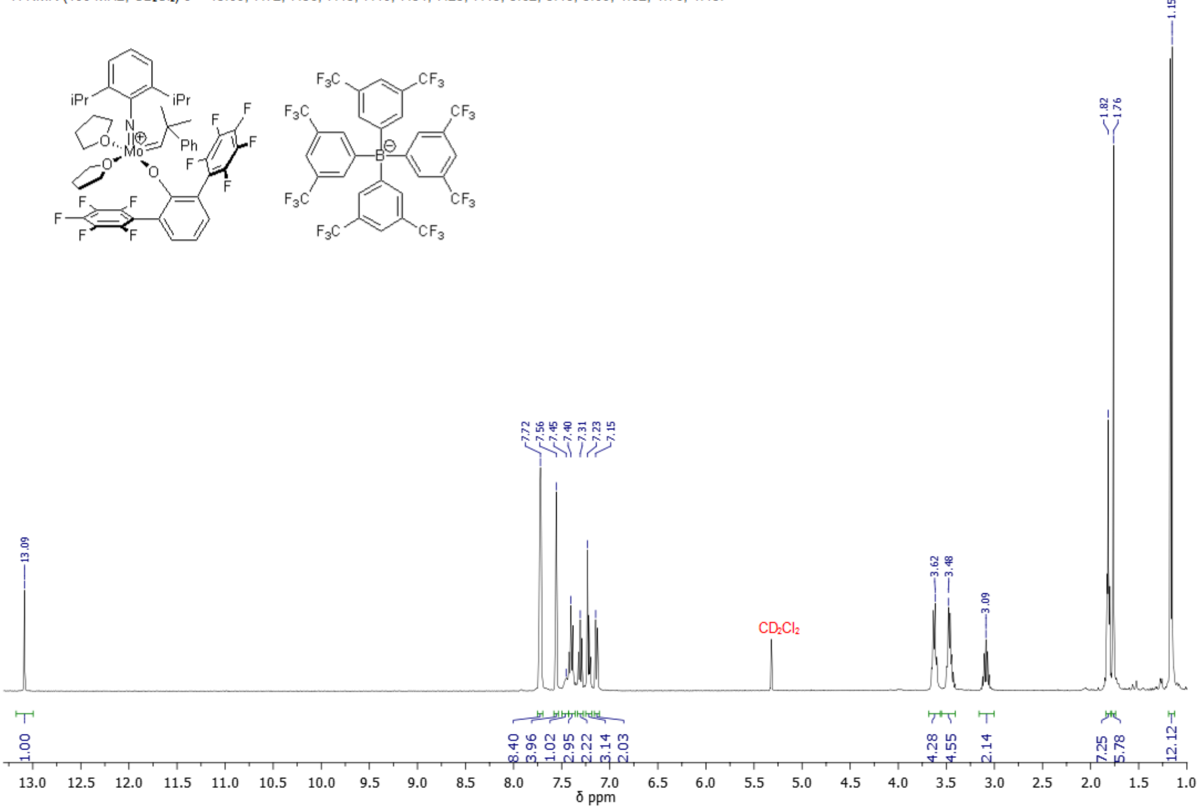

Figure S1. ^{1}H NMR (400 MHz, 25 °C, CD_2Cl_2) of **2a**.

^{19}F NMR (376 MHz, CD_2Cl_2) δ = -62.90, -138.40, -138.92, -151.36, -153.14, -159.61, -160.78.

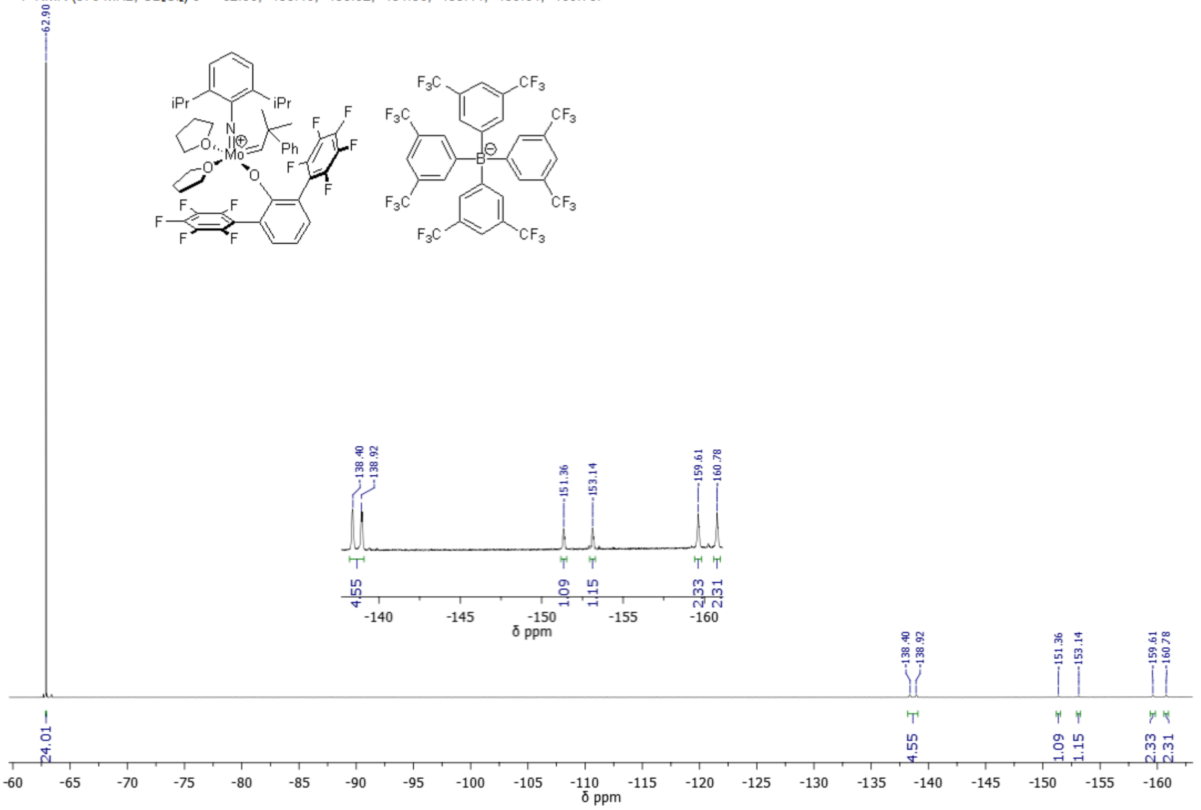

Figure S2. ^{19}F NMR (375 MHz, 25 °C, CD_2Cl_2) of **2a**.

^{13}C NMR (101 MHz, CD_2Cl_2) δ = 298.9, 162.3, 160.4, 151.9, 146.9, 144.9, 135.6, 134.1, 130.9, 129.9, 129.4, 128.7, 125.8, 125.2, 125.1, 122.3, 118.0, 80.8, 57.7, 30.3, 28.9, 26.3, 24.7.

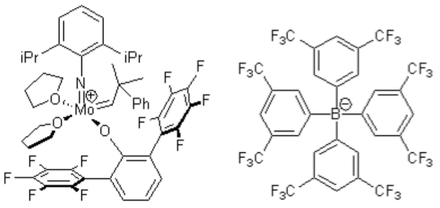

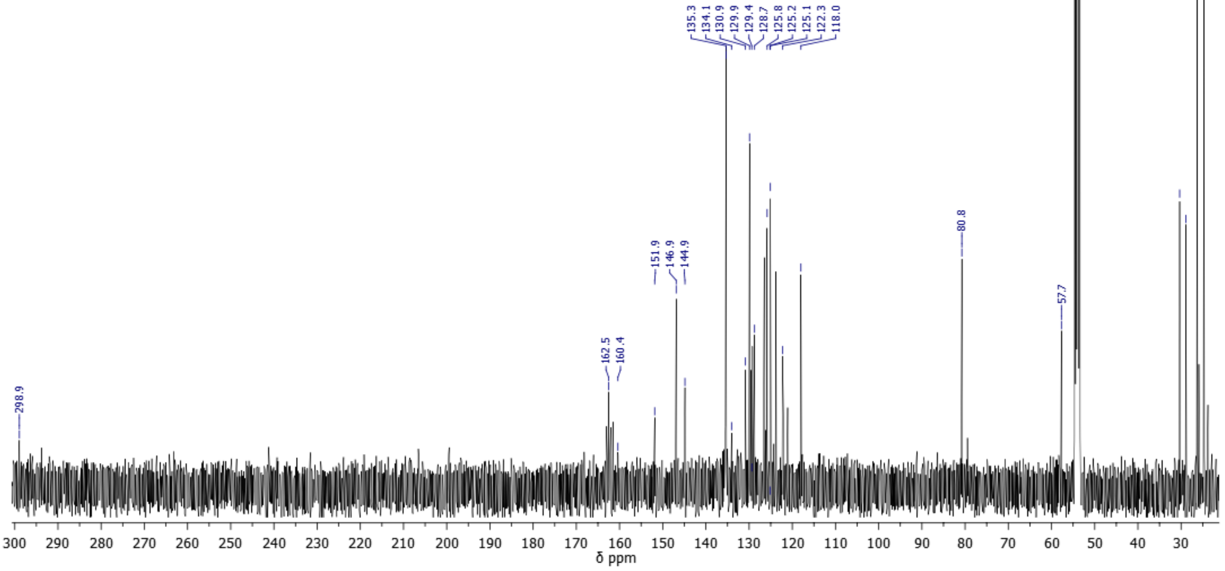

Figure S3. ^{13}C NMR (100 MHz, 25 °C, CD_2Cl_2) of **2a**.

^{1}H NMR (400 MHz, $CDCl_3$) δ = 14.08, 7.73, 7.55, 7.31, 7.24, 7.19, 7.17, 6.24, 6.19, 5.80, 3.73, 2.96, 2.35, 1.75, 1.59, 1.20.

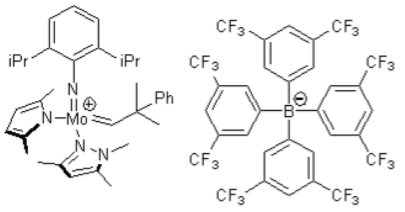

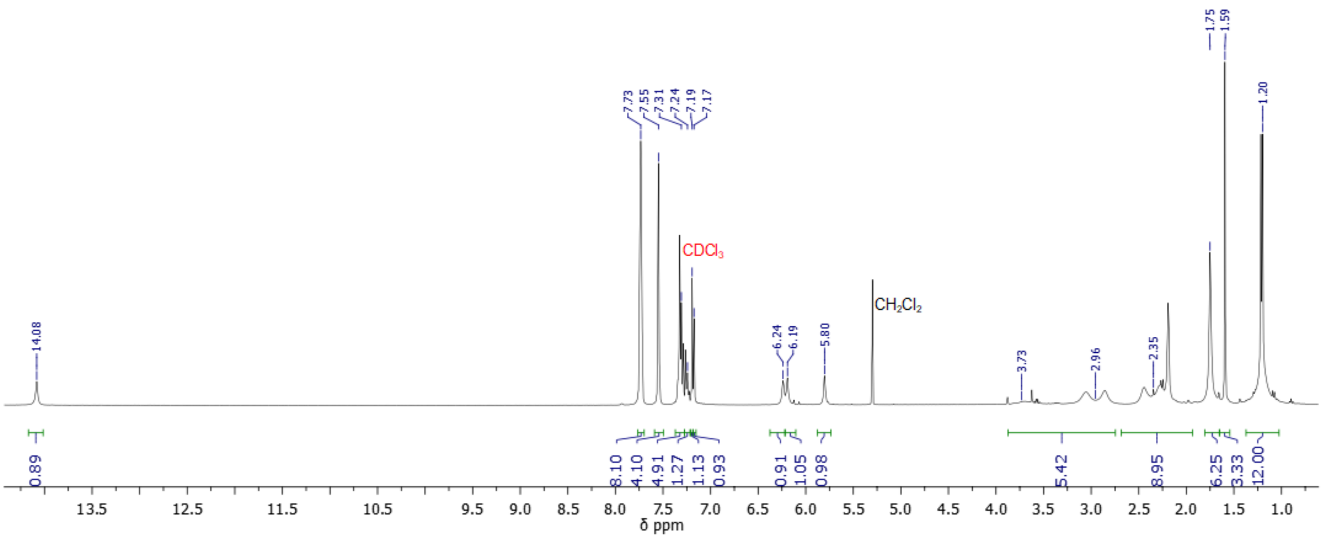

Figure S4. ^{1}H NMR (400 MHz, 25 °C, $CDCl_3$) of **2b**.

^{19}F NMR (376 MHz, $CDCl_3$) δ = -62.34.

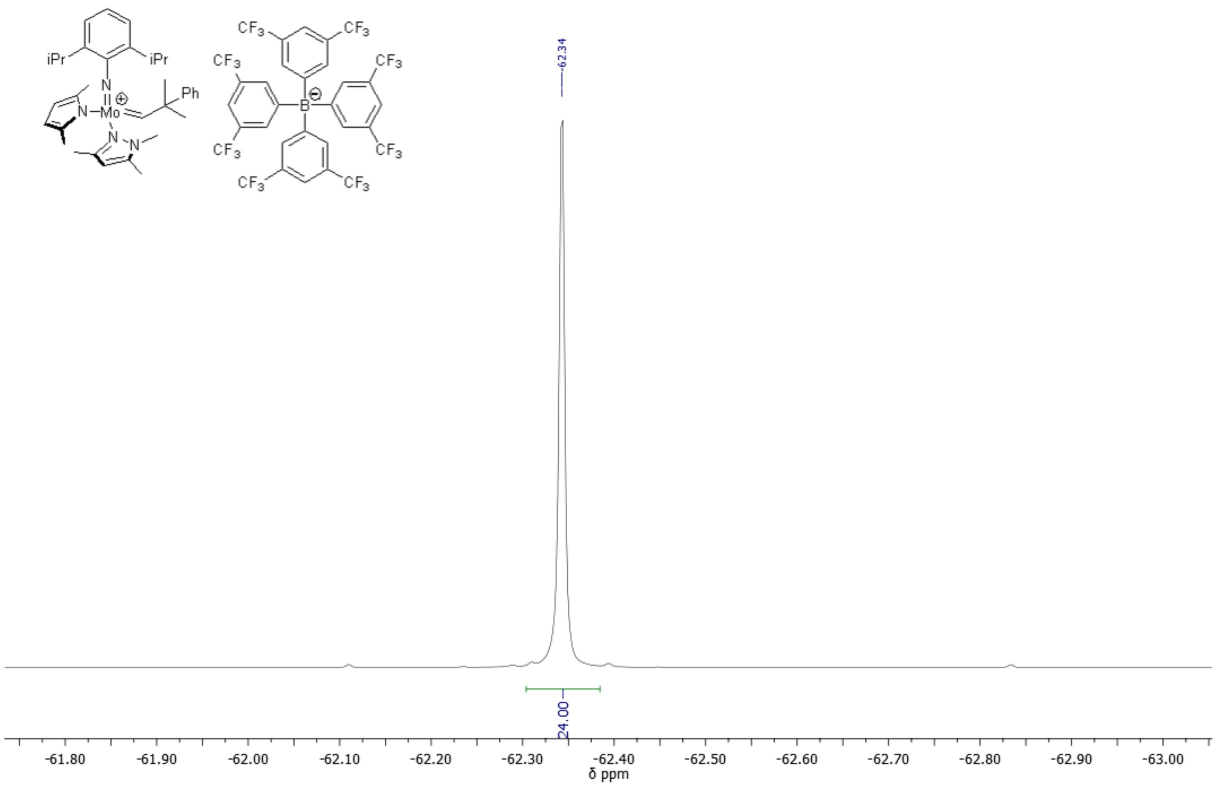

Figure S5. ^{19}F NMR (375 MHz, 25 °C, $CDCl_3$) of **2b**.

^{13}C NMR (101 MHz, $CDCl_3$) δ = 333.6, 162.1, 153.7, 151.3, 148.9, 146.9, 145.2, 142.7, 135.0, 129.9, 129.1, 128.9, 126.4, 124.8, 124.6, 117.6, 110.5, 104.7, 104.0, 61.4, 53.6, 37.3, 30.4, 30.1, 28.3, 25.0, 23.5, 18.2, 17.6, 15.0, 12.7.

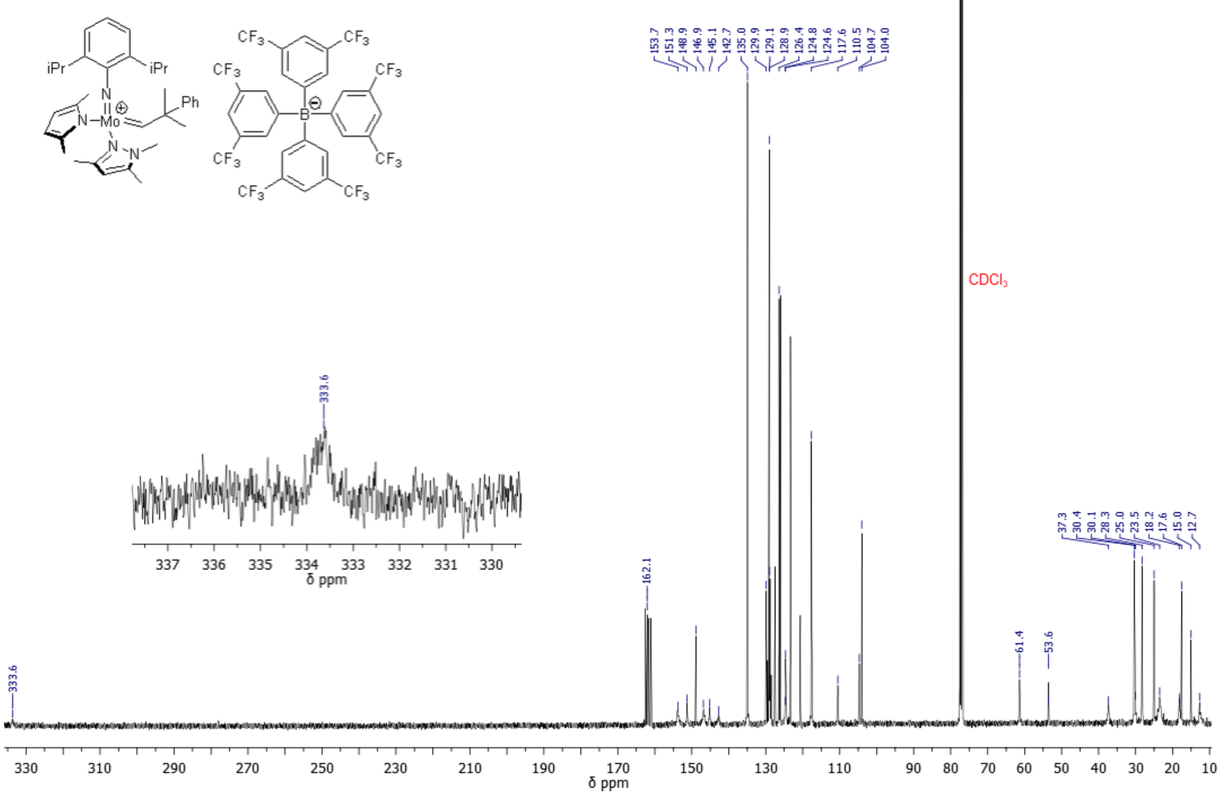

Figure S6. ^{13}C NMR (100 MHz, 25 °C, $CDCl_3$) of **2b**.

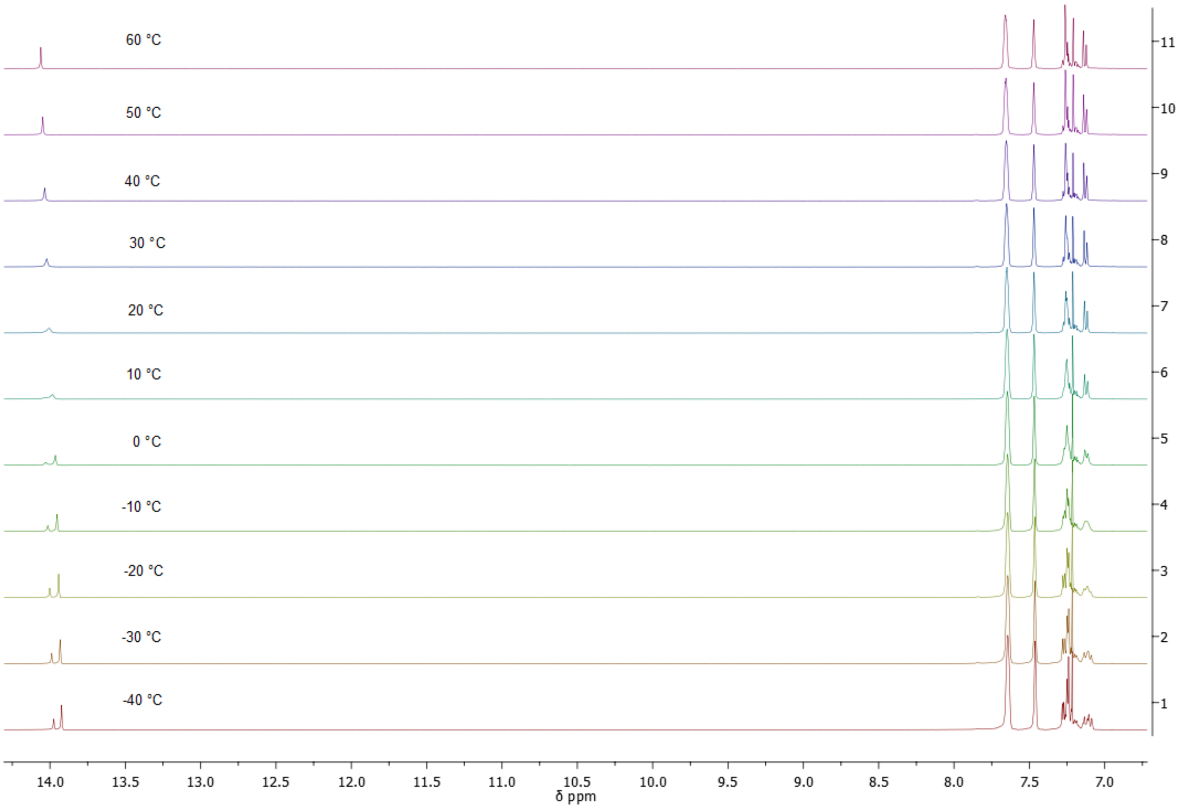

Figure S7. Variable temperature ^{1}H NMR (400 MHz, $CDCl_3$) of **2b**, alkylidene and aromatic region.

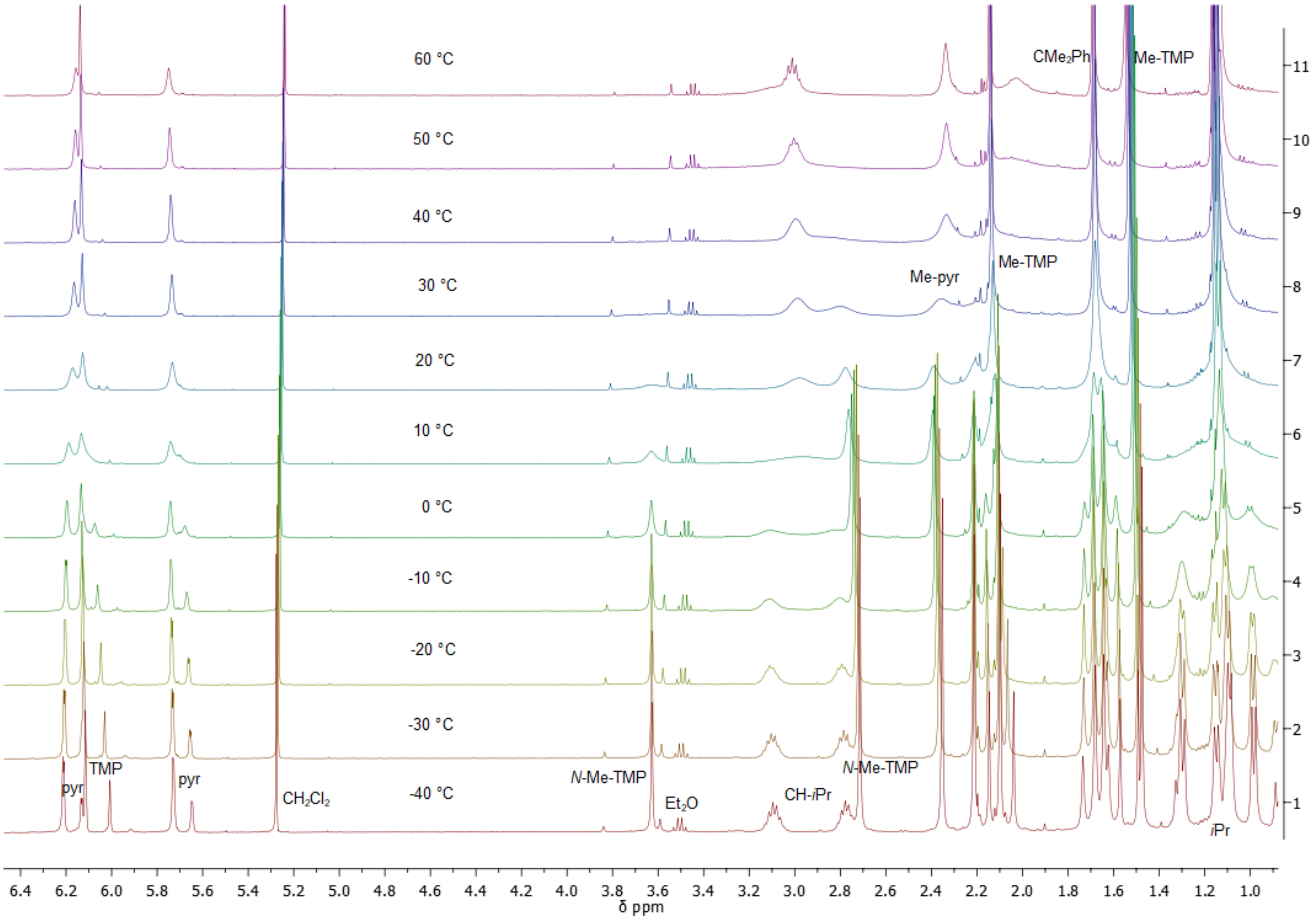

Figure S8. Variable temperature ^{1}H NMR (400 MHz, $CDCl_3$) of **2b**, expansion.

^{1}H NMR (400 MHz, $CDCl_3$) δ = 12.59, 7.72, 7.52, 7.39, 7.27, 7.27, 7.26, 7.25, 7.21, 7.17, 6.97, 6.82, 2.20, 2.02, 1.08.

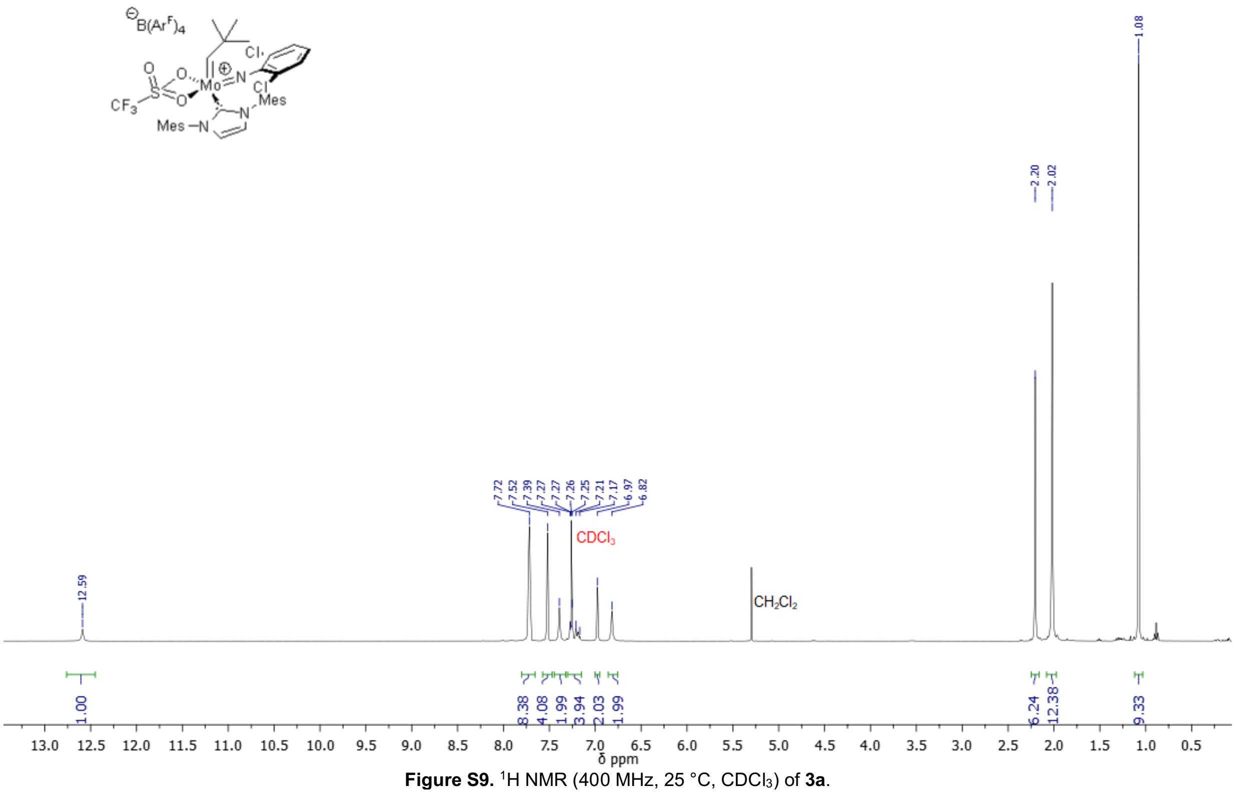

Figure S9. ^{1}H NMR (400 MHz, 25 °C, $CDCl_3$) of **3a**.

^{19}F NMR (376 MHz, $CDCl_3$) δ = -62.41, -73.72.

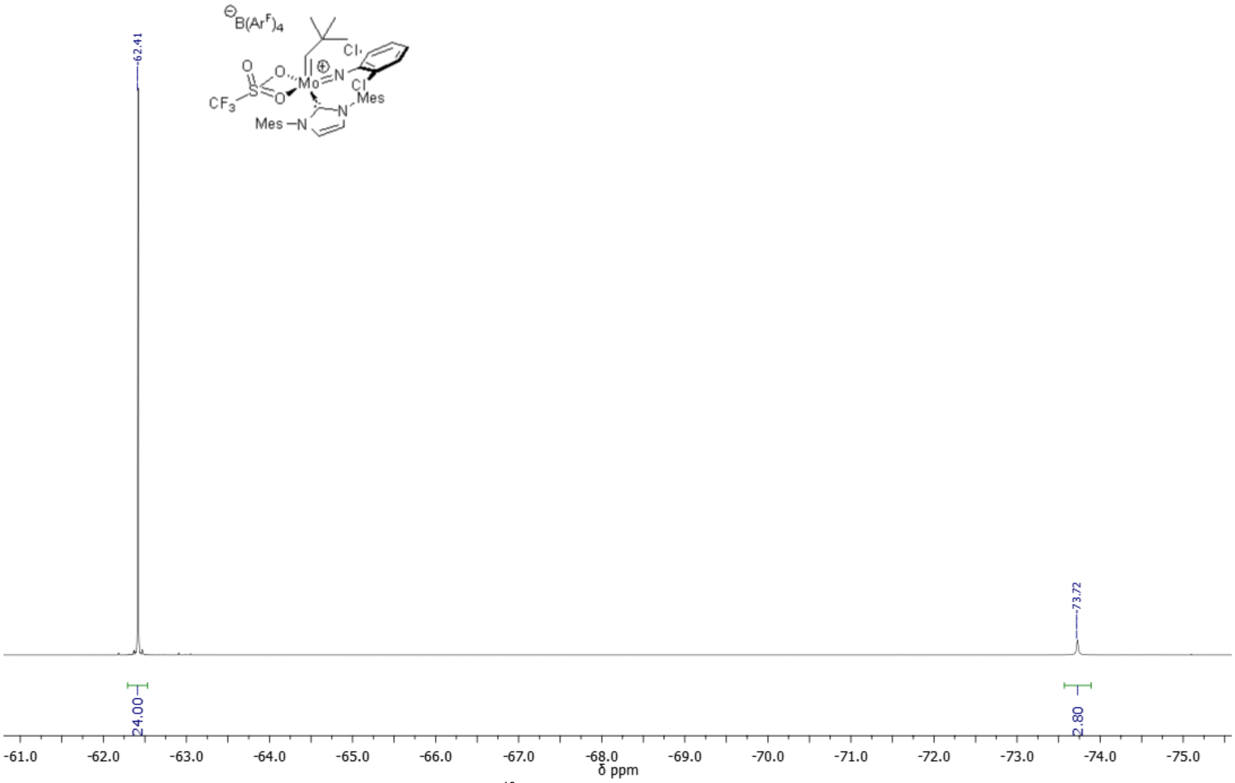

Figure S10. ^{19}F NMR (375 MHz, 25 °C, $CDCl_3$) of **3a**.

^{13}C NMR (101 MHz, $CDCl_3$) δ = 328.9, 181.8, 162.6, 162.1, 161.6, 161.1, 149.4, 142.1, 135.5, 135.0, 134.6, 133.7, 131.8, 130.7, 129.9, 129.3, 129.2, 129.2, 129.2, 129.0, 128.9, 128.9, 128.9, 128.8, 128.6, 126.1, 126.1, 123.4, 120.7, 117.6, 51.9, 30.1, 21.1, 18.2, 17.9.

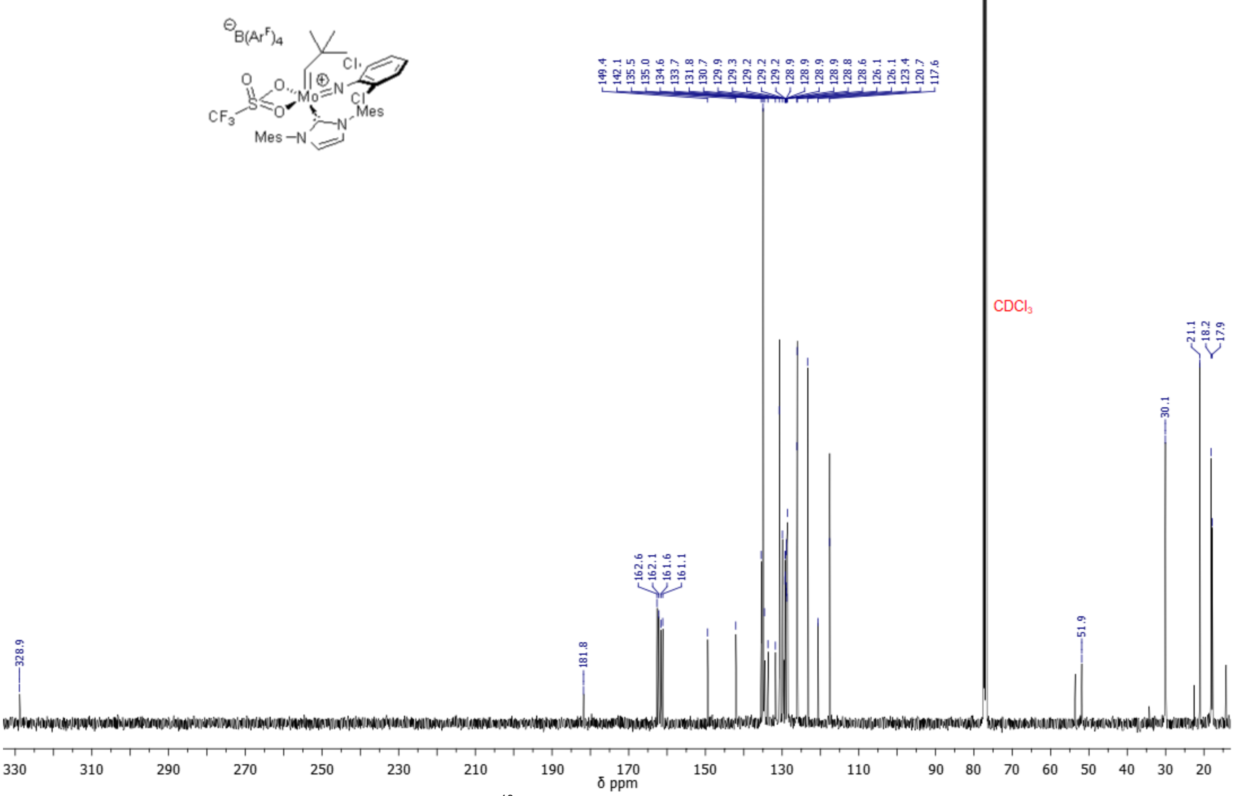

Figure S11. ^{13}C NMR (100 MHz, 25 °C, $CDCl_3$) of **3a**.

^{1}H NMR (400 MHz, $CDCl_3$) δ = 13.52, 7.70, 7.52, 7.19, 7.15, 7.07, 6.99, 6.63, 2.26, 2.05, 1.99, 1.96, 0.88.

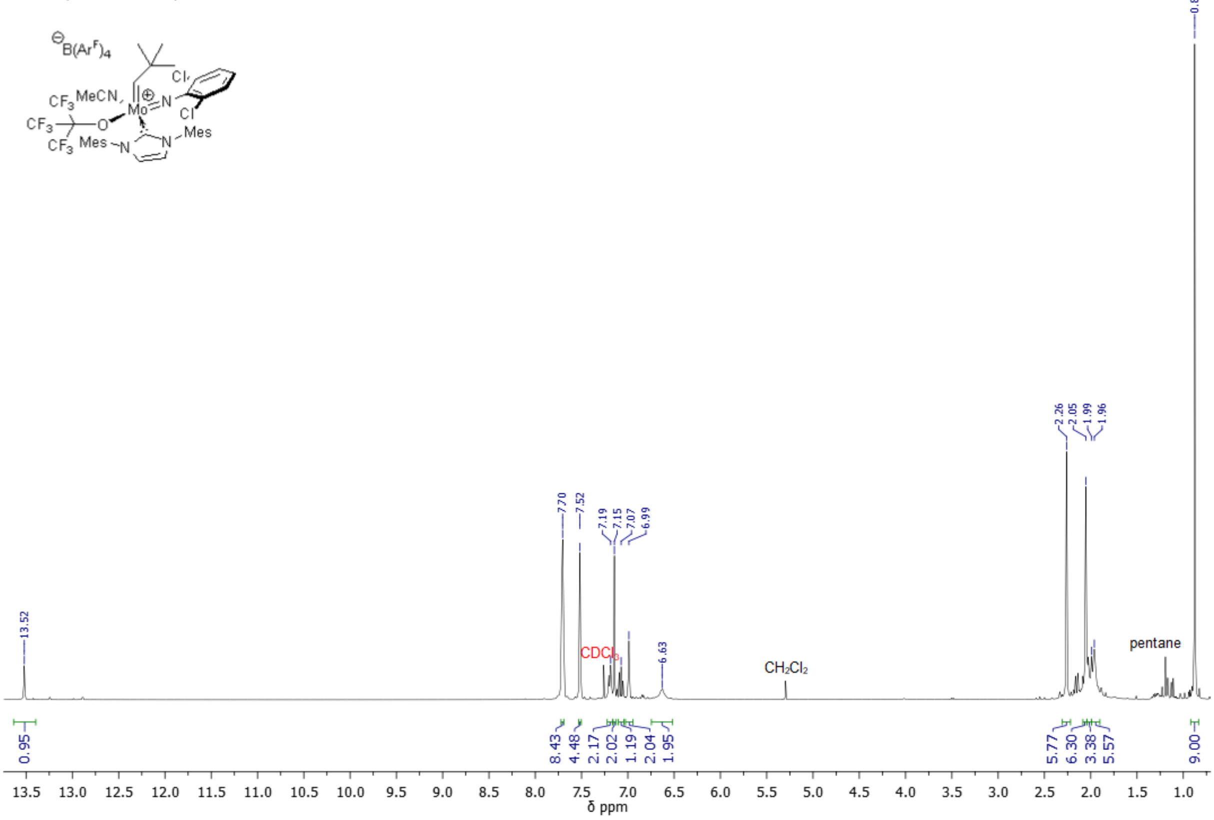

Figure S12. ^{1}H NMR (400 MHz, 25 °C, $CDCl_3$) of **3b**.

^{19}F NMR (376 MHz, $CDCl_3$) δ = -62.43, -71.61.

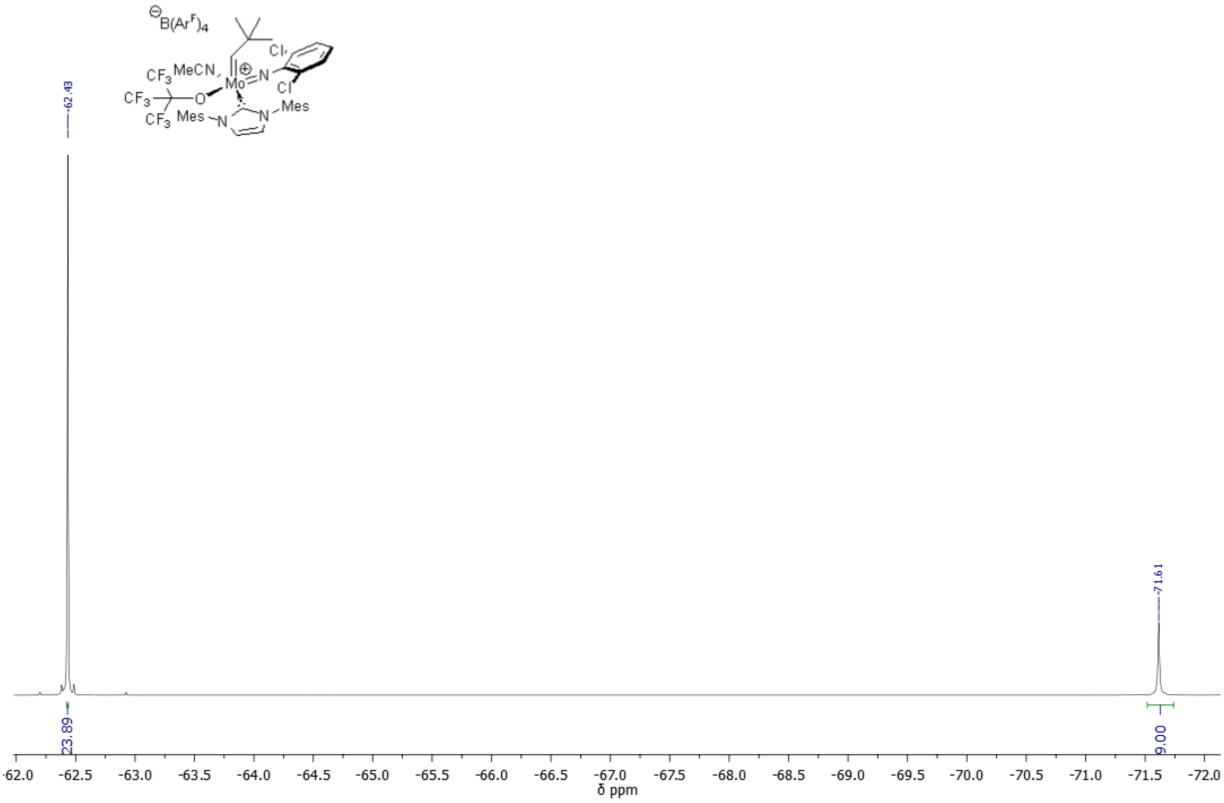

Figure S13. ^{19}F NMR (375 MHz, 25 °C, $CDCl_3$) of **3b**.

^{13}C NMR (101 MHz, $CDCl_3$) δ = 332.0, 183.2, 161.6, 148.8, 141.4, 135.6, 135.4, 134.9, 134.8, 130.5, 129.4, 125.7, 124.7, 121.2, 117.6, 85.4, 50.9, 29.7, 21.0, 18.1, 18.0, 2.3.

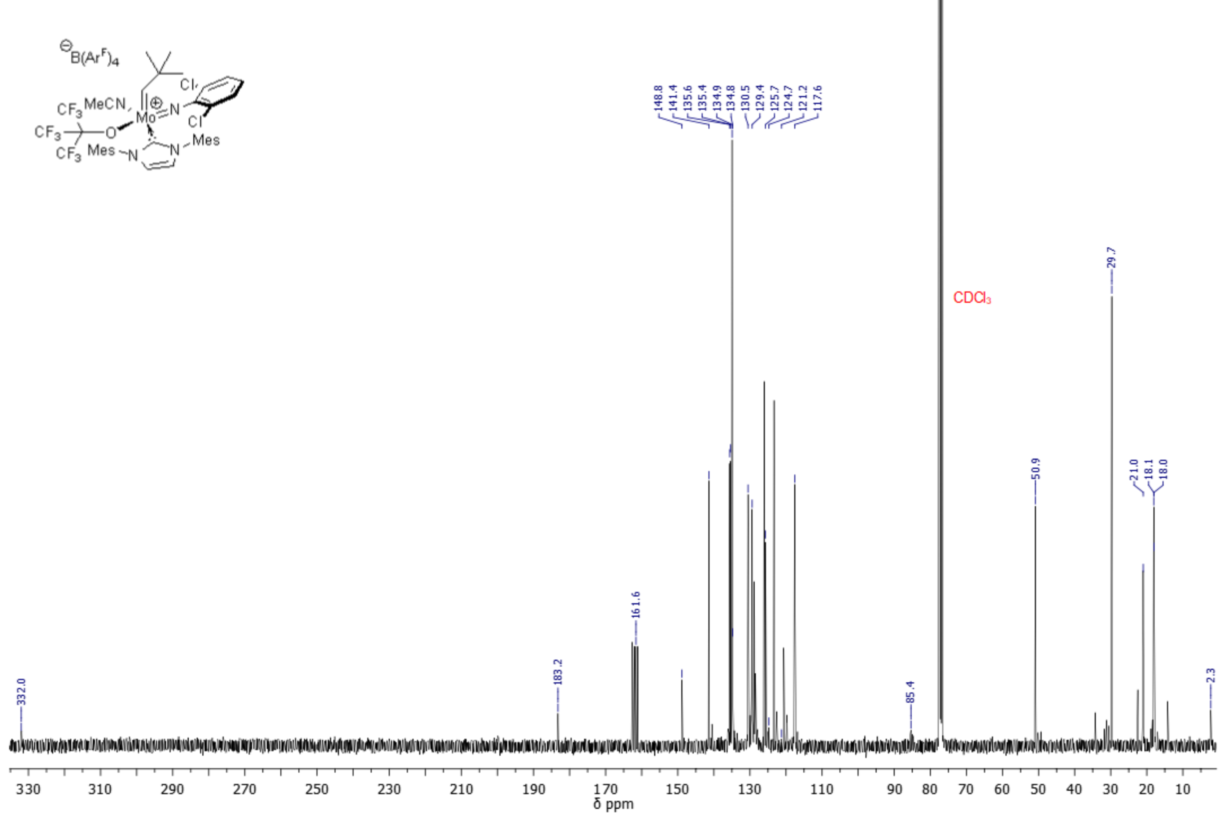

Figure S14. ^{13}C NMR (100 MHz, 25 °C, $CDCl_3$) of **3b**.

^1H NMR (400 MHz, $CDCl_3$) δ = 12.72, 7.70, 7.51, 7.28, 7.21, 6.90, 6.79, 4.14, 2.27, 2.16, 1.08.

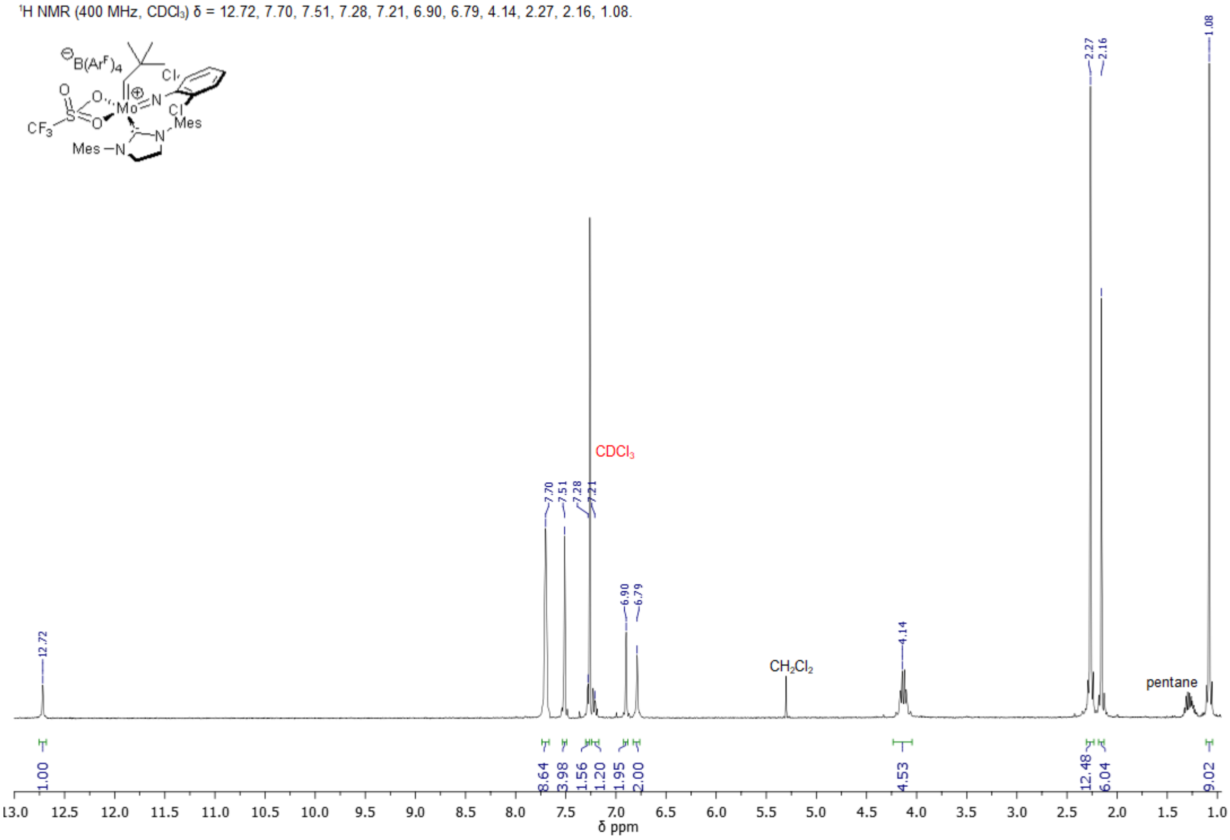

Figure S15. ^1H NMR (400 MHz, 25 °C, $CDCl_3$) of **3c**.

^{13}C NMR (101 MHz, $CDCl_3$) δ = 336.5, 207.0, 161.6, 148.6, 140.5, 136.3, 135.8, 134.9, 131.5, 131.1, 129.8, 128.3, 117.7, 52.0, 51.4, 29.7, 20.9, 18.5, 18.3.

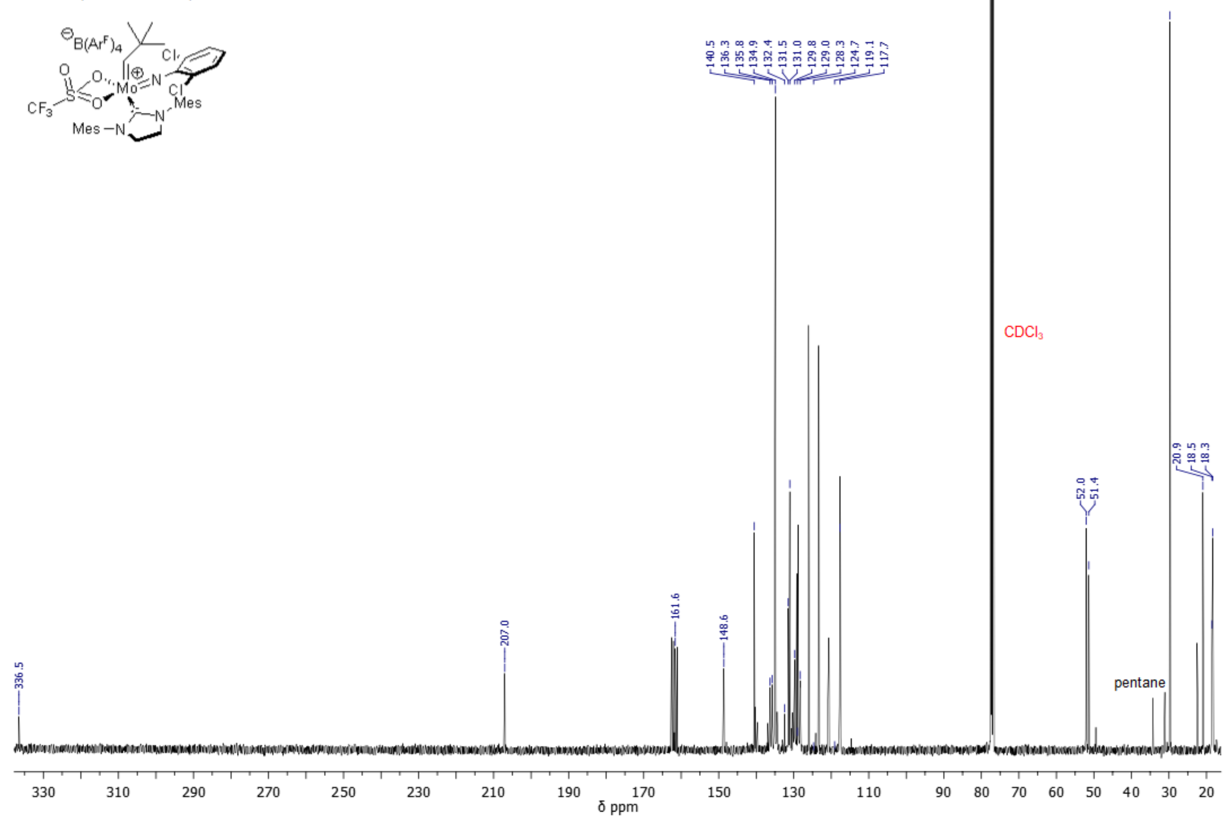

Figure S16. ^{13}C NMR (100 MHz, 25 °C, $CDCl_3$) of **3c**.

^{19}F NMR (376 MHz, $CDCl_3$) δ = -62.42, -73.78.

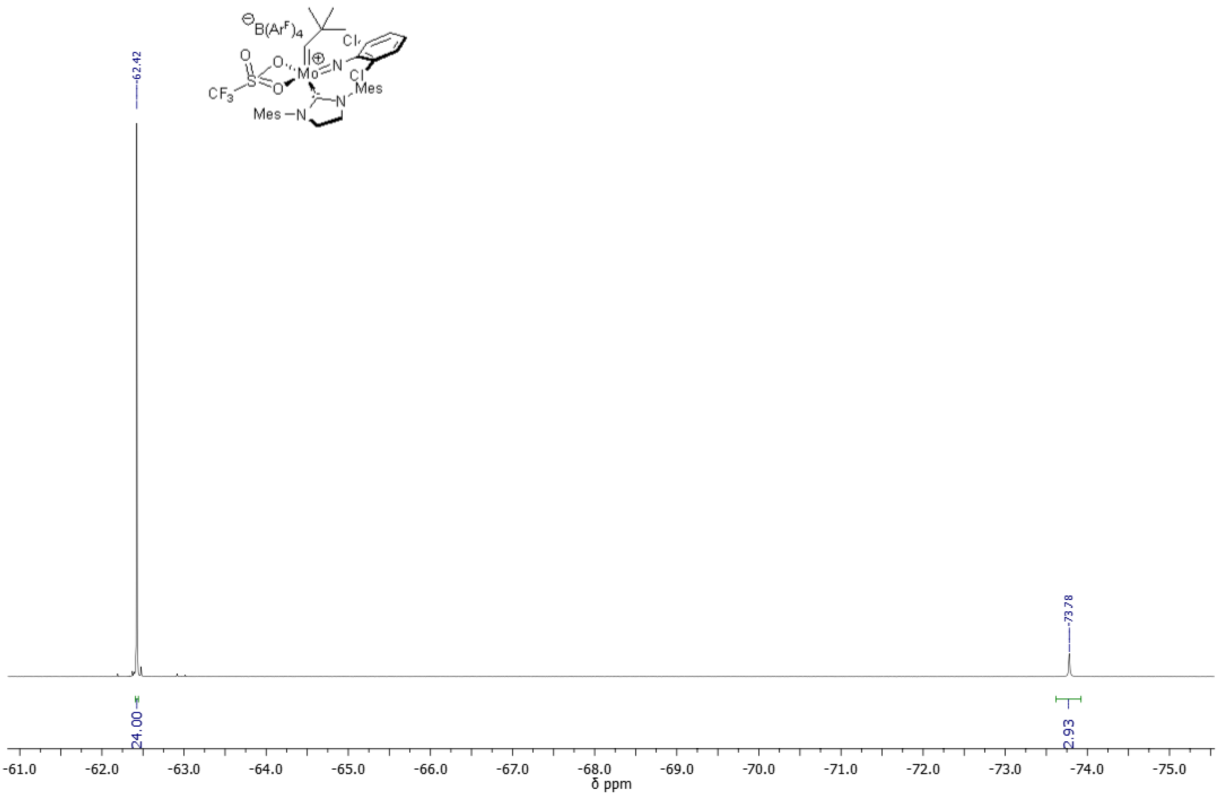

Figure S17. ^{19}F NMR (375 MHz, 25 °C, $CDCl_3$) of **3c**.

^{1}H NMR (400 MHz, CD_2Cl_2) δ = 12.95, 7.27, 7.14, 7.01, 6.73, 2.24, 2.11, 2.09, 1.14.

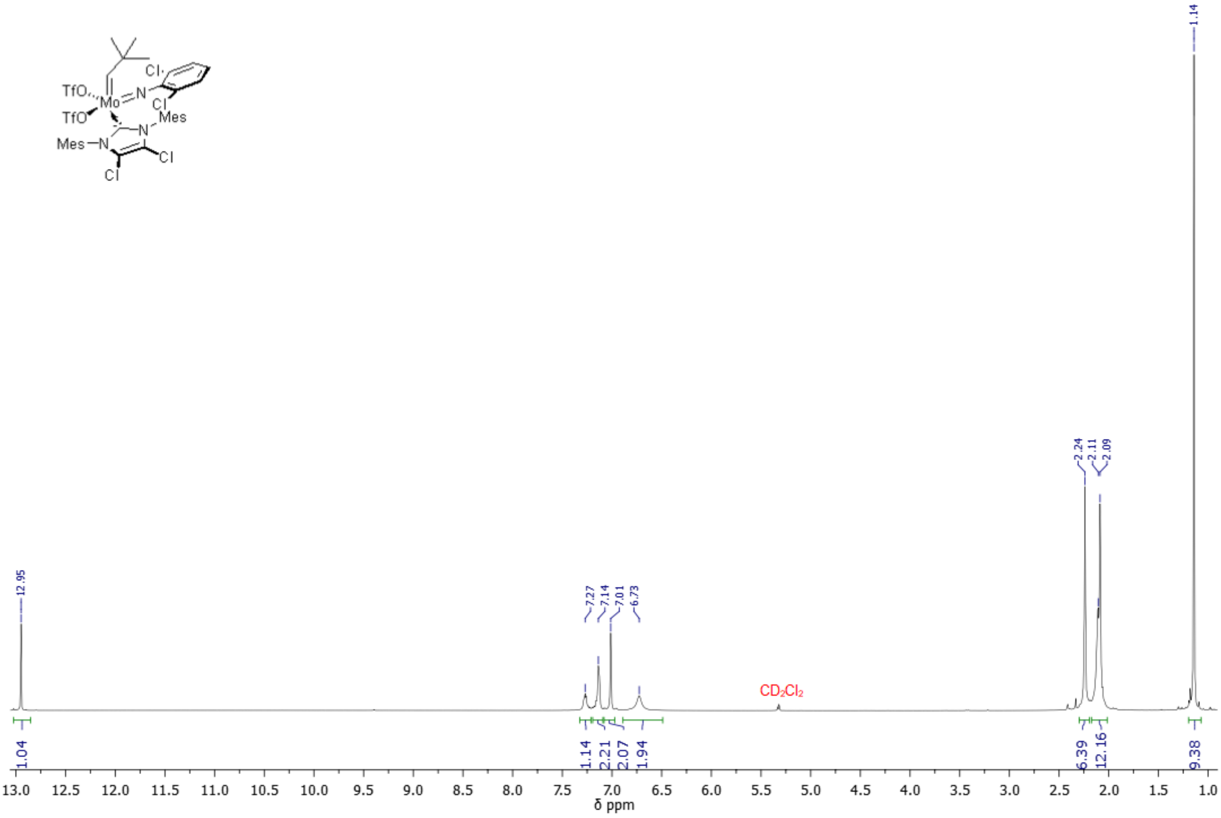

Figure S18. ^{1}H NMR (400 MHz, 25 °C, CD_2Cl_2) of **3d-p1**.

^{13}C NMR (101 MHz, CD_2Cl_2) δ = 329.5, 185.5, 149.6, 142.0, 142.0, 136.9, 136.6, 132.7, 131.0, 130.6, 129.5, 128.3, 127.9, 122.1, 119.7, 50.7, 31.3, 21.4, 18.9, 18.9.

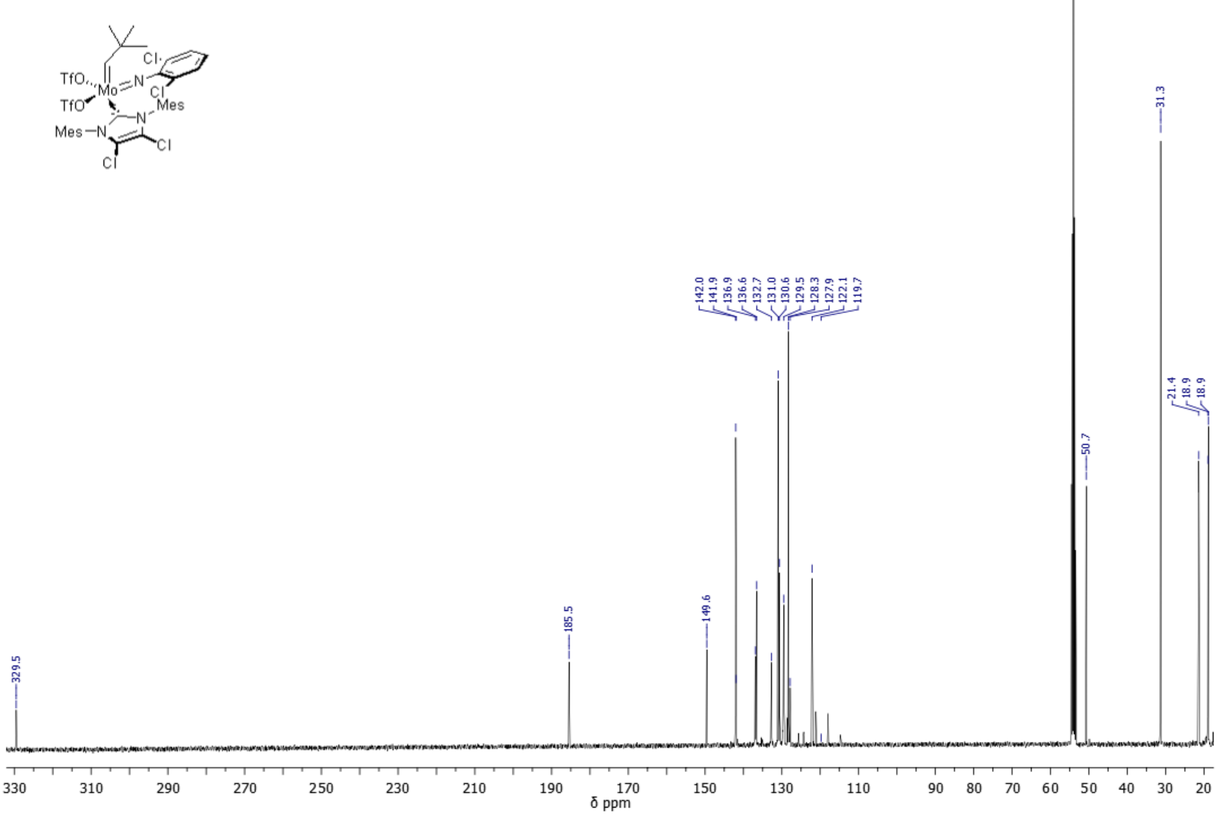

Figure S19. ^{13}C NMR (100 MHz, 25 °C, CD_2Cl_2) of **3d-p1**.

^{19}F NMR (376 MHz, CD_2Cl_2) δ = -75.05, -76.58.

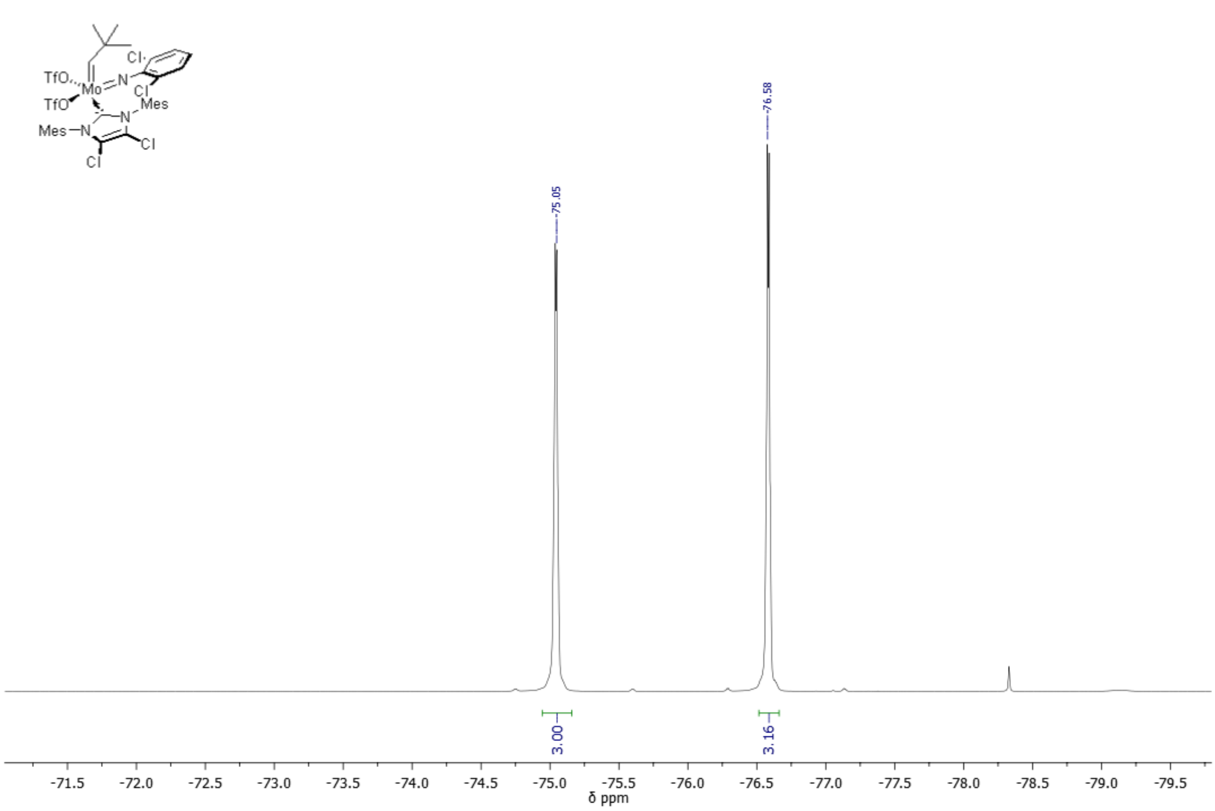

Figure S20. ^{19}F NMR (375 MHz, 25 °C, CD_2Cl_2) of **3d-p1**.

^{1}H NMR (400 MHz, $CDCl_3$) δ = 12.68, 7.74, 7.54, 7.26, 7.20, 7.03, 6.88, 2.23, 2.05, 1.10.

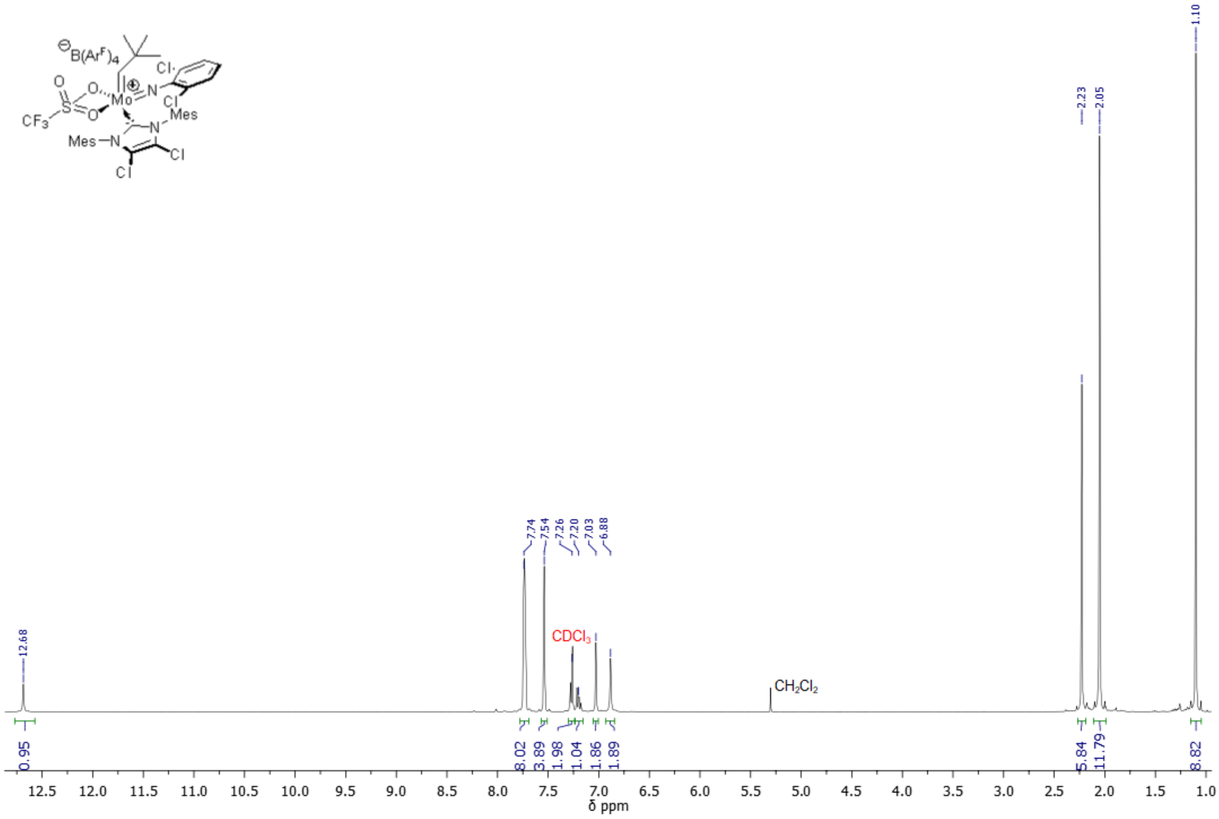

Figure S21. ^{1}H NMR (400 MHz, 25 °C, $CDCl_3$) of **3d**.

^{13}C NMR (101 MHz, $CDCl_3$) δ = 331.4, 181.4, 161.9, 149.5, 143.2, 136.2, 135.2, 135.0, 132.2, 131.1, 131.0, 130.2, 129.1, 128.6, 122.9, 120.6, 117.6, 52.2, 30.1, 21.2, 18.3, 17.9.

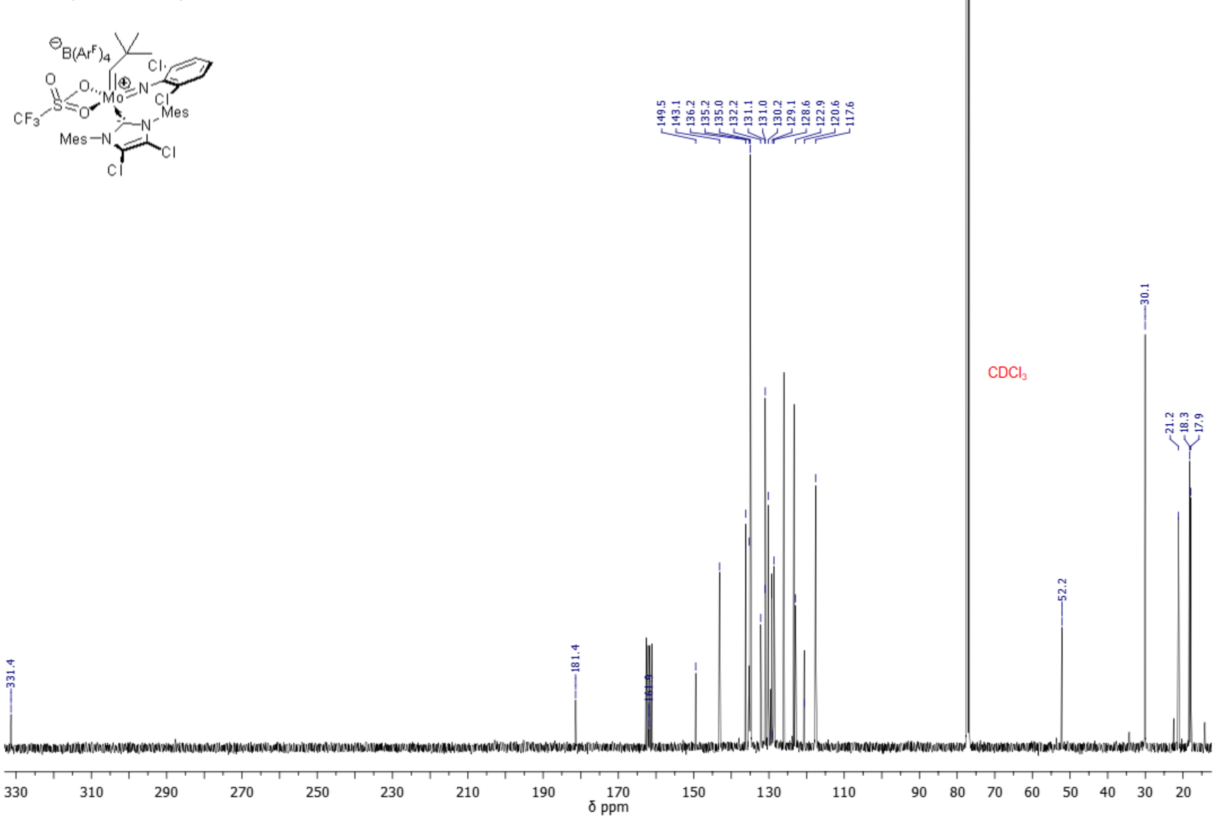

Figure S22. ^{13}C NMR (100 MHz, 25 °C, $CDCl_3$) of **3d**.

^{19}F NMR (376 MHz, $CDCl_3$) δ = -62.41, -73.69.

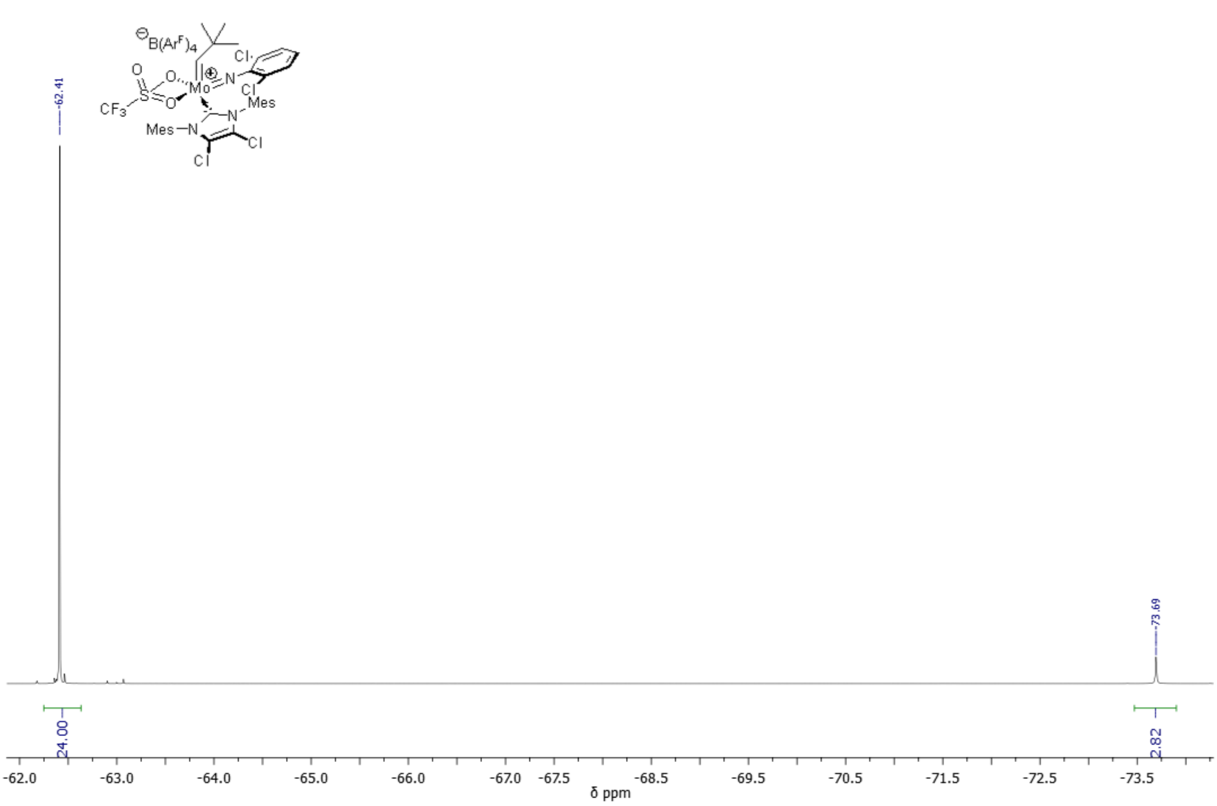

Figure S23. ^{19}F NMR (375 MHz, 25 °C, $CDCl_3$) of **3d**.

^{1}H NMR (400 MHz, $CDCl_3$) δ = 13.28, 7.72, 7.52, 7.48, 7.43, 7.32, 7.23, 7.21, 7.14, 7.04, 6.97, 6.66, 2.23, 2.08, 1.92, 1.80, 1.79, 1.01.

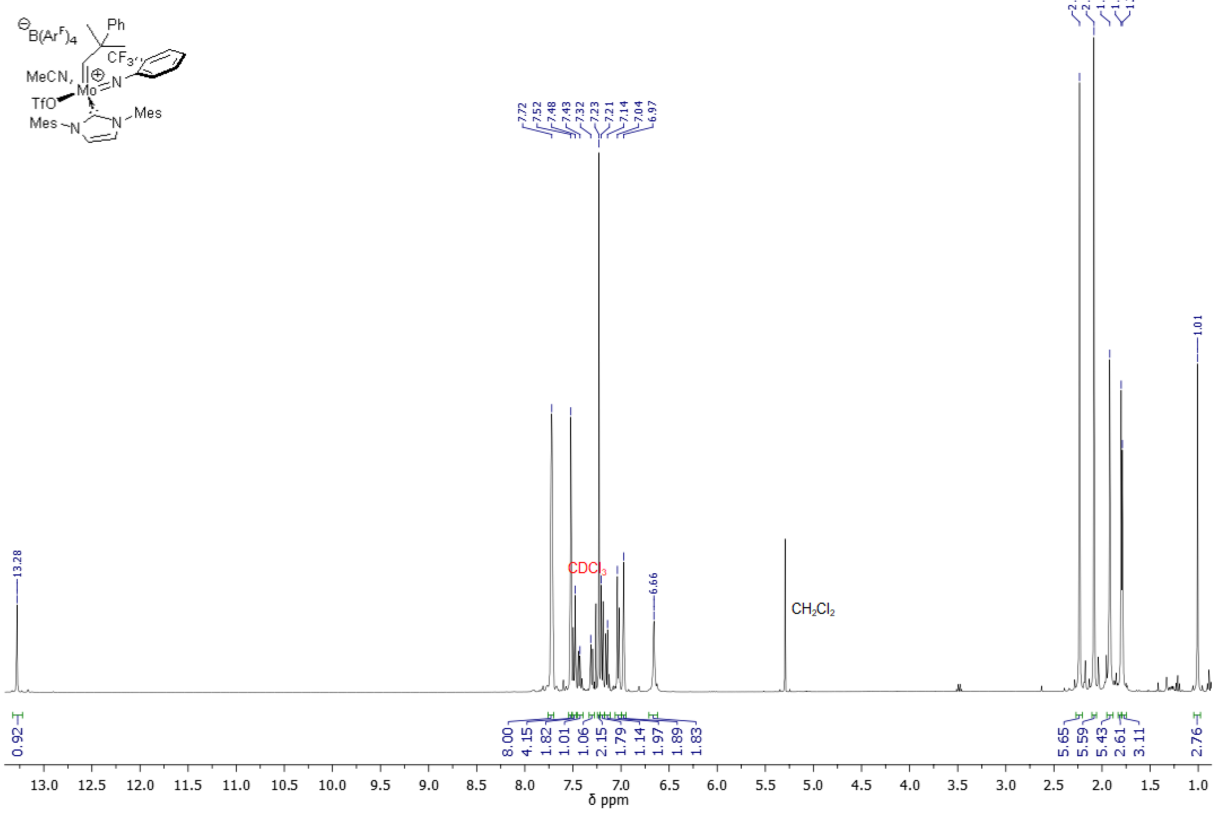

Figure S24. ^{1}H NMR (400 MHz, 25 °C, $CDCl_3$) of **4c**.

^{13}C NMR (101 MHz, $CDCl_3$) δ = 328.0, 182.2, 161.6, 149.4, 143.0, 141.5, 135.3, 134.9, 134.5, 134.2, 132.3, 131.0, 130.6, 129.8, 129.5, 128.8, 127.3, 126.2, 125.7, 124.7, 122.8, 117.6, 58.0, 29.1, 28.7, 20.9, 18.1, 18.0, 2.3.

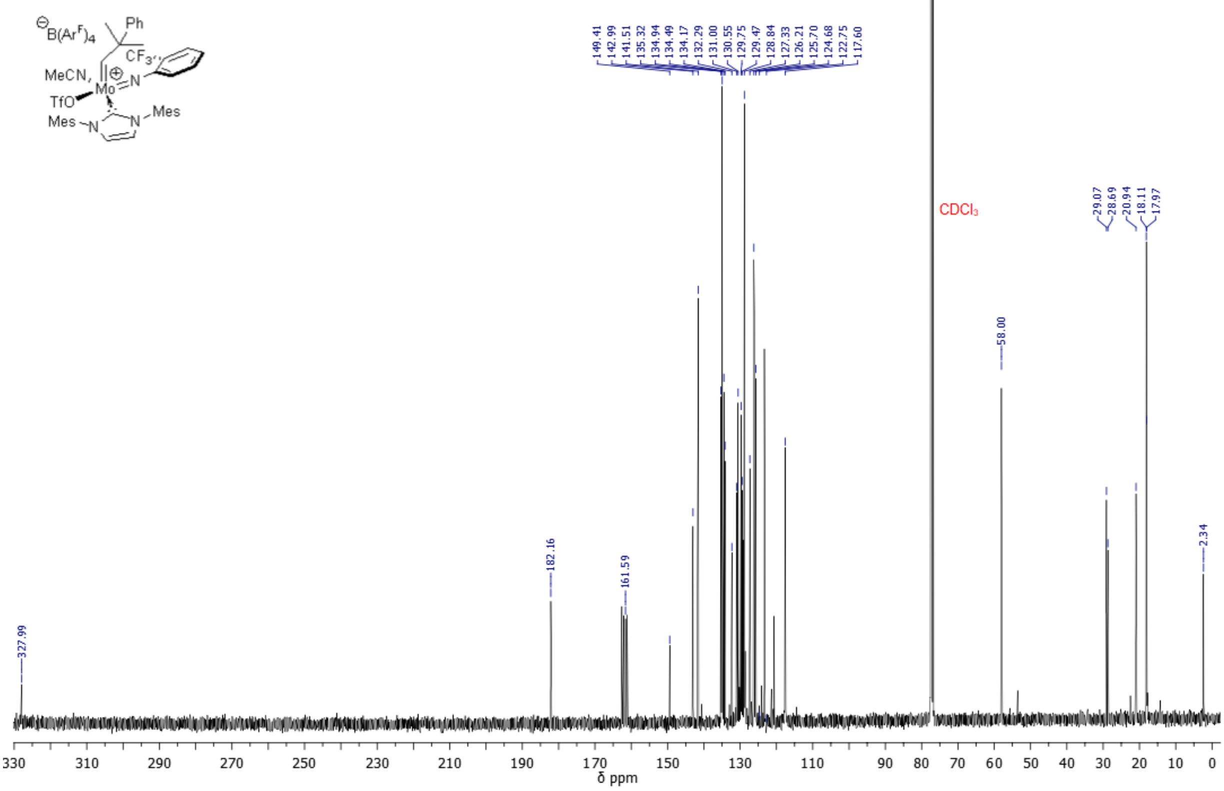

Figure S25. ^{13}C NMR (100 MHz, 25 °C, $CDCl_3$) of **4c**.

^{19}F NMR (376 MHz, $CDCl_3$) δ = -59.24, -62.41, -75.62.

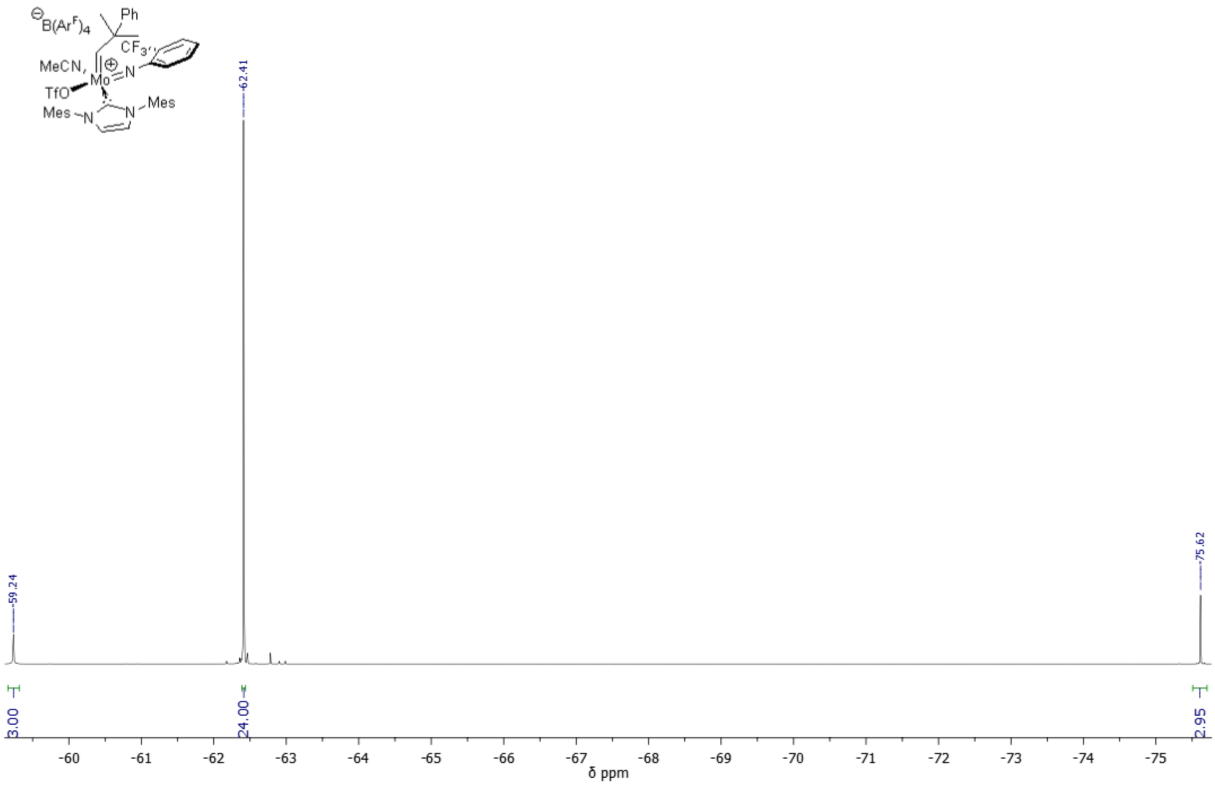

Figure S26. ^{19}F NMR (375 MHz, 25 °C, $CDCl_3$) of **4c**.

^{1}H NMR (400 MHz, CD_2Cl_2) δ = 7.11, 6.92, 4.01, 3.99.

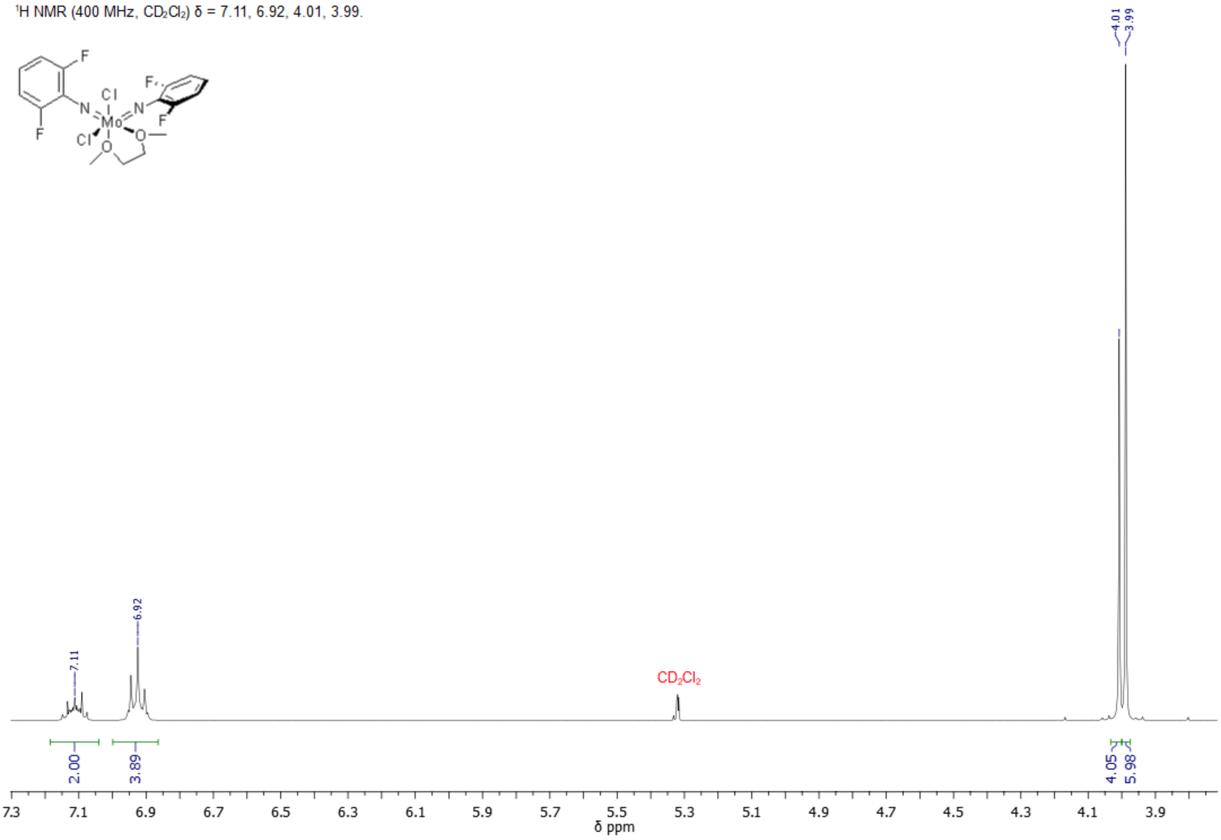

Figure S27. ^{1}H NMR (400 MHz, 25 °C, CD_2Cl_2) of **4d-p1**.

^{13}C NMR (101 MHz, CD_2Cl_2) δ = 159.6, 157.0, 135.5, 129.2, 112.0, 72.0, 64.7.

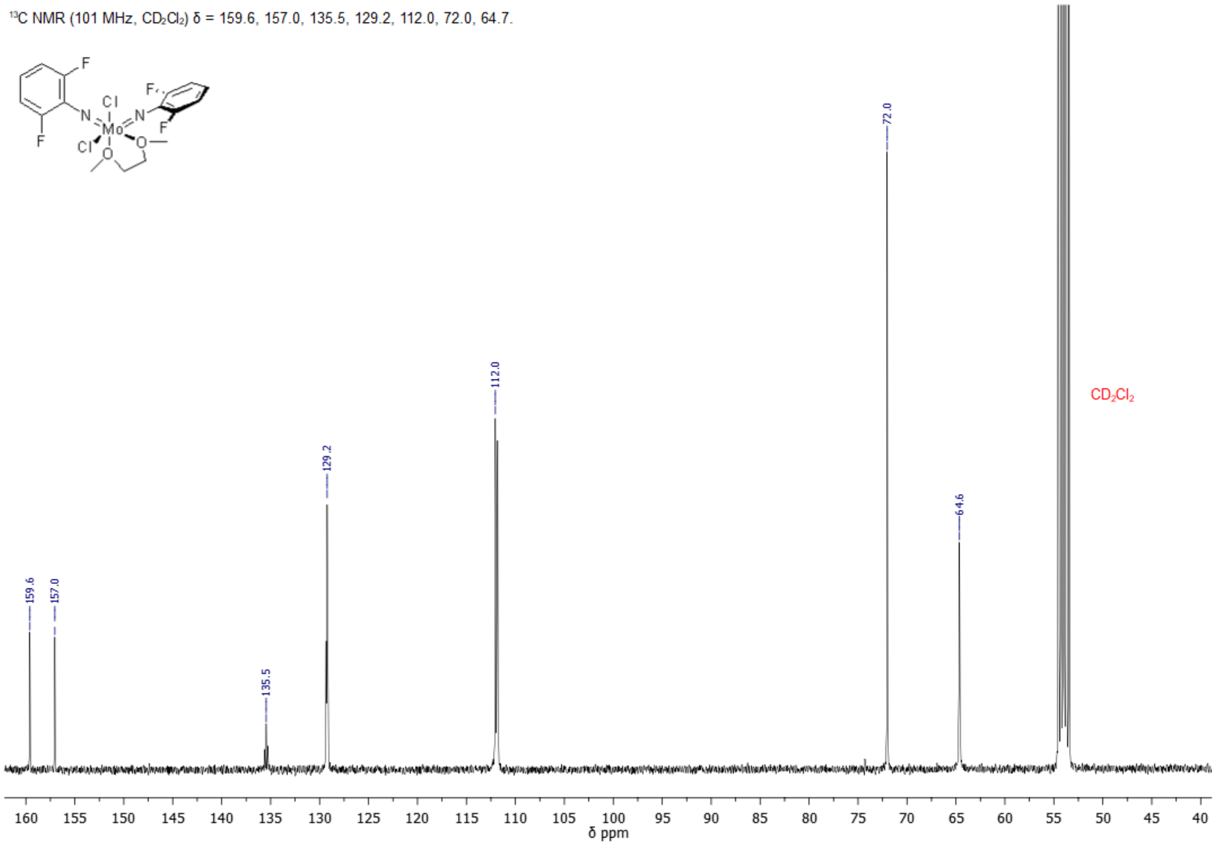

Figure S28. ^{13}C NMR (100 MHz, 25 °C, CD_2Cl_2) of **4d-p1**.

^{19}F NMR (376 MHz, CD_2Cl_2) δ = -117.37.

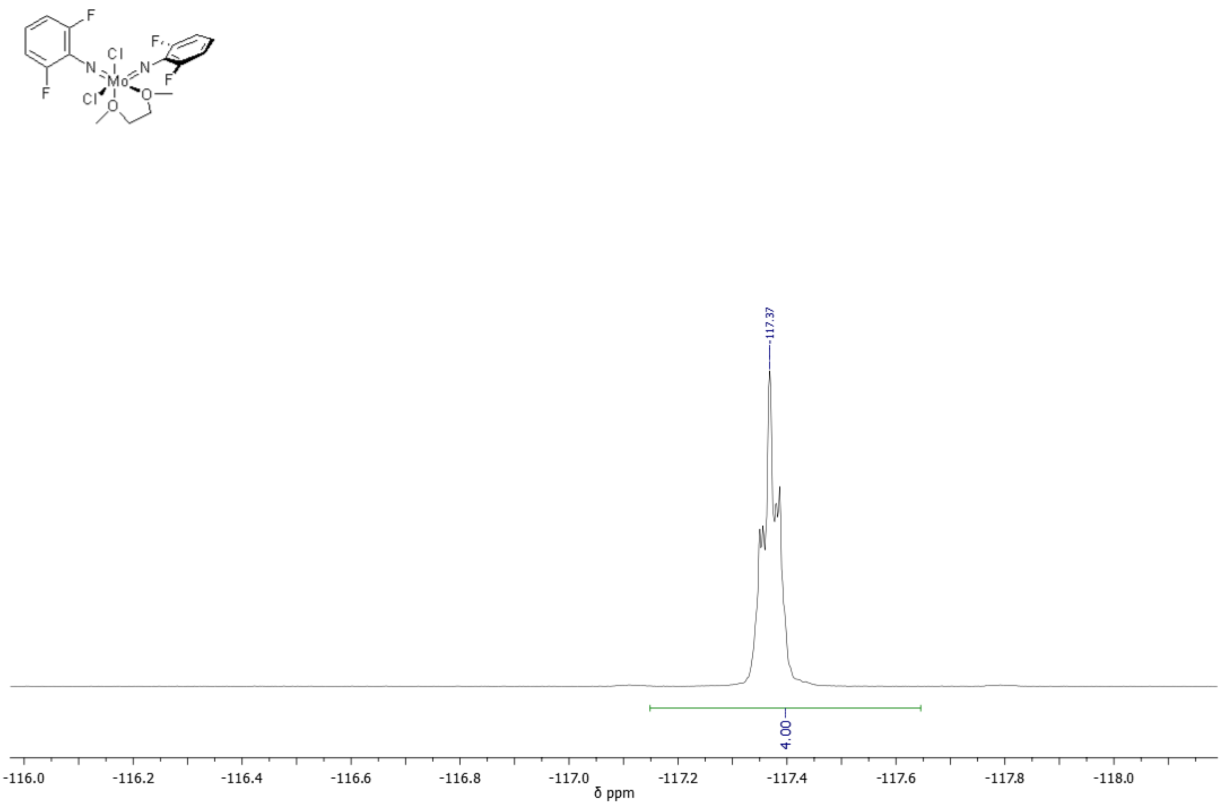

Figure S29. ^{19}F NMR (375 MHz, 25 °C, CD_2Cl_2) of **4d-p1**.

^{1}H NMR (400 MHz, CD_2Cl_2) δ = 7.37, 7.22, 7.08, 6.97, 6.82, 1.97, 1.40.

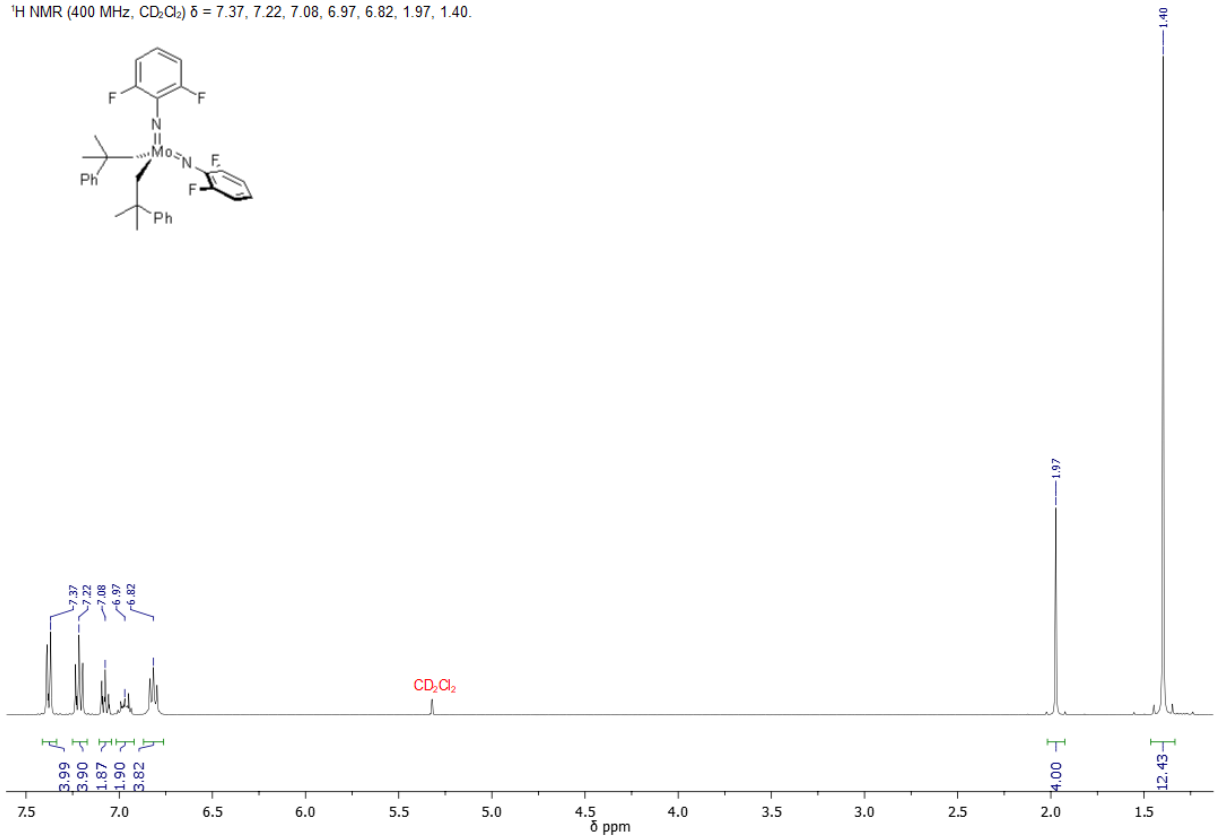

Figure S30. ^{1}H NMR (400 MHz, 25 °C, CD_2Cl_2) of **4d-p2**.

^{13}C NMR (101 MHz, CD_2Cl_2) δ = 157.8, 155.4, 151.2, 135.2, 128.7, 126.3, 126.3, 125.1, 111.4, 83.4, 41.2, 32.2.

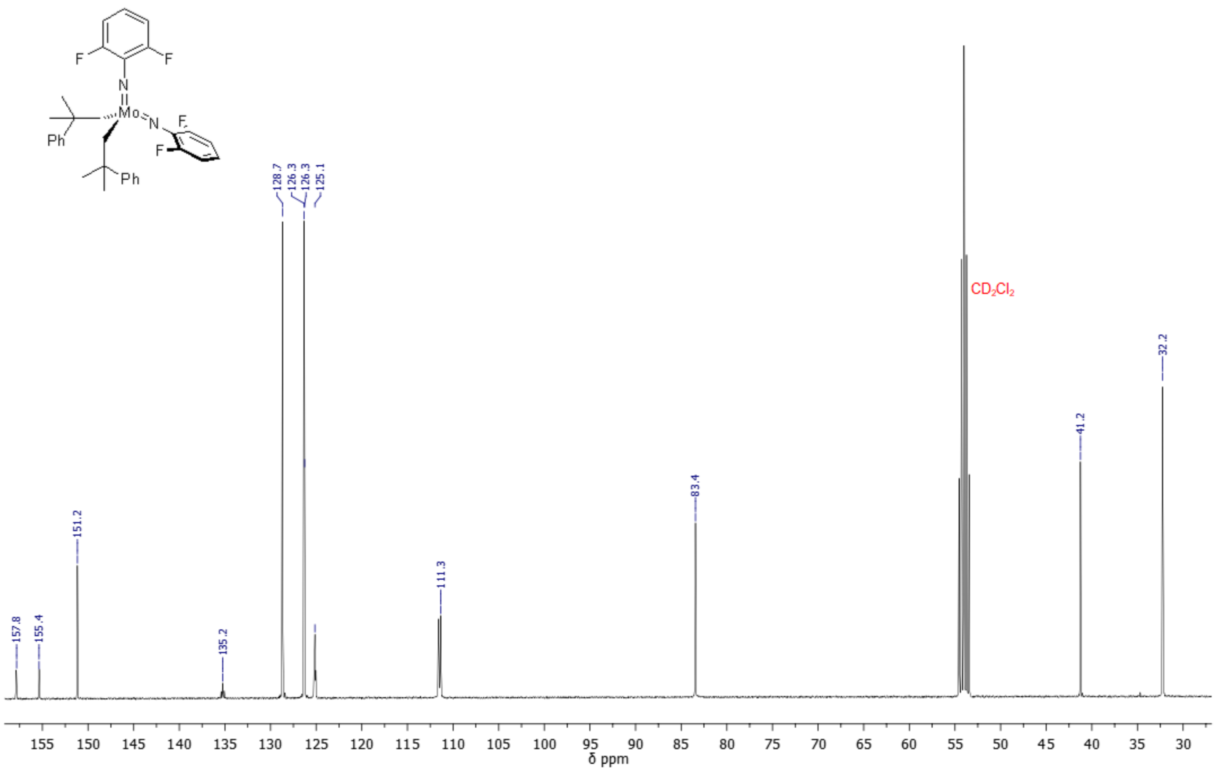

Figure S31. ^{13}C NMR (100 MHz, 25 °C, CD_2Cl_2) of **4d-p2**.

^{19}F NMR (376 MHz, CD_2Cl_2) δ = -119.52.

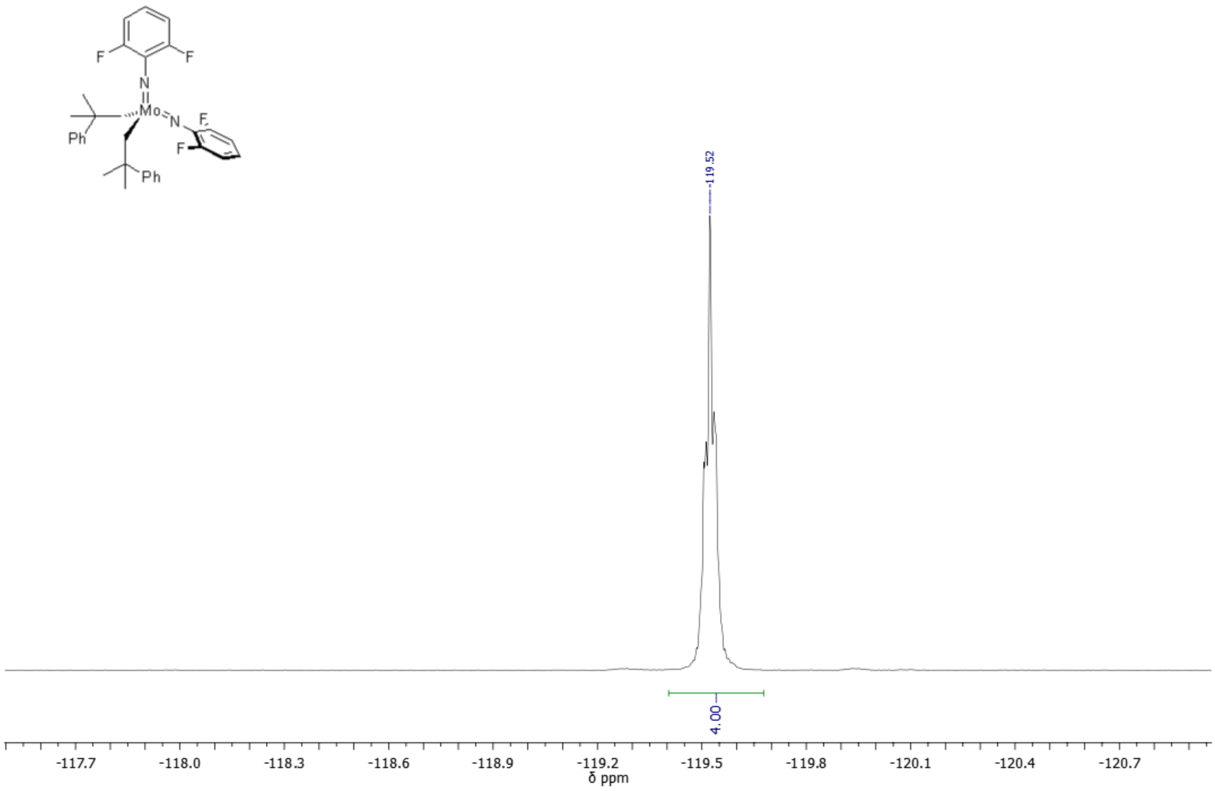

Figure S32. ^{19}F NMR (375 MHz, 25 °C, CD_2Cl_2) of **4d-p2**.

^{1}H NMR (400 MHz, CD_2Cl_2) δ = 14.74, 13.89, 7.47, 7.30, 7.19, 7.08, 6.98, 6.87, 6.82, 4.19, 4.11, 4.00, 3.88, 3.67, 3.60, 3.56, 1.83, 1.62, 1.50.

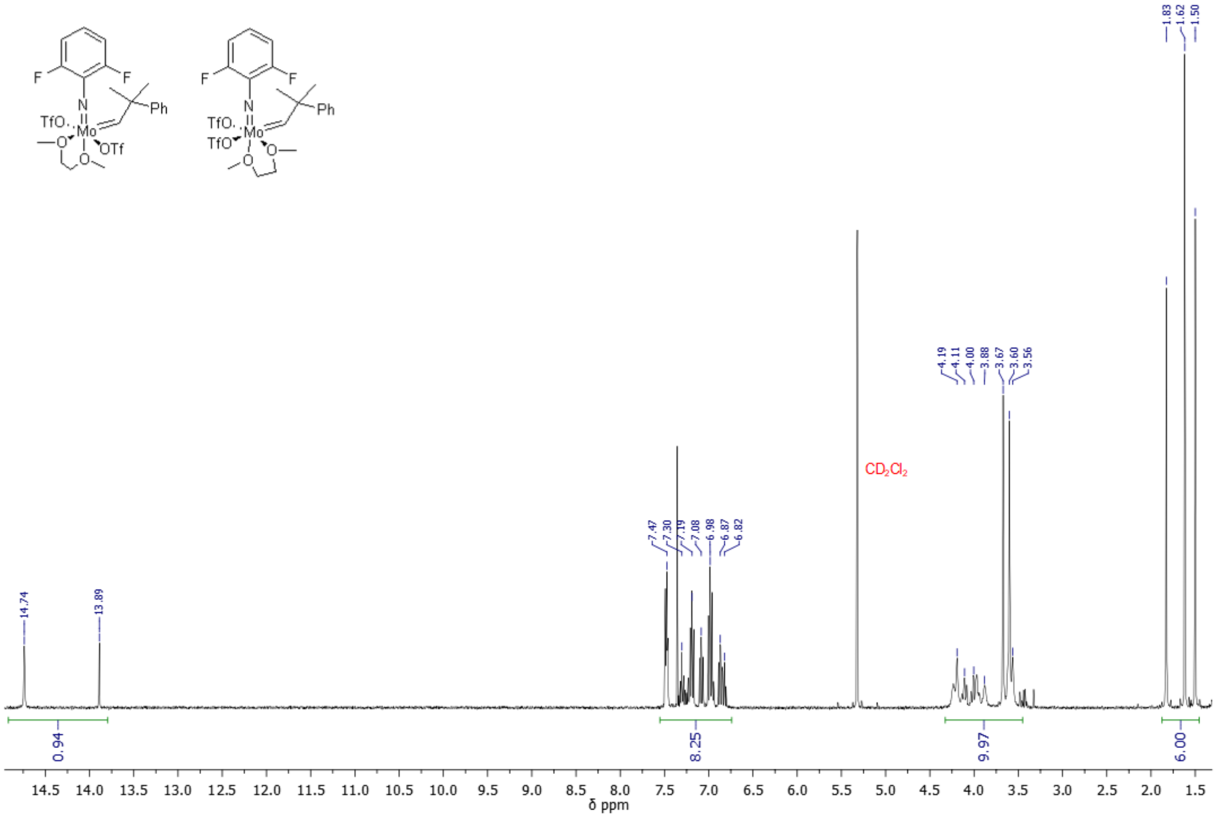

Figure S33. ^{1}H NMR (400 MHz, 25 °C, CD_2Cl_2) of **4d-p3**.

^{13}C NMR (101 MHz, CD_2Cl_2) δ = 335.6, 327.1, 162.4, 162.1, 159.8, 159.6, 148.5, 147.0, 133.3, 131.3, 131.0, 129.0, 128.5, 127.7, 127.3, 126.9, 120.0, 119.8, 112.7, 112.4, 79.0, 77.1, 75.0, 70.9, 65.7, 63.2, 61.7, 58.8, 58.8, 29.9, 29.6, 29.2.

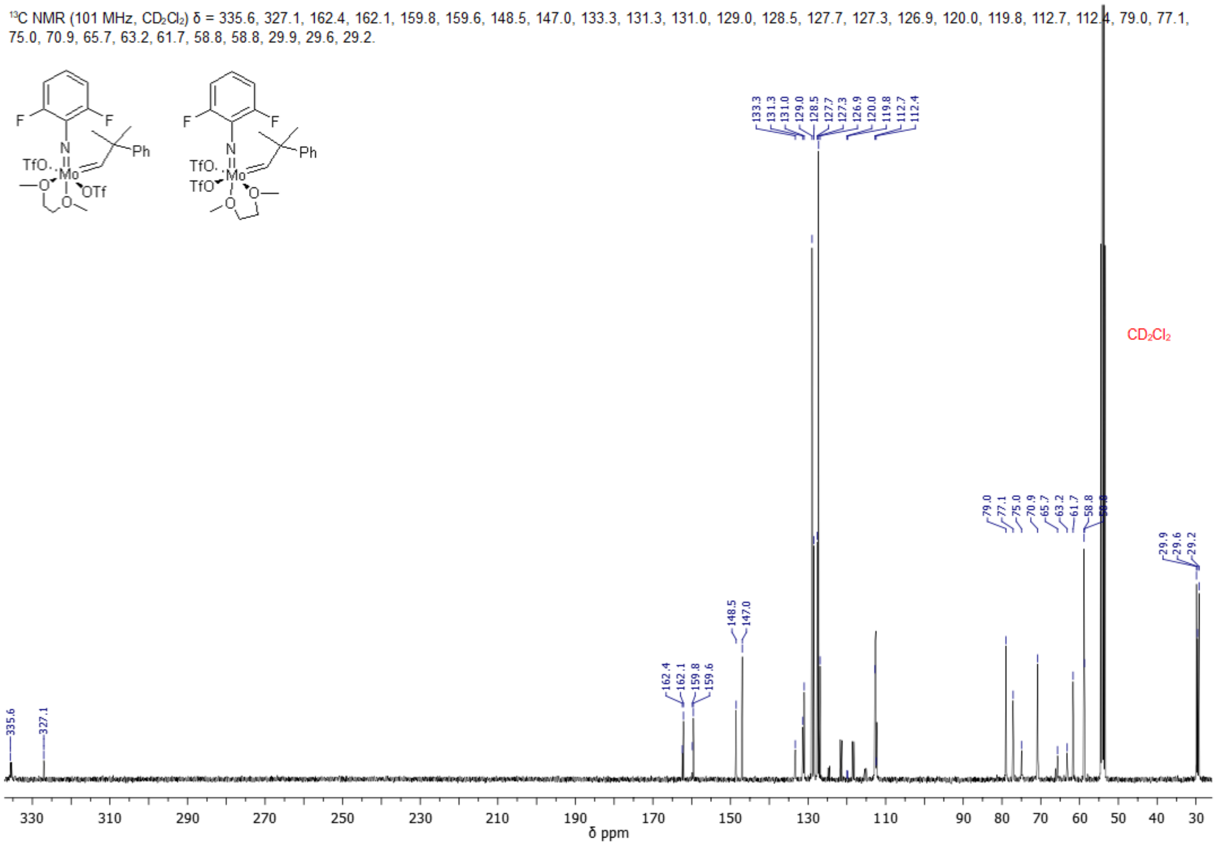

Figure S34. ^{13}C NMR (100 MHz, 25 °C, CD_2Cl_2) of **4d-p3**.

^{19}F NMR (376 MHz, CD_2Cl_2) δ = -77.31, -77.35, -78.24, -111.85, -114.36.

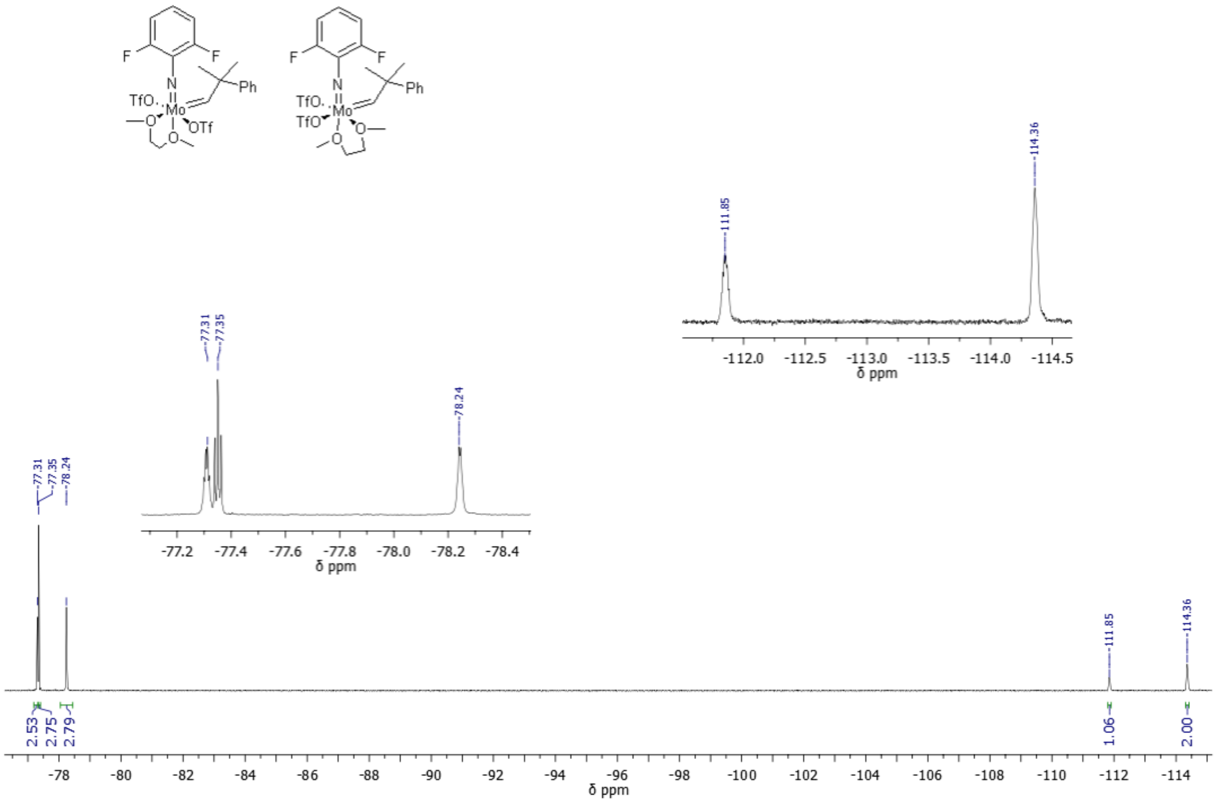

Figure S35. ^{19}F NMR (375 MHz, 25 °C, CD_2Cl_2) of **4d-p3**.

^{1}H NMR (400 MHz, CD_2Cl_2) δ = 13.32, 7.26, 7.22, 7.21, 7.09, 6.83, 6.73, 6.63, 2.15, 2.03, 2.00, 1.71, 1.69.

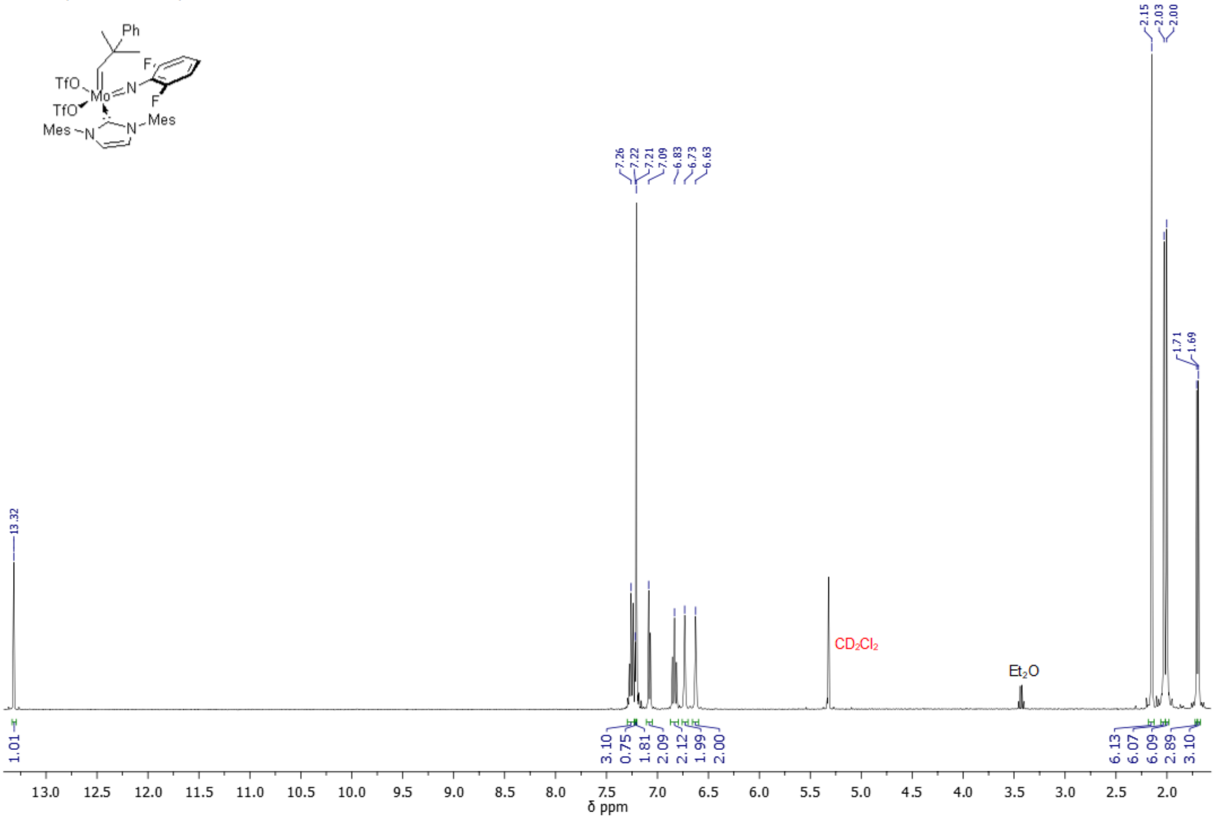

Figure S36. ^{1}H NMR (400 MHz, 25 °C, CD_2Cl_2) of **4d-p4**.

^{13}C NMR (101 MHz, CD_2Cl_2) δ = 320.4, 184.0, 160.2, 148.1, 141.0, 136.0, 135.3, 135.1, 134.0, 131.2, 130.4, 129.7, 128.8, 127.0, 126.0, 126.0, 119.9, 111.8, 56.5, 35.1, 27.9, 21.4, 18.5.

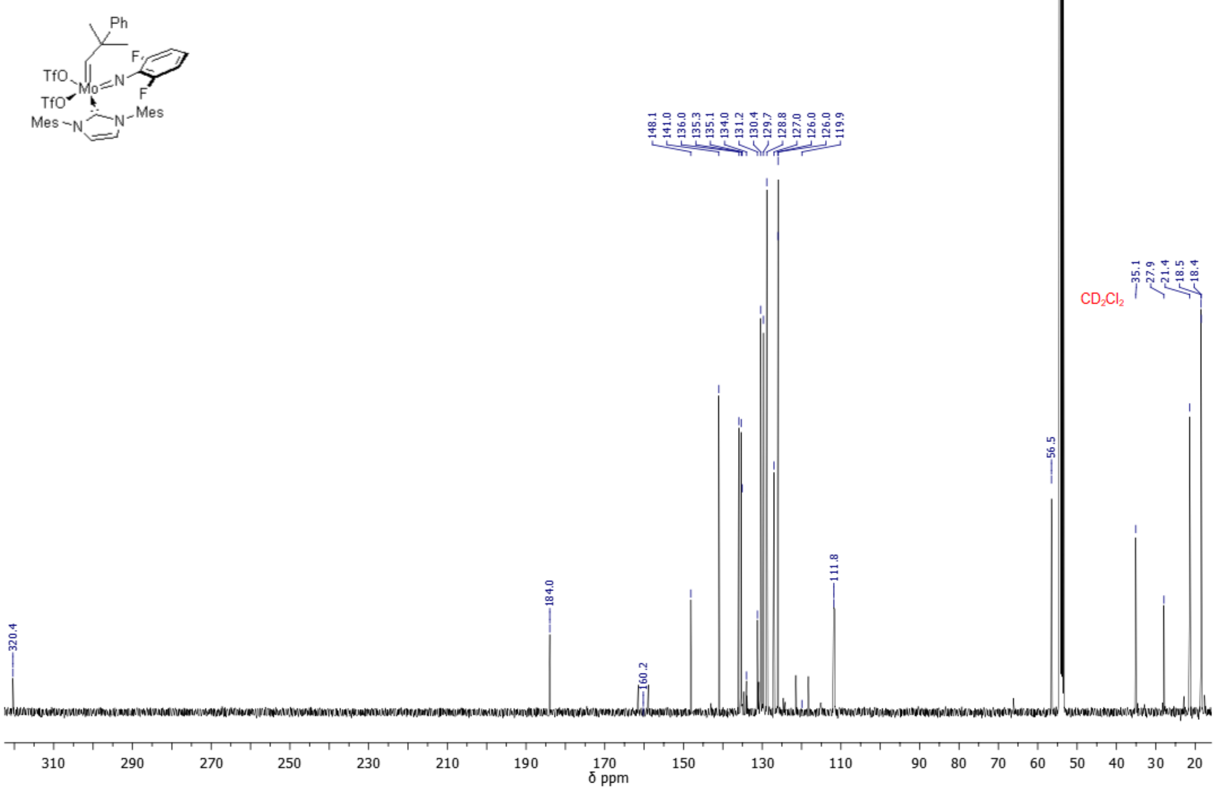

Figure S37. ^{13}C NMR (100 MHz, 25 °C, CD_2Cl_2) of **4d-p4**.

^{19}F NMR (376 MHz, CD_2Cl_2) δ = -75.77, -77.02, -109.53.

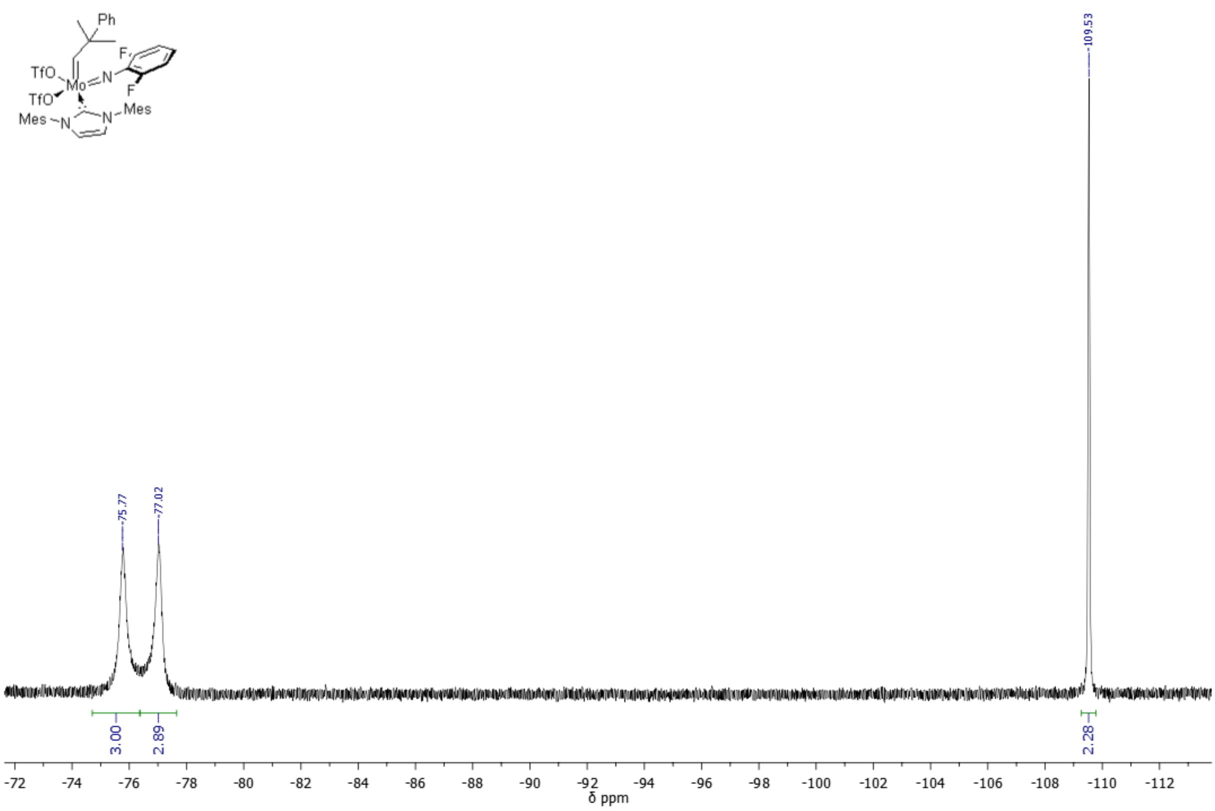

Figure S38. ^{19}F NMR (375 MHz, 25 °C, CD_2Cl_2) of **4d-p4**.

^{1}H NMR (400 MHz, CD_2Cl_2) δ = 13.26, 7.73, 7.56, 7.33, 7.30, 7.22, 7.15, 7.07, 6.95, 6.88, 6.78, 2.22, 2.05, 1.87, 1.78, 1.10.

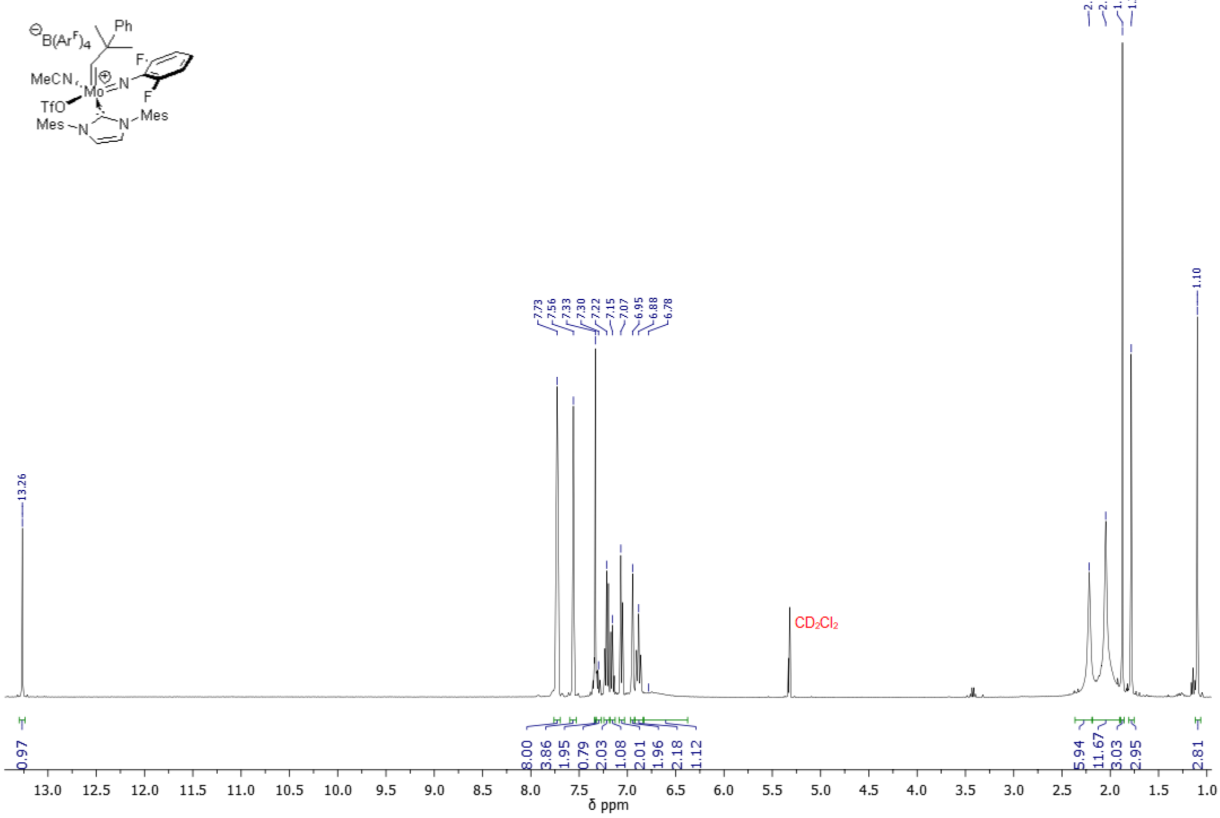

Figure S39. ^{1}H NMR (400 MHz, 25 °C, CD_2Cl_2) of **4d**.

^{13}C NMR (101 MHz, CD_2Cl_2) δ = 328.6, 182.8, 162.1, 144.2, 141.5, 135.9, 135.2, 134.8, 134.4, 132.7, 132.5, 130.6, 130.1, 129.8, 129.2, 129.0, 127.5, 126.4, 125.8, 125.0, 119.5, 117.9, 112.3, 56.5, 28.5, 27.8, 21.3, 18.1, 3.0.

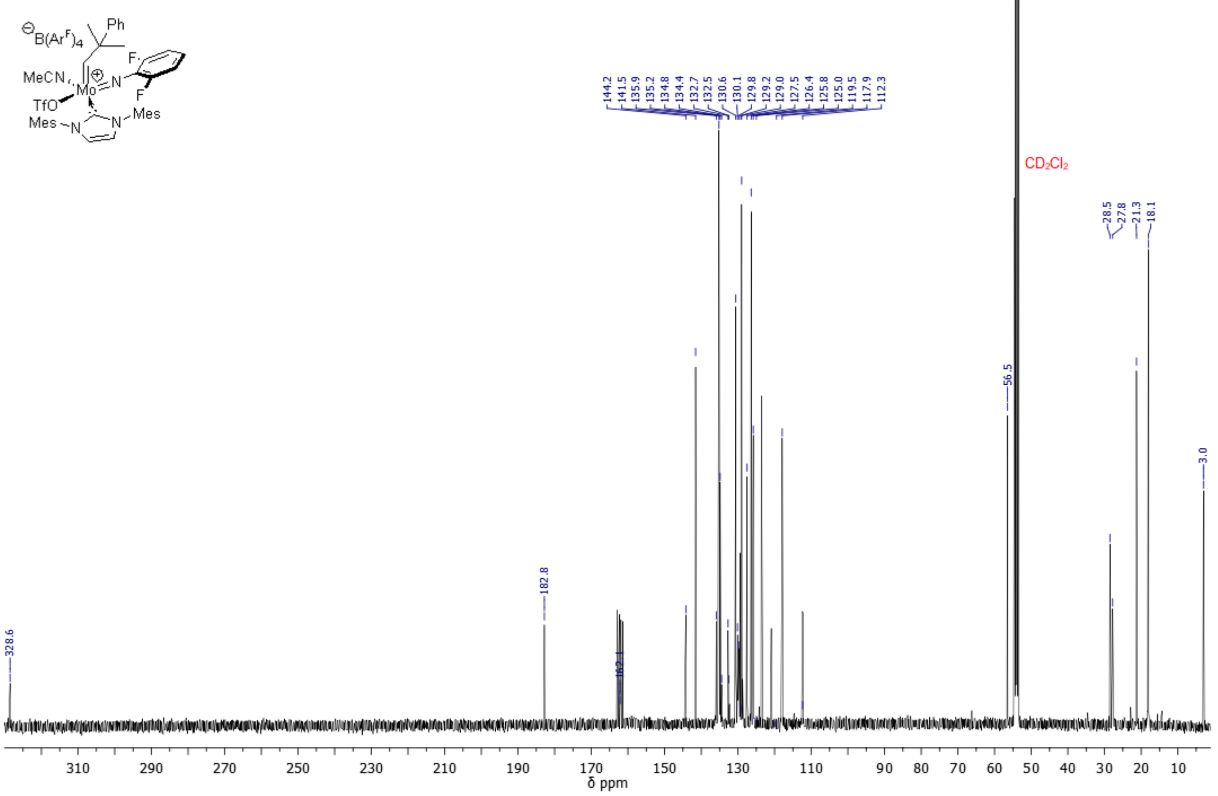

Figure S40. ^{13}C NMR (100 MHz, 25 °C, CD_2Cl_2) of **4d**.

^{19}F NMR (376 MHz, CD_2Cl_2) δ = -62.76, -76.28, -111.14.

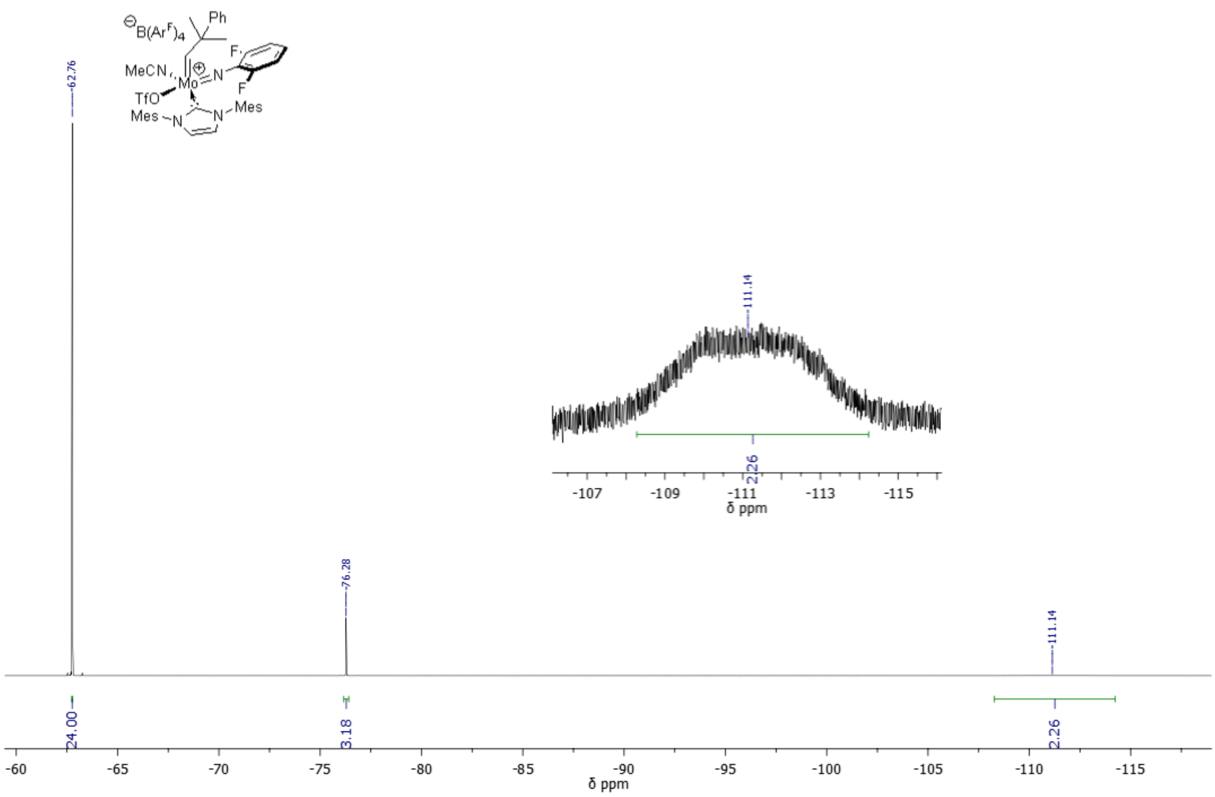

Figure S41. ^{19}F NMR (375 MHz, 25 °C, CD_2Cl_2) of **4d**.

^1H NMR (400 MHz, $CDCl_3$) δ = 13.03, 7.70, 7.51, 7.34, 7.07, 7.00, 6.87, 6.80, 2.31, 2.08, 2.02, 1.85, 1.10, 0.98.

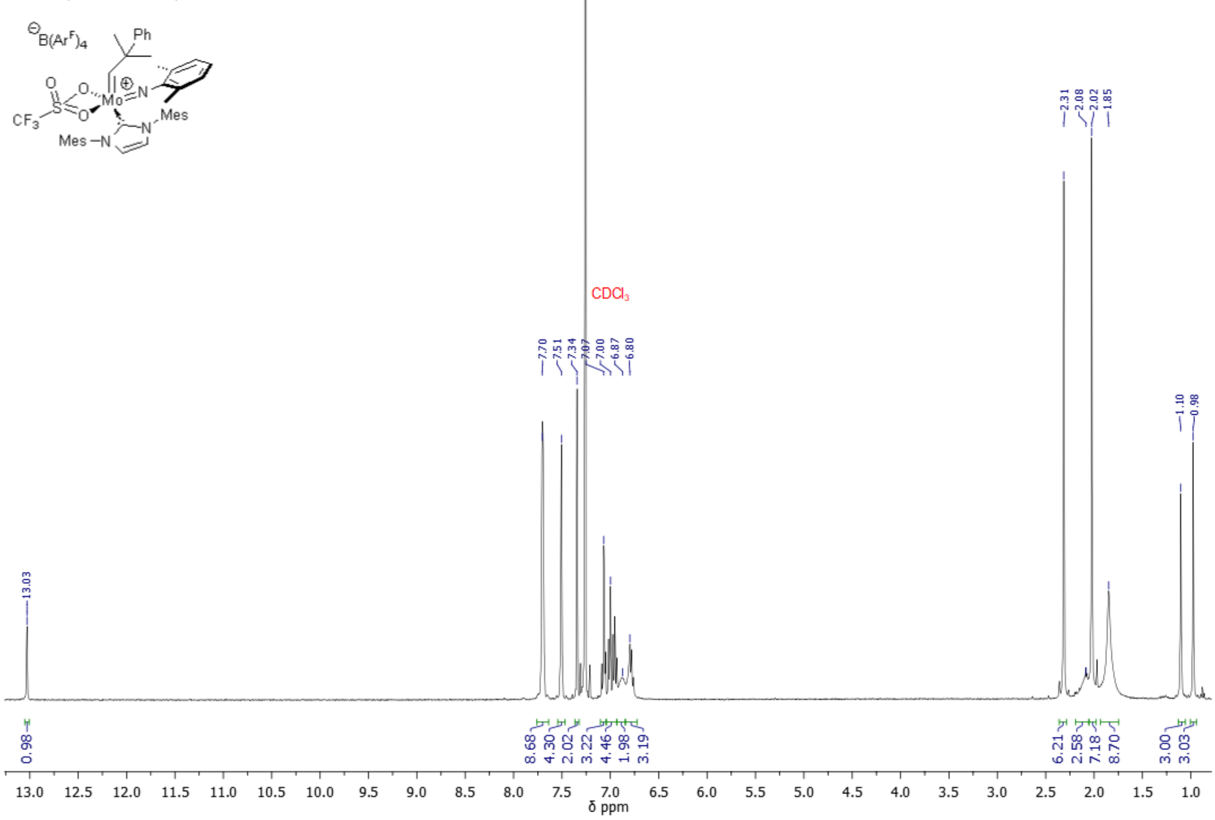

Figure S42. ^1H NMR (400 MHz, 25 °C, $CDCl_3$) **5a**.

^{19}F NMR (376 MHz, $CDCl_3$) δ = -62.42, -73.22.

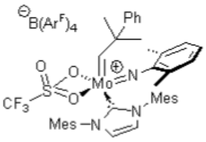

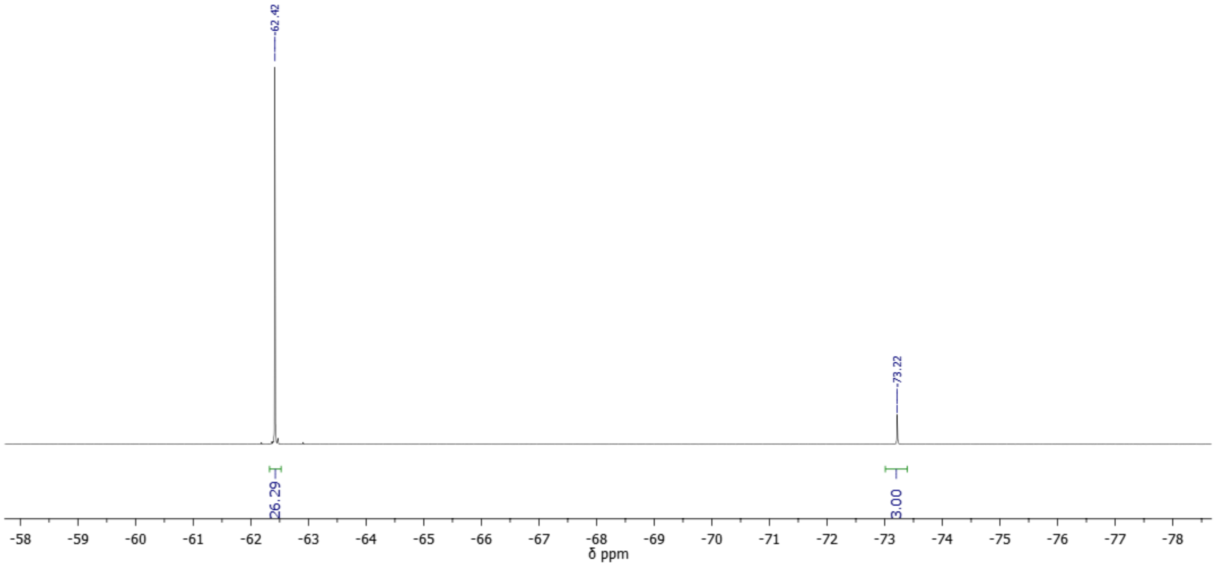

Figure S43. ^{19}F NMR (375 MHz, 25 °C, $CDCl_3$) of **5a**.

^{13}C NMR (101 MHz, $CDCl_3$) δ = 321.5, 179.9, 161.9, 154.3, 145.5, 142.4, 135.4, 135.3, 135.0, 132.7, 131.1, 130.8, 130.1, 129.1, 128.3, 127.2, 126.3, 126.2, 124.7, 119.0, 117.6, 58.1, 30.5, 26.2, 21.1, 19.5, 17.9, 17.4.

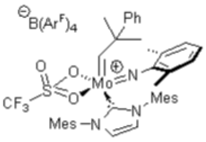

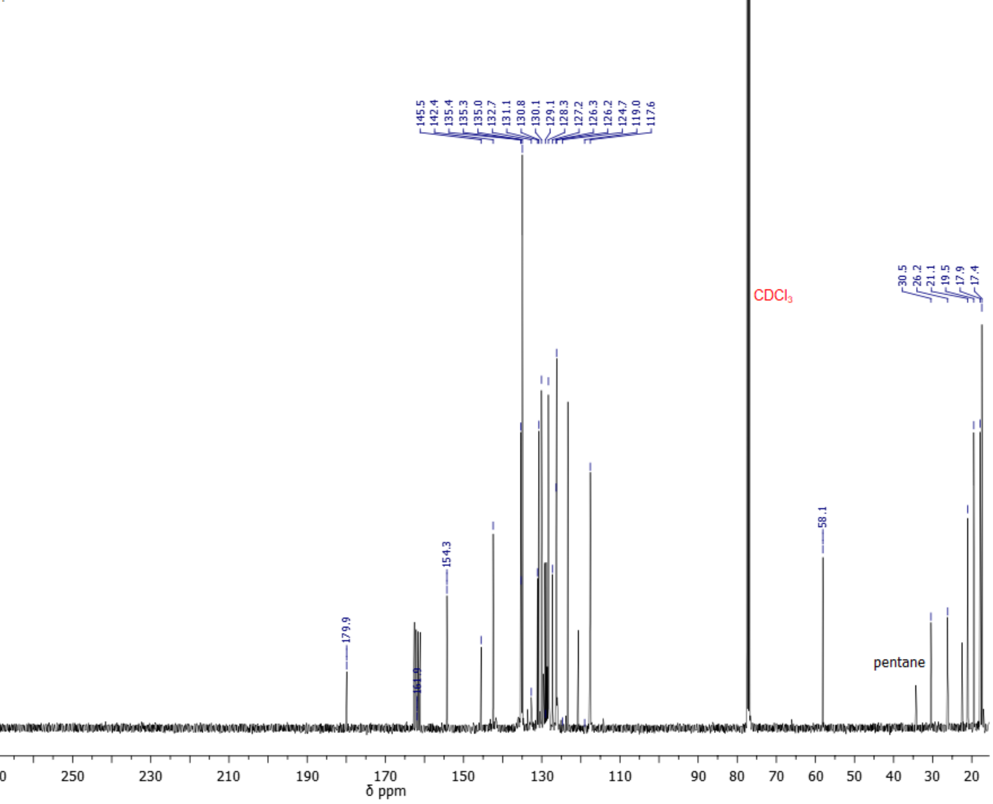

Figure S44. ^{13}C NMR (100 MHz, 25 °C, $CDCl_3$) of **5a**.

^{1}H NMR (400 MHz, CD_2Cl_2) δ = 12.69, 7.72, 7.56, 7.47, 7.12, 7.09, 7.00, 6.99, 6.96, 6.85, 2.33, 2.07, 1.91, 1.24, 0.95.

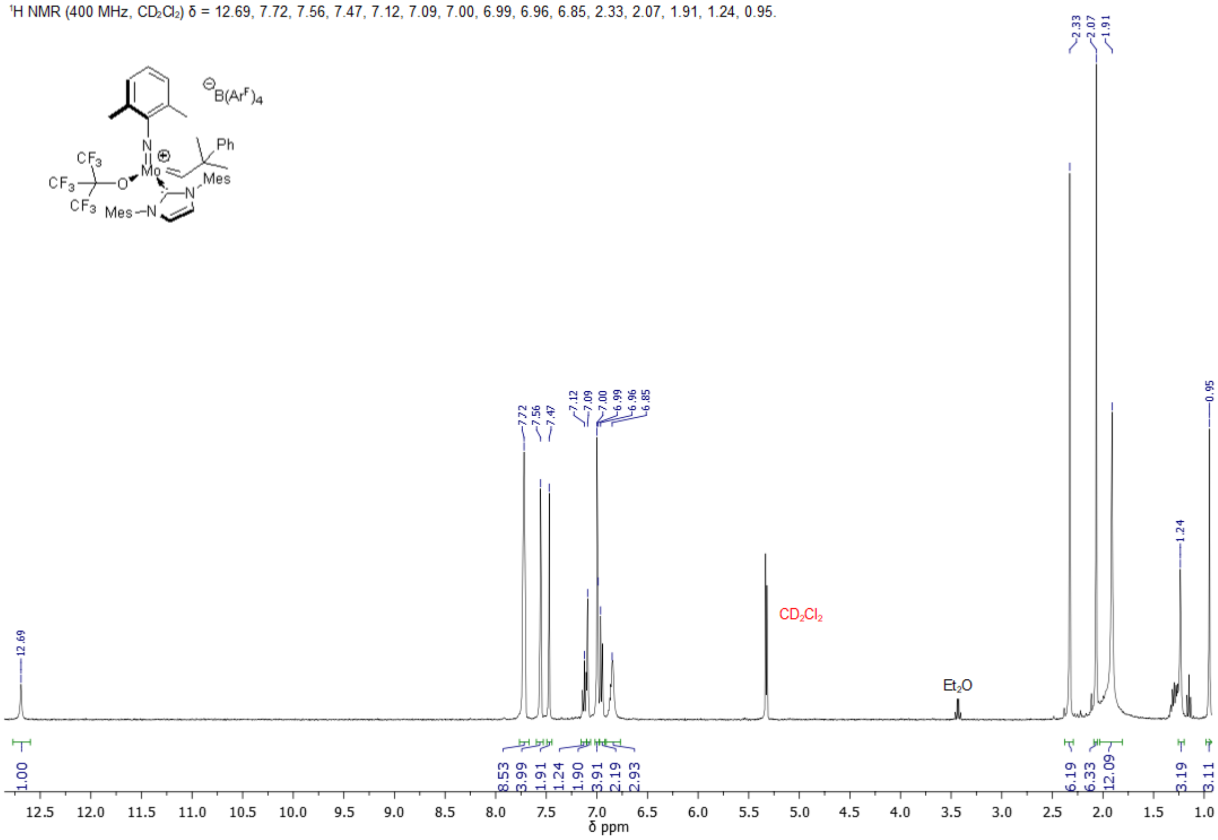

Figure S45. ^{1}H NMR (400 MHz, 25 °C, CD_2Cl_2) of **5b**.

^{19}F NMR (376 MHz, CD_2Cl_2) δ = -62.88, -73.34.

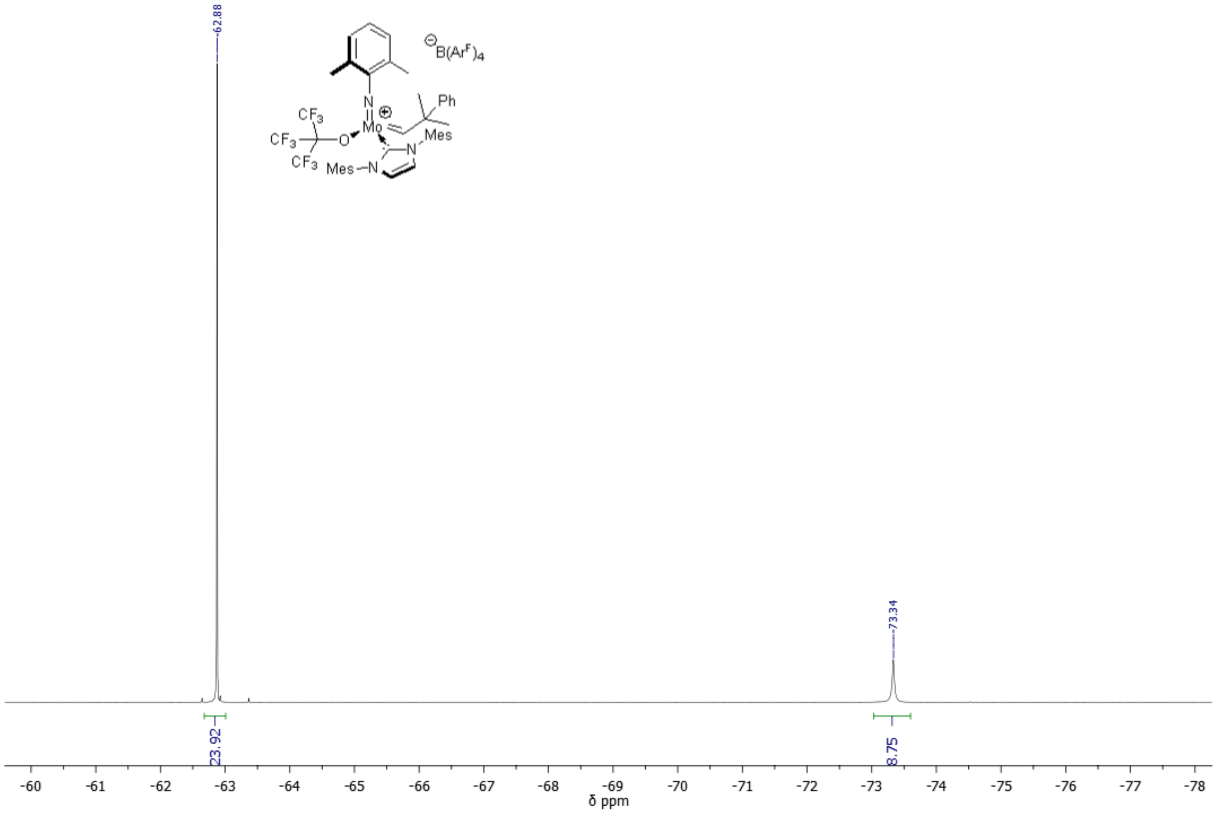

Figure S46. ^{19}F NMR (375 MHz, 25 °C, CD_2Cl_2) of **5b**.

^{13}C NMR (101 MHz, CD_2Cl_2) δ = 315.6, 180.1, 162.3, 156.1, 146.5, 142.5, 135.4, 134.2, 131.2, 131.1, 130.3, 129.3, 128.7, 128.6, 127.5, 127.2, 126.3, 125.1, 121.1, 118.0, 84.4, 57.4, 31.3, 27.9, 21.3, 19.7, 18.1.

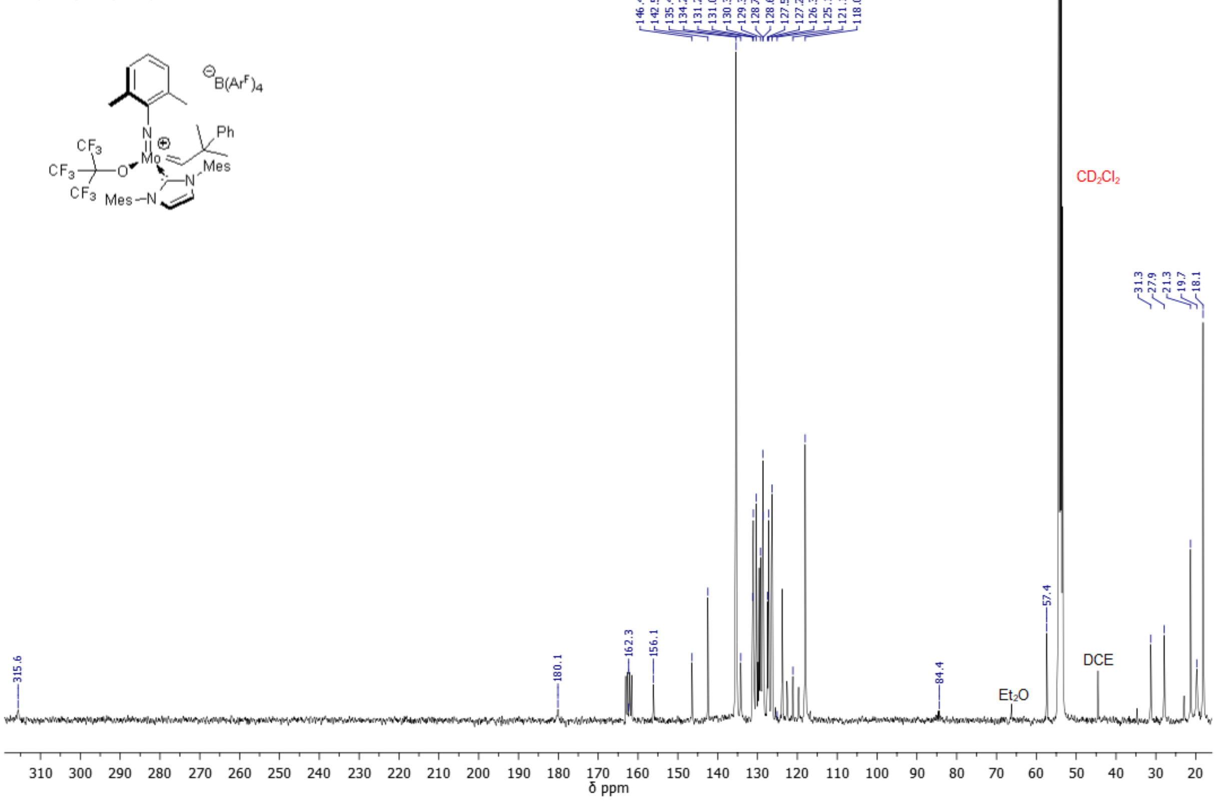

Figure S47. ^{13}C NMR (100 MHz, 25 °C, CD_2Cl_2) of **5b**.

^1H NMR (400 MHz, $CDCl_3$) δ = 13.38, 7.95, 7.70, 7.51, 7.16, 7.16, 6.99, 6.97, 6.67, 5.11, 4.21, 3.83, 3.67, 2.27, 1.98, 1.86.

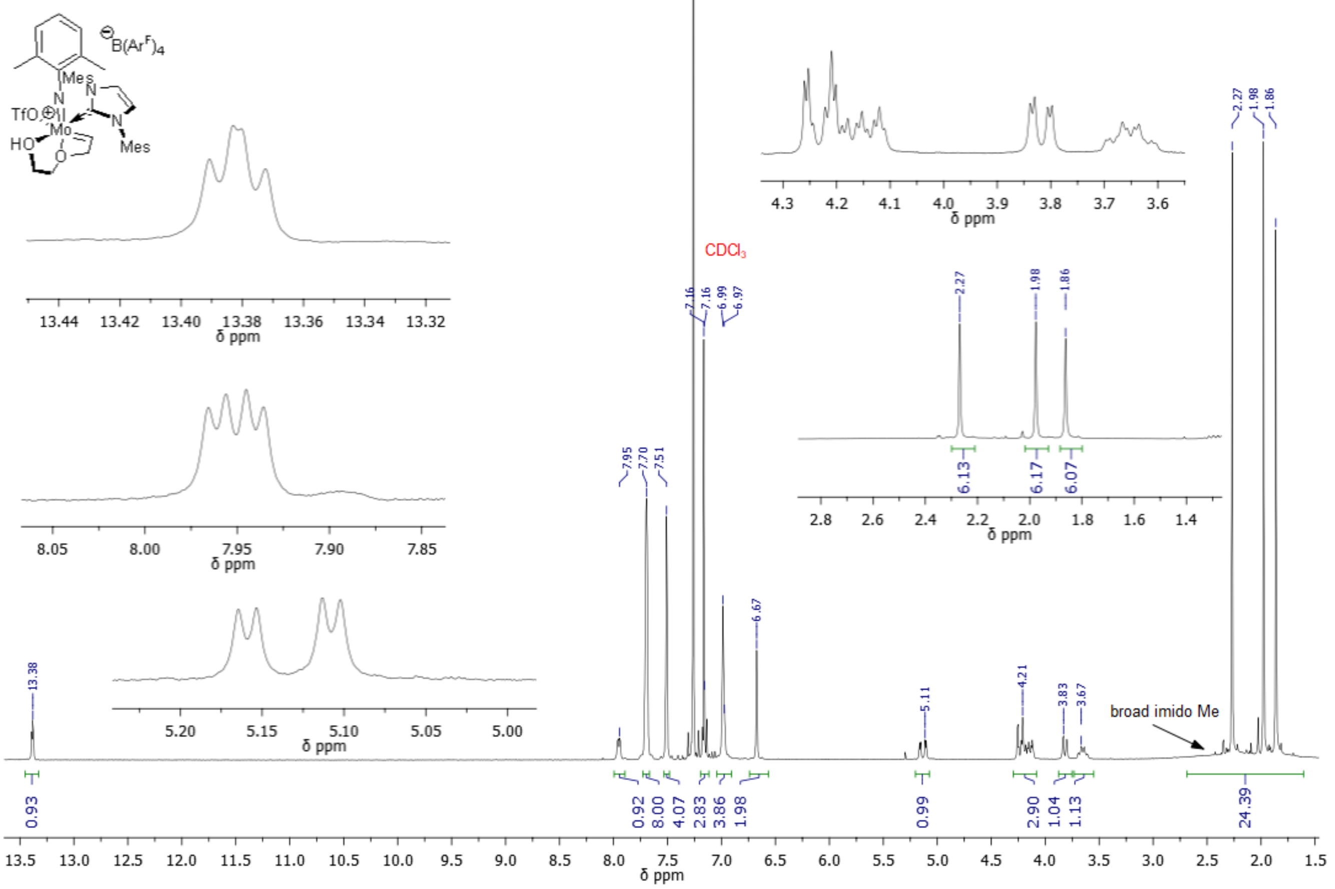

Figure S48. ^1H NMR (400 MHz, 25 °C, $CDCl_3$) of **6**.

^{19}F NMR (376 MHz, CDCl$_3$) δ = -62.39, -76.70.

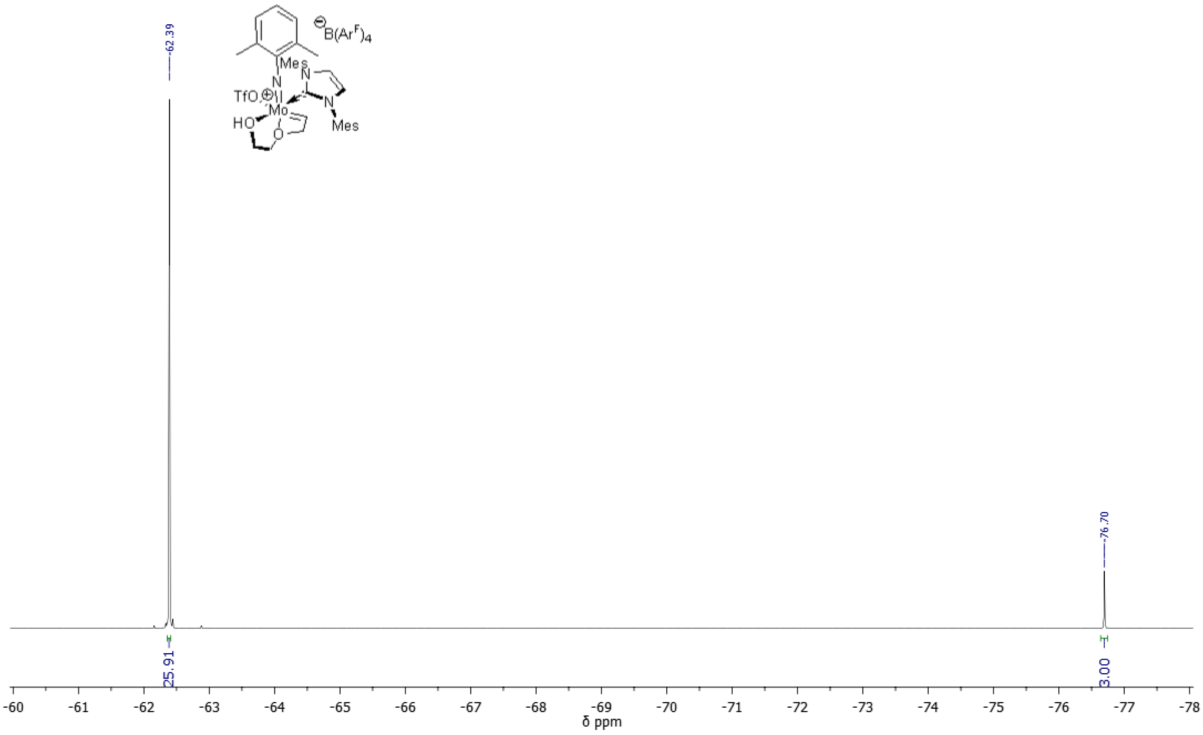

Figure S49. ^{19}F NMR (375 MHz, 25 °C, CDCl$_3$) of **6**.

^{13}C NMR (101 MHz, CDCl$_3$) δ = 309.5, 209.7, 180.4, 161.6, 155.2, 141.5, 135.7, 135.0, 131.3, 130.0, 129.9, 129.2, 128.9, 128.7, 126.0, 126.0, 12[illegible].3, 117.6, 82.3, 71.5, 63.6, 21.1, 19.5, 17.8, 17.5.

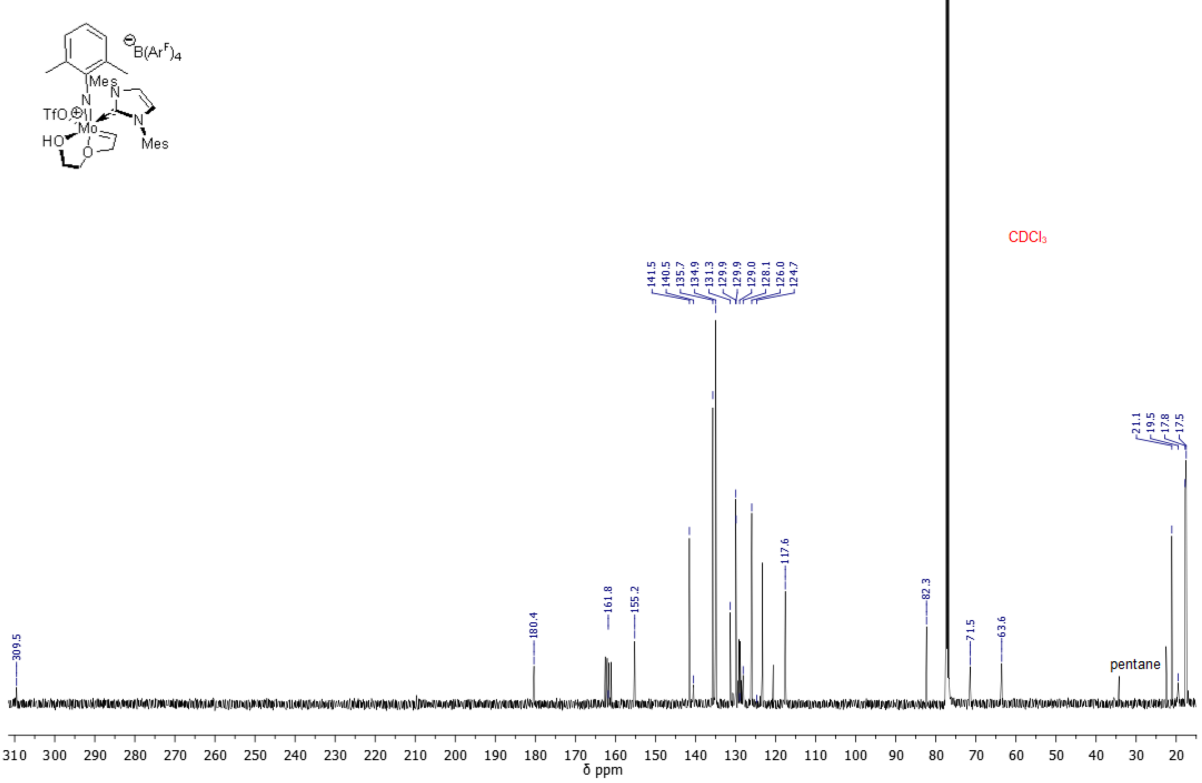

Figure S50. ^{13}C NMR (100 MHz, 25 °C, CDCl$_3$) of **6**.

^{1}H NMR (400 MHz, $CDCl_3$) δ = 13.59, 7.18, 7.06, 6.82, 2.35, 2.18, 1.89, 1.66, 1.64, 1.10.

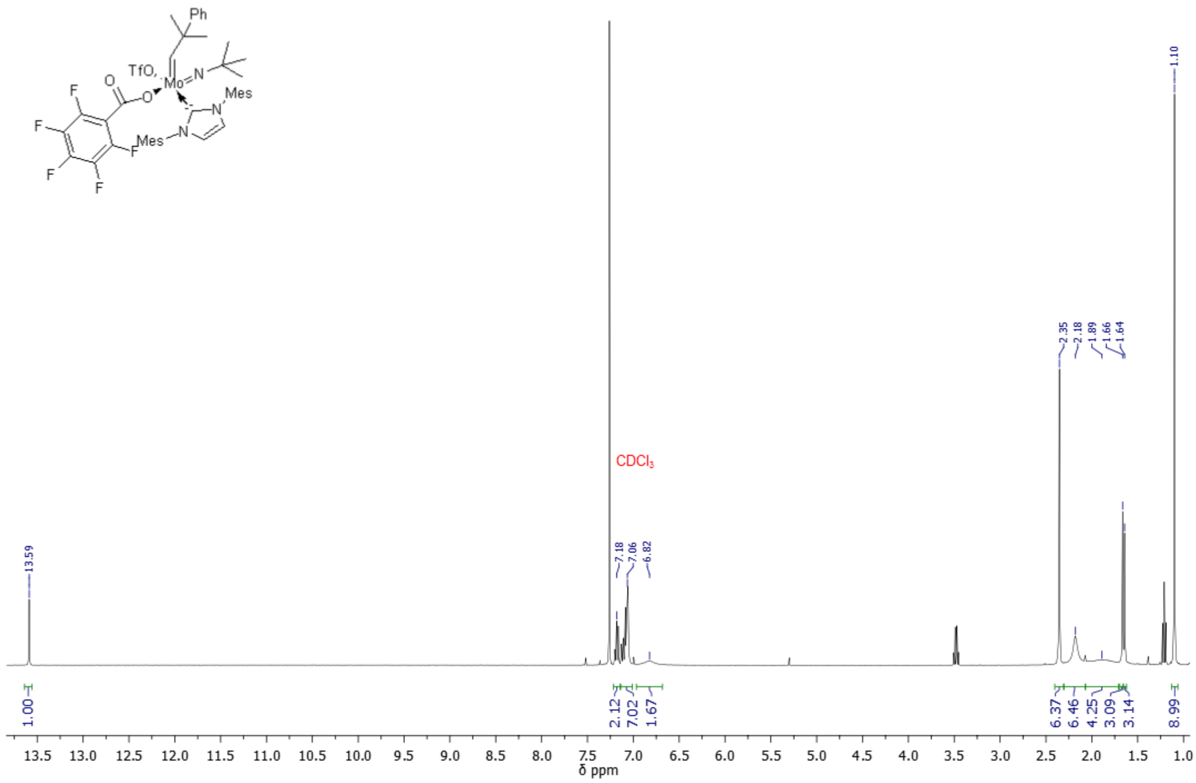

Figure S51. ^{1}H NMR (400 MHz, 25 °C, $CDCl_3$) of **10**.

^{13}C NMR (101 MHz, $CDCl_3$) δ = 317.2, 188.4, 174.4, 149.4, 140.0, 130.0, 129.5, 128.2, 126.1, 126.1, 125.0, 76.4, 53.00, 33.0, 29.7, 29.7, 21.2, 18.5, 18.3.

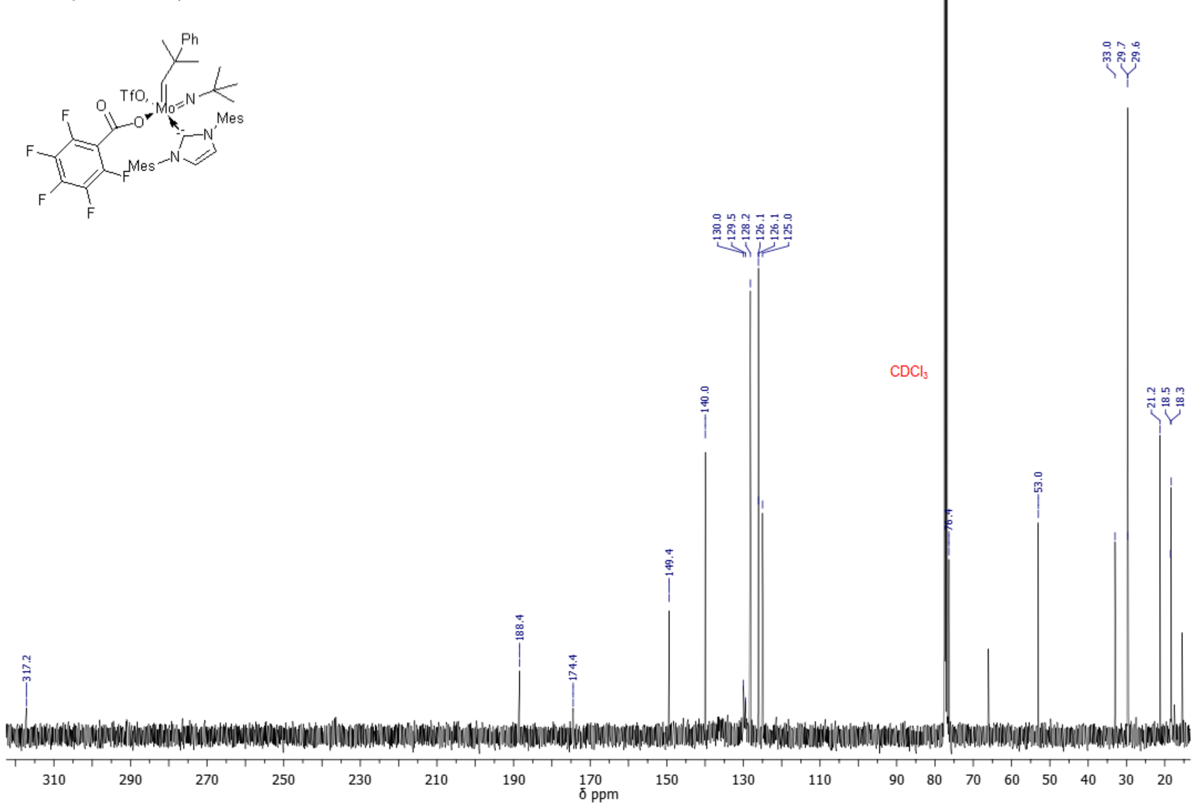

Figure S52. ^{13}C NMR (100 MHz, 25 °C, $CDCl_3$) of **10**.

^{19}F NMR (376 MHz, $CDCl_3$) δ = -77.38, -136.57, -149.92, -162.69.

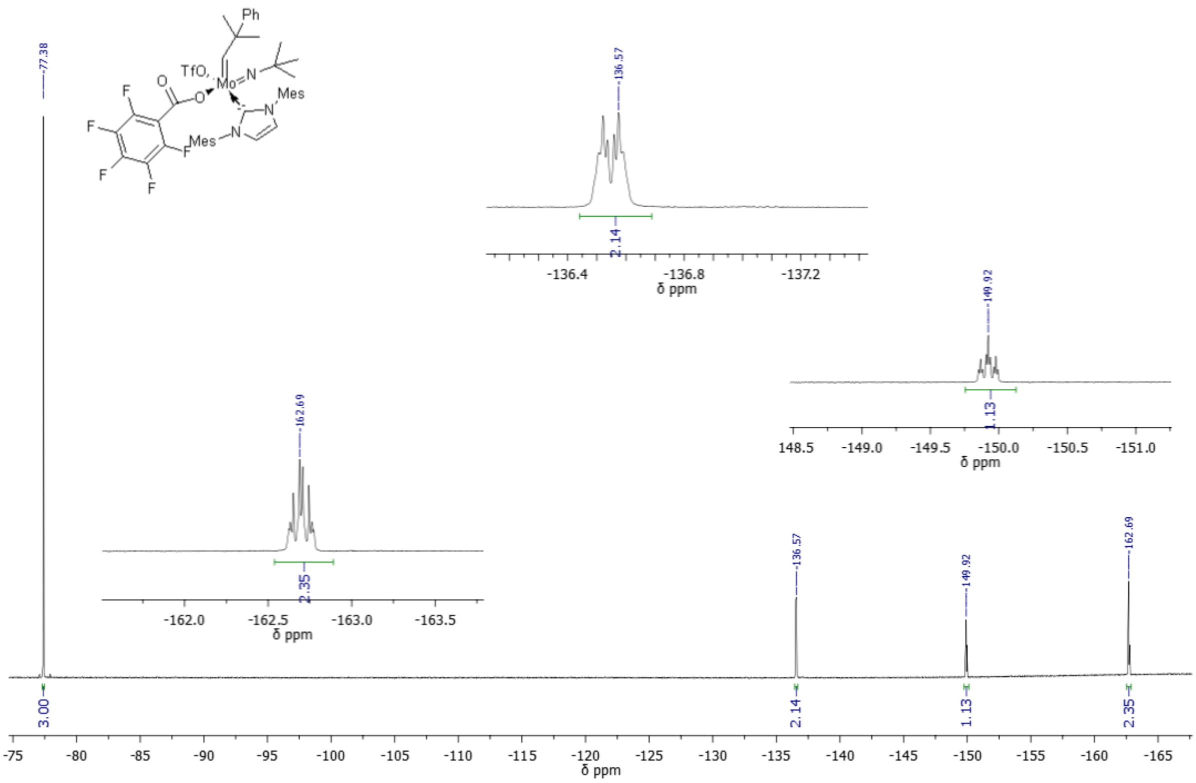

Figure S53. ^{19}F NMR (375 MHz, 25 °C, $CDCl_3$) of **10**.

^{1}H NMR (400 MHz, $CDCl_3$) δ = 13.06, 7.71, 7.51, 7.25, 7.12, 7.06, 6.86, 2.28, 2.11, 1.90, 1.45, 1.40, 0.87.

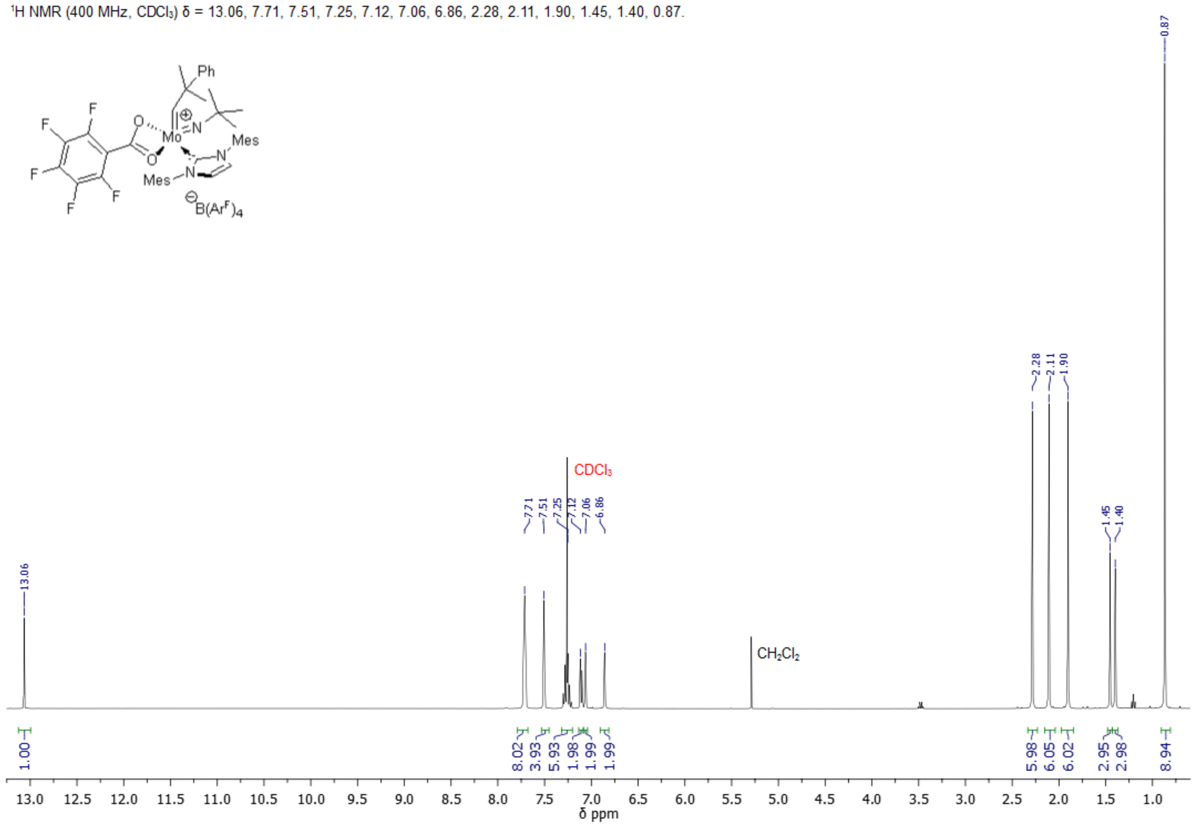

Figure S54. ^{1}H NMR (400 MHz, 25 °C, $CDCl_3$) of **11**.

^{19}F NMR (376 MHz, $CDCl_3$) δ = -62.46, -134.69, -141.37, -158.72.

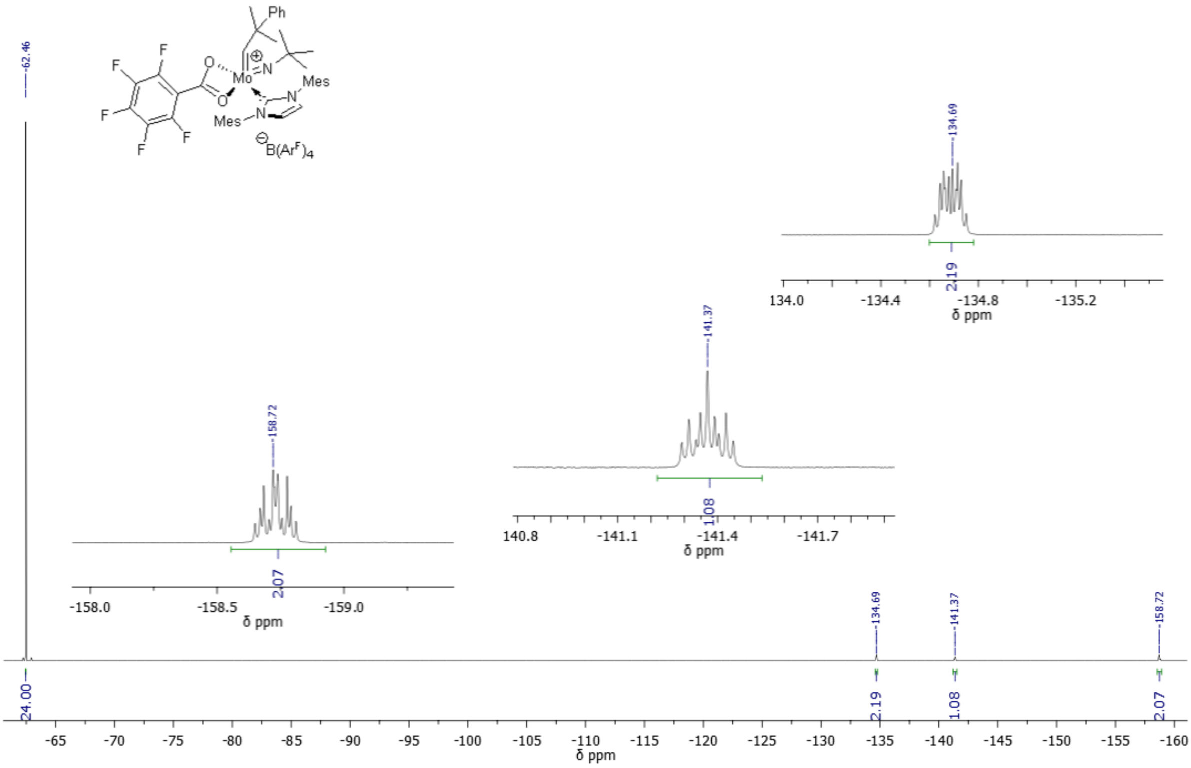

Figure S55. ^{19}F NMR (375 MHz, 25 °C, $CDCl_3$) of **11**.

^{13}C NMR (101 MHz, $CDCl_3$) δ = 315.4, 182.2, 180.0, 162.1, 148.5, 147.2, 145.9, 141.9, 139.5, 136.9, 135.5, 135.0, 134.8, 134.5, 130.7, 130.5, 129.2, 128.7, 127.2, 126.3, 126.1, 126.0, 123.3, 120.6, 117.6, 79.1, 55.3, 32.5, 30.0, 29.5, 21.1, 17.7.

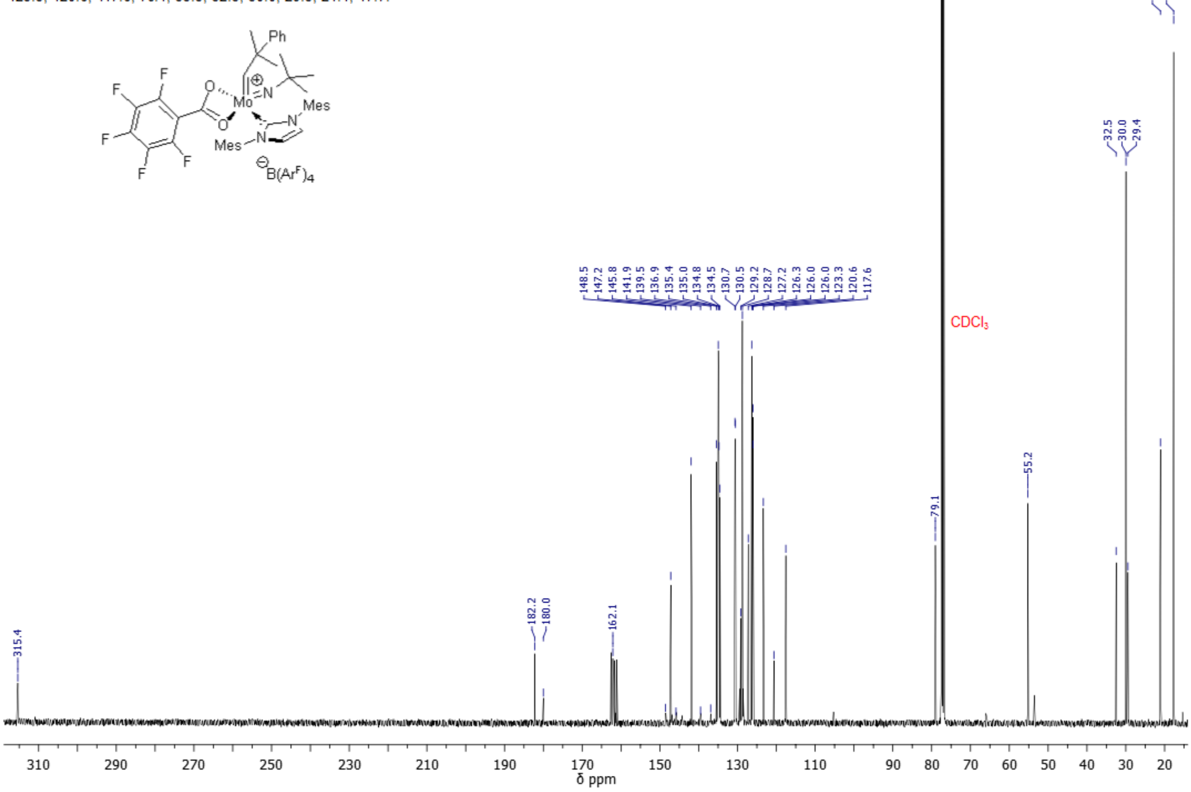

Figure S56. ^{13}C NMR (100 MHz, 25 °C, $CDCl_3$) of **11**.

^1H NMR (400 MHz, CD_2Cl_2) δ = 12.99, 7.44, 7.11, 7.04, 7.03, 6.97, 6.95, 6.90, 6.80, 2.29, 2.07, 1.98, 1.39, 1.04.

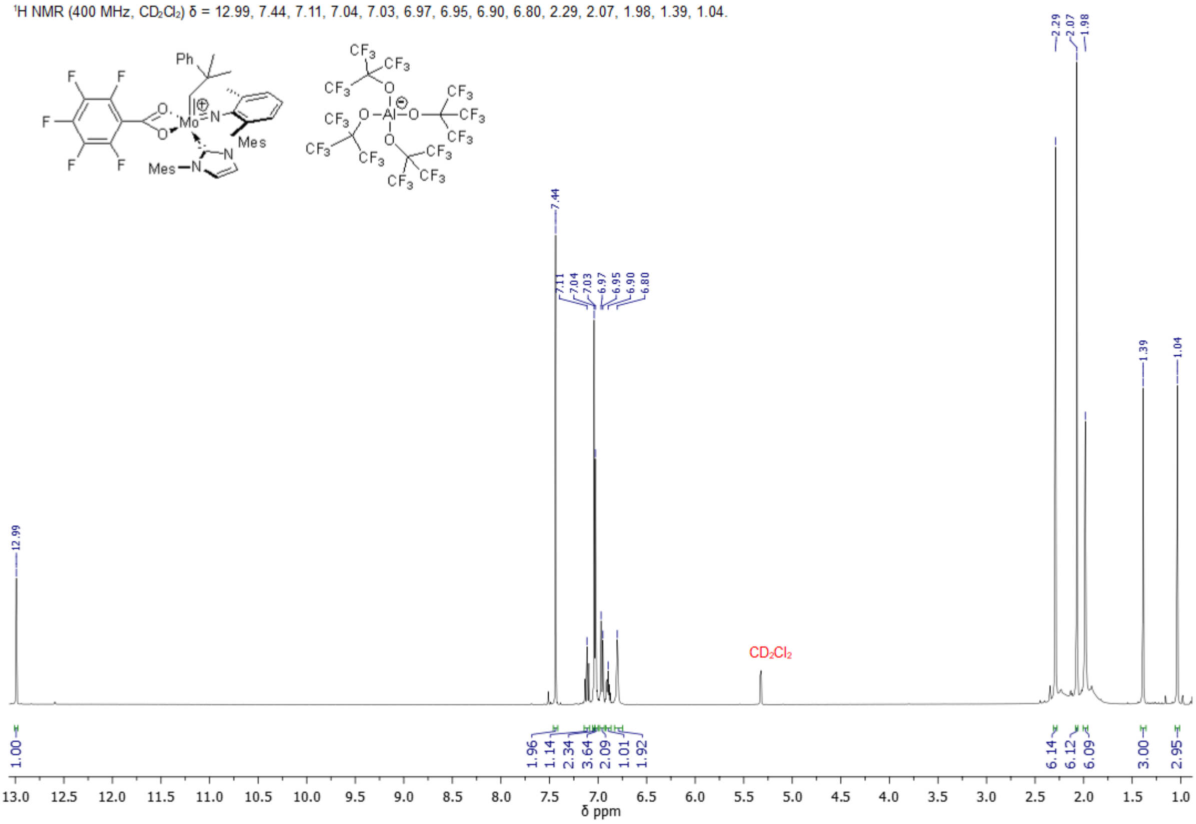

Figure S57. ^1H NMR (400 MHz, 25 °C, CD_2Cl_2) of **12**.

^{19}F NMR (376 MHz, CD_2Cl_2) δ = -75.75, -135.61, -142.79, -159.83.

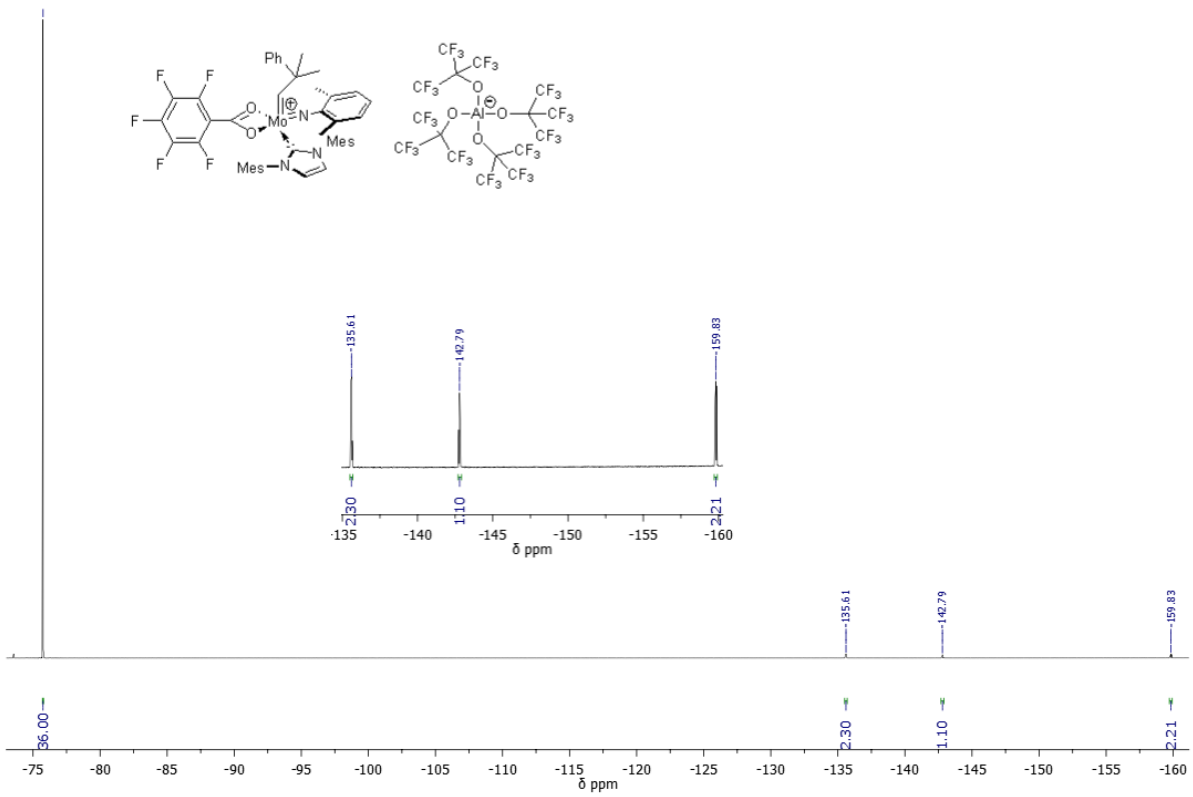

Figure S58. ^{19}F NMR (375 MHz, 25 °C, CD_2Cl_2) of **12**.

^{13}C NMR (101 MHz, CD_2Cl_2) δ = 312.1, 182.7, 179.2, 154.4, 147.2, 142.3, 136.1, 135.6, 133.9, 130.6, 130.4, 130.4, 130.4, 128.7, 128.4, 127.2, 126.4, 126.3, 123.3, 120.4, 117.5, 57.0, 29.1, 29.0, 21.3, 19.8, 18.0, 18.0.

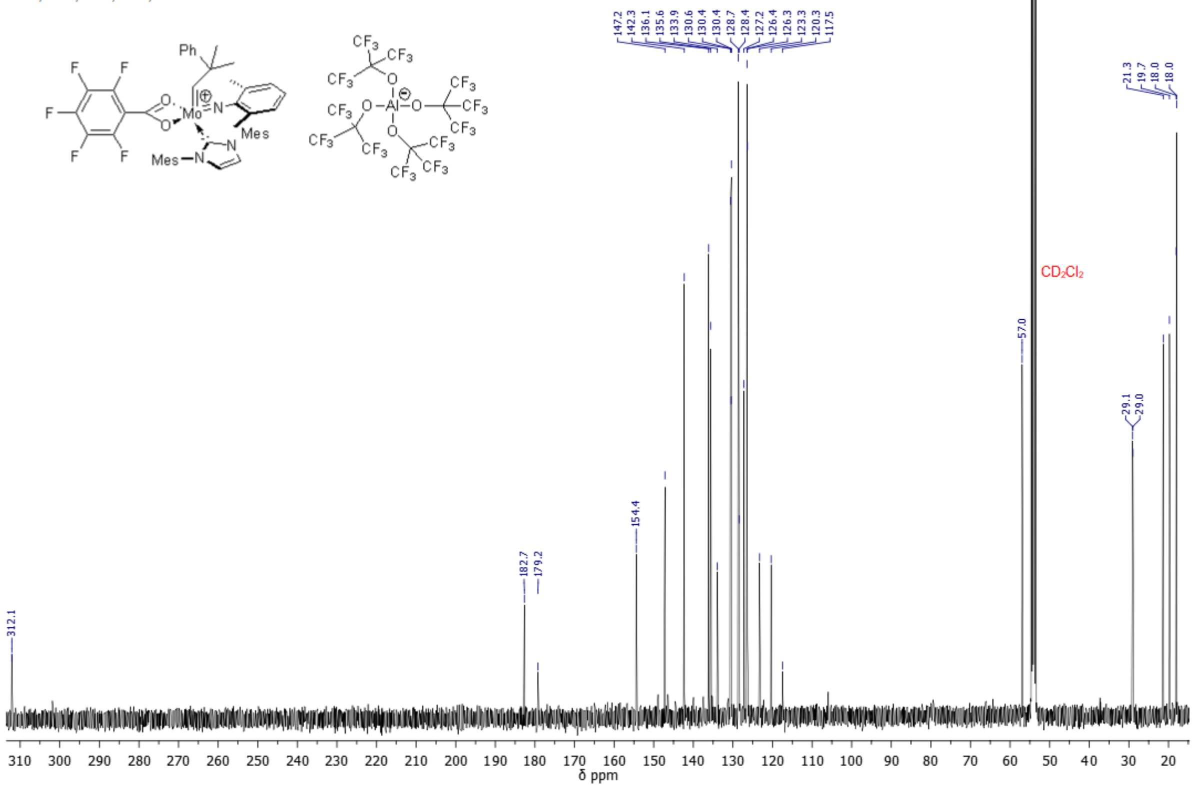

Figure S59. ^{13}C NMR (100 MHz, 25 °C, CD_2Cl_2) of **12**.

^{1}H NMR (400 MHz, CD_2Cl_2) δ = 13.01, 7.40, 7.20, 7.10, 7.01, 6.87, 6.78, 6.49, 2.27, 2.20, 2.08, 1.99, 1.58, 1.37.

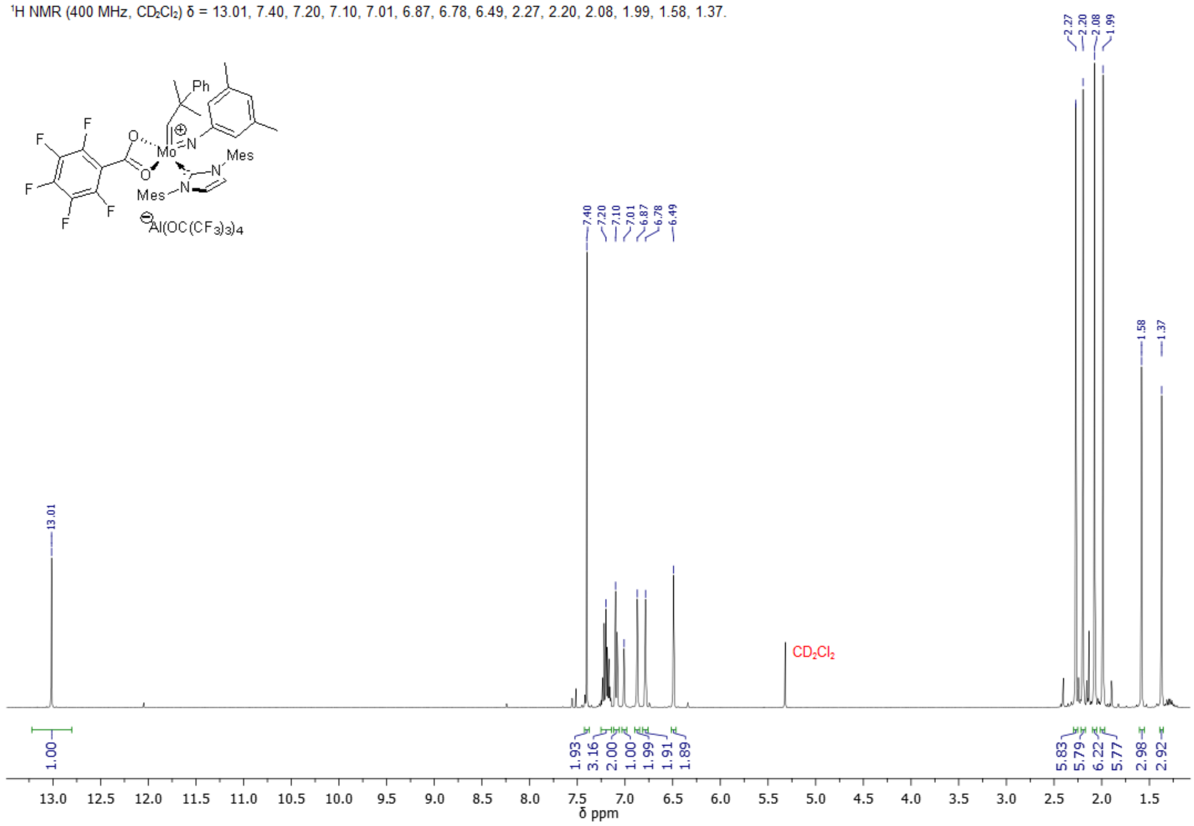

Figure S60. ^{1}H NMR (400 MHz, 25 °C, CD_2Cl_2) of **13**.

^{19}F NMR (376 MHz, CD_2Cl_2) δ = -75.75, -134.88, -142.29, -159.79.

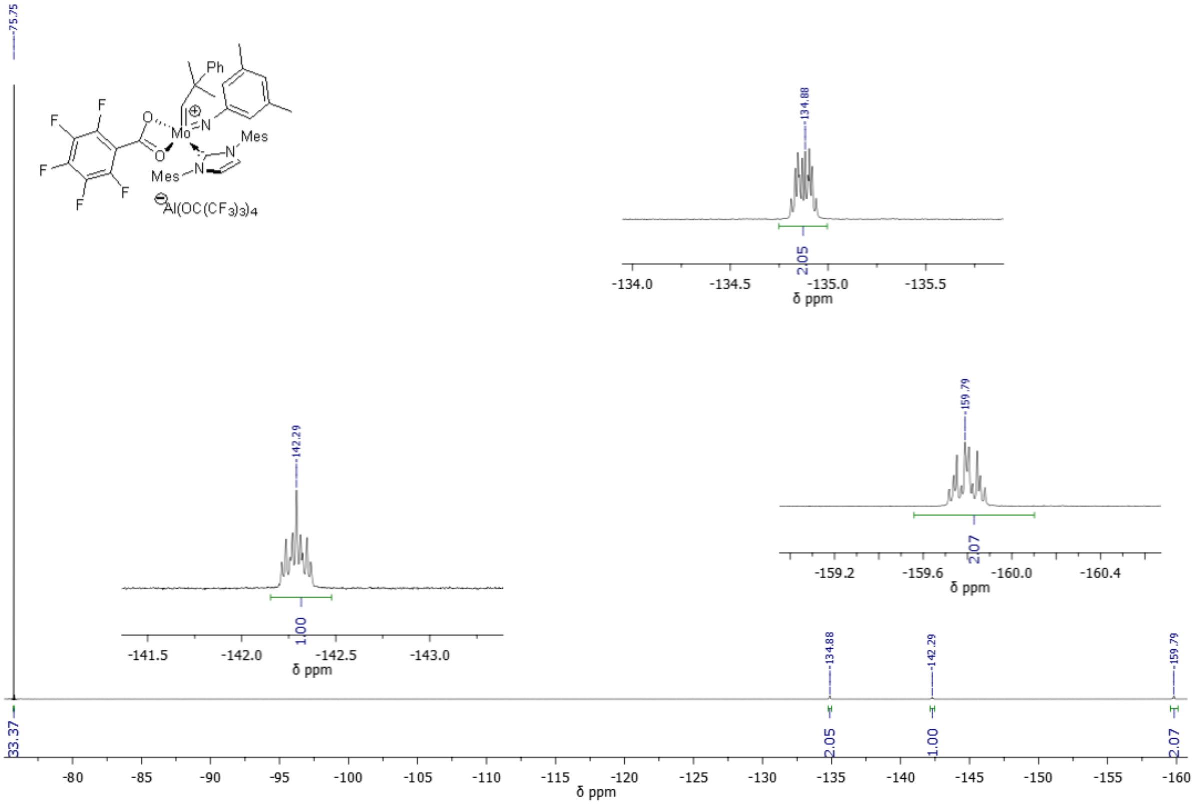

Figure S61. ^{19}F NMR (375 MHz, 25 °C, CD_2Cl_2) of **13**.

^{13}C NMR (101 MHz, CD_2Cl_2) δ = 313.5, 183.6, 180.5, 154.3, 149.1, 146.7, 146.5, 141.8, 139.9, 138.9, 137.5, 135.9, 134.7, 134.6, 133.2, 130.5, 130.5, 129.1, 127.5, 126.7, 126.2, 126.1, 121.8, 56.7, 33.0, 28.9, 21.3, 21.3, 18.1, 18.0.

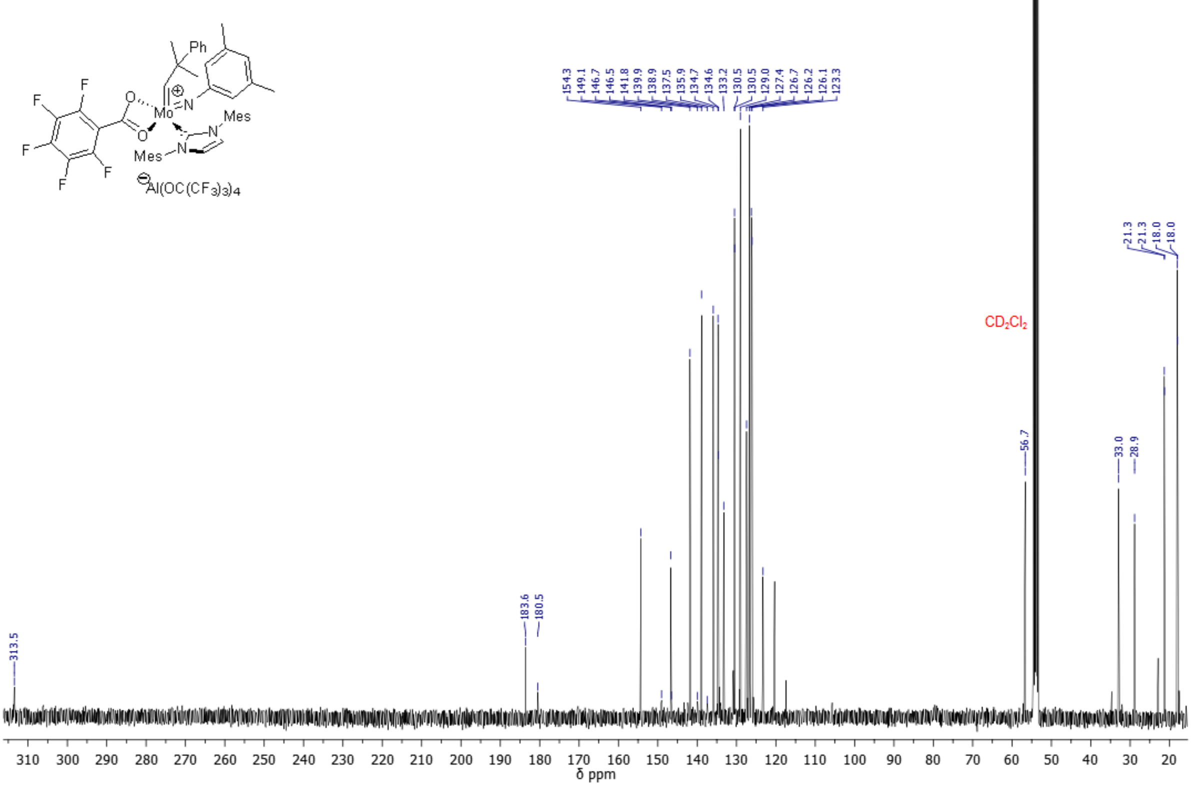

Figure S62. ^{13}C NMR (100 MHz, 25 °C, CD_2Cl_2) of **13**.

^{1}H NMR (400 MHz, $CDCl_3$) δ = 12.75, 7.70, 7.50, 7.34, 7.23, 7.15, 6.91, 6.81, 2.17, 2.08, 2.00, 1.14.

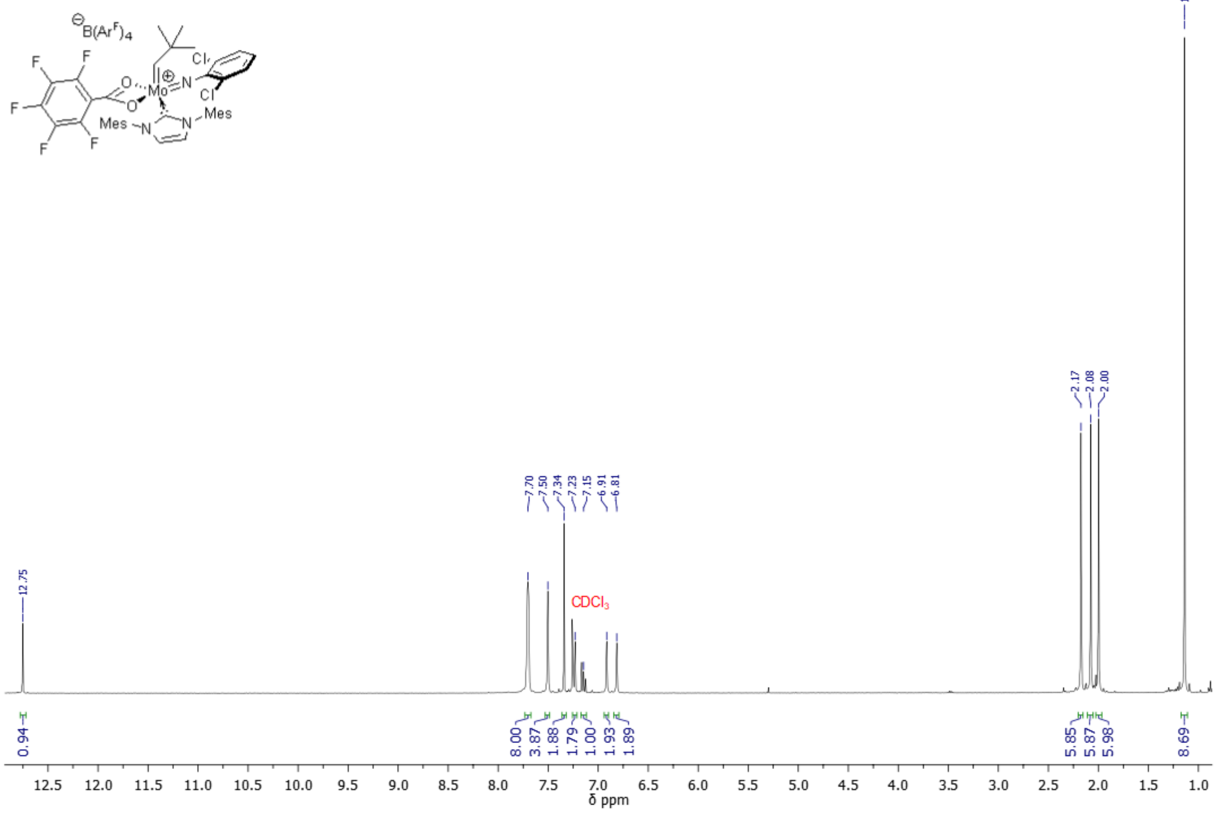

Figure S63. ^{1}H NMR (400 MHz, 25 °C, $CDCl_3$) of **14**.

^{13}C NMR (101 MHz, $CDCl_3$) δ = 321.2, 183.7, 180.0, 161.9, 148.8, 148.7, 146.0, 141.5, 139.5, 136.9, 135.7, 134.9, 134.4, 134.2, 130.6, 130.4, 129.9, 129.0, 128.3, 125.6, 124.7, 117.6, 105.1, 50.3, 30.6, 21.2, 18.1, 17.9.

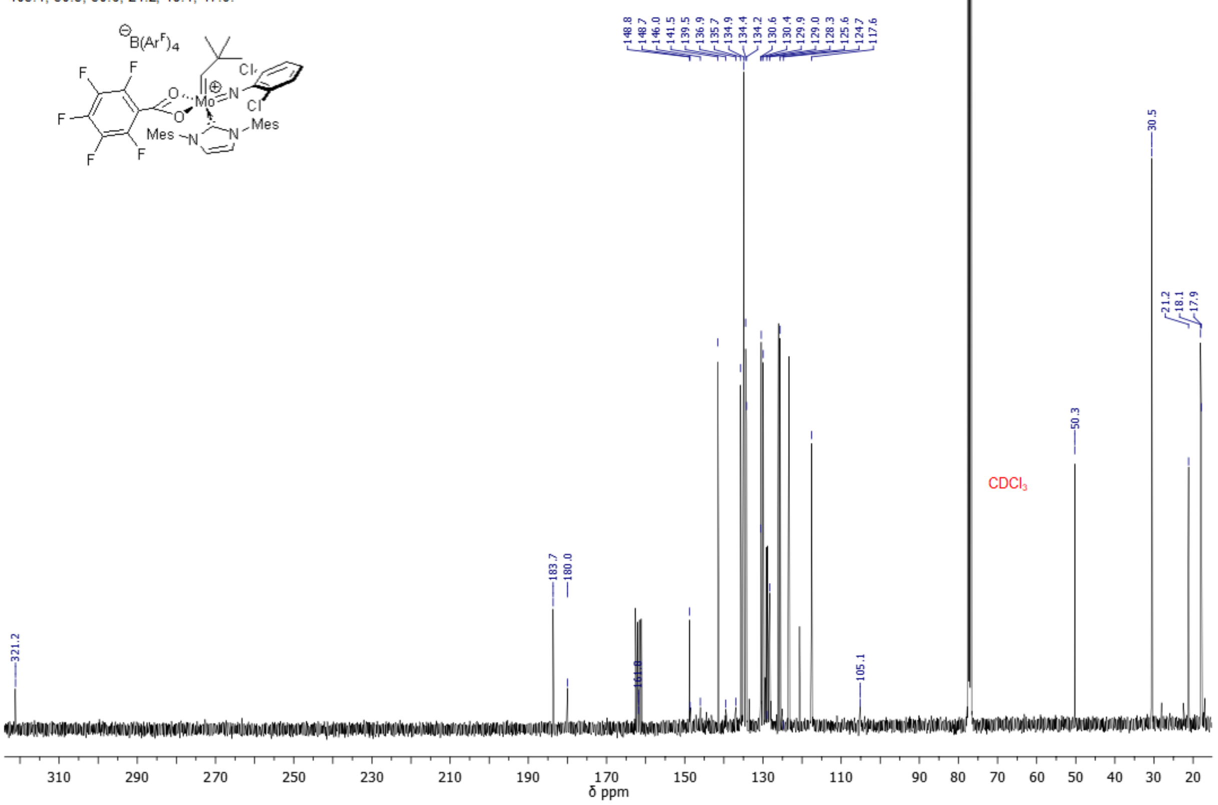

Figure S64. ^{13}C NMR (100 MHz, 25 °C, $CDCl_3$) of **14**.

^{19}F NMR (376 MHz, $CDCl_3$) δ = -62.45, -134.52, -140.55, -158.35.

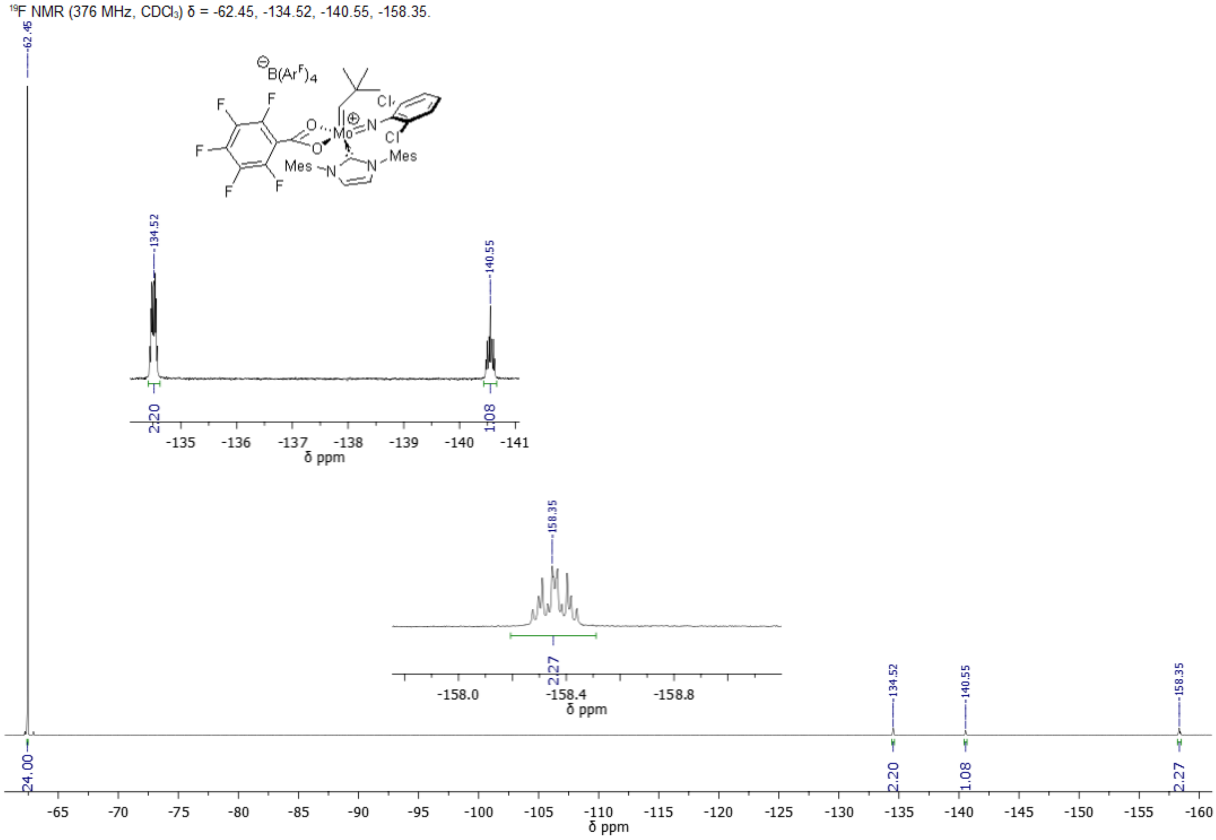

Figure S65. ^{19}F NMR (375 MHz, 25 °C, $CDCl_3$) of **14**.

^{1}H NMR (400 MHz, CD_2Cl_2) δ = 12.81, 8.02, 7.88, 7.43, 7.34, 7.32, 7.24, 7.06, 6.82, 2.28, 2.06, 2.04, 1.09.

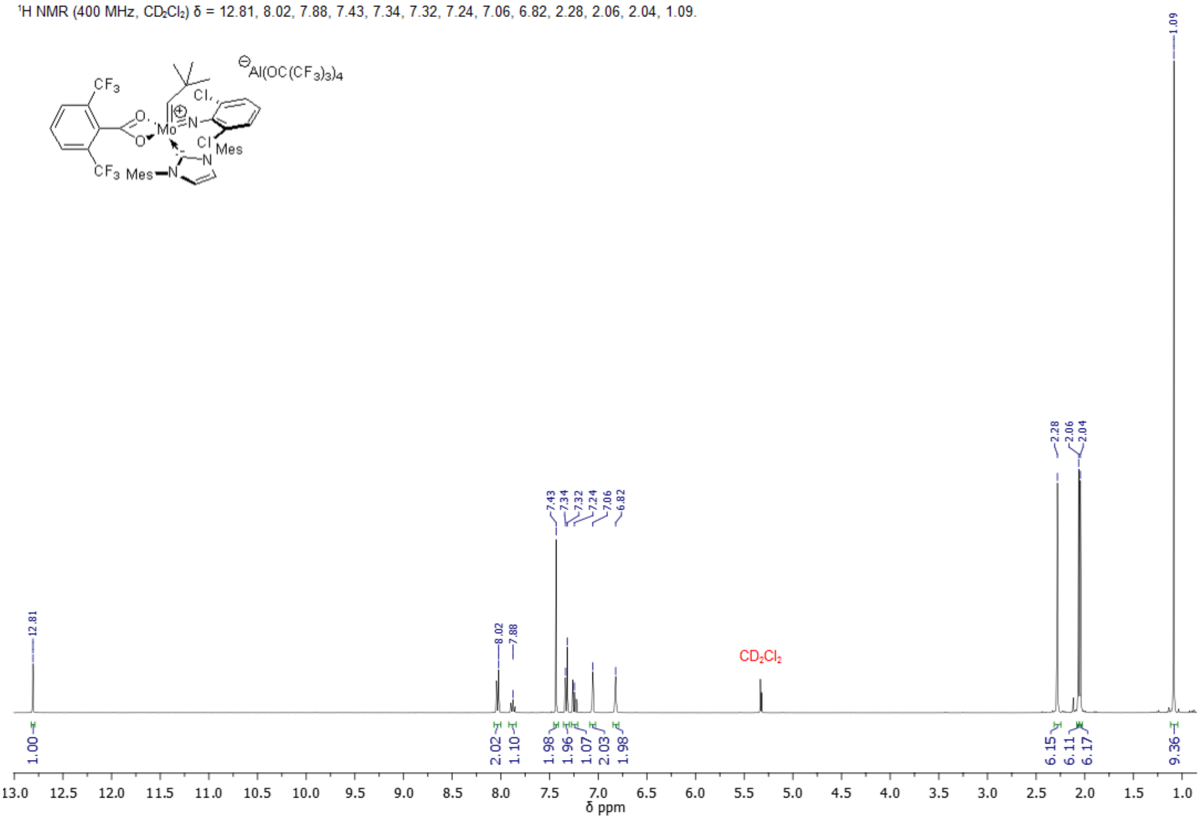

Figure S66. ^{1}H NMR (400 MHz, 25 °C, CD_2Cl_2) of **15a**.

^{13}C NMR (101 MHz, CD_2Cl_2) δ = 321.5, 186.3, 183.7, 149.9, 142.0, 136.1, 135.3, 134.7, 132.7, 131.1, 131.0, 131.0, 130.1, 128.7, 51.0, 30.3, 21.4, 18.4, 18.2.

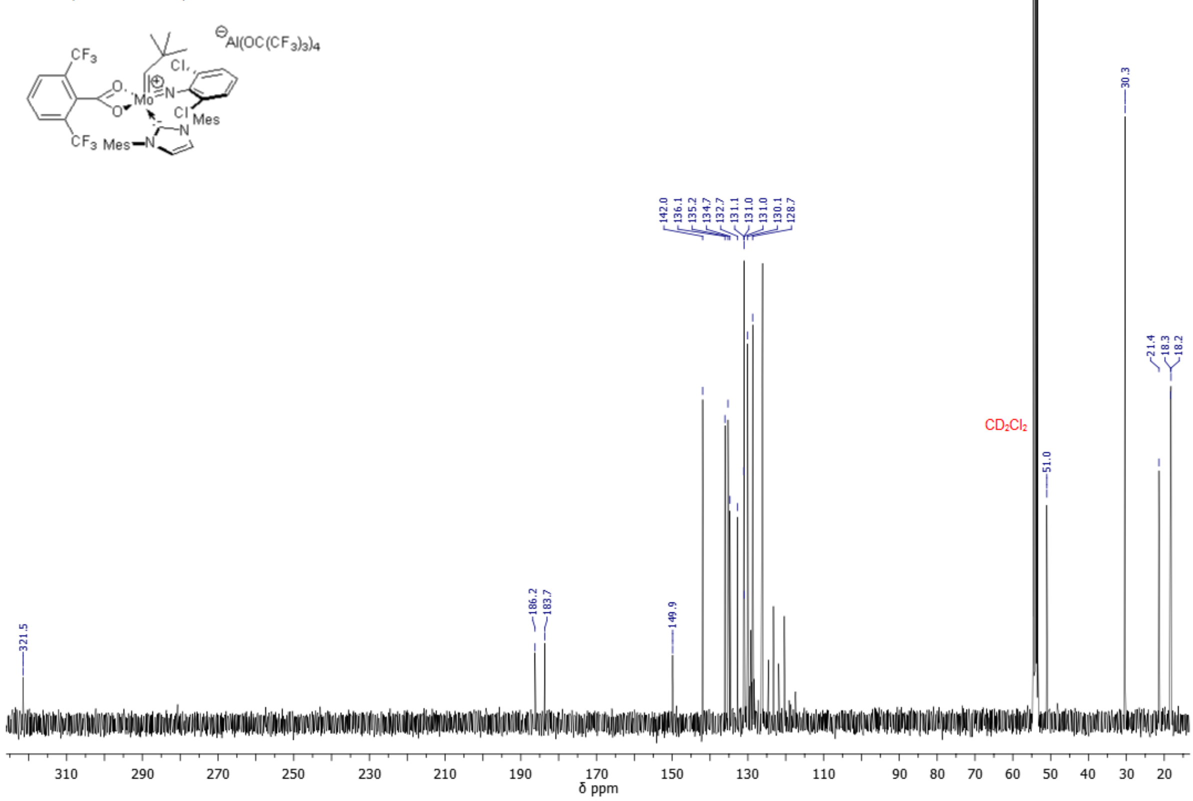

Figure S67. ^{13}C NMR (100 MHz, 25 °C, CD_2Cl_2) of **15a**.

^{19}F NMR (376 MHz, CD_2Cl_2) δ = -59.15, -75.75.

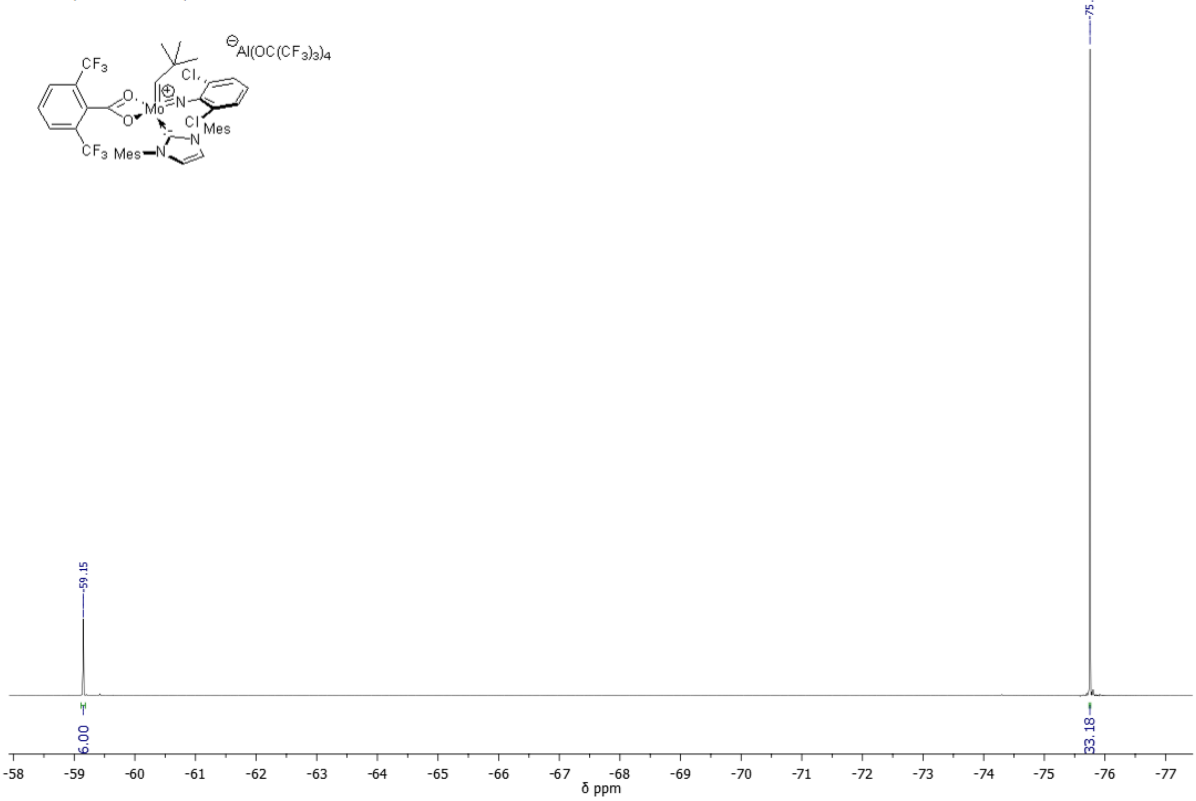

Figure S68. ^{19}F NMR (375 MHz, 25 °C, CD_2Cl_2) of **15a**.

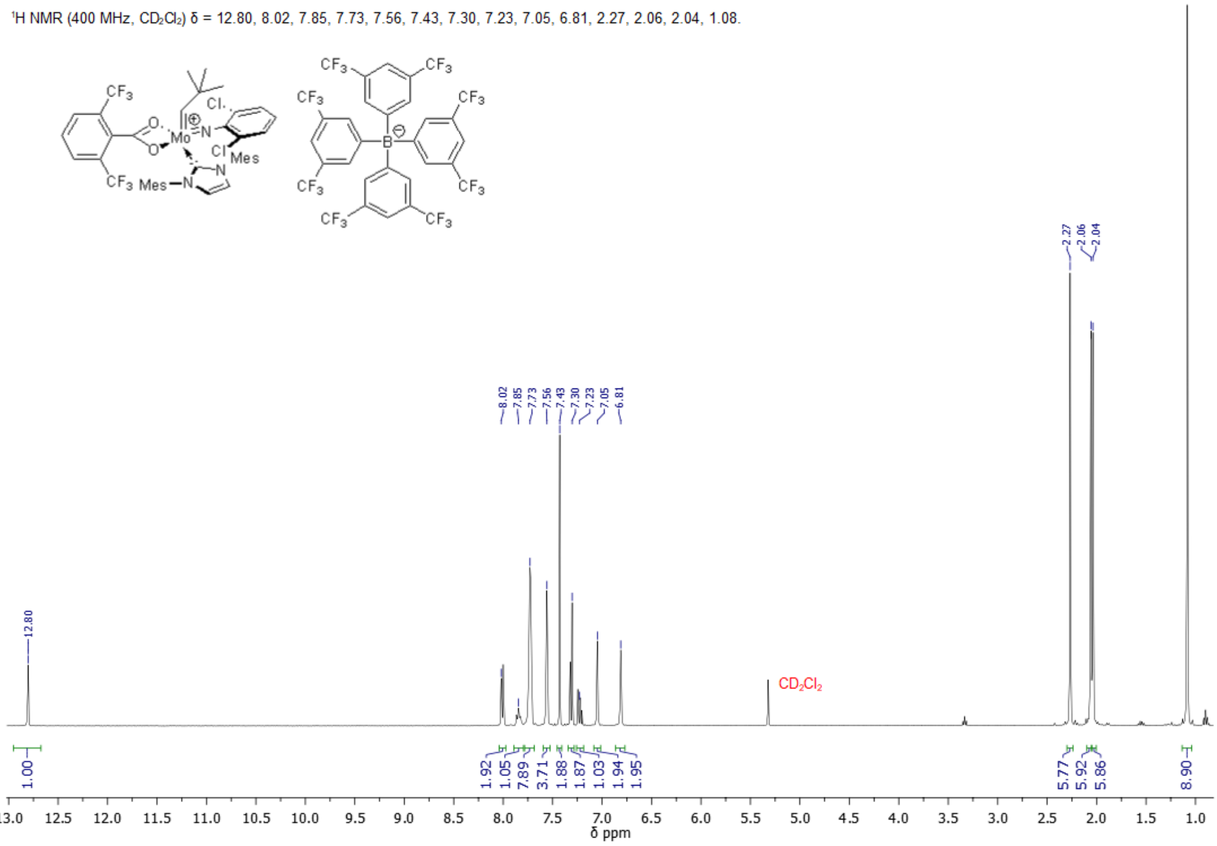

Figure S69. ^{1}H NMR (400 MHz, 25 °C, CD_2Cl_2) of **15b**.

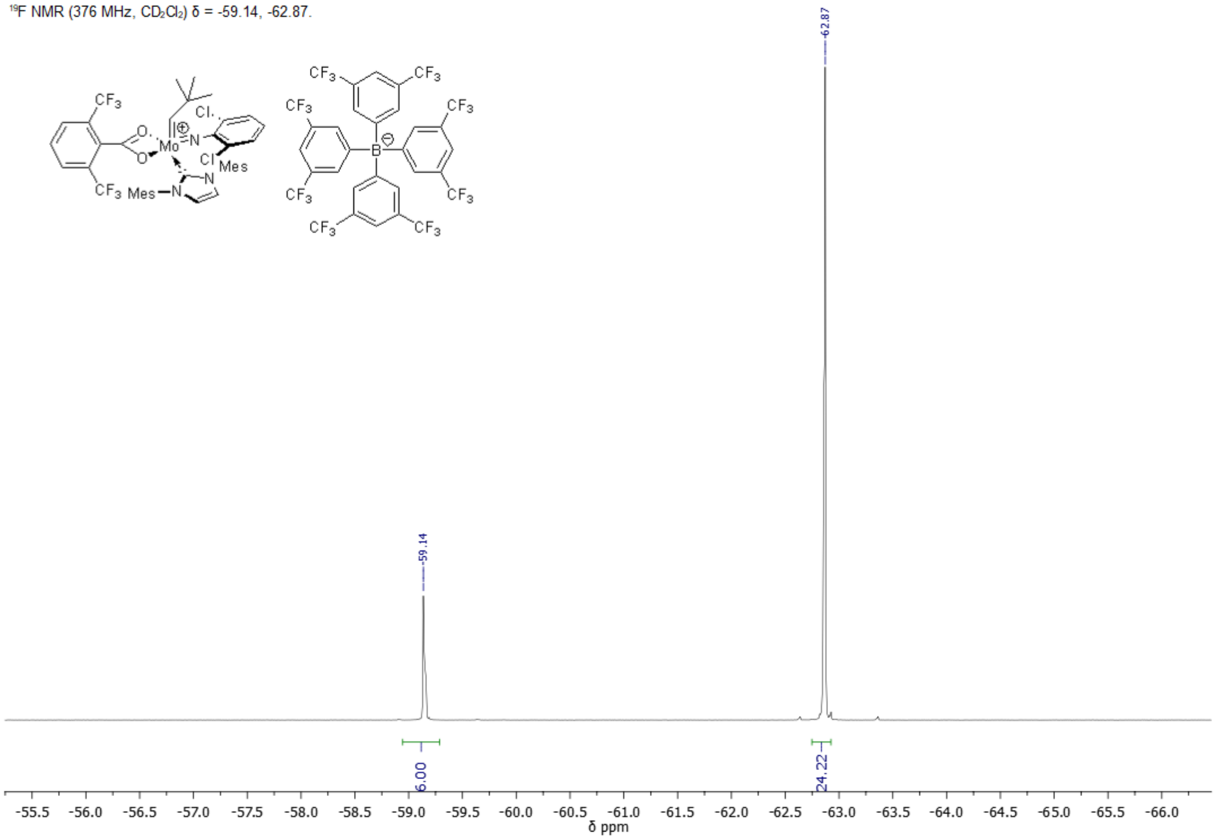

Figure S70. ^{19}F NMR (375 MHz, 25 °C, CD_2Cl_2) of **15b**.

^{13}C NMR (101 MHz, CD_2Cl_2) δ = 321.5, 186.2, 183.7, 162.6, 149.9, 141.9, 136.1, 135.4, 135.2, 134.7, 132.7, 131.1, 131.0, 130.1, 129.2, 128.9, 128.7, 126.5, 126.1, 124.6, 123.8, 121.9, 121.1, 118.0, 51.0, 30.3, 21.4, 18.4, 18.2.

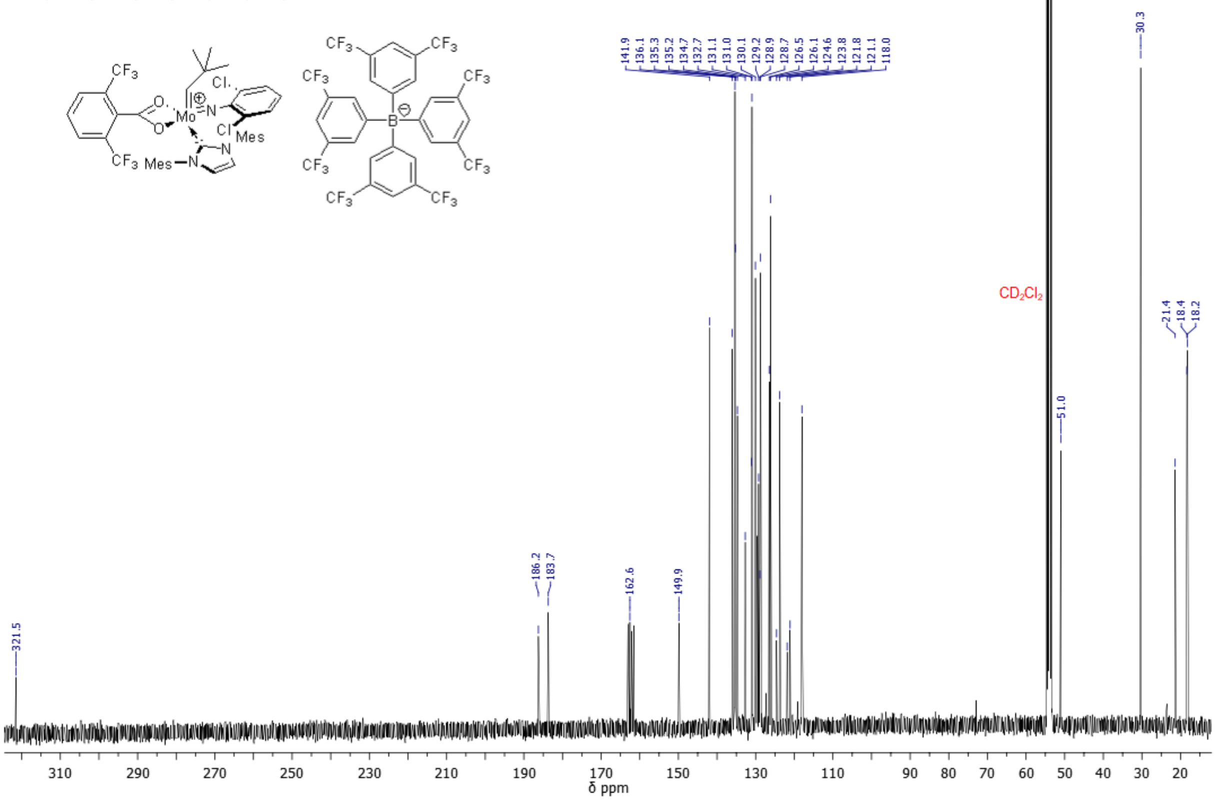

Figure S71. ^{13}C NMR (100 MHz, 25 °C, CD_2Cl_2) of **15b.**

^{1}H NMR (400 MHz, $CDCl_3$) δ = 13.09, 8.00, 7.85, 7.34, 7.28, 7.17, 7.03, 6.83, 4.07, 3.95, 2.32, 2.29, 2.14, 1.85, 1.65, 1.53, 1.45, 1.34, 1.24.

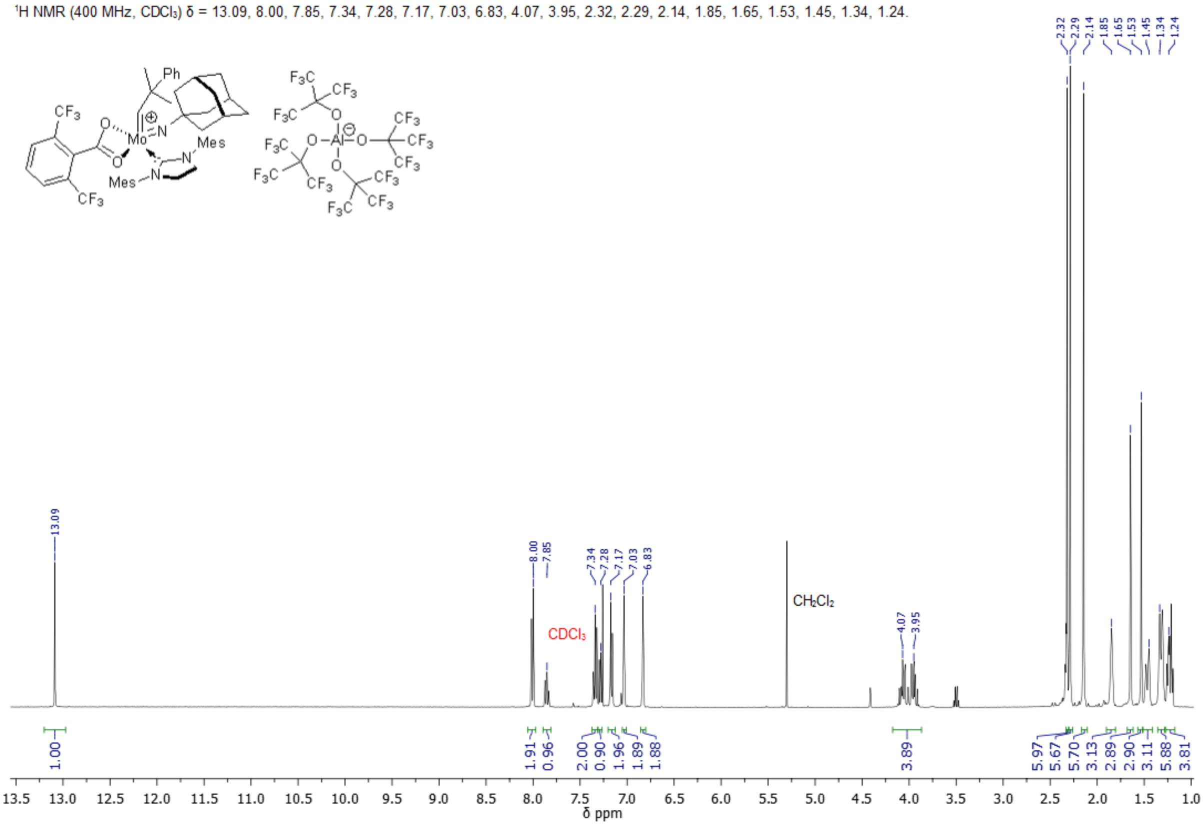

Figure S72. ^{1}H NMR (400 MHz, 25 °C, $CDCl_3$) of **17a**.

^{19}F NMR (376 MHz, CDCl$_3$) δ = -59.22, -75.48.

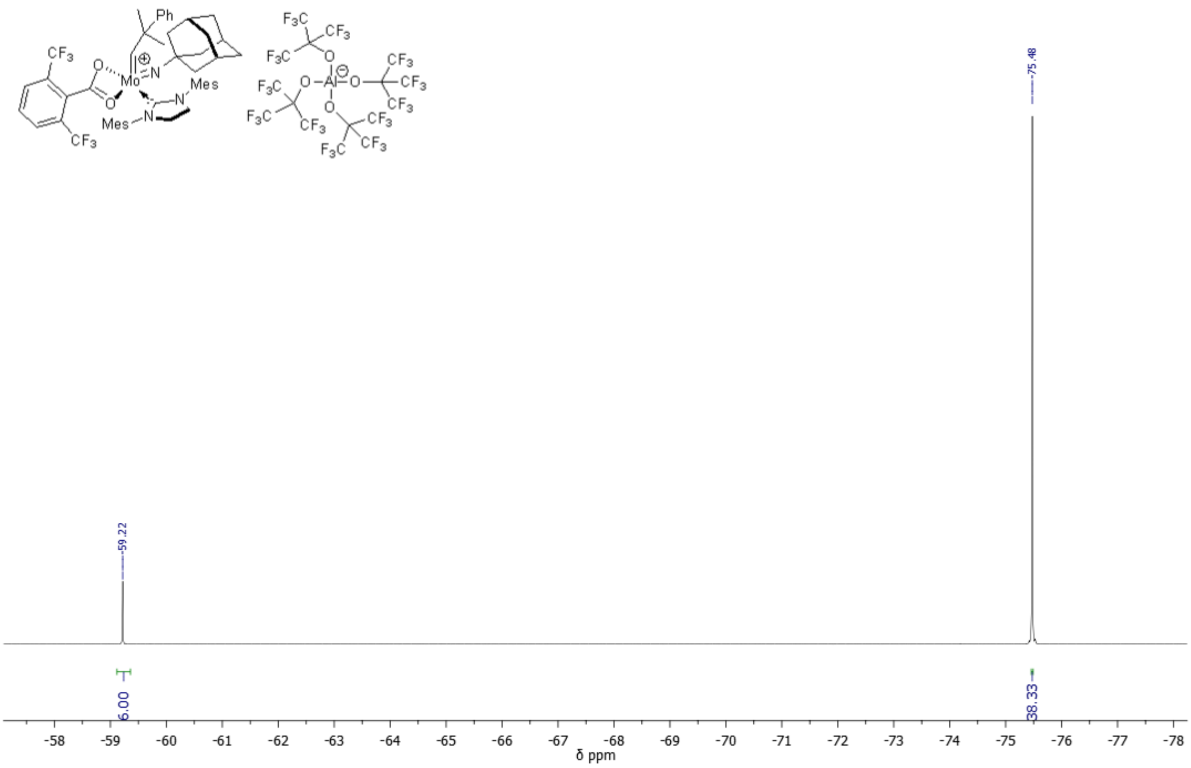

Figure S73. ^{19}F NMR (375 MHz, 25 °C, CDCl$_3$) of **17a**.

^{13}C NMR (101 MHz, CDCl$_3$) δ = 321.4, 207.6, 187.0, 147.4, 140.8, 136.6, 135.5, 134.5, 132.1, 131.0, 130.8, 130.5, 128.8, 127.2, 126.4, 82.0, 55.7, 42.1, 35.0, 32.8, 29.5, 29.3, 21.1, 17.9.

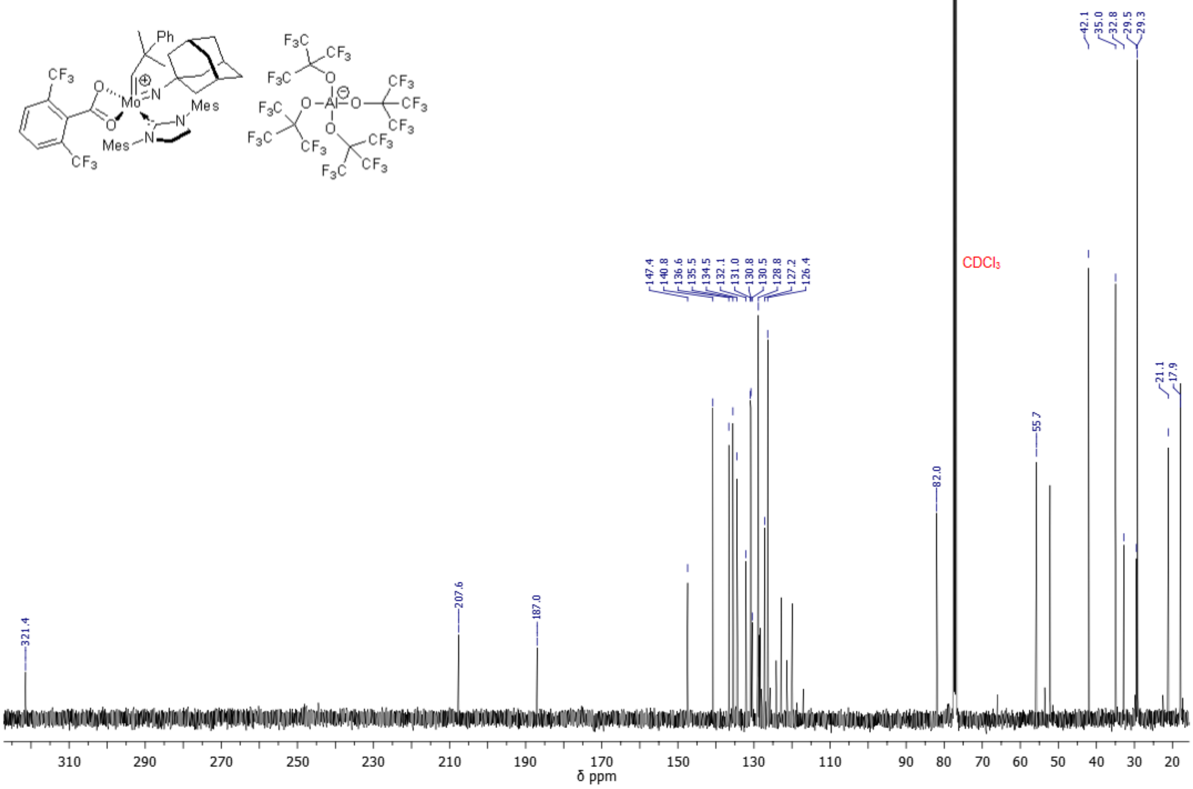

Figure S74. ^{13}C NMR (100 MHz, 25 °C, CDCl$_3$) of **17a**.

^{1}H NMR (400 MHz, CD_2Cl_2) δ = 13.80, 8.04, 7.89, 7.41, 7.30, 7.28, 4.21, 2.14, 2.09, 1.67, 1.32, 1.28.

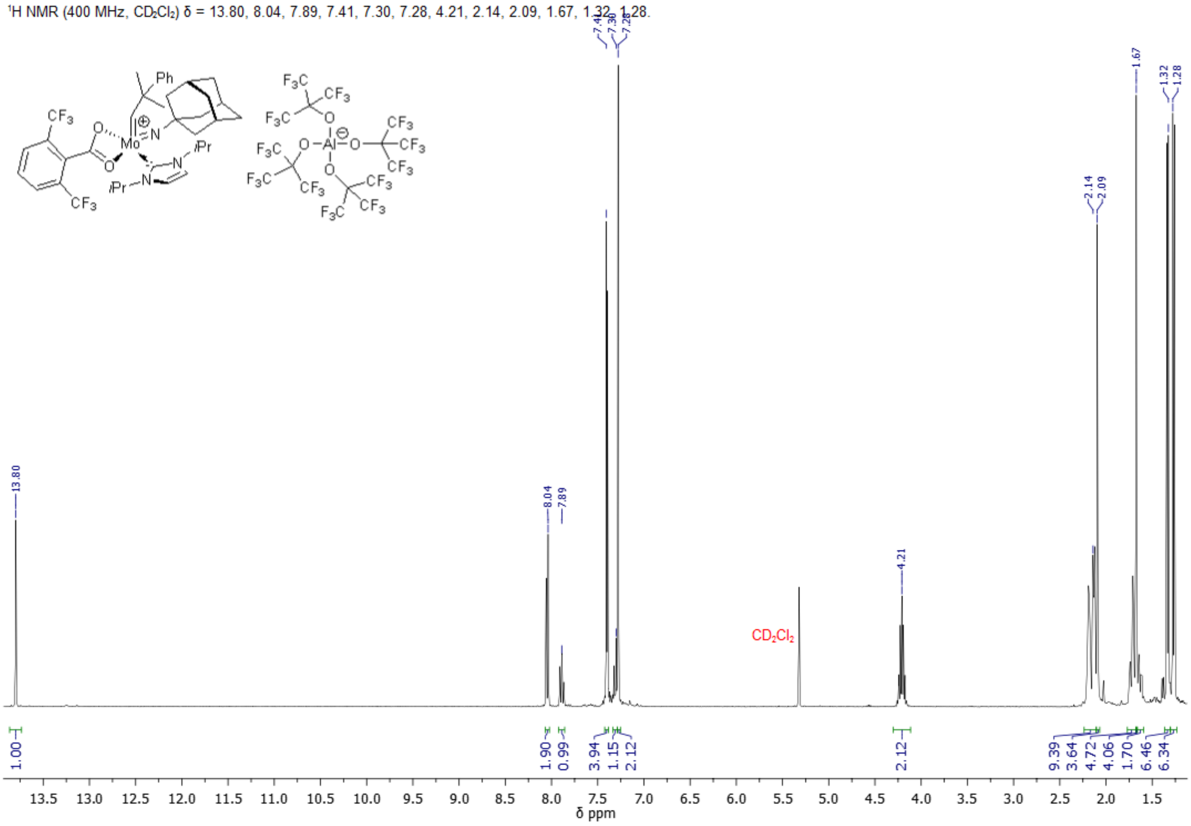

Figure S75. ^{1}H NMR (400 MHz, 25 °C, CD_2Cl_2) of **17b**.

^{13}C NMR (101 MHz, CD_2Cl_2) δ = 308.7, 188.5, 175.5, 146.0, 132.7, 131.0, 129.7, 128.0, 126.2, 120.6, 80.1, 55.1, 54.6, 44.7, 35.8, 31.9, 30.0, 29.6, 23.9, 23.7.

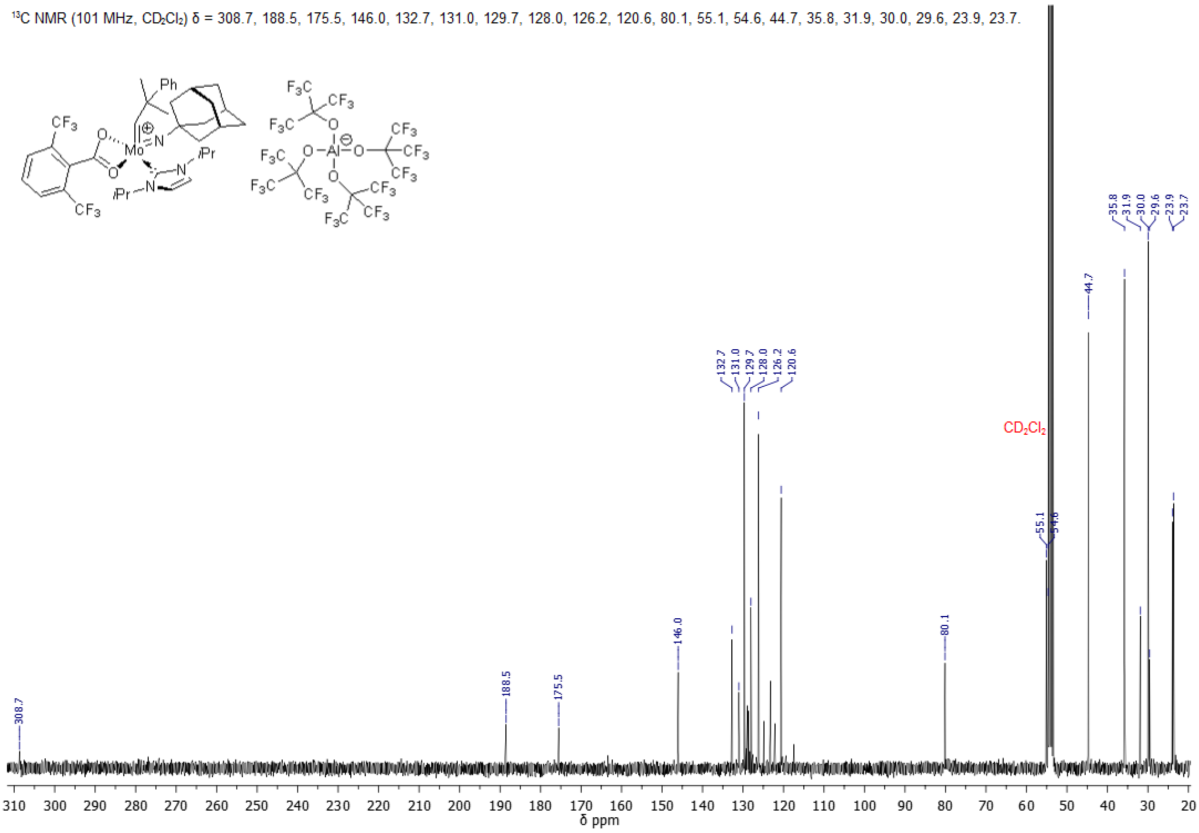

Figure S76. ^{13}C NMR (100 MHz, 25 °C, CD_2Cl_2) of **17b**.

^{19}F NMR (376 MHz, CD_2Cl_2) δ = -59.53, -75.75.

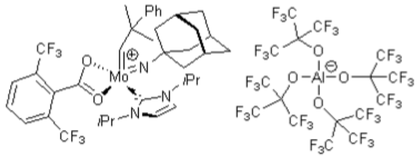

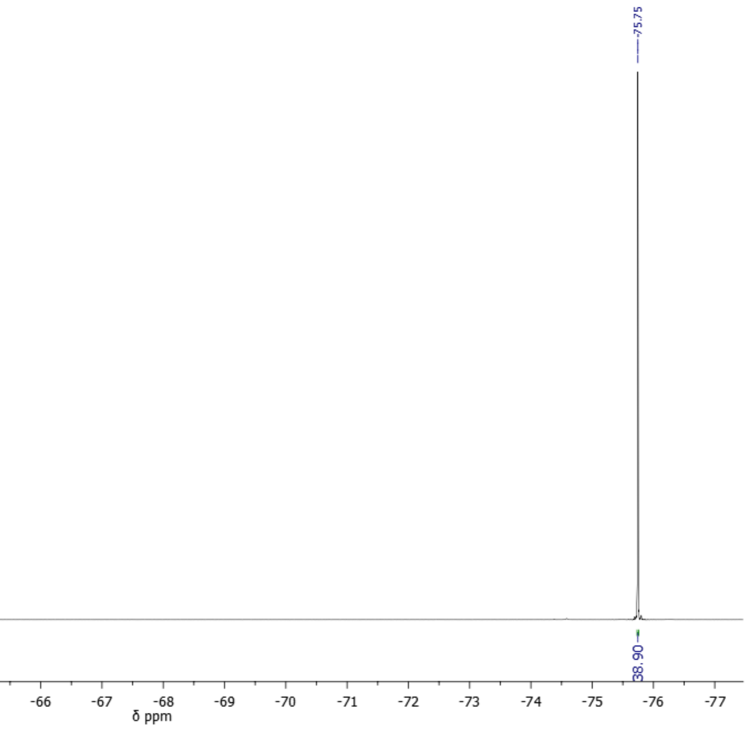

Figure S77. ^{19}F NMR (375 MHz, 25 °C, CD_2Cl_2) of **17b**.

NMR Spectra of Organic Compounds

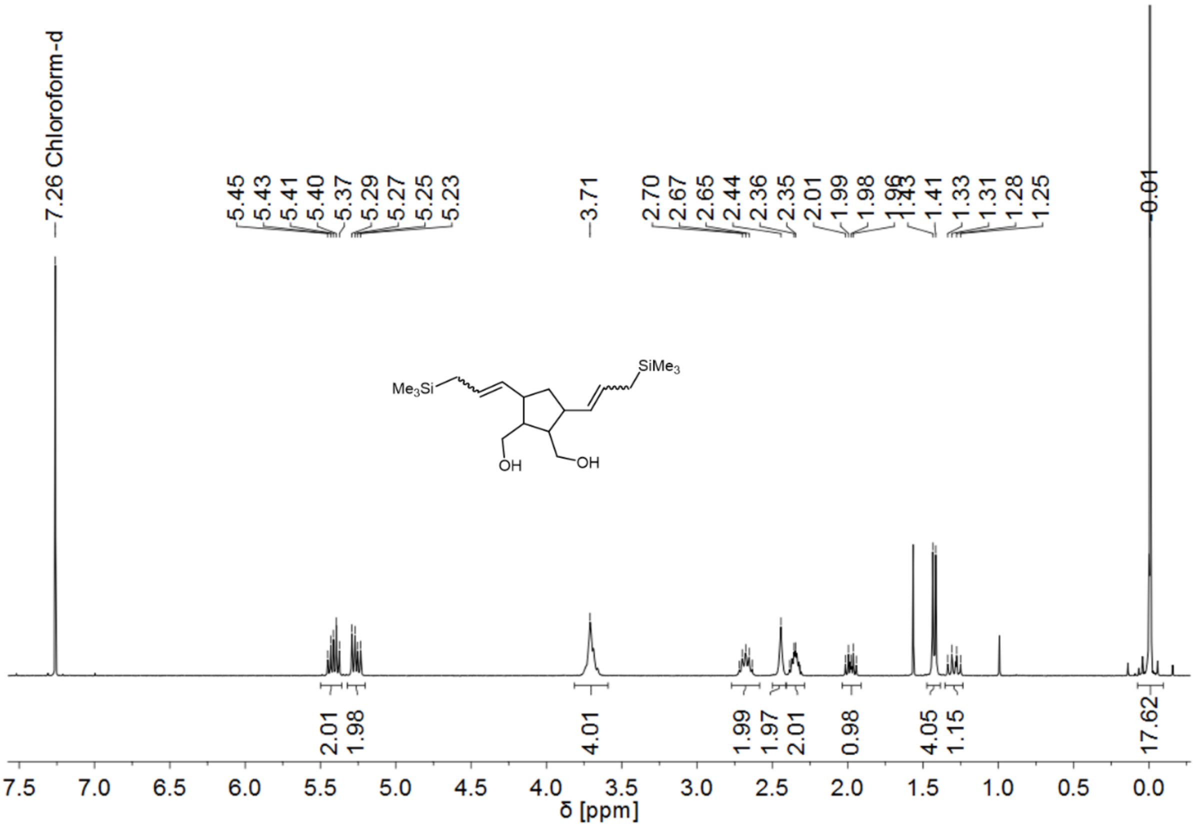

Figure S78. ^{1}H NMR (400 MHz, 25 °C, $CDCl_3$) of (3,5-bis(3-(trimethylsilyl)prop-1-en-1-yl)cyclopentane-1,2-diyl)dimethanol.

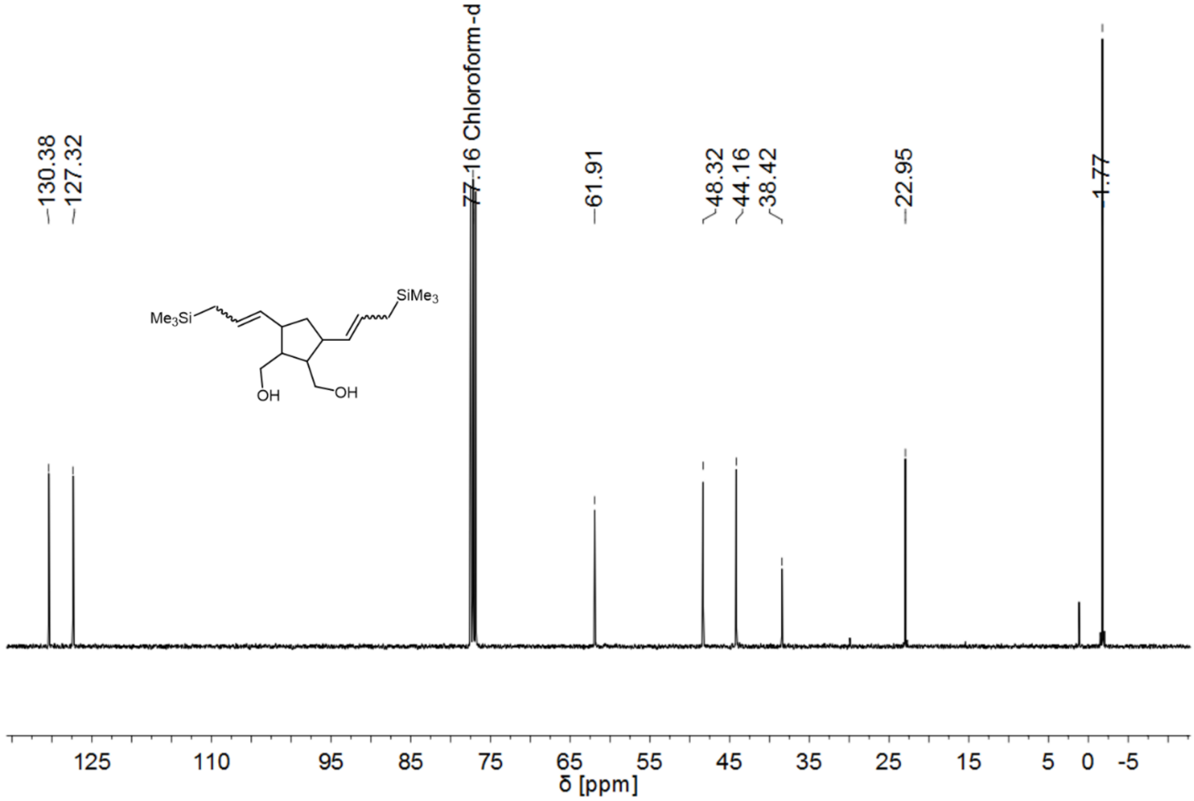

Figure S79. ^{13}C NMR (100 MHz, 25 °C, $CDCl_3$) of (3,5-bis(3-(trimethylsilyl)prop-1-en-1-yl)cyclopentane-1,2-diyl)dimethanol.

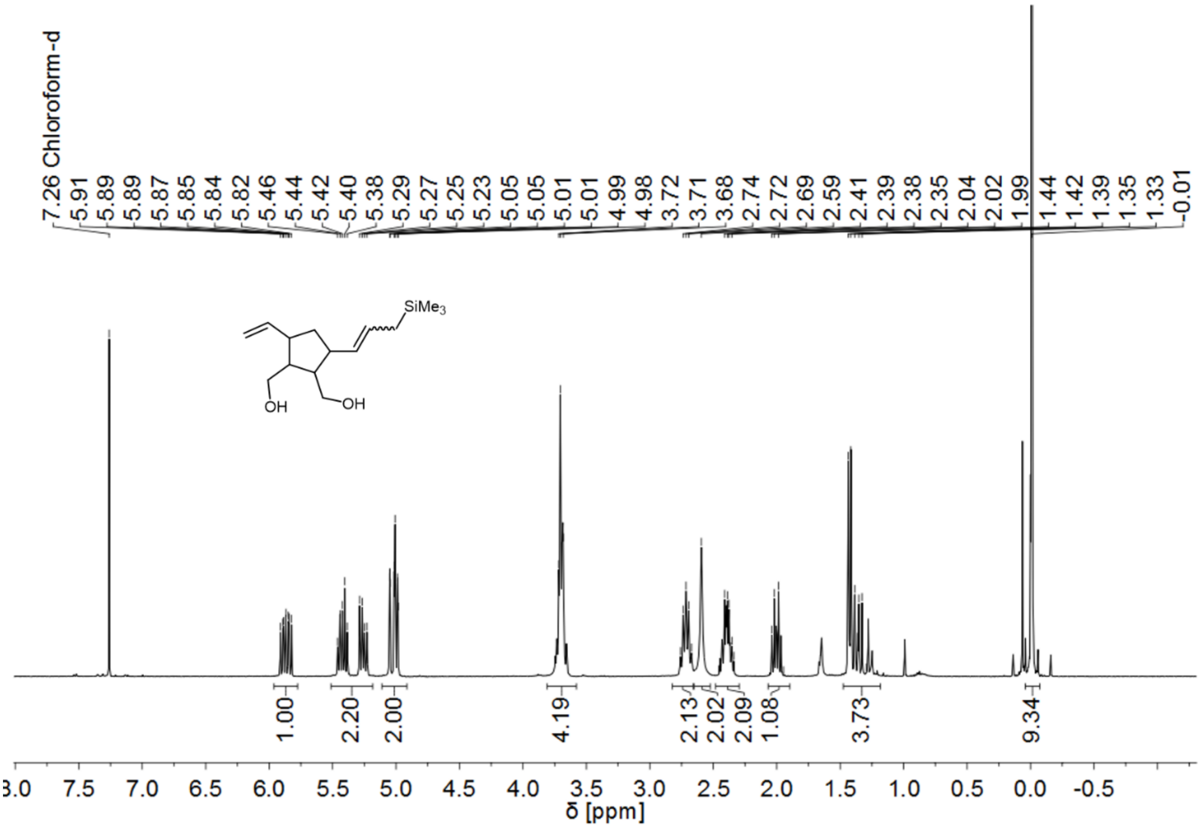

Figure S80. ^{1}H NMR (400 MHz, 25 °C, $CDCl_3$) of (3-(3-(trimethylsilyl)prop-1-en-1-yl)-5-vinylcyclopentane-1,2-diyl)dimethanol.

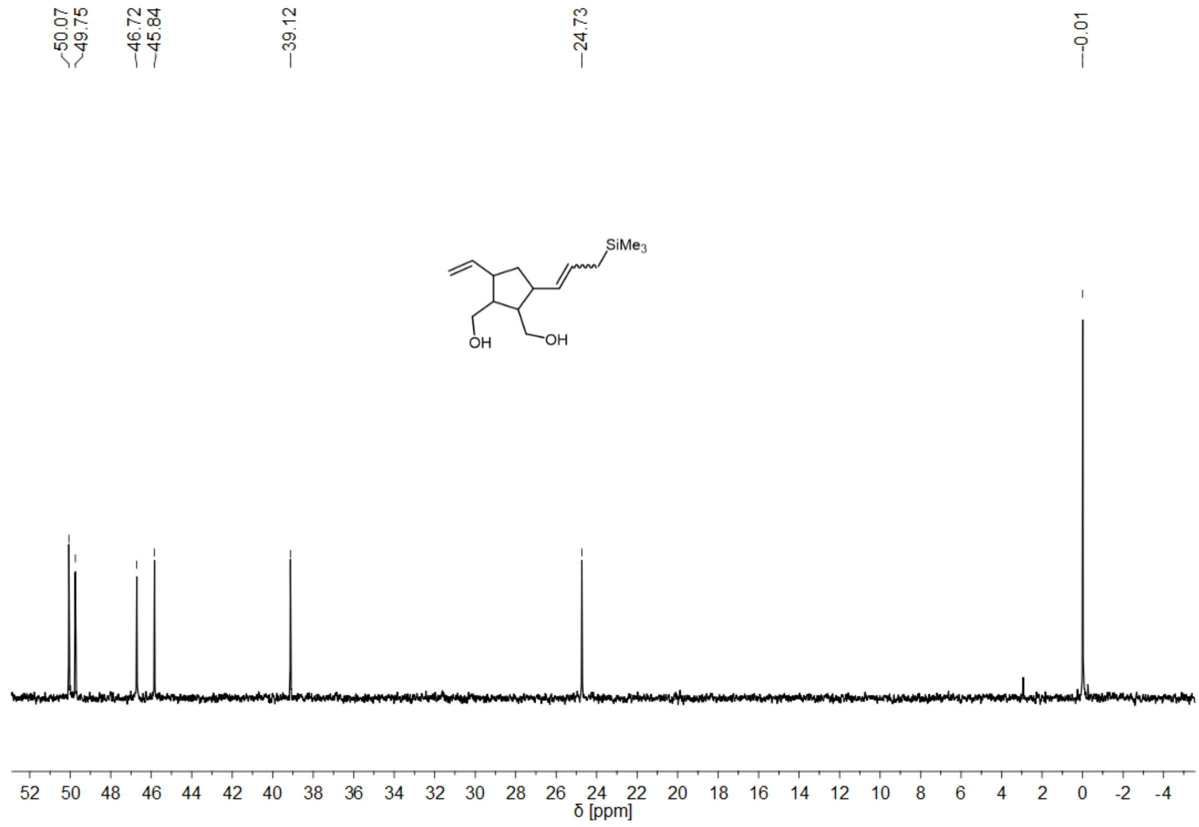

Figure S81. ^{13}C NMR (100 MHz, 25 °C, $CDCl_3$) of (3-(3-(trimethylsilyl)prop-1-en-1-yl)-5-vinylcyclopentane-1,2-diyl)dimethanol.

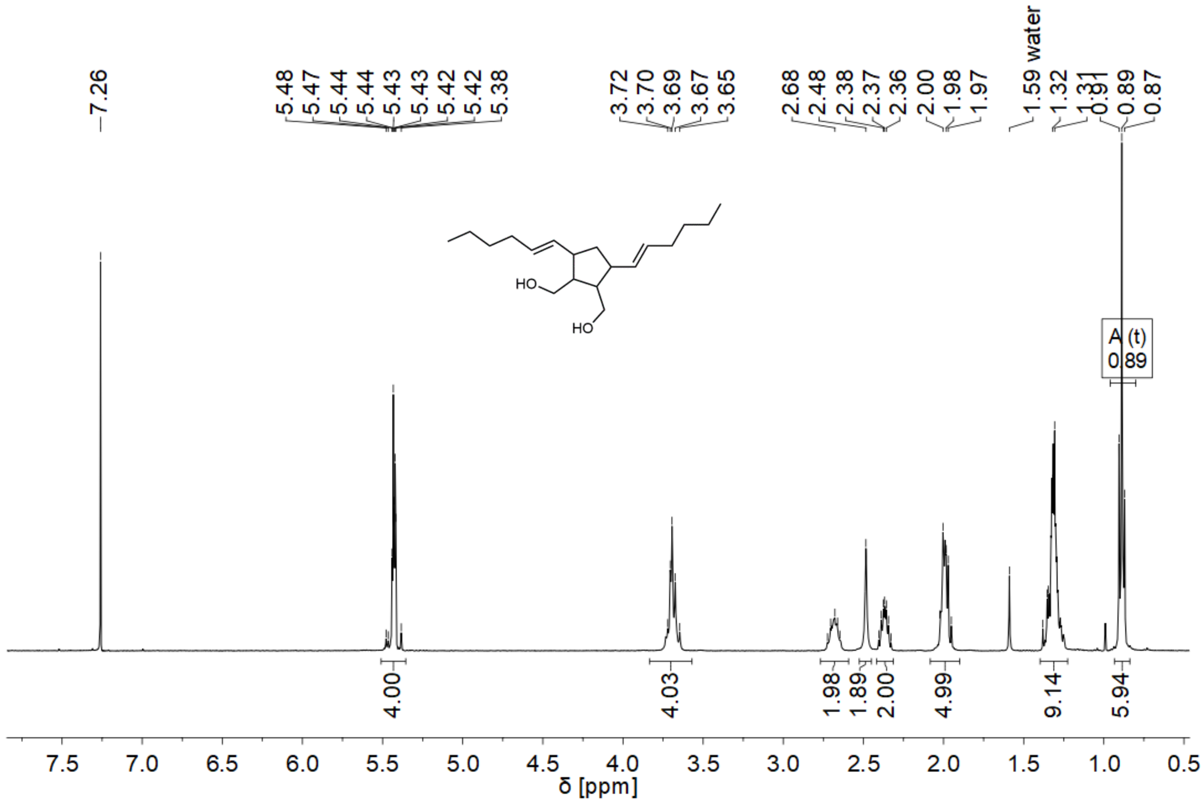

Figure S82. ^{1}H NMR (400 MHz, 25 °C, $CDCl_3$) of (3,5-bis(hex-1-en-1-yl)cyclopentane-1,2-diyl)dimethanol.

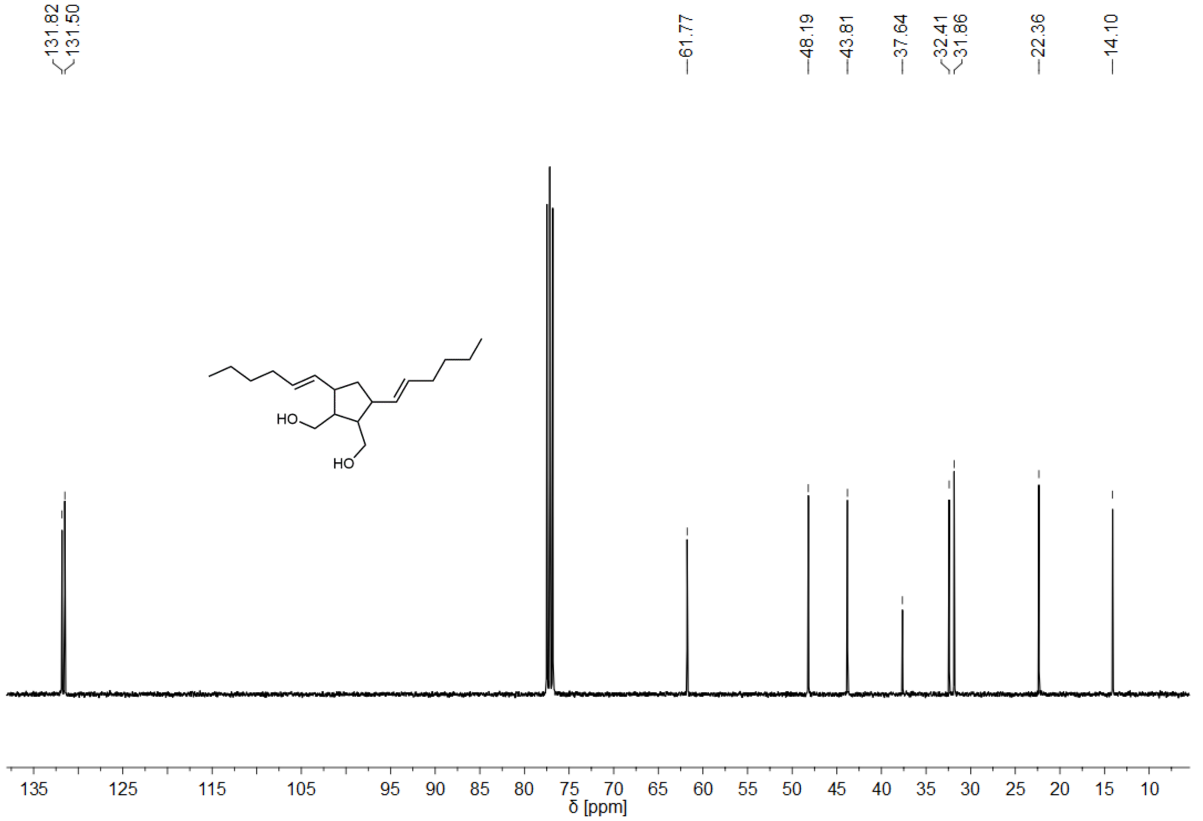

Figure S83. ^{13}C NMR (100 MHz, 25 °C, $CDCl_3$) of (3,5-bis(hex-1-en-1-yl)cyclopentane-1,2-diyl)dimethanol.

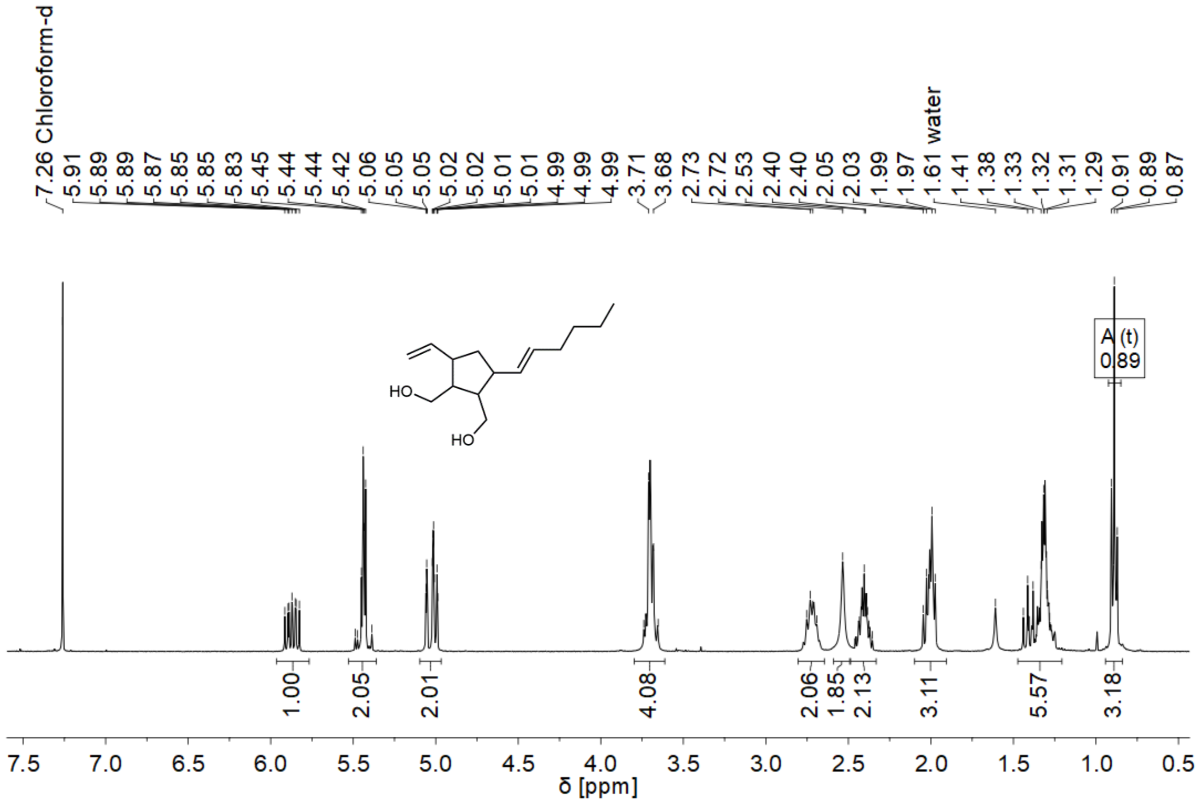

Figure S84. ^{1}H NMR (400 MHz, 25 °C, $CDCl_3$) of (3-(hex-1-en-1-yl)-5-vinylcyclopentane-1,2-diyl)dimethanol.

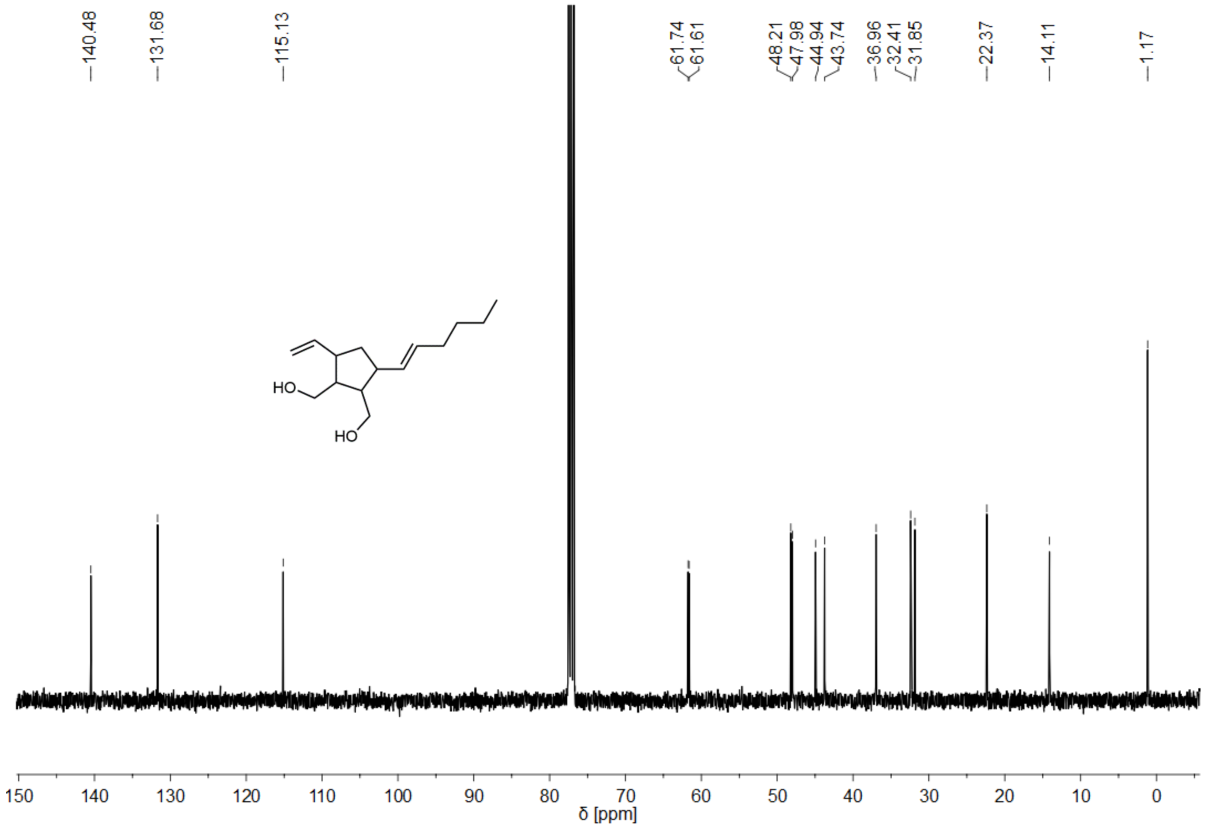

Figure S85. ^{13}C NMR (100 MHz, 25 °C, $CDCl_3$) of (3-(hex-1-en-1-yl)-5-vinylcyclopentane-1,2-diyl)dimethanol.

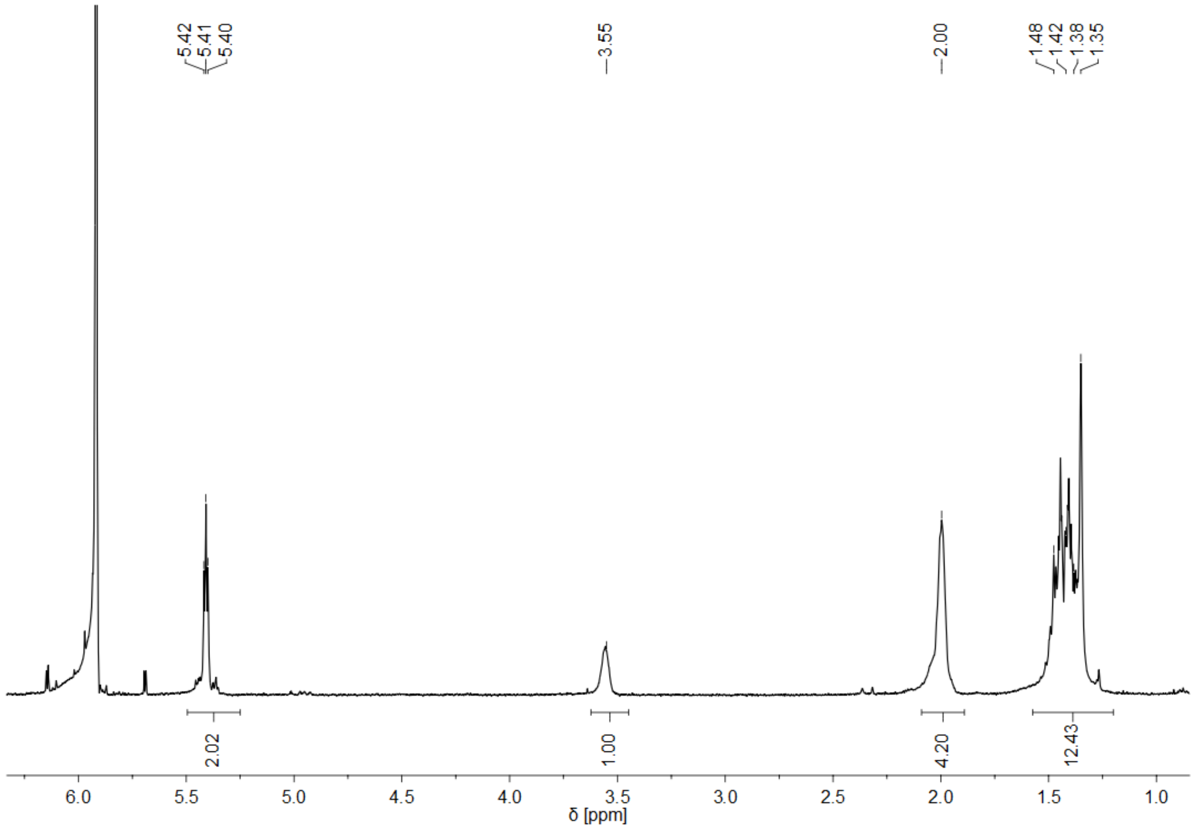

Figure S86. ^{1}H NMR (400 MHz, 100 °C, $C_2D_4Cl_2$) of poly(**M1**).

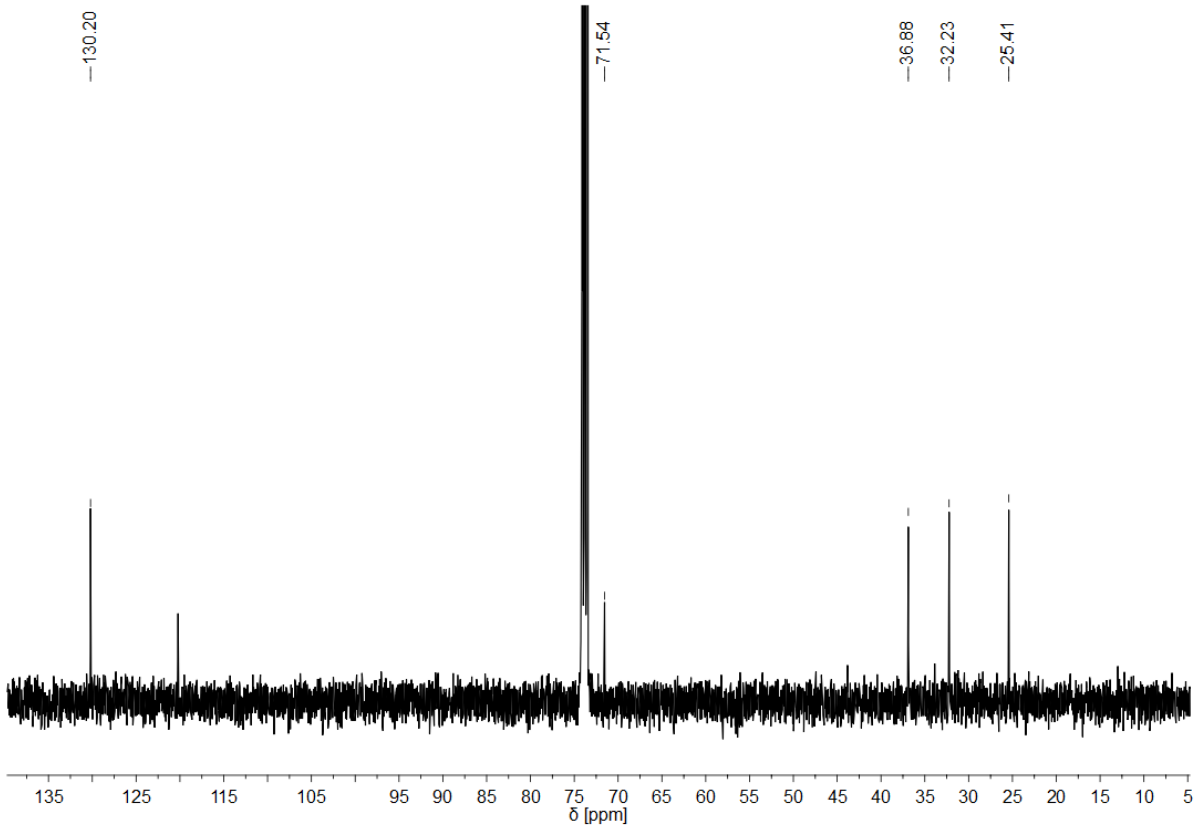

Figure S87. ^{13}C NMR (100 MHz, 100 °C, $C_2D_4Cl_2$) of poly(**M1**).

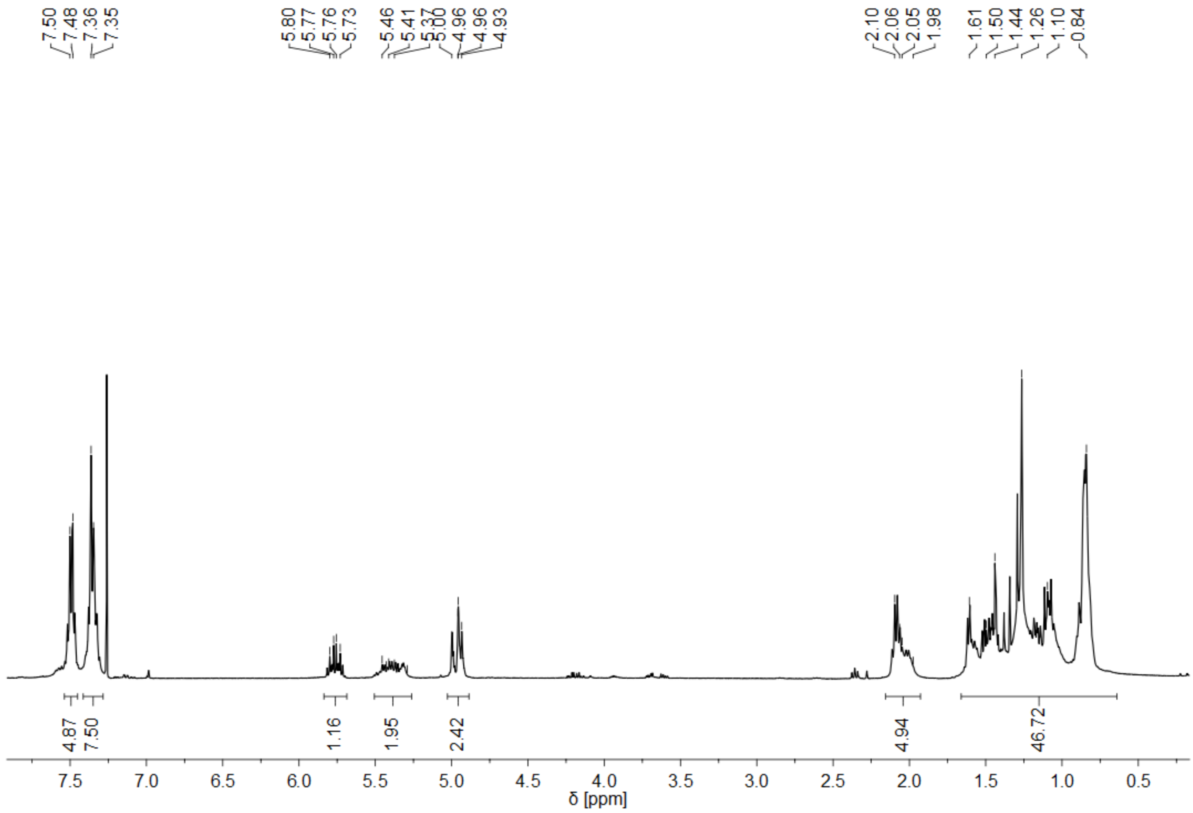

Figure S88. ^{1}H NMR (400 MHz, 25 °C, $CDCl_3$) of poly(**M2**).

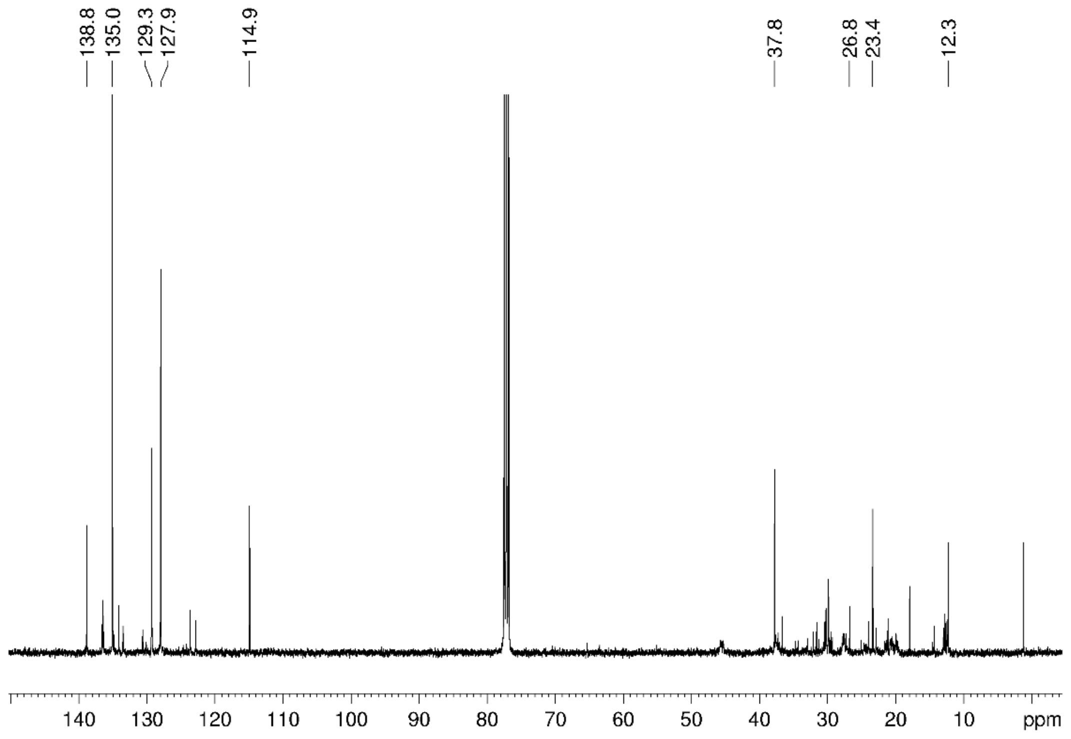

Figure S89. ^{13}C NMR (100 MHz, 25 °C, $CDCl_3$) of poly(**M2**).

NMR Spectra of Stability Experiments

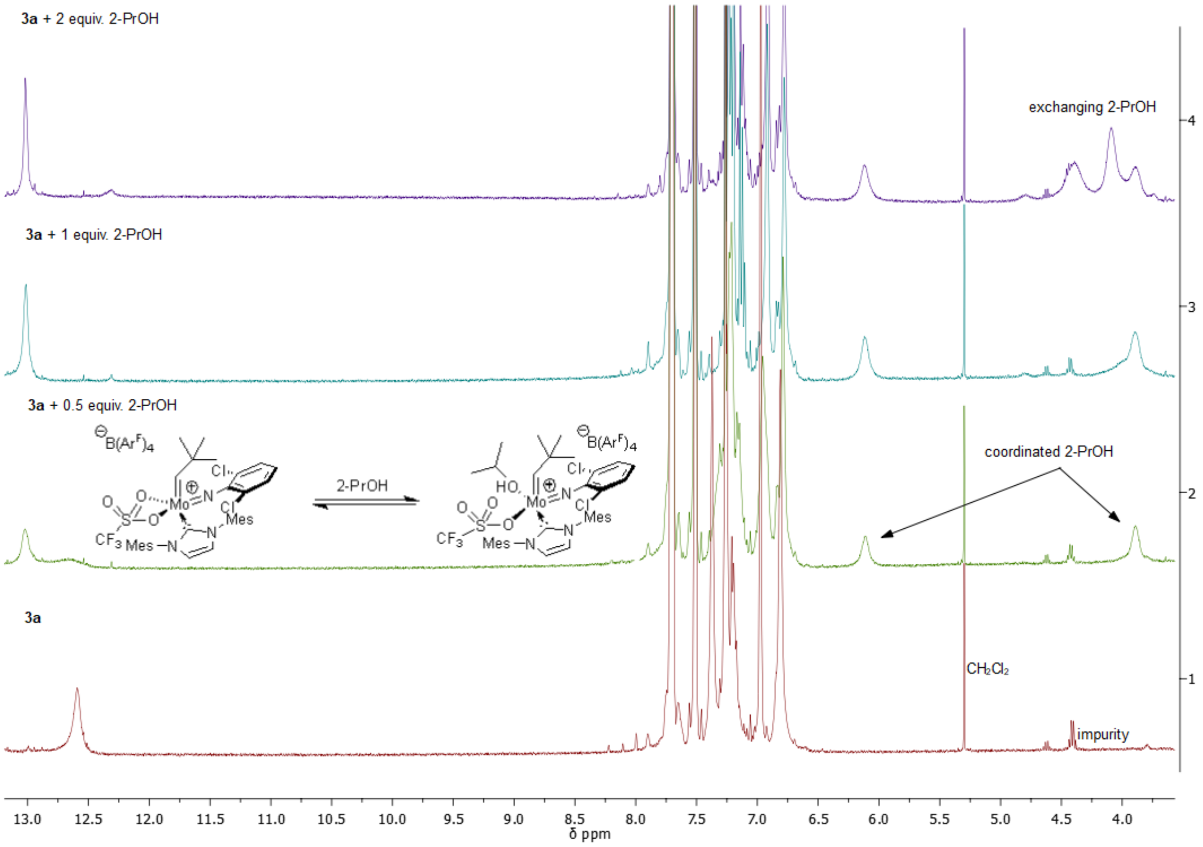

Figure S90. ^1H NMR (400 MHz, 25 °C, $CDCl_3$) of **3a** and **3a** in the presence of 2-PrOH.

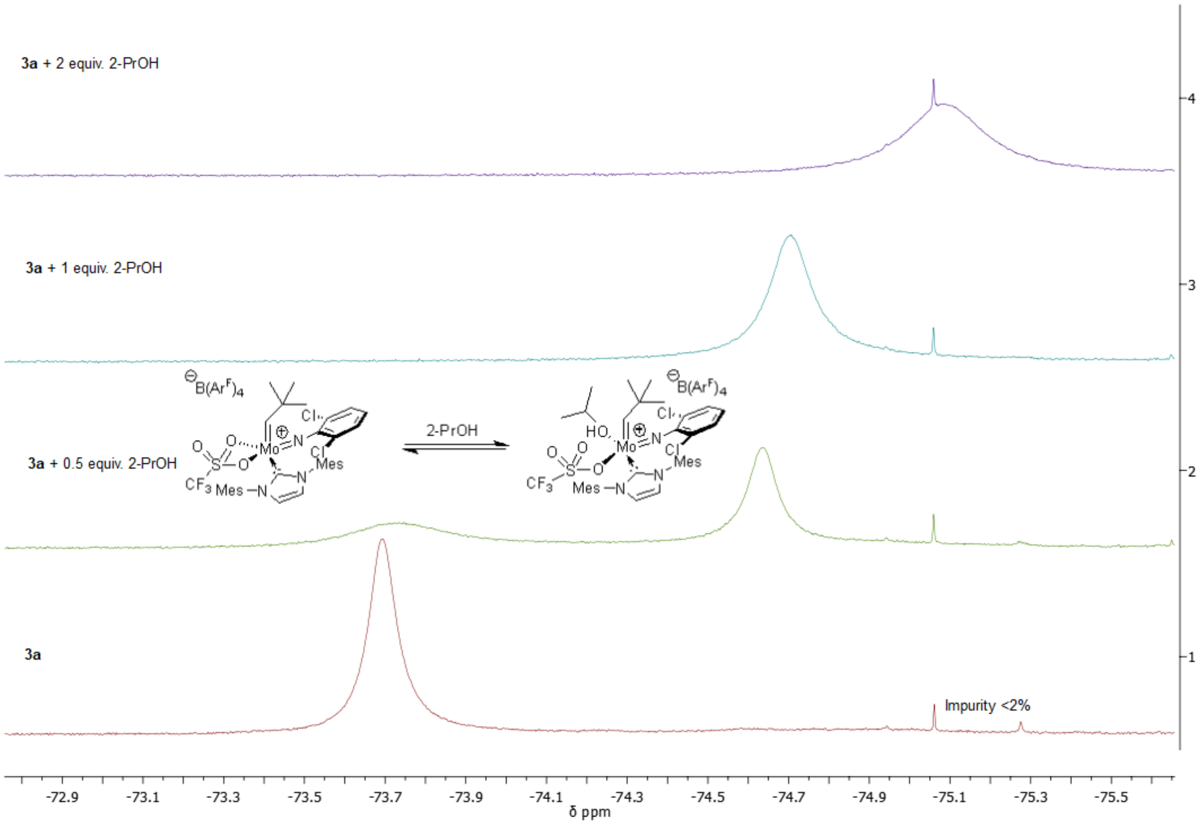

Figure S91. ^{19}F NMR (375 MHz, 25 °C, $CDCl_3$) of pure **3a** and **3a** in the presence of 2-PrOH.

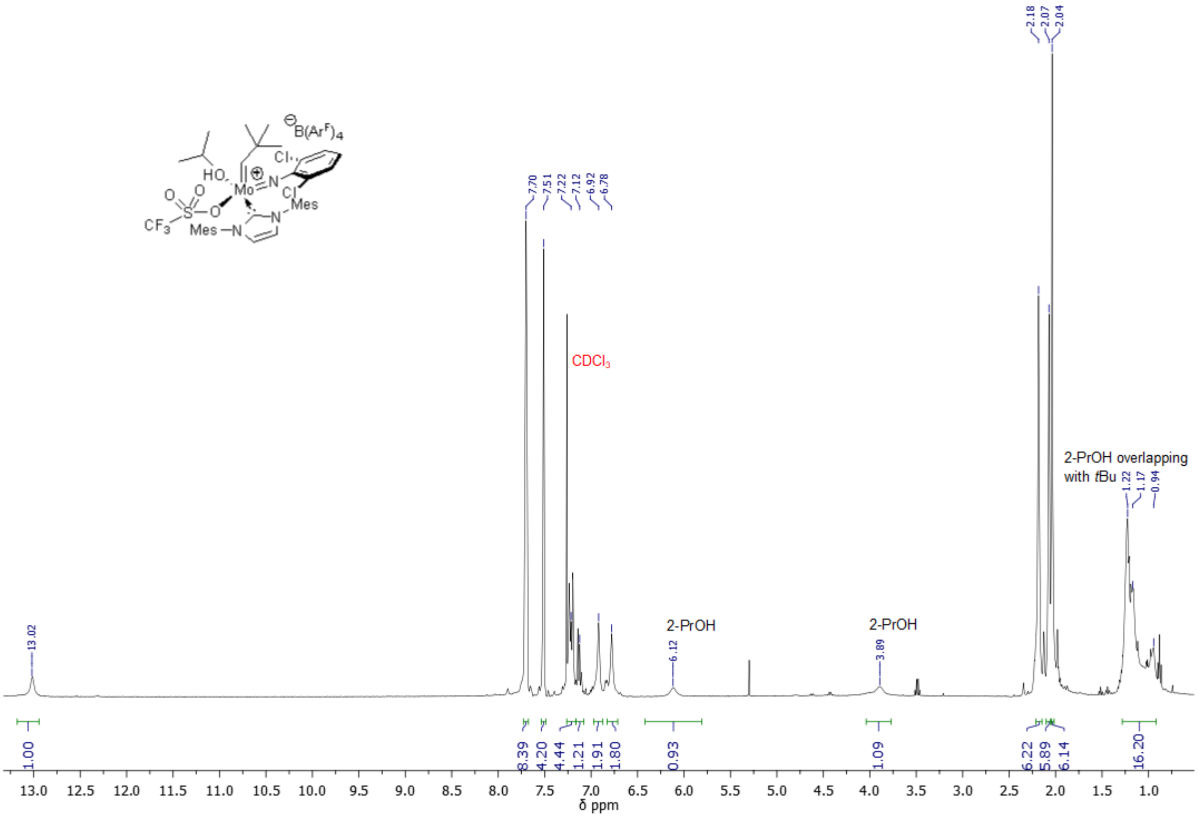

Figure S92. ^1H NMR (400 MHz, 25 °C, $CDCl_3$) **3a** in the presence of 1 equiv. 2-PrOH.

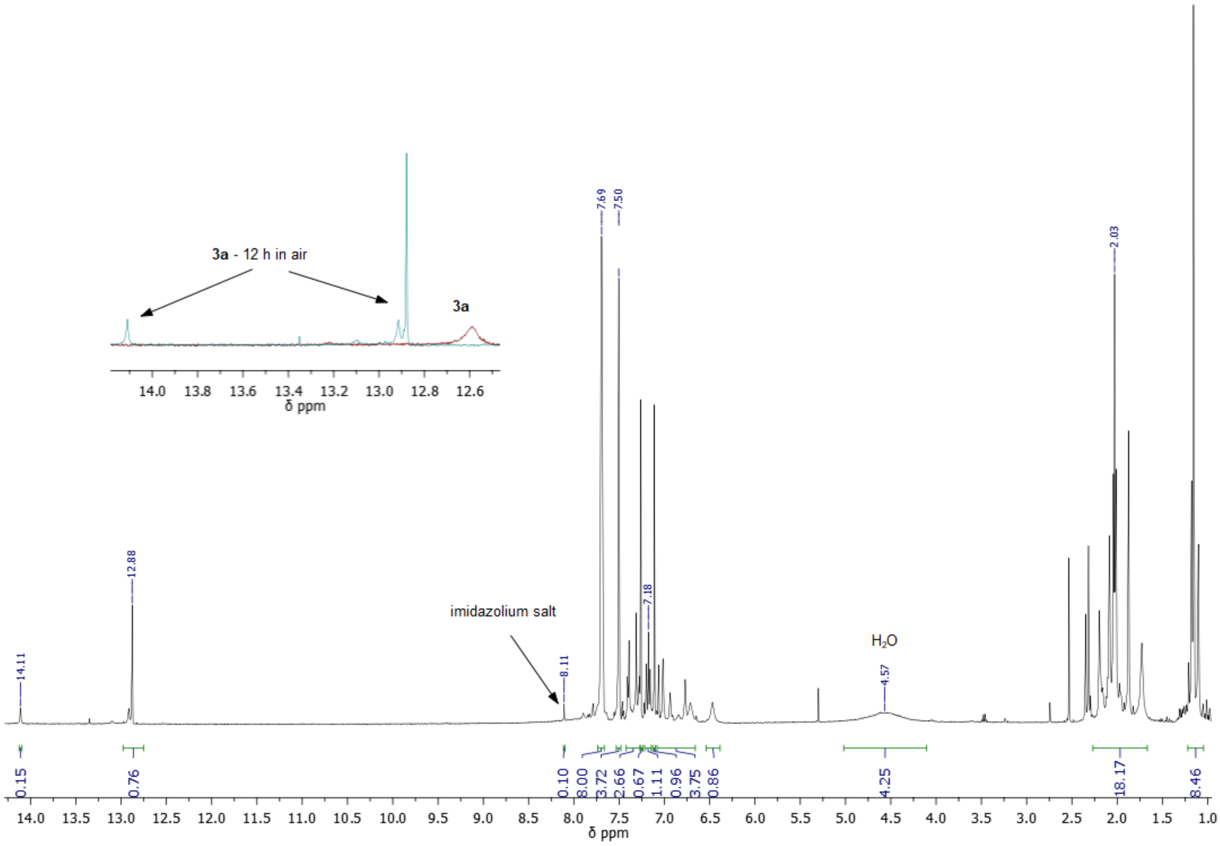

Figure S93. ^1H NMR (400 MHz, 25 °C, $CDCl_3$) of **3a** after 12 h in air containing 2 equiv. H_2O and superimposed spectra (expansion) of the alkylidene region of pure **3a** (red) and **3a** after 12 h in air (blue).

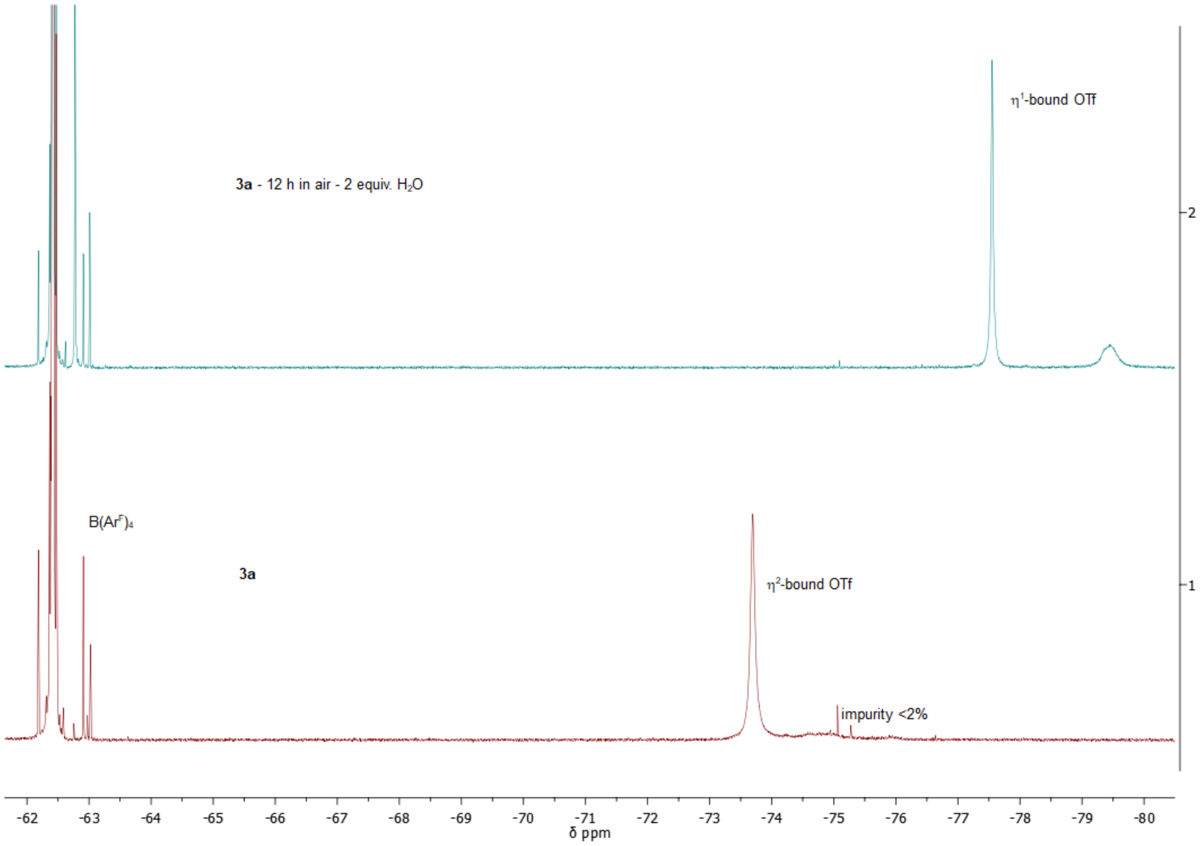

Figure S94. ^{19}F NMR (375 MHz, 25 °C, $CDCl_3$) of pure **3a** and **3a** after 12 h in air.

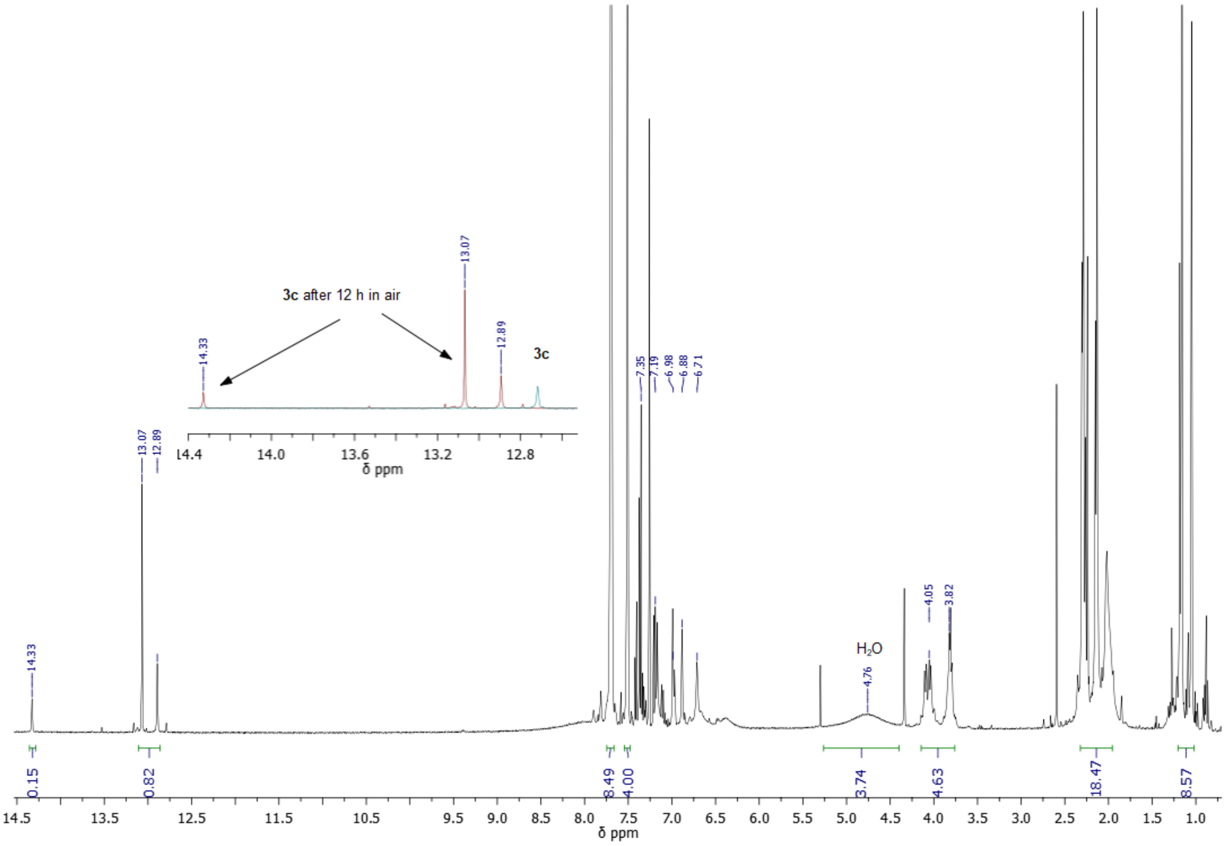

Figure S95. ^{1}H NMR (400 MHz, 25 °C, $CDCl_3$) of **3c** after 12 h in air containing 2 equiv. H_2O and superimposed spectra (expansion) of the alkylidene region of pure **3c** (blue) and **3a** after 12 h in air (red).

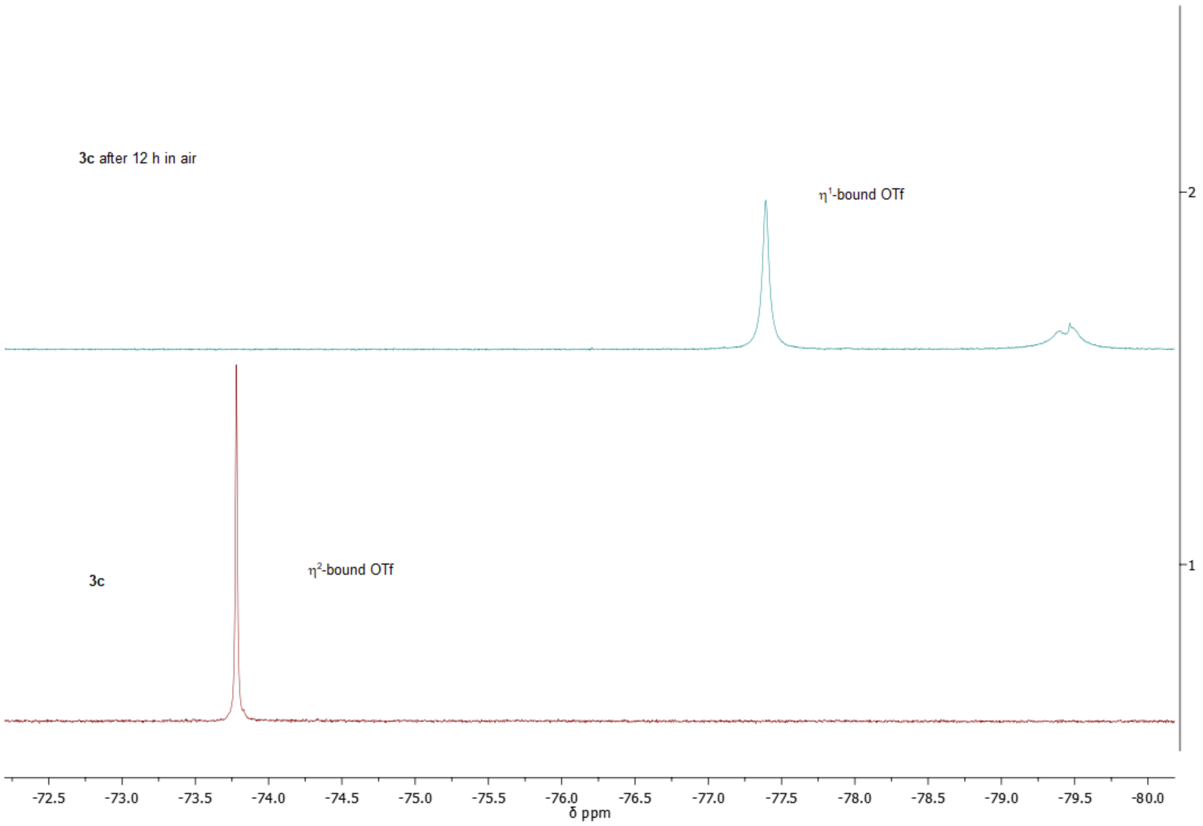

Figure S96. ^{19}F NMR (375 MHz, 25 °C, $CDCl_3$) of pure **3c** and **3c** after 12 h in air.

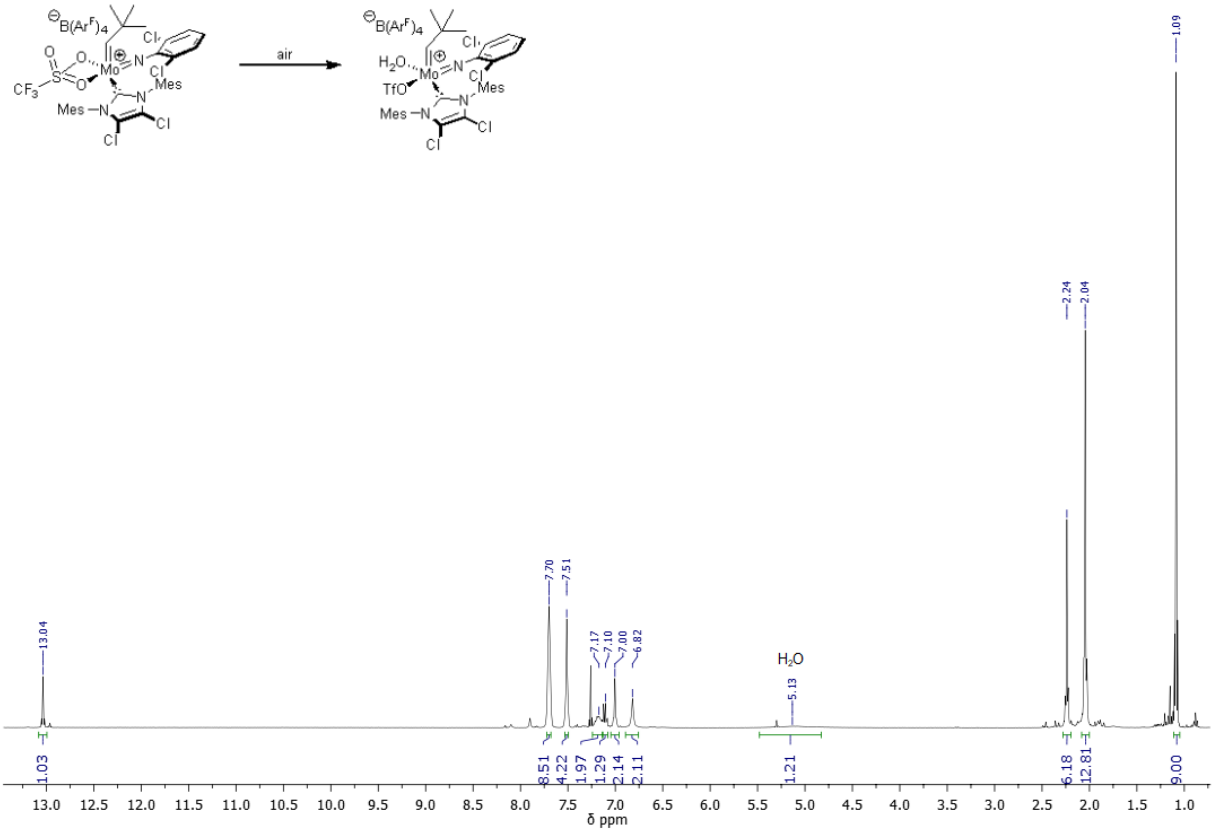

Figure S97. ^{1}H NMR (400 MHz, 25 °C, $CDCl_3$) of **3d** after 12 h in air.

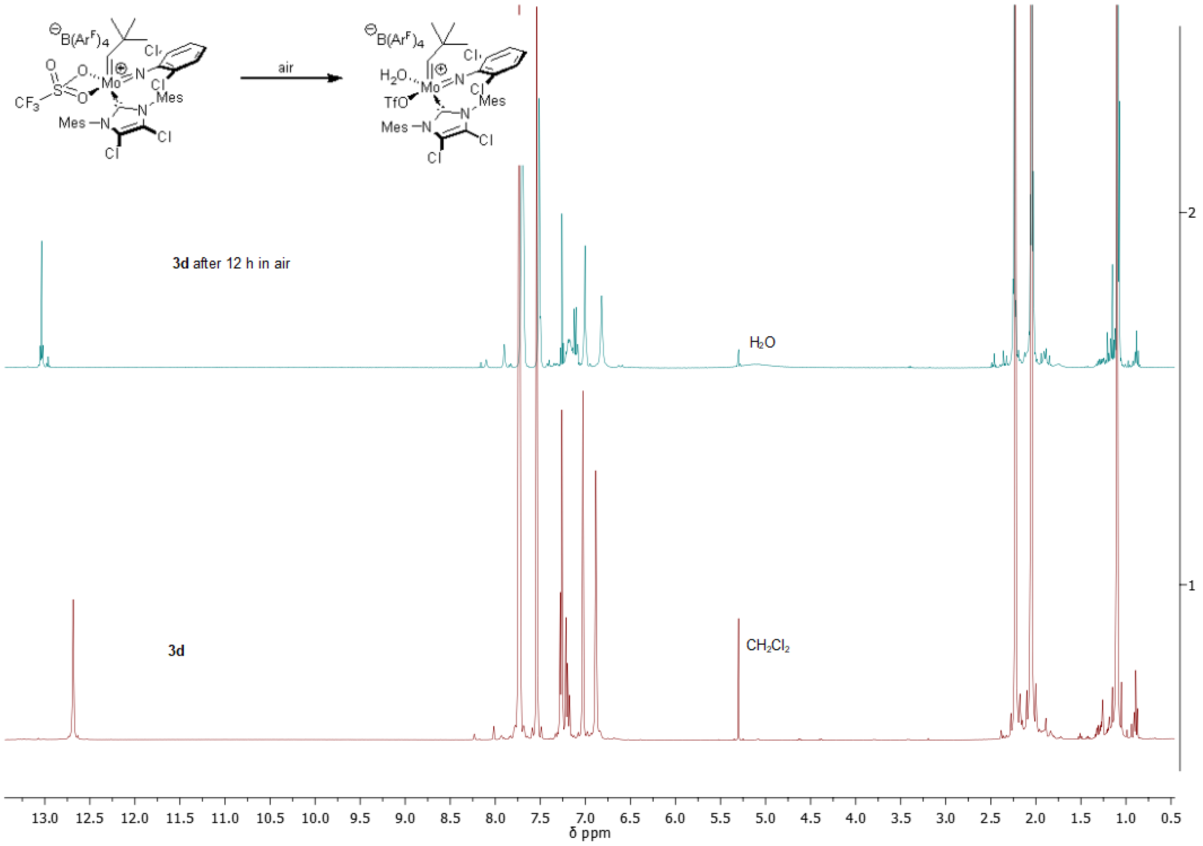

Figure S98. ^{1}H NMR (400 MHz, 25 °C, $CDCl_3$) of **3d** (bottom) and **3d** after 12 h in air (top).

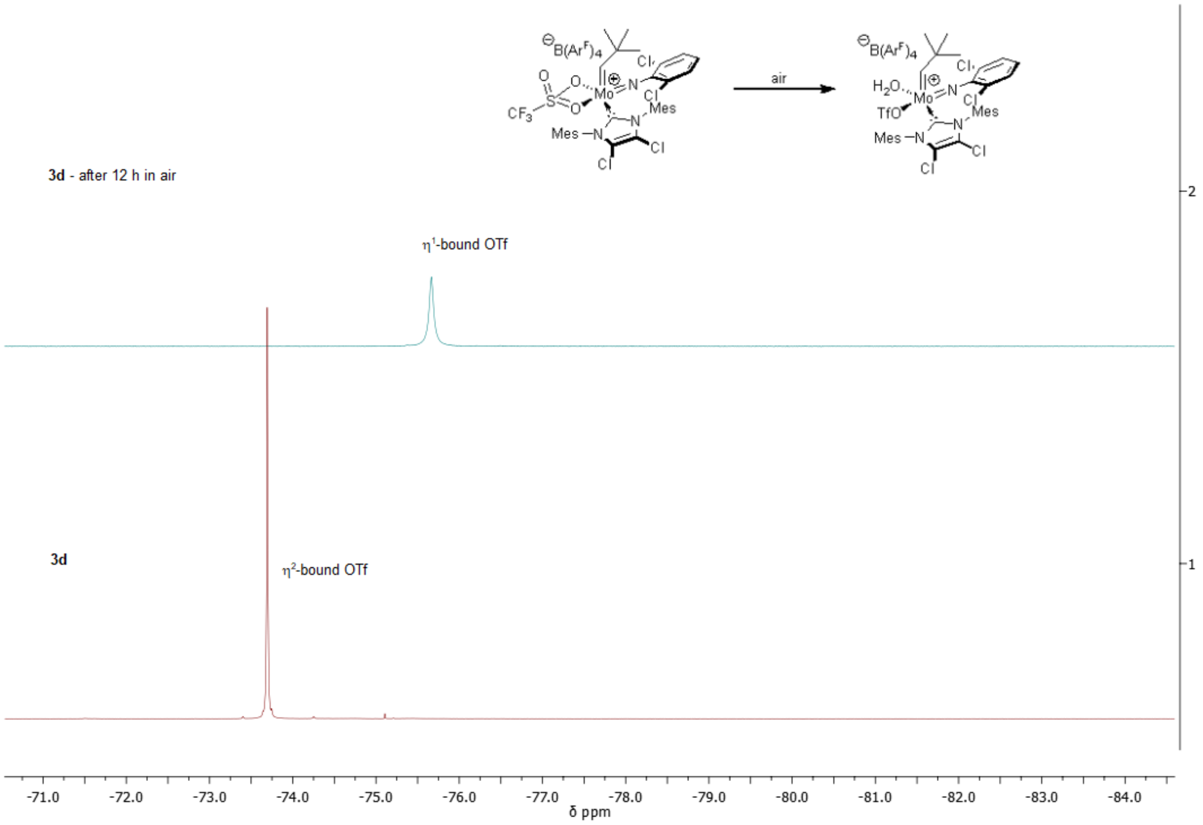

Figure S99. ^{19}F NMR (375 MHz, 25 °C, $CDCl_3$) of pure **3d** and **3d** after 12 h in air.

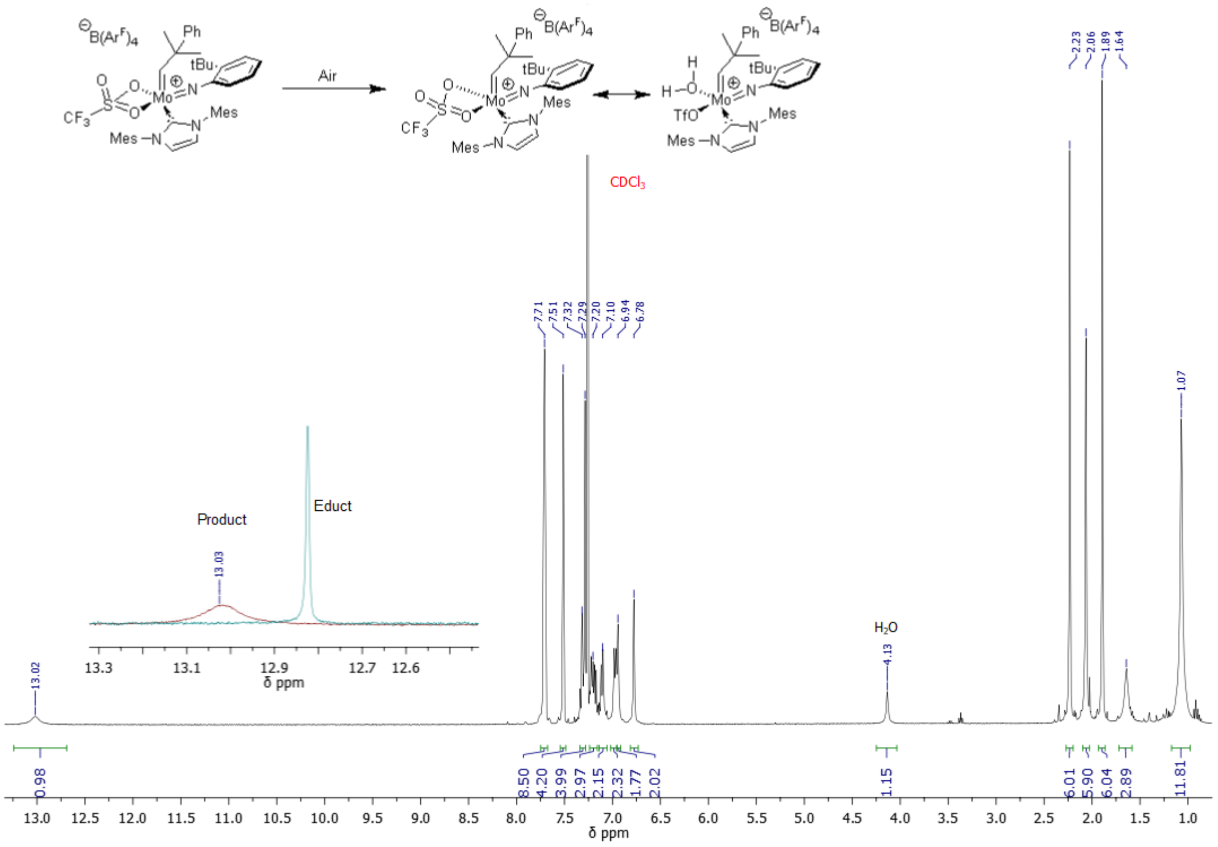

Figure S100. ^{1}H NMR (400 MHz, 25 °C, $CDCl_3$) of **4a** after 24 h in air containing 0.5 equiv. H_2O and superimposed spectra (expansion) of the alkylidene region of pure **4a** (blue) and **4a** with 0.5 equiv. H_2O (red).

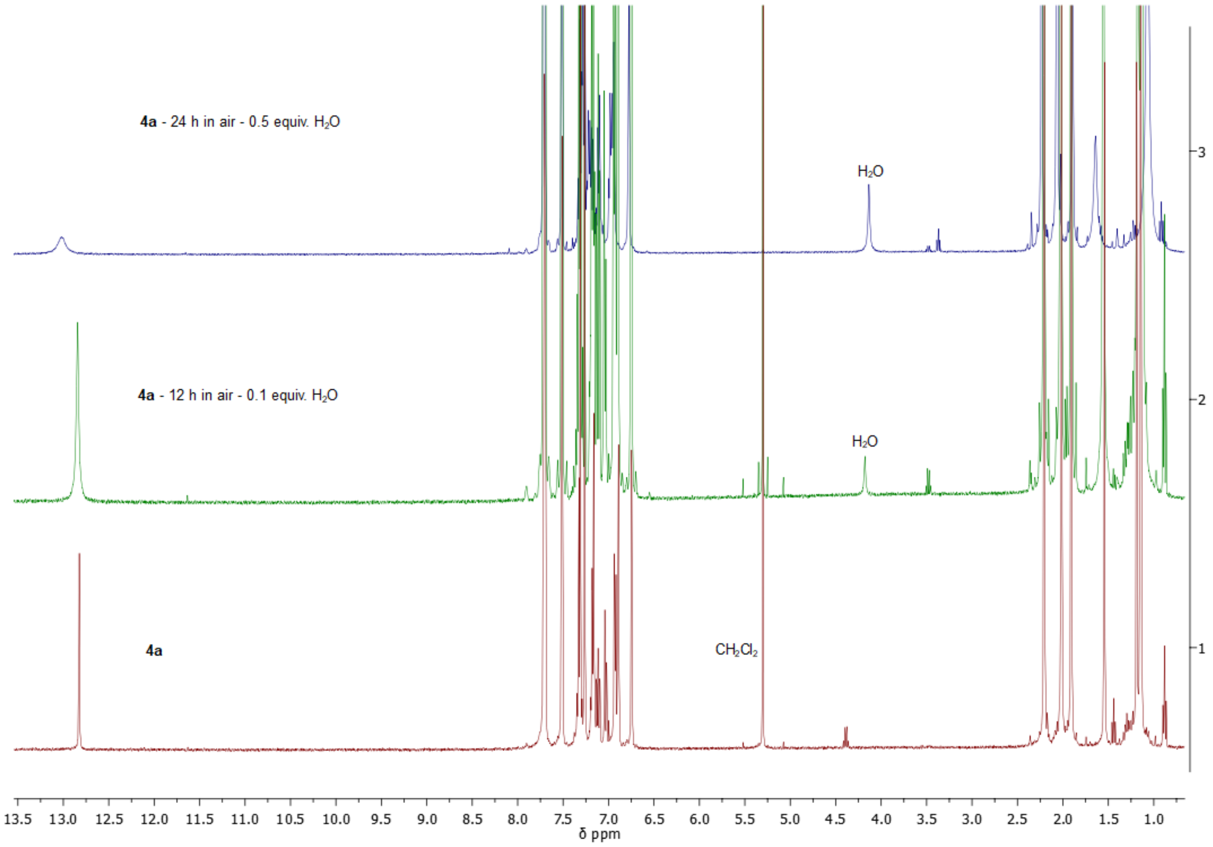

Figure S101. ^{1}H NMR (400 MHz, 25 °C, $CDCl_3$) of **4a** (bottom), **4a** after 12 (middle) and 24 h in air (top).

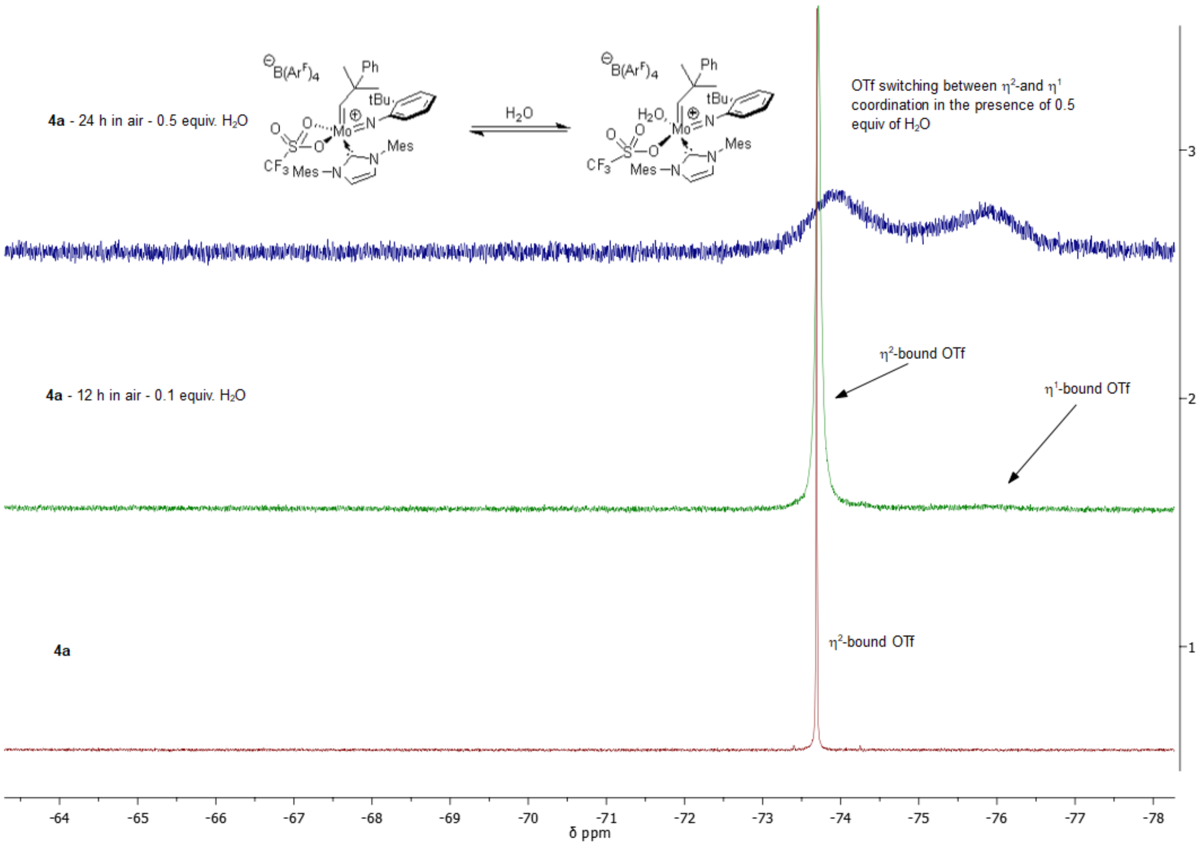

Figure S102. ^{19}F NMR (375 MHz, 25 °C, $CDCl_3$) of **4a** (bottom), **4a** after 12 (middle) and 24 h in air (top).

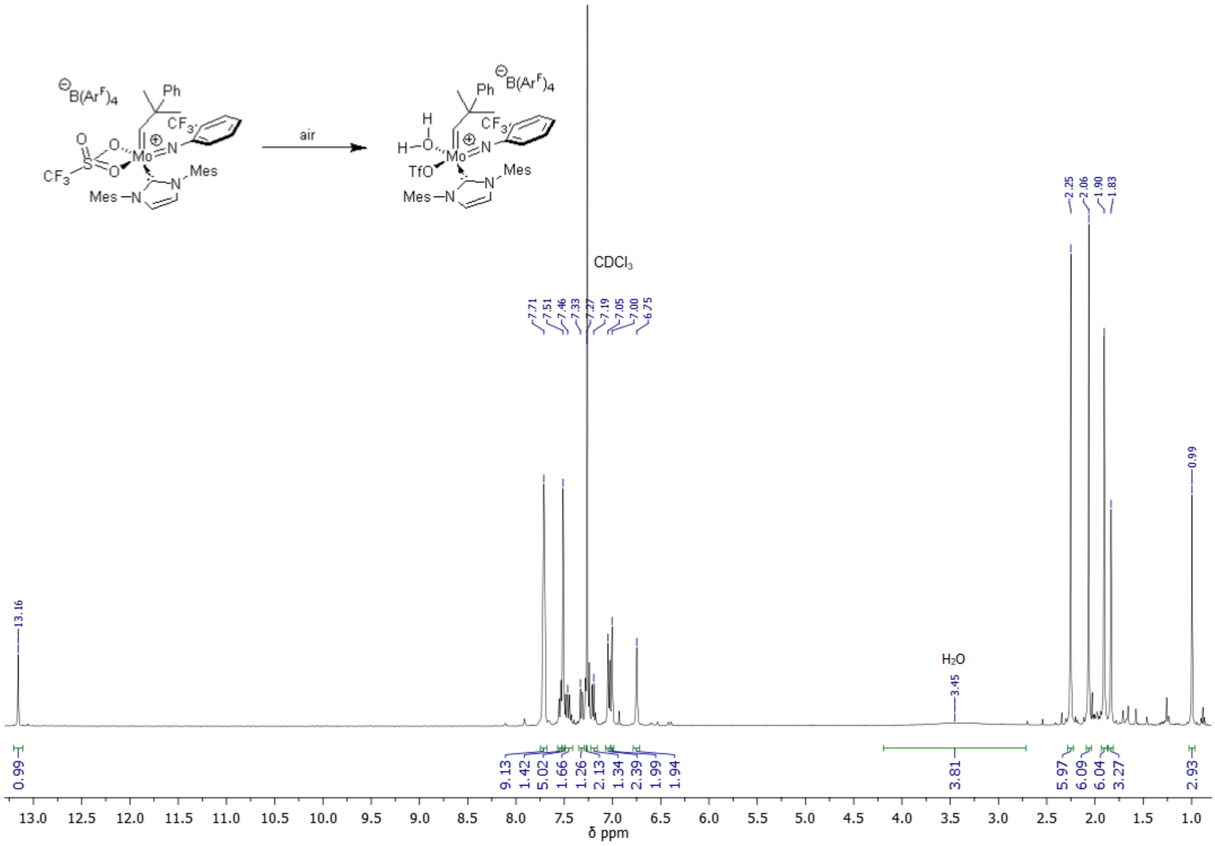

Figure S103. ^{1}H NMR (400 MHz, 25 °C, $CDCl_3$) of **4b** after 12 h in air containing ~1.5 - 2 equiv. H_2O.

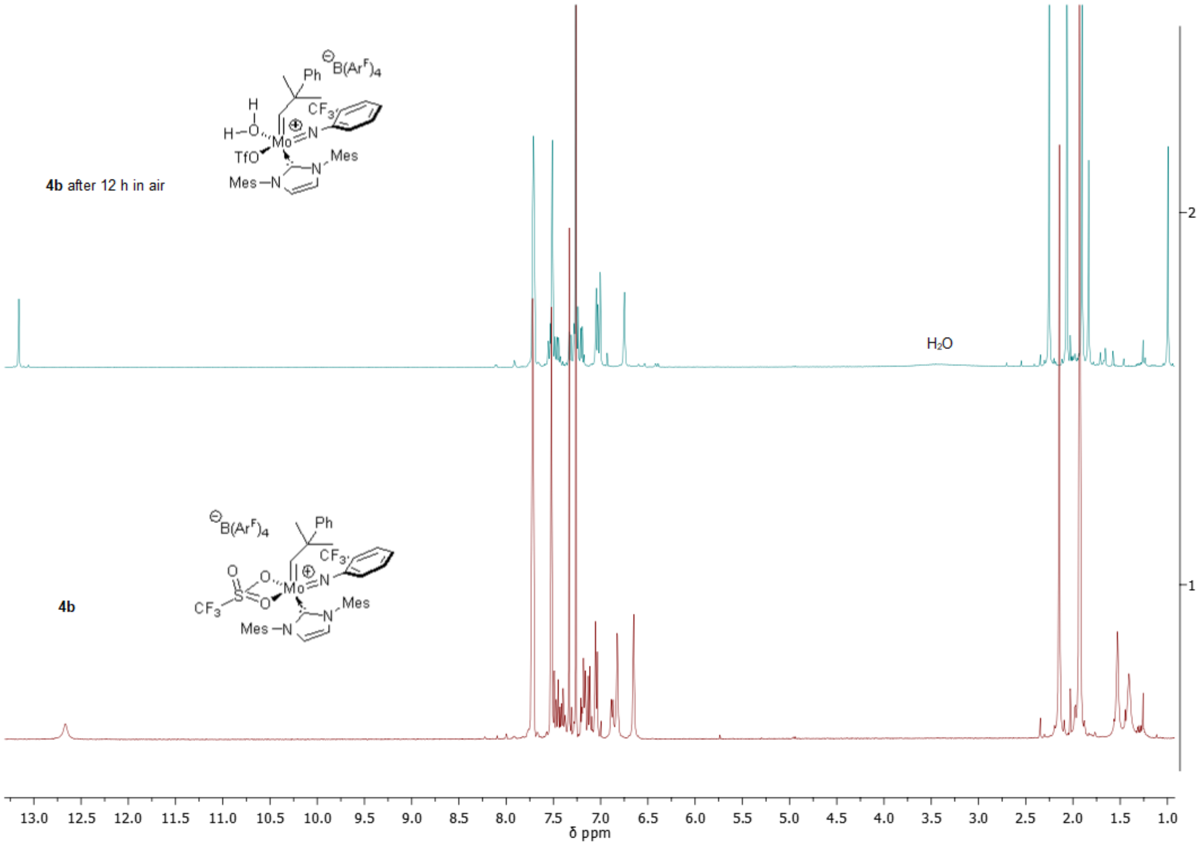

Figure S104. ^{1}H NMR (400 MHz, 25 °C, $CDCl_3$) of pure **4b** (bottom) and **4b** after 12 h in air (top).

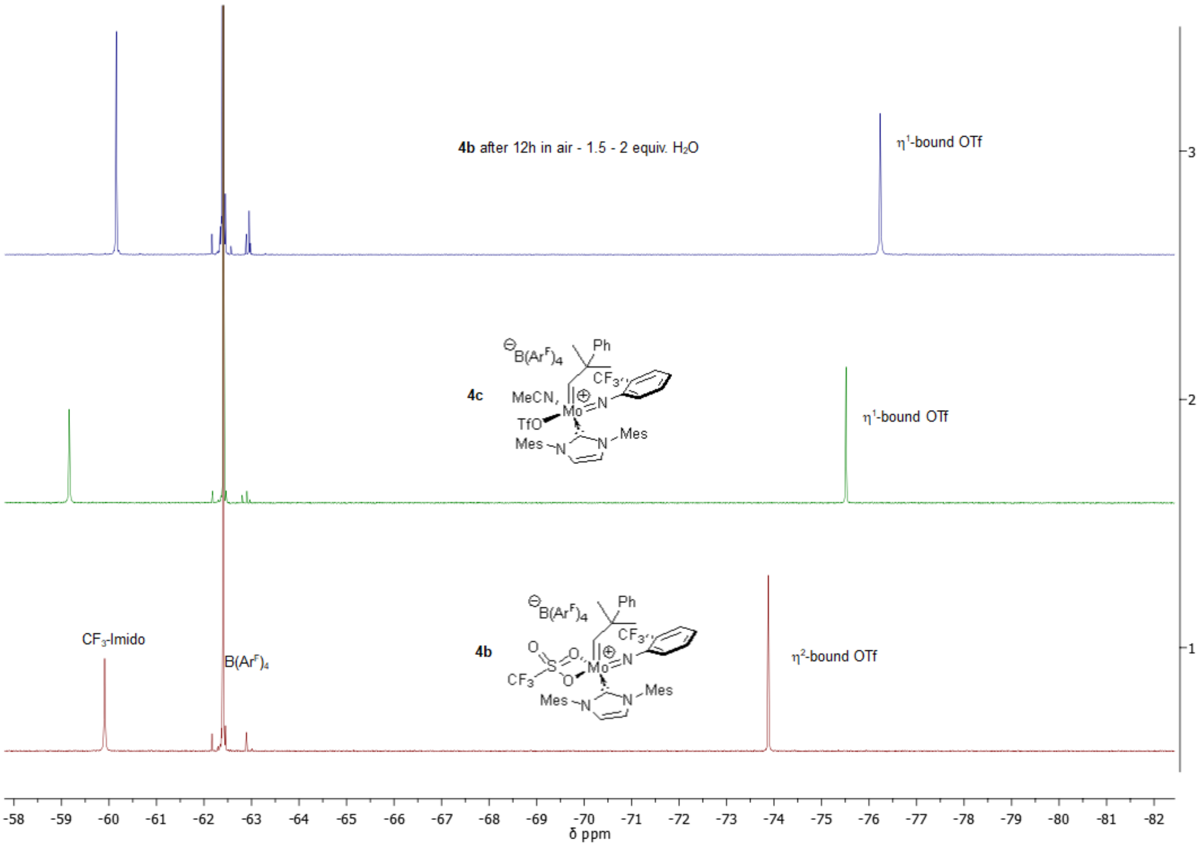

Figure S105. ^{19}F NMR (375 MHz, 25 °C, $CDCl_3$) of pure **4b** (bottom), **4c** (middle) and **4b** after 12 h in air (top).

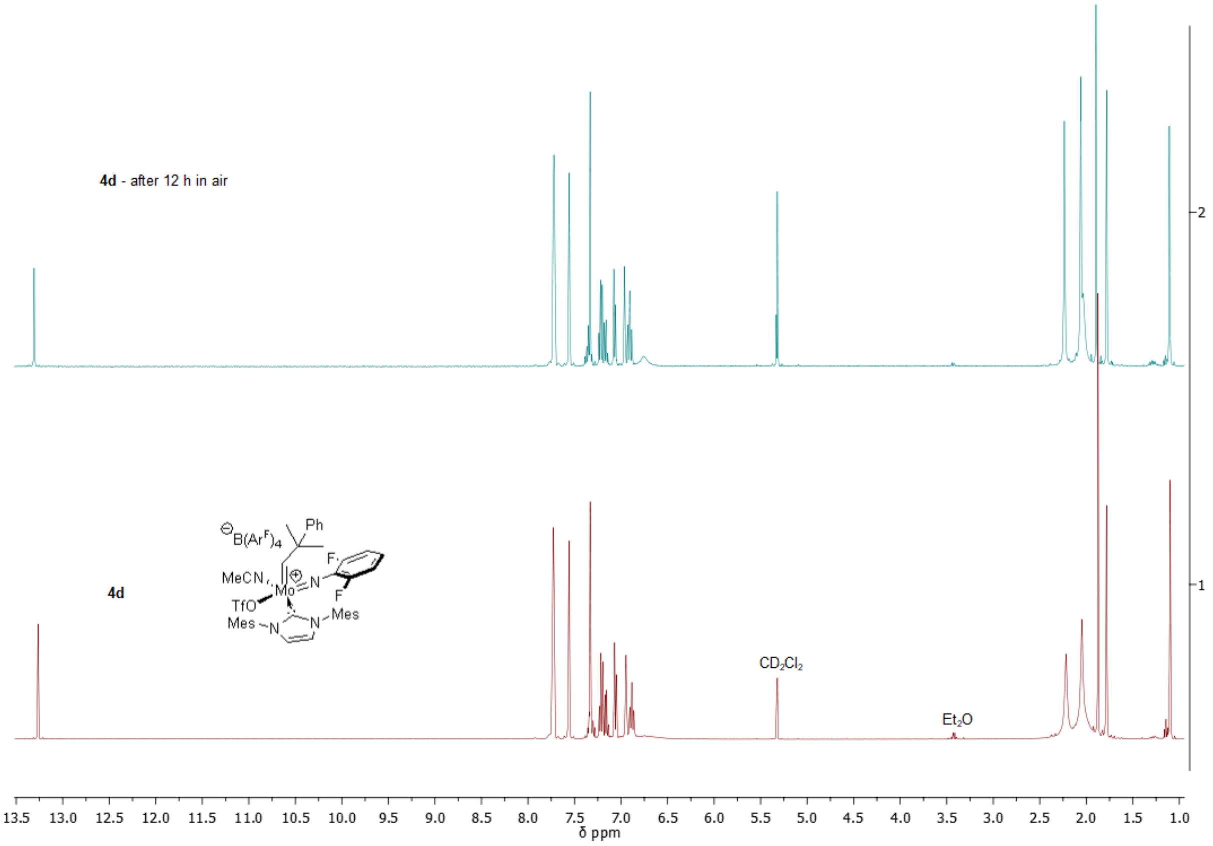

Figure S106. ^{1}H NMR (400 MHz, 25 °C, CD_2Cl_2) of pure **4d** (bottom) and **4d** after 12 h in air (top).

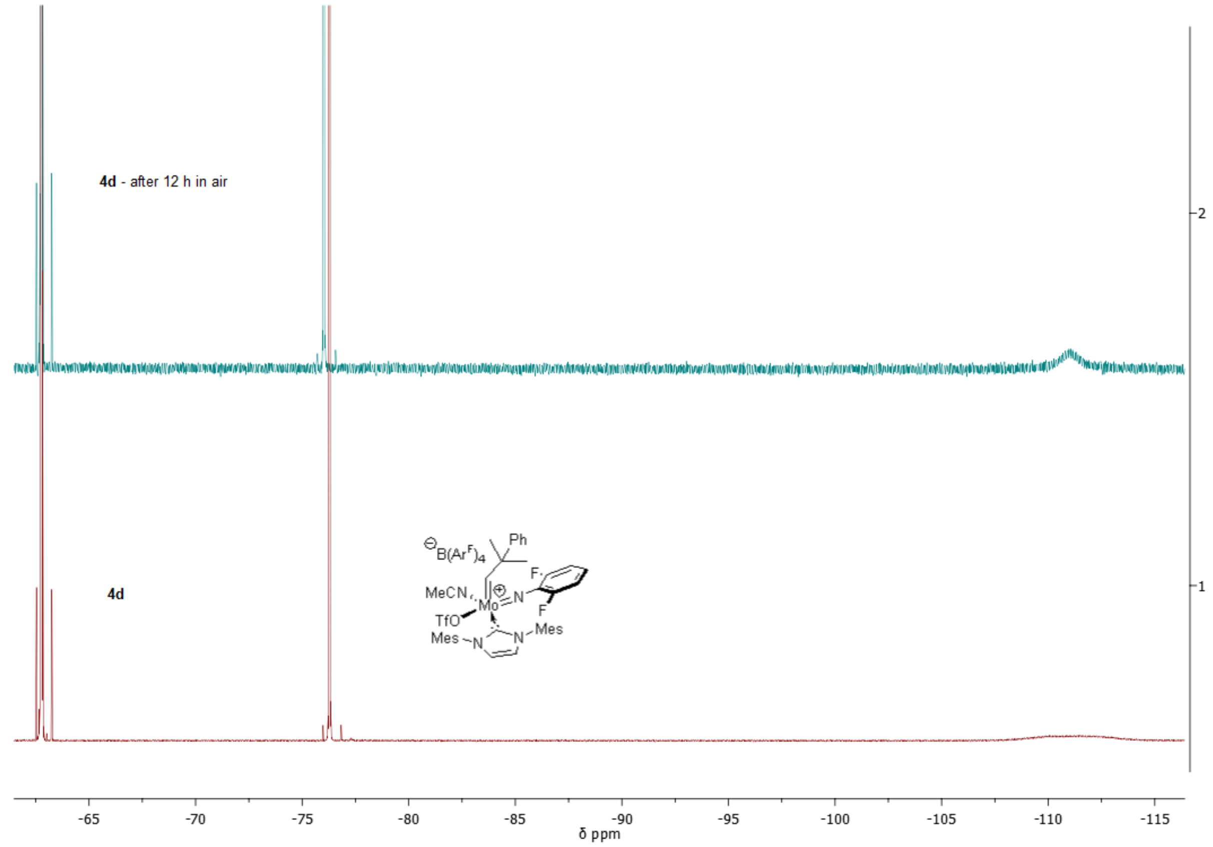

Figure S107. ^{19}F NMR (375 MHz, 25 °C, CD_2Cl_2) of pure **4d** (bottom) and **4d** after 12 h in air (top).

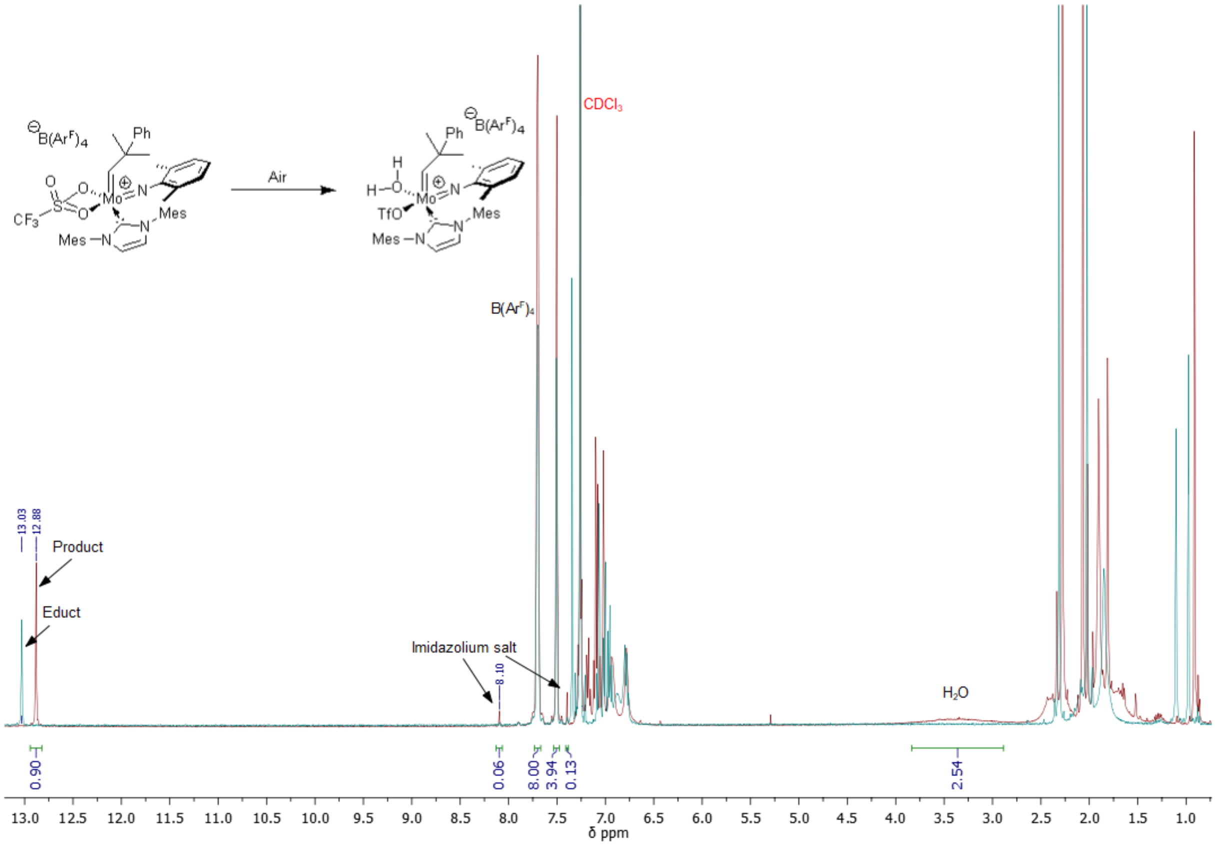

Figure S108. ^{1}H NMR (400 MHz, 25 °C, $CDCl_3$) superimposed spectra of pure **5a** (blue) and **5a** after 24 h in air (red).

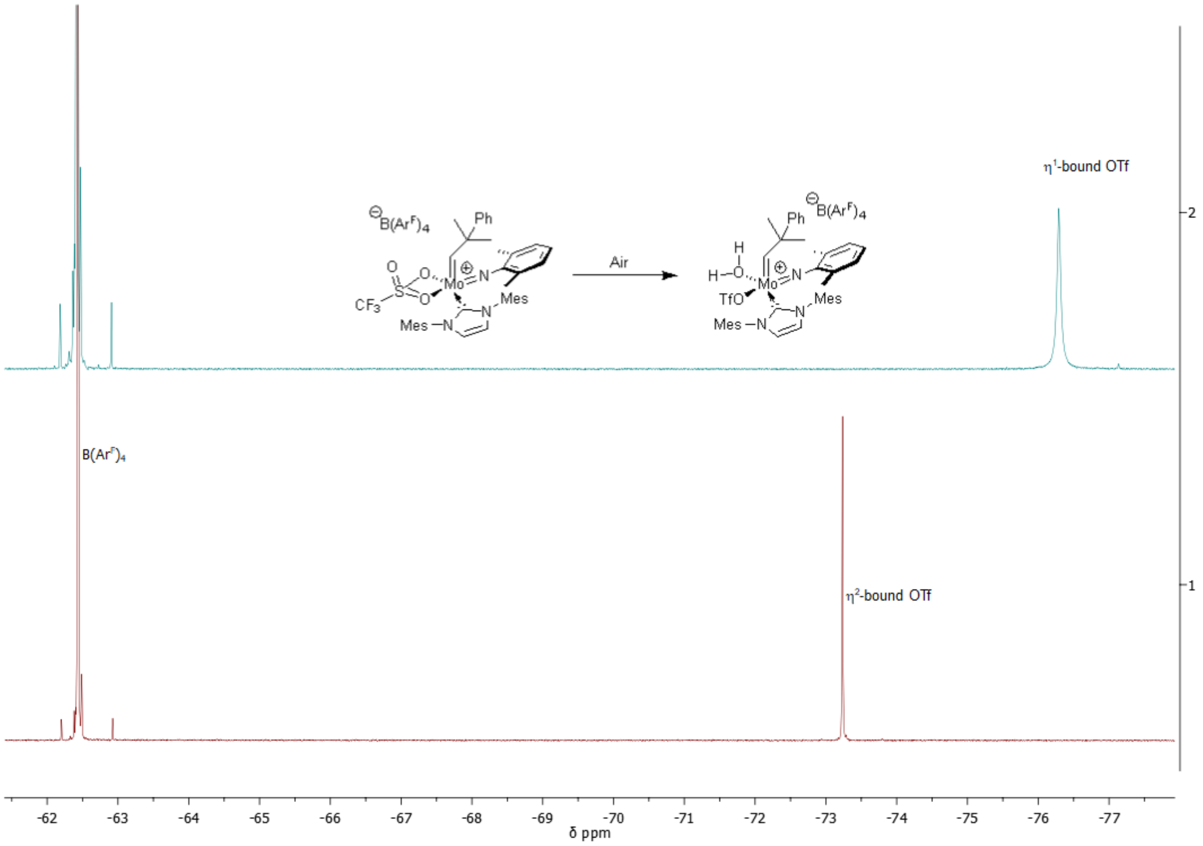

Figure S109. ^{19}F NMR (375 MHz, 25 °C, $CDCl_3$) of pure **5a** (red, bottom) and after 24 h in air (blue, top).

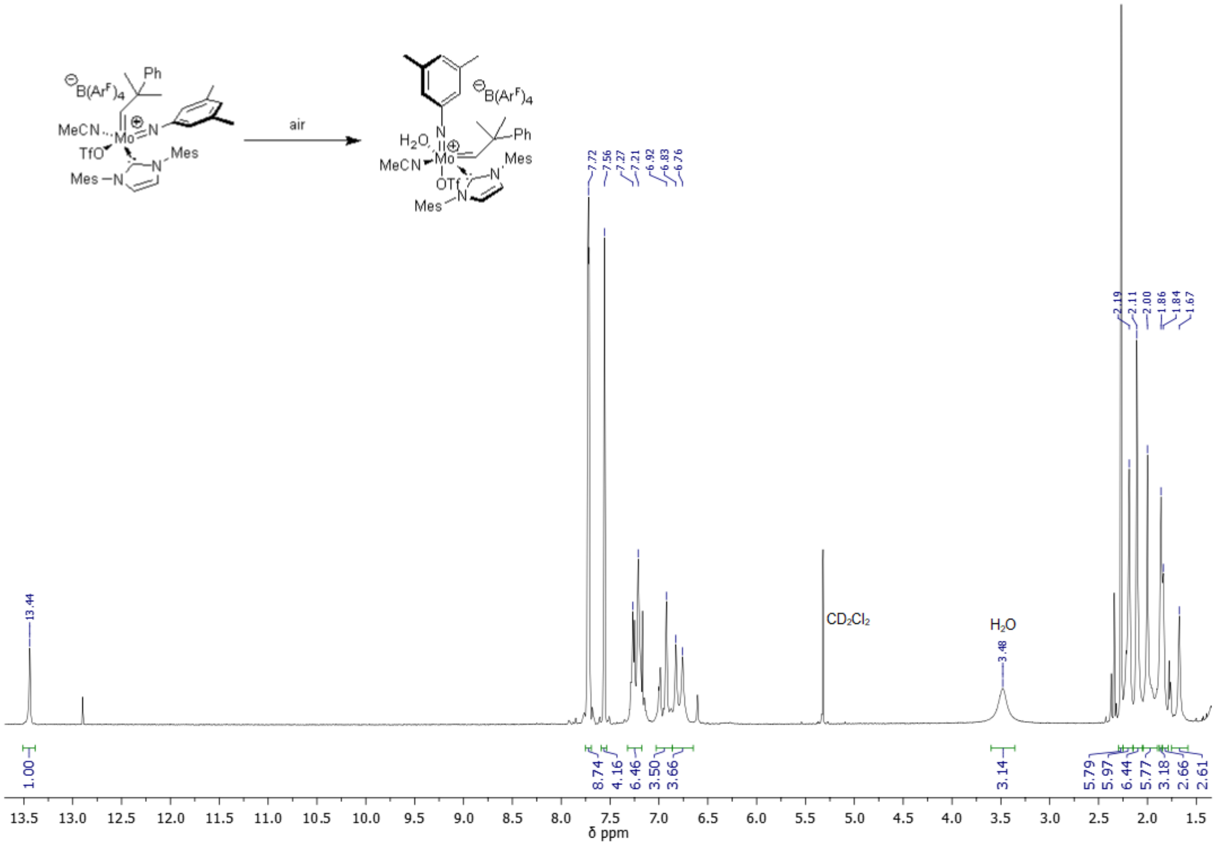

Figure S110. ^{1}H NMR (400 MHz, 25 °C, CD_2Cl_2) of **7** after 12 h in air.

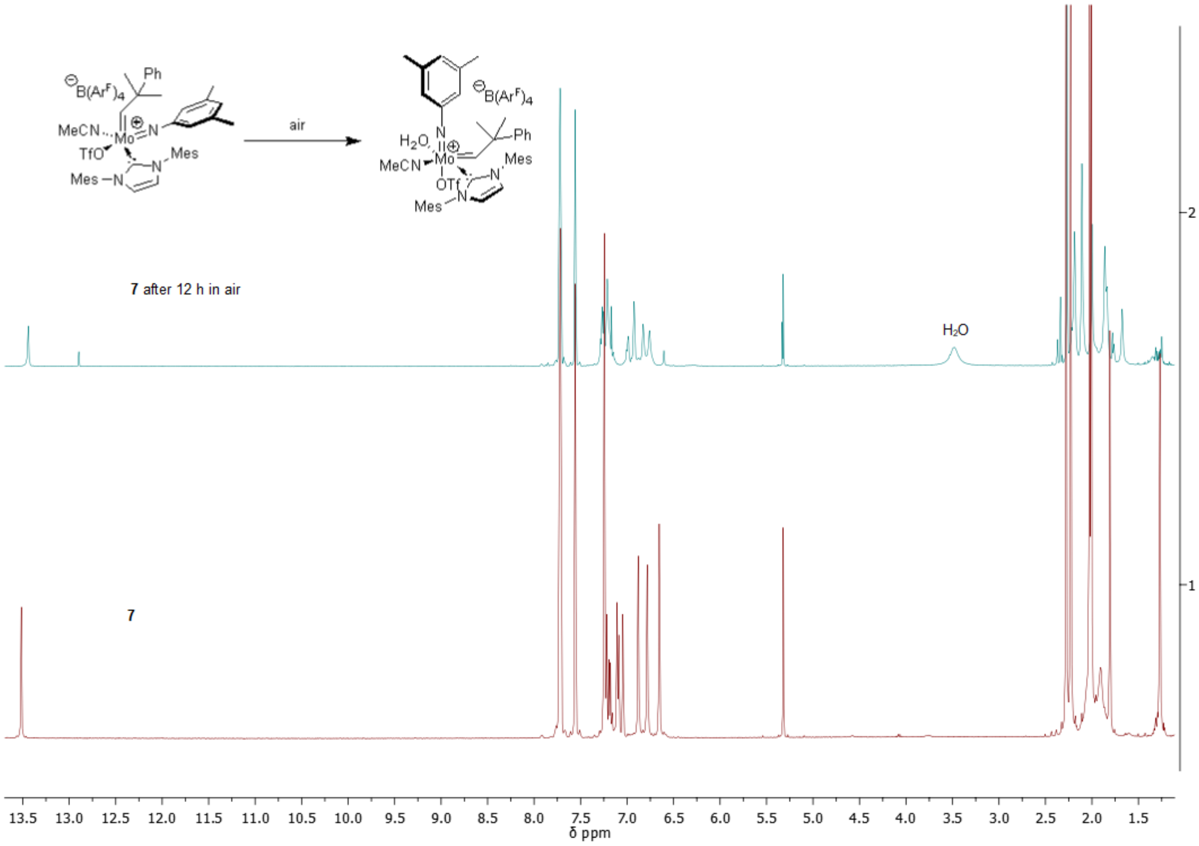

Figure S111. ^{1}H NMR (400 MHz, 25 °C, CD_2Cl_2) of pure **7** (bottom) and **7** after 12 h in air (top).

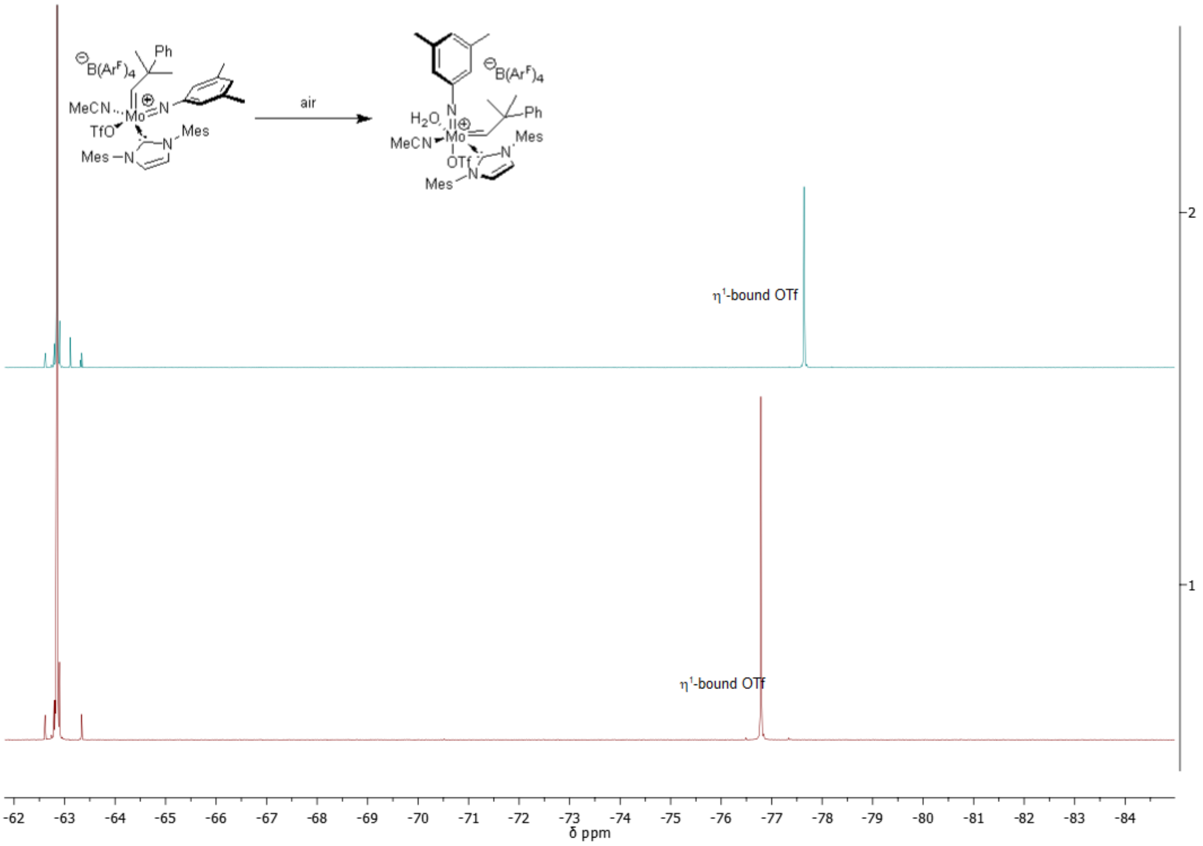

Figure S112. ^{19}F NMR (375 MHz, 25 °C, CD_2Cl_2) of pure **7** (bottom) and after 12 h in air (top).

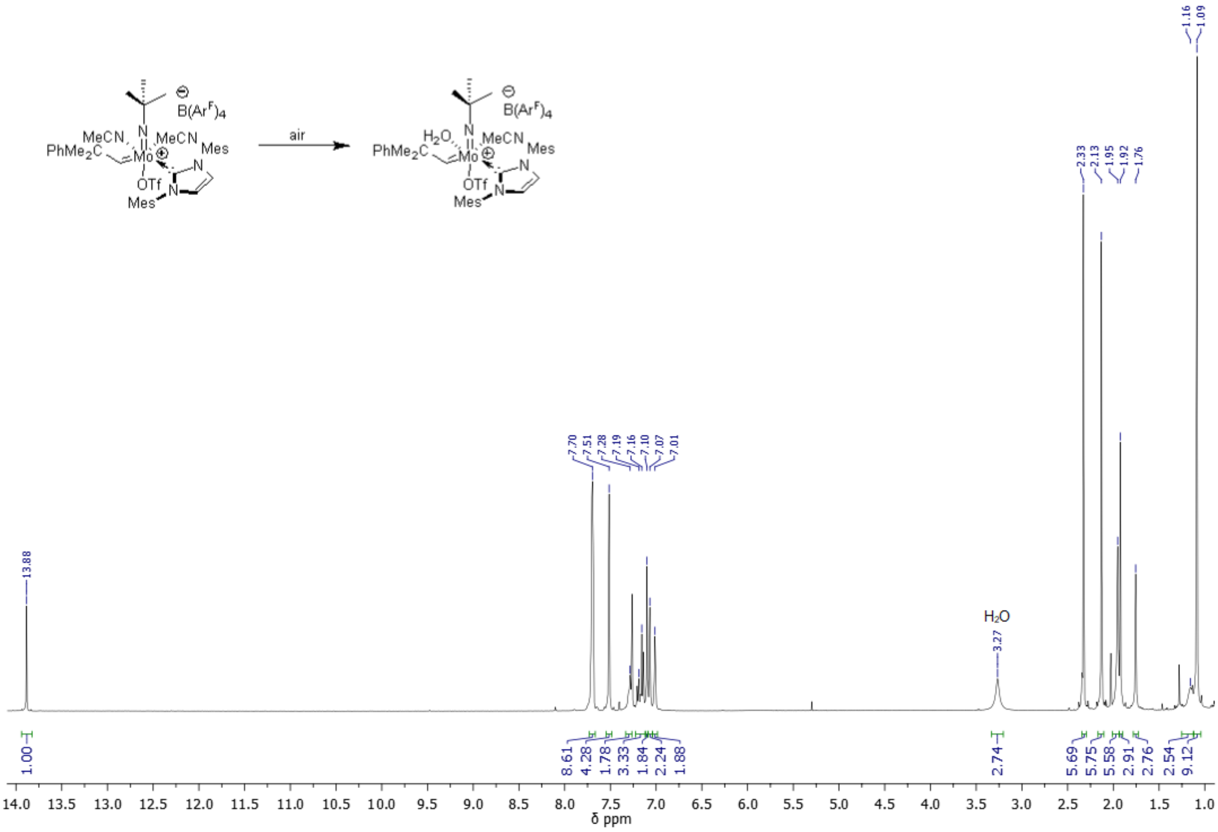

Figure S113. ^{1}H NMR (400 MHz, 25 °C, $CDCl_3$) of **8** after 12 h in air.

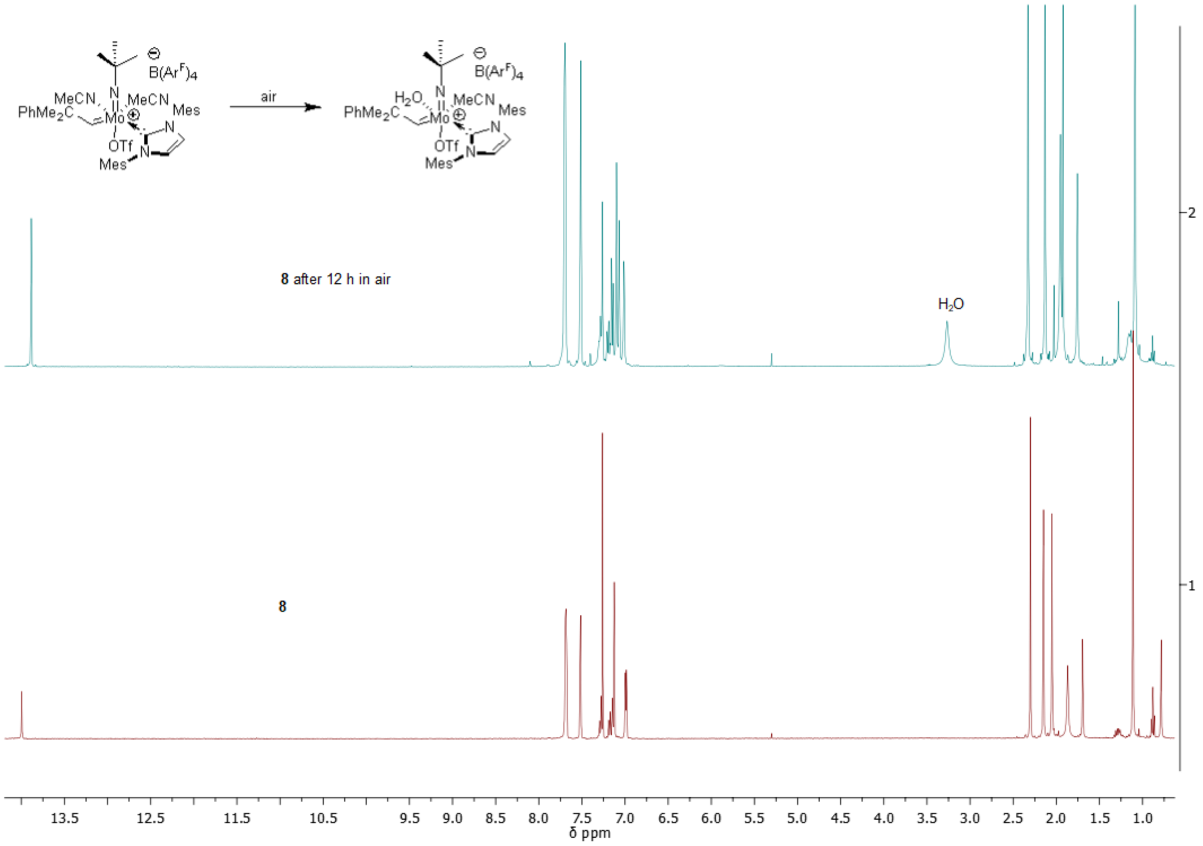

Figure S114. ^{1}H NMR (400 MHz, 25 °C, $CDCl_3$) of pure **8** and **8** after 12 h in air.

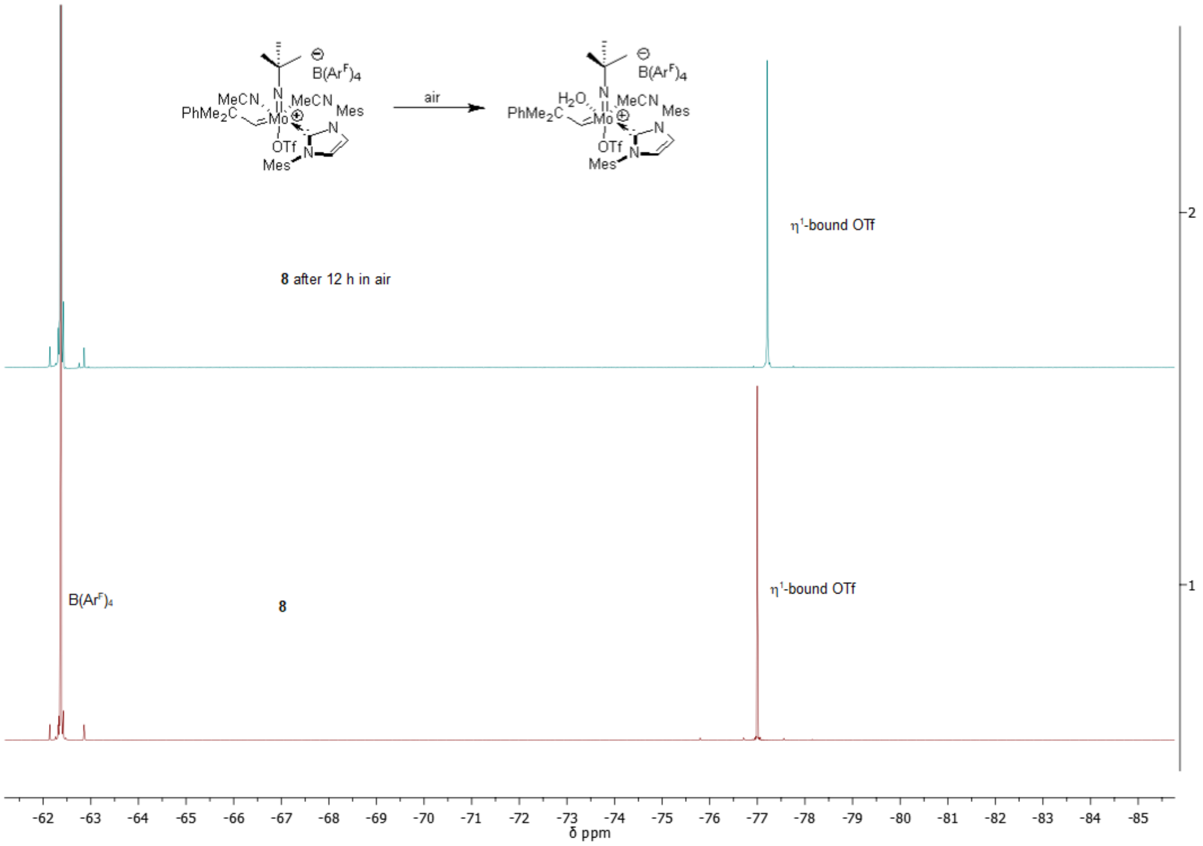

Figure S115. ^{19}F NMR (375 MHz, 25 °C, $CDCl_3$) of pure **8** (bottom) and after 12 h in air (top).

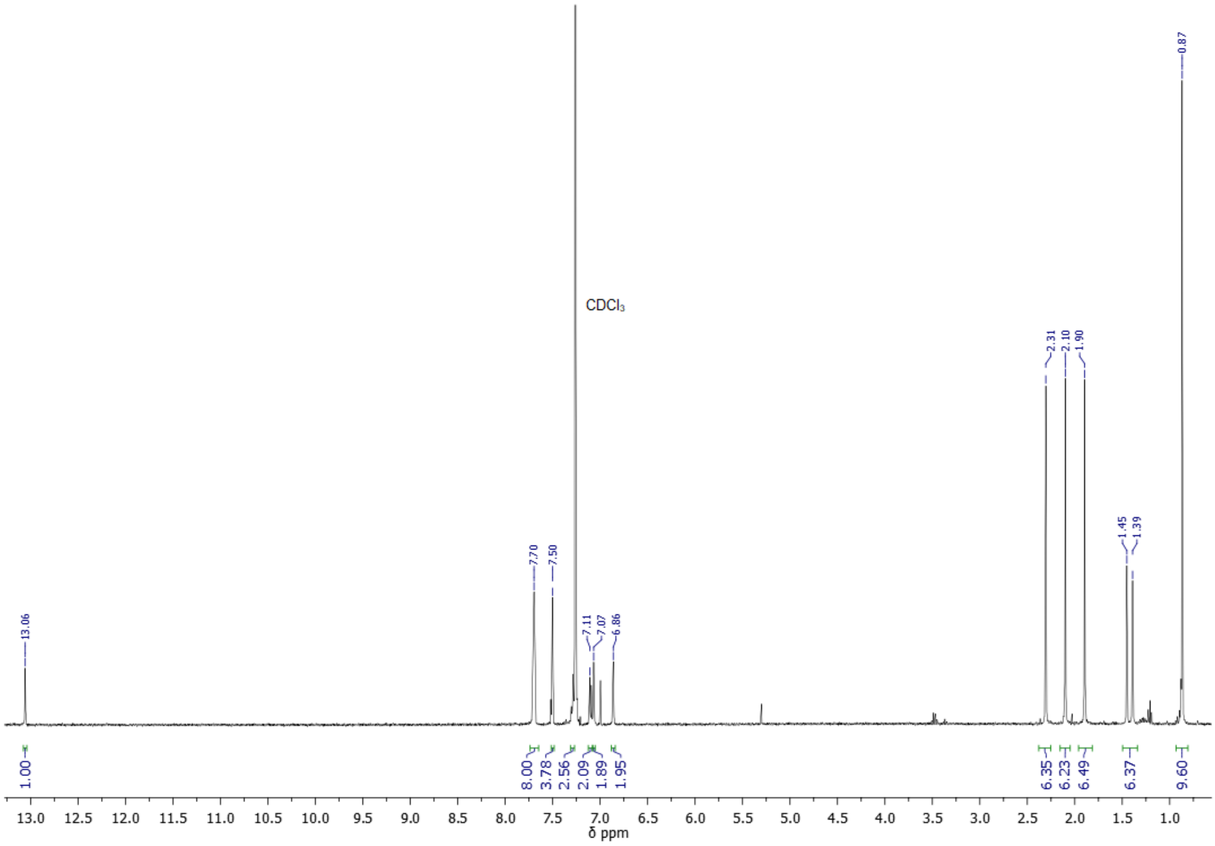

Figure S116. ^{1}H NMR (400 MHz, 25 °C, $CDCl_3$) of **11** after 5 days in air.

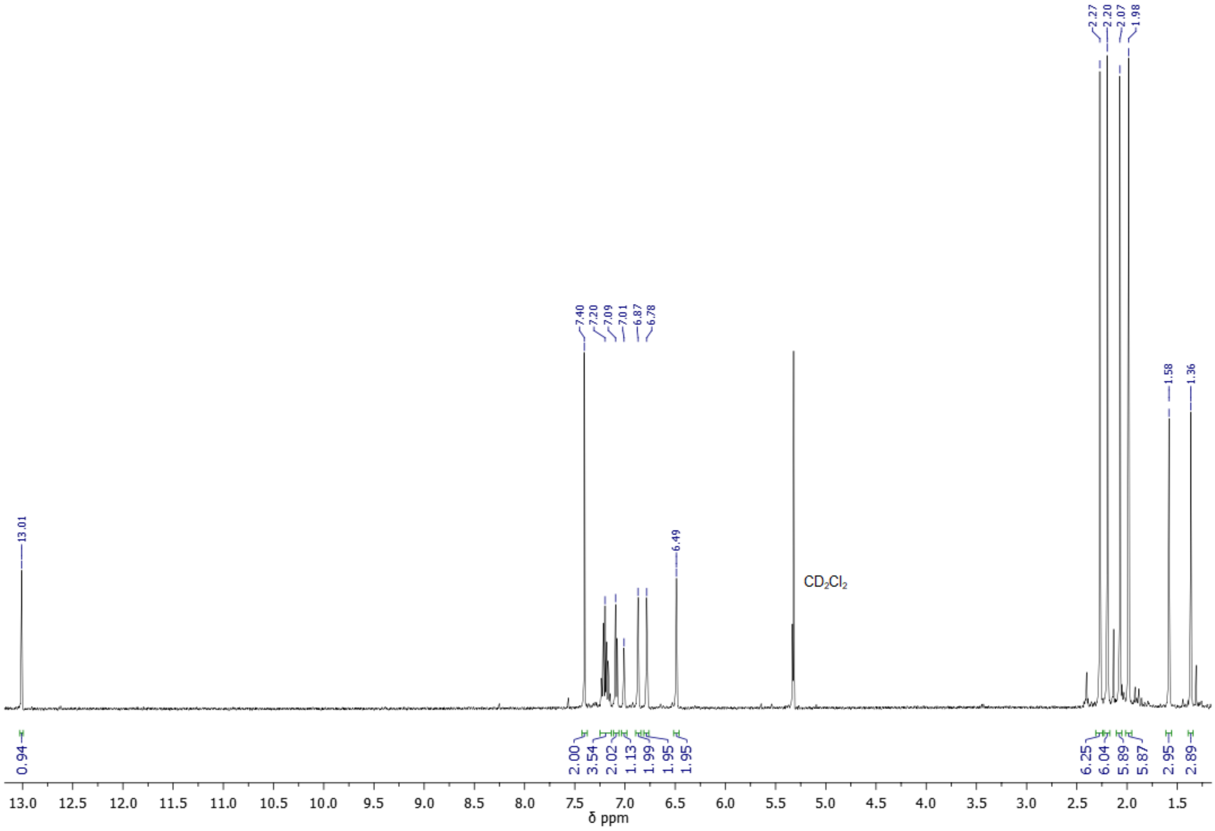

Figure S117. ^{1}H NMR (400 MHz, 25 °C, CD_2Cl_2) of **13** after 5 days in air.

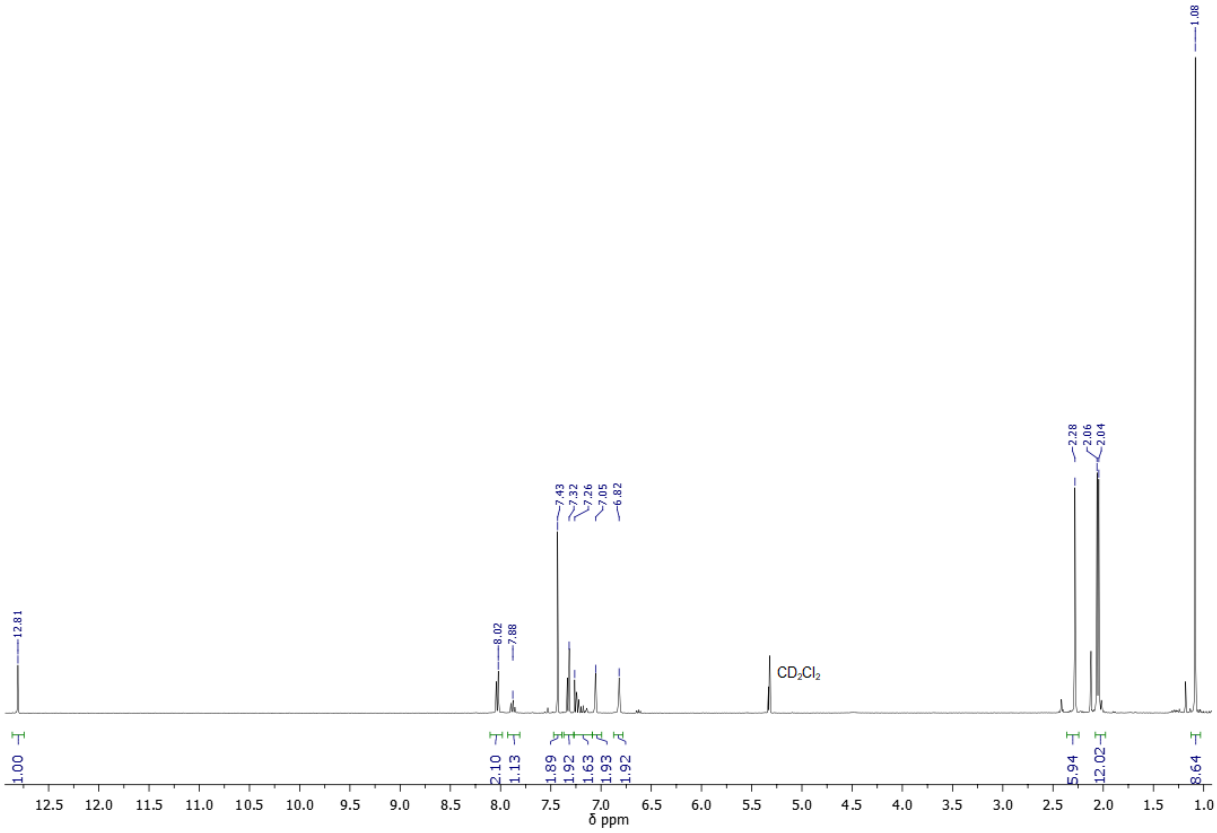

Figure S118. ^{1}H NMR (400 MHz, 25 °C, CD_2Cl_2) of **15a** after 5 days in air.

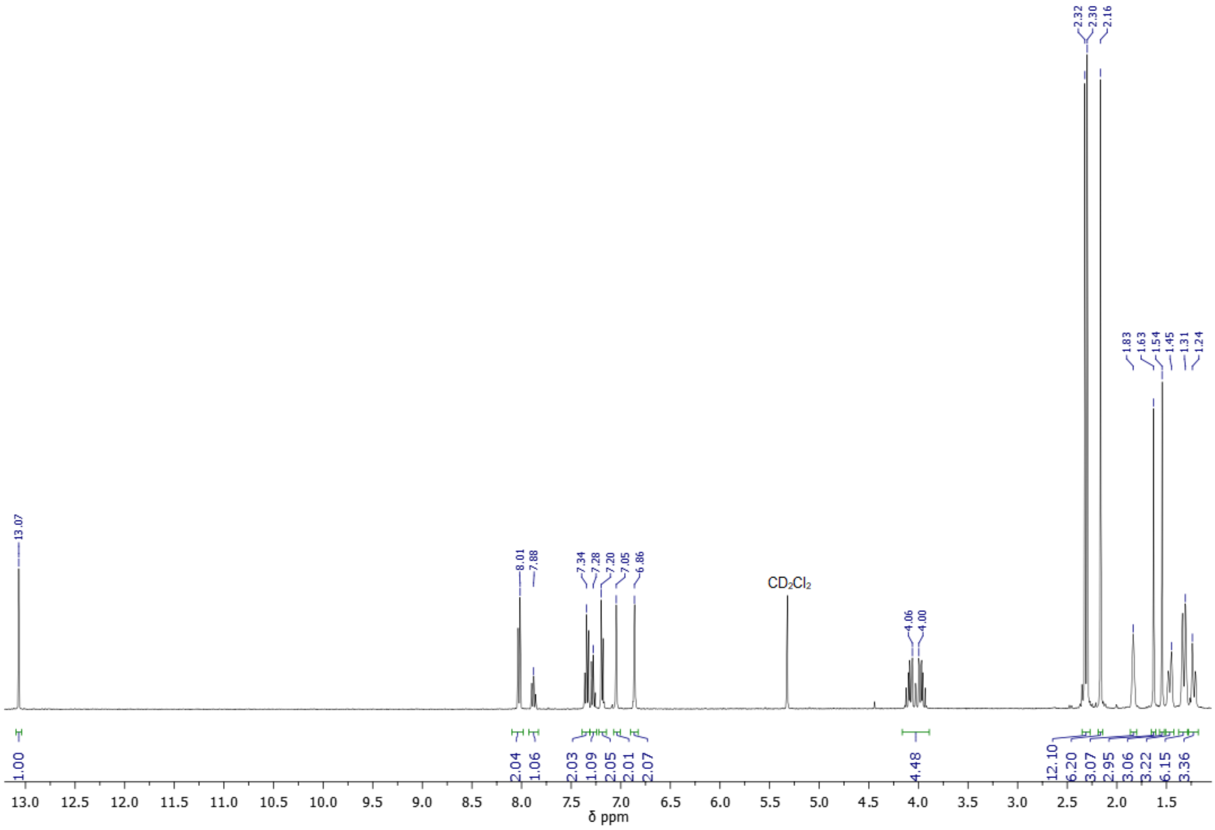

Figure S119. ^{1}H NMR (400 MHz, 25 °C, CD_2Cl_2) of **17a** after 5 days in air.

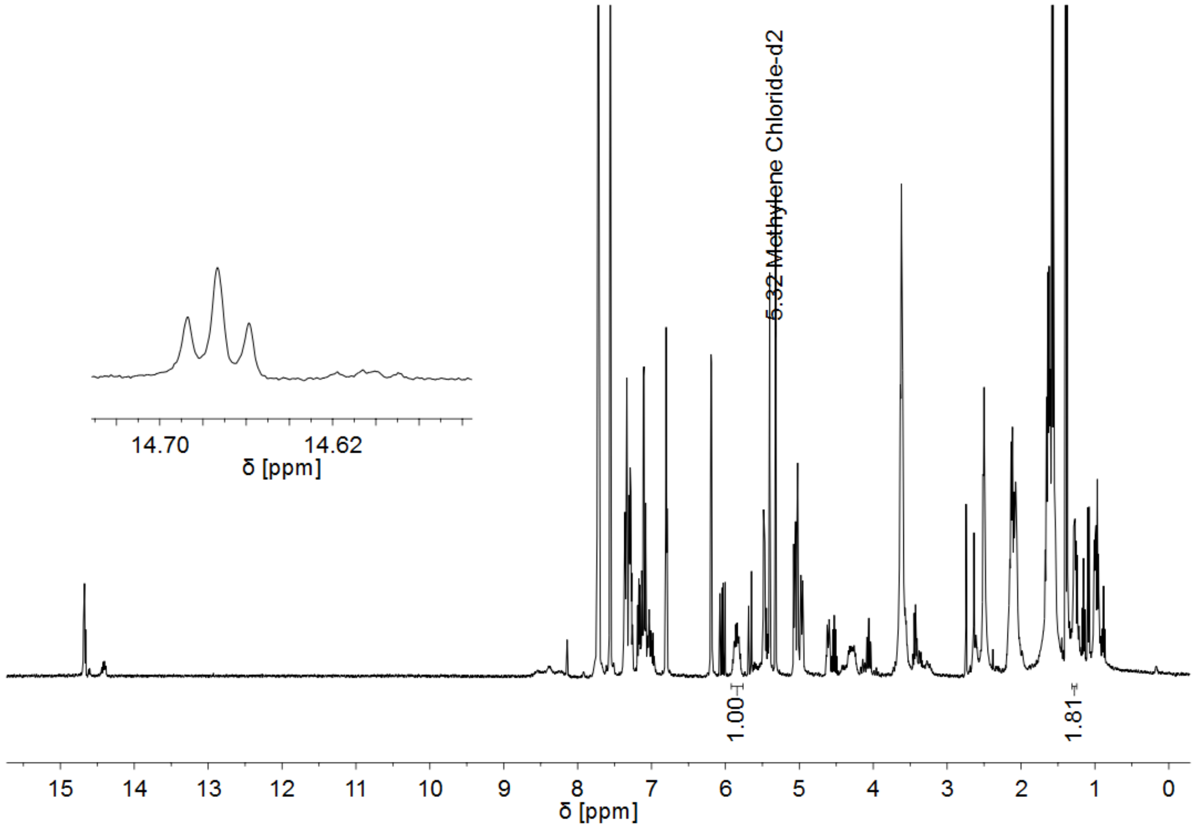

Figure S120. ^{1}H NMR (400 MHz, 25 °C, CD_2Cl_2) of **5c** after treatment with 4-penten-1-ol, expansion of the alkylidene region.

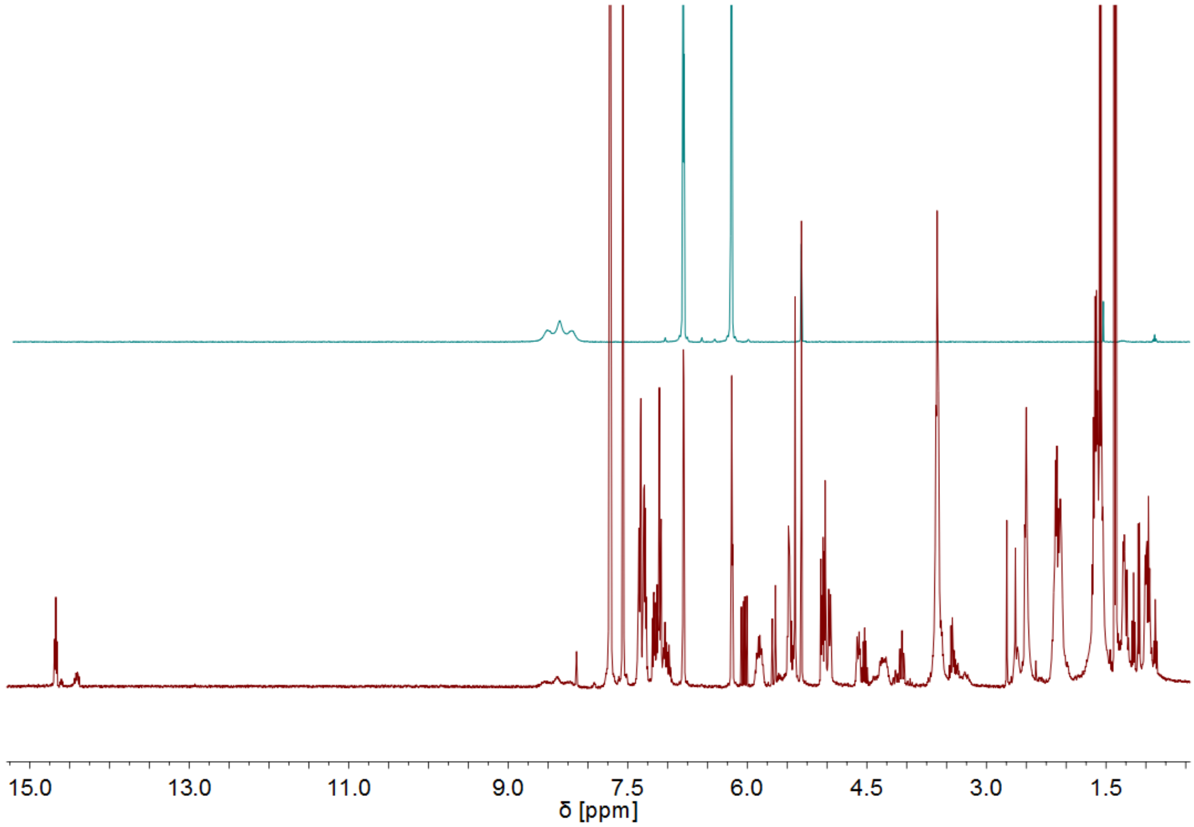

Figure S121. ^{1}H NMR (400 MHz, 25 °C, CD_2Cl_2) of **5c** after treatment with 4-penten-1-ol (bottom) and pyrrole (top).

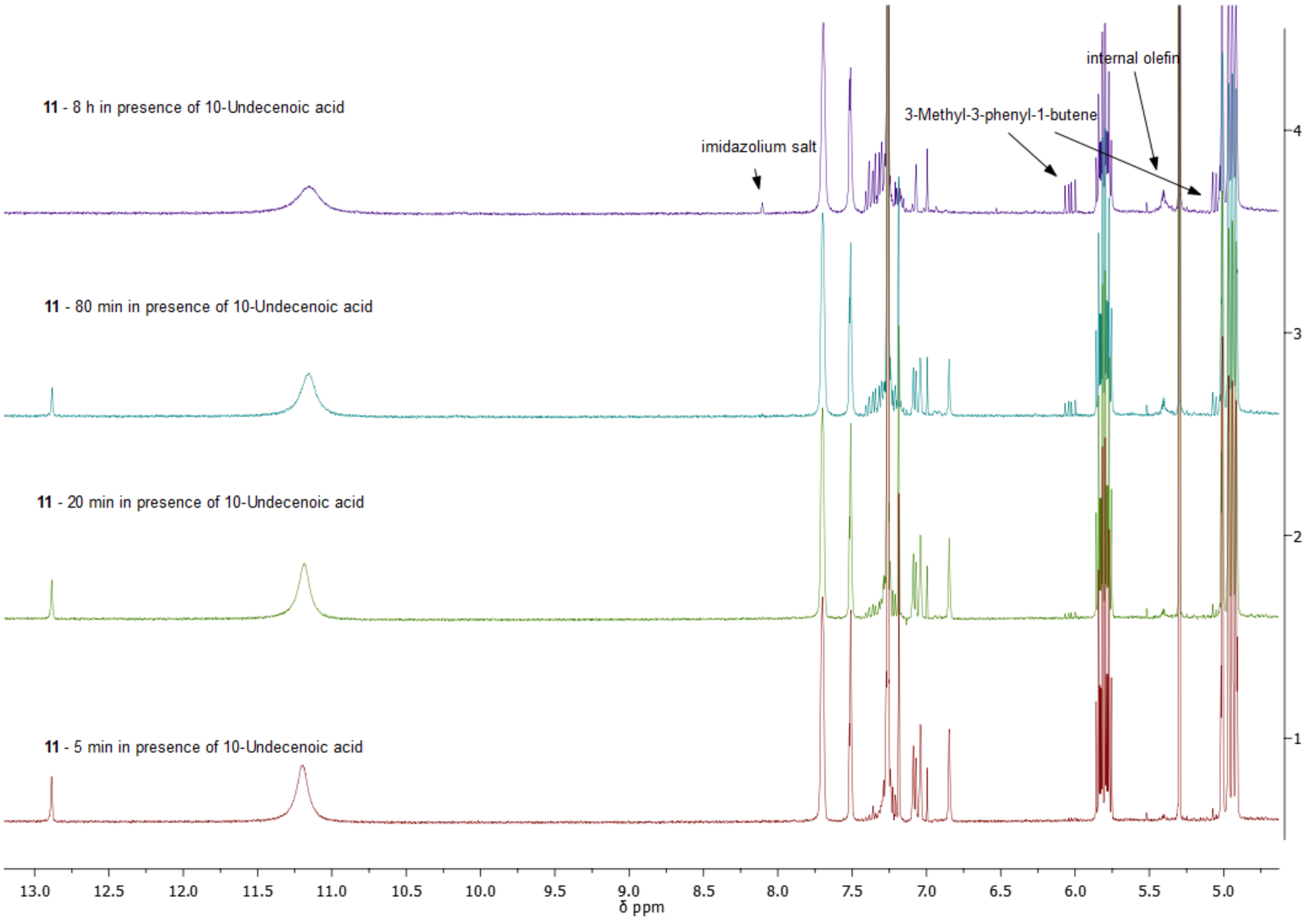

Figure S122. ^{1}H NMR (400 MHz, 25 °C, $CDCl_3$) of **11** in the presence of 10 equiv. 10-undecenoic acid.

Single Crystal X-Ray Analyses

X-ray Analysis: Single crystal X-ray measurements were carried out on a Bruker Kappa APEXII Duo diffractometer with Mo-Kα radiation at the Institute of Organic Chemistry, University of Stuttgart. CCDC 1946042 (**3a**), 1946041 (**4a*H₂O**), 1959263 (**4d**), 1959261 (**5d**), 1959260 (**5d-MeCN**), 1946043 (**6**) and 1959262 (**15b**) contain the supplementary crystallographic data for this paper. These data can be obtained free of charge from the Cambridge Crystallographic Data Centre.

Comment on checkcif: The B level alert in the analysis of compound **4a*H₂O** stems from the water molecules; one hydrogen of each water molecule lacks a classic acceptor for hydrogen bonding (here a carbon). This is routinely indicated by the software.

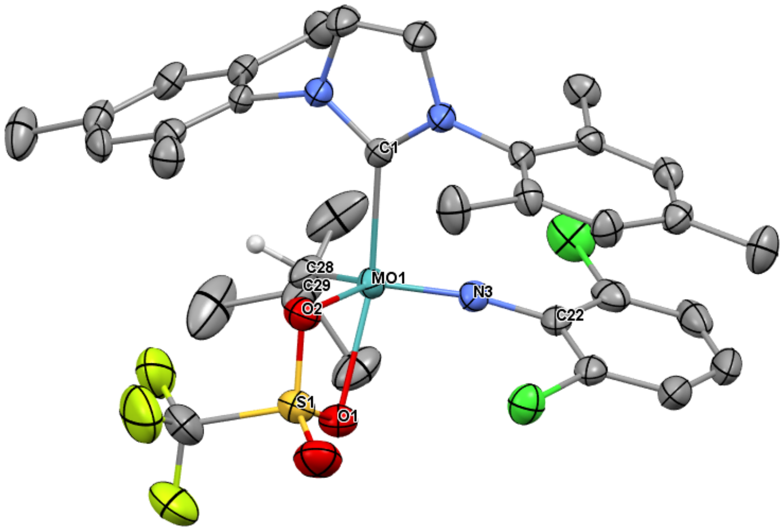

Figure S123. Single crystal X-ray structure of [Mo(N-2,6-Cl_2-C_6H_3)(CHCMe3)(IMes)(OTf)][B(Ar^F)^4], **3a**. Relevant bond lengths [pm] and angles [°]: Mo-N3 = 173.4, Mo-C28 = 186.7, Mo-O2 = 217.9, Mo-C1 = 218.2, Mo-O1 = 226.9, Mo-S = 280.2; N3-Mo-C28 = 104.81, N3-Mo-O2 = 136.15, C28-Mo-O2 = 115.56, N3-Mo-C1 = 99.87, C28-Mo-C1 = 101.72, O2-Mo-C1 = 88.45, N3-Mo-O1 = 98.45, C28-Mo-O1 = 94.12, O2-Mo-O1 = 63.52, C1-Mo-O1 = 151.75, C22-N3-Mo = 160.5, C29-C28-Mo = 144.3. Thermal ellipsoids are set at a 50% probability level. One CH_2Cl_2 molecule, the anion and hydrogens omitted for clarity.

Table S1. Crystal data and structure refinement for [Mo(*N*-2,6-Cl_2-C_6H_3)($CHCMe_3$)(IMes)(OTf)][B(Ar^F)^4].

Empirical formula	C65 H49 B Cl2 F27 Mo N3 O3 S
Formula weight	1642.78 g/mol
Temperature	130(2) K
Wavelength	0.71073 Å
Crystal system	monoclinic
Space-group	P 2_1/n
Cell parameters	a = 18.9245(17) Å, b = 16.5339(15) Å, c = 22.735(2) Å
	α = γ = 90 °, β = 94.994(3) °
Cell volume	7086.6(11) Å^3
Z	4
Calc. density	1.540 g/cm^3
Absorption coefficient	0.407 mm^{-1}
F(000)	3296
Crystal size	0.520 x 0.314 x 0.116 mm
Theta range for data collection	1.638 to 28.259 °
Limiting indices	-25<=h<=25, -21<=k<=22, -30<=l<=30
Reflections collected / unique	73512 / 17461 [R(int) = 0.0573]
Completeness to theta = 25.242	99.9%
Absorption correction	Semi-empirical from equivalents
Max. and min. transmission	0.7457 and 0.6847
Refinement method	Full-matrix least-squares on F^2
Data / restraints / parameters	17461 / 36 / 938
Goodness-of-fit on F^2	1.027
Final R indices [I>2σ(I)]	R1 = 0.0623, wR2 = 0.1496
R indices (all data)	R1 = 0.1154, wR2 = 0.1656
Largest diff. peak and hole	1.828 and -0.775 e.A^{-3}

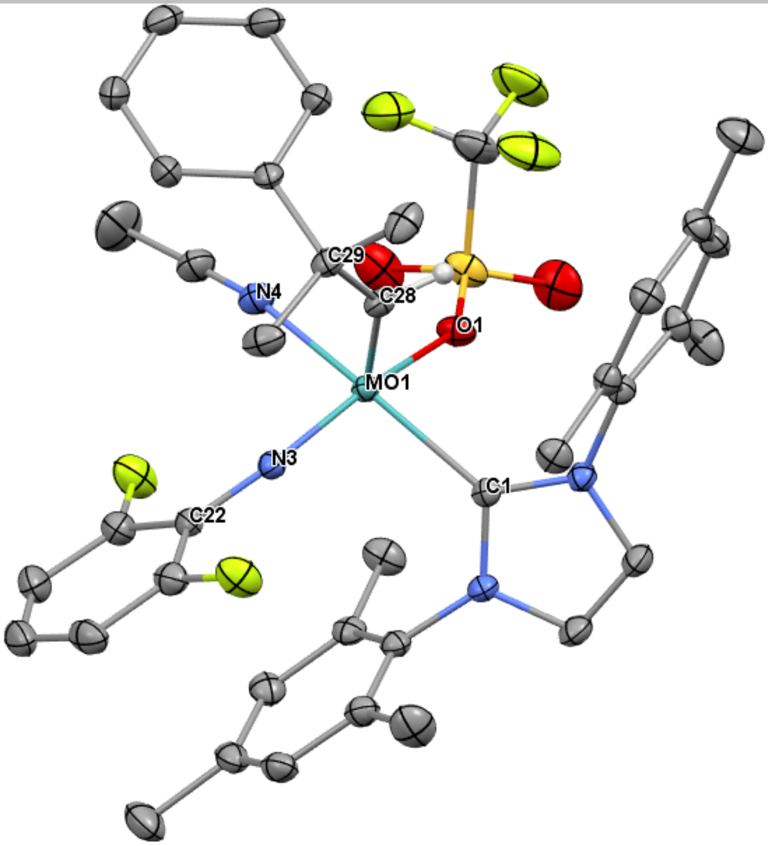

Figure S124. Single crystal X-ray structure of [Mo(N-2,6-F_2-C_6H_3)($CHCMe_2Ph$)(IMes)(OTf)(MeCN)][B(Ar^F)$_4$], **4d**. Relevant bond lengths [pm] and angles [°]: Mo-N3 = 173.1, Mo-C28 = 187.1, Mo-O1 = 208.9, Mo-N4 = 217.2, Mo-C1 = 218.3; N3-Mo-C28 = 102.03, N3-Mo-O1 = 151.93, C28-Mo-O1 = 105.03, N3-Mo-C1 = 97.06, C28-Mo-C1 = 102.72, N4-Mo-C1 = 160.29, N3-Mo-O1 = 151.93, C28-Mo-O1 = 105.03, N4-Mo-O1 = 81.88, C1-Mo-O1 = 84.21, C22-N3-Mo = 166.38, C29-C28-Mo = 143.56. The anion and hydrogens omitted for clarity. Thermal ellipsoids are set at a 50% probability level.

Table S2. Crystal data and structure refinement for [Mo(*N*-2,6-F_2-C_6H_3)($CHCMe_2Ph$)(IMes)(OTf)(MeCN)][B(Ar^F)^4].

Empirical formula	C72 H54 B F29 Mo N4 O3 S
Formula weight	1713.00 g/mol
Temperature	135(2) K
Wavelength	0.71073 Å
Crystal system	Triclinic
Space-group	$P\bar{1}$
Cell parameters	a = 12.7917(5) Å, b = 18.1854(7) Å, c = 18.6054(8) Å
	α = 64.059(2) °, γ = 73.654(2) °, β = 70.333(2) °
Cell volume	3618.6(3) $Å^3$
Z	2
Calc. density	1.572 g/cm^3
Absorption coefficient	0.335 mm^{-1}
F(000)	1724
Crystal size	0.643 x 0.470 x 0.461 mm
Theta range for data collection	1.712 to 33.254 °
Limiting indices	-19<=h<=17, -28<=k<=21, -28<=l<=28
Reflections collected / unique	109223 / 27676 [R(int) = 0.0317]
Completeness to theta = 25.242	100%
Absorption correction	Semi-empirical from equivalents
Max. and min. transmission	0.7465 and 0.7150
Refinement method	Full-matrix least-squares on F^2
Data / restraints / parameters	27676 / 168 / 1084
Goodness-of-fit on F^2	1.046
Final R indices [I>2σ(I)]	R1 = 0.0399, wR2 = 0.0969
R indices (all data)	R1 = 0.0640, wR2 = 0.1065
Largest diff. peak and hole	0.672 and -0.590 e.A^{-3}

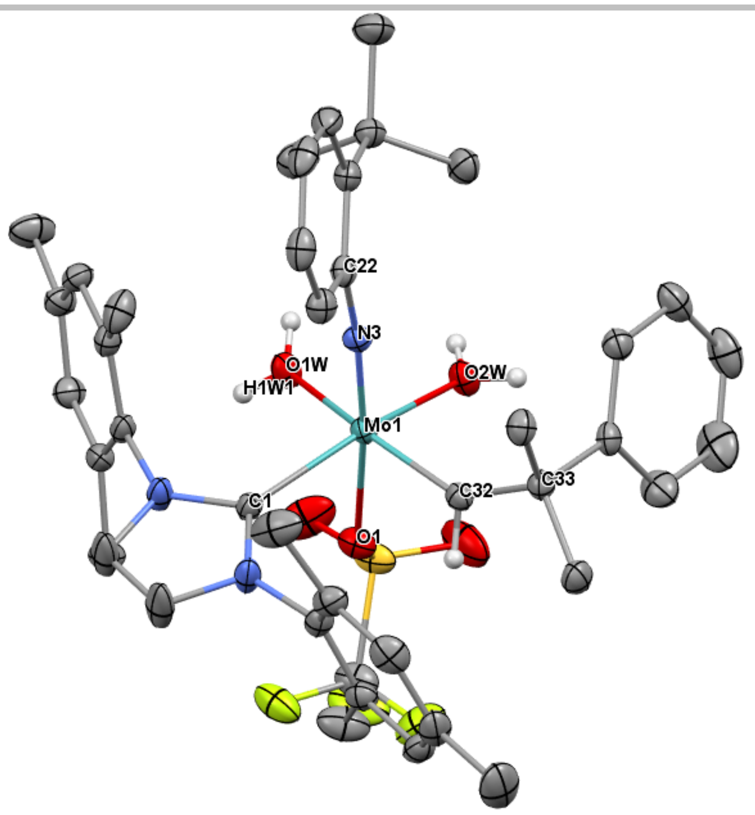

Figure S125. Single crystal X-ray structure of [Mo(*N*-2-*t*Bu-C_6H_4)($CHCMe_2Ph$)(IMes)(OTf)$(H_2O)_2$][B(Ar^F)$_4$], **4a*H₂O**. Relevant bond lengths [pm] and angles [°]: Mo-N3 = 173.2, Mo-C32 = 191.8, Mo-O2W = 219.2, Mo-C1 = 224.1, Mo-O1 = 226.8, Mo-O1W = 232.11; N3-Mo-C32 = 99.47, N3-Mo-O2W = 99.39, C32-Mo-O2W = 88.23, N3-Mo-C1 = 95.05, C32-Mo-C1 = 96.37, O2W-Mo-C1 = 163.92, N3-Mo-O1 = 172.82, C32-Mo-O1 = 87.69, O2W-Mo-O1 = 81.21, C1-Mo-O1 = 83.58, N3-Mo-O1W = 99.06, C32-Mo-O1W = 154.92, O2W-Mo-O1W = 72.15, C1-Mo-O1W = 98.69, O1-Mo-O1W = 74.25, C33-C32-Mo = 142.38, C22-N3-Mo = 172.02. Thermal ellipsoids are set at a 50% probability level. One CH_2Cl_2 molecule, anion and hydrogens were omitted for clarity.

Table S3. Crystal data and structure refinement for [Mo(*N*-2-*t*Bu-C_6H_4)($CHCMe_2Ph$)(IMes)(OTf)$(H_2O)_2$][B(Ar^F)$_4$].

Empirical formula	C75 H67 B Cl2 F27 Mo N3 O5 S
Formula weight	1813.03 g/mol
Temperature	130(2) K
Wavelength	0.71073 Å
Crystal system	Triclinic
Space-group	$P\overline{1}$
Cell parameters	a = 15.4478(14) Å, b = 16.3148(15) Å, c = 17.2214(16) Å
	α = 96.014(5) °, γ = 97.988(4) °, β = 110.634(5) °
Cell volume	3966.7(6) Å^3
Z	2
Calc. density	1.518 g/cm^3
Absorption coefficient	0.374 mm^{-1}
F(000)	1836
Crystal size	0.42 x 0.39 x 0.28 mm
Theta range for data collection	1.53 to 30.52 °
Limiting indices	-22<=h<=22, -23<=k<=23, -24<=l<=24
Reflections collected / unique	85852 / 24106 [R(int) = 0.0363]
Completeness to theta = 30.52	99.4%
Absorption correction	Semi-empirical from equivalents
Max. and min. transmission	0.7461 and 0.7168
Refinement method	Full-matrix least-squares on F^2
Data / restraints / parameters	24106 / 116 / 1083
Goodness-of-fit on F^2	1.038
Final R indices [I>2σ(I)]	R1 = 0.0452, wR2 = 0.1149
R indices (all data)	R1 = 0.0702, wR2 = 0.1246
Largest diff. peak and hole	1.075 and -0.840 e.A^{-3}

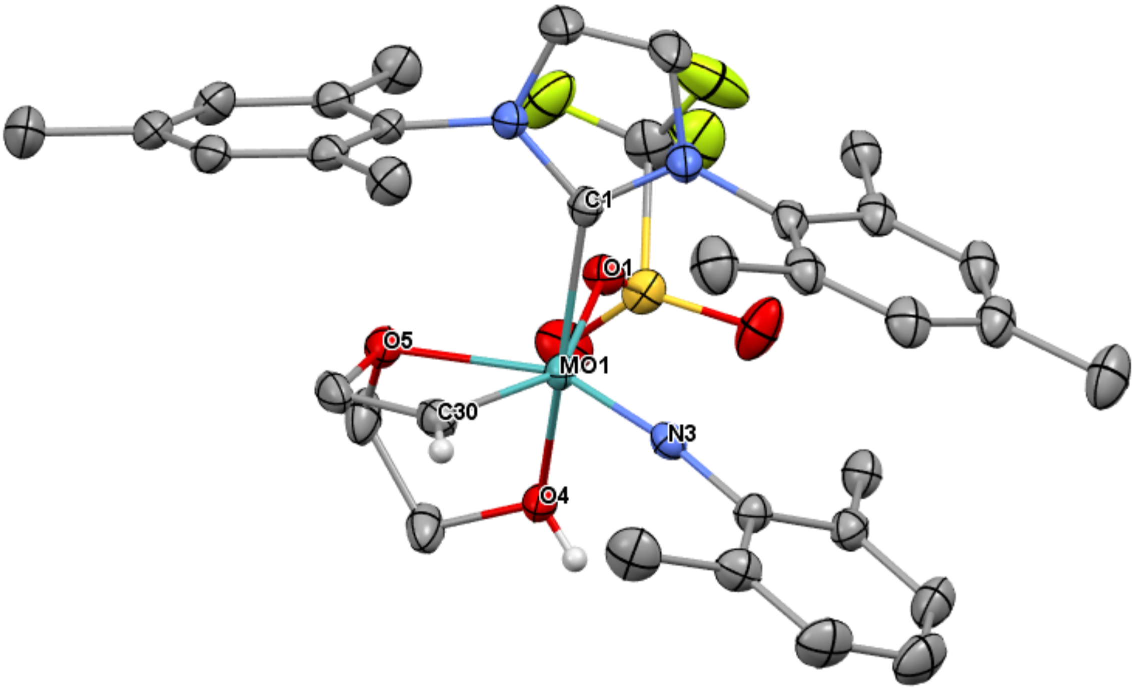

Figure S126. Single crystal X-ray structure of [Mo(*N*-2,6-Me_2-C_6H_4)($CHCH_2O(CH_2)_2OH$)(IMes)(OTf)][B(Ar^F)$_4$], **6**. Selected bond lengths [pm] and angles [°]: Mo-N3 = 171.8, Mo-C30 = 192.2, Mo-O4 = 217.7, Mo-O1 = 218.9, Mo-C1 = 220.5, Mo-O5 = 229.3; N3-Mo-C30 = 98.63, N3-Mo-O4 = 94.36, C30-Mo-O4 = 97.11, N3-Mo-O1 = 117.38, C30-Mo-O1 = 143.97, O4-Mo-O1 = 79.88, N3-Mo-C1 = 96.24, C30-Mo-C1 = 95.16, O4-Mo-C1 = 162.38, O1-Mo-C1 = 82.75, N3-Mo-O5 = 157.22, C30-Mo-O5 = 64.87, O4-Mo-O5 = 73.49, O1-Mo-O5 = 80.10, C1-Mo-O5 = 100.64, C22-N3-Mo = 168.47. Thermal ellipsoids are set at a 50% probability level. The anion, hydrogens and one Pr_2O molecule coordinating to the hydrogen on O4 were omitted for clarity.

Table S4. Crystal data and structure refinement for [Mo(*N*-2,6-Me_2-C_6H_4)($CHCH_2O(CH_2)_2OH$)(IMes)(OTf)][B(Ar^F)$_4$].

Empirical formula	C72 H67 B F27 Mo N3 O6 S
Formula weight	1722.09 g/mol
Temperature	130(2) K
Wavelength	0.71073 Å
Crystal system	Triclinic
Space-group	$P\overline{1}$
Cell parameters	a = 13.3310(6) Å, b = 17.3449(7) Å, c = 18.1979(9) Å
	α = 78.865(2) °, γ = 88.528(2) °, β= 88.052(2) °
Cell volume	4125.4(3) Å^3
Z	2
Calc. density	1.386g/cm^3
Absorption coefficient	0.293 mm^{-1}
F(000)	1748
Crystal size	0.264 x 0.223 x 0.060 mm
Theta range for data collection	1.486 to 26.491 °
Limiting indices	-16<=h<=16, -21<=k<=21, -22<=l<=22
Reflections collected / unique	75808 / 16980 [R(int) = 0.0309]
Completeness to theta = 25.242	100%
Absorption correction	Semi-empirical from equivalents
Max. and min. transmission	0.7454 and 0.7030
Refinement method	Full-matrix least-squares on F^2
Data / restraints / parameters	16980 / 72 / 1086
Goodness-of-fit on F^2	1.033
Final R indices [I>2σ(I)]	R1 = 0.0366, wR2 = 0.0915
R indices (all data)	R1 = 0.0487, wR2 = 0.0958
Largest diff. peak and hole	0.824 and -0.637e.A^{-3}

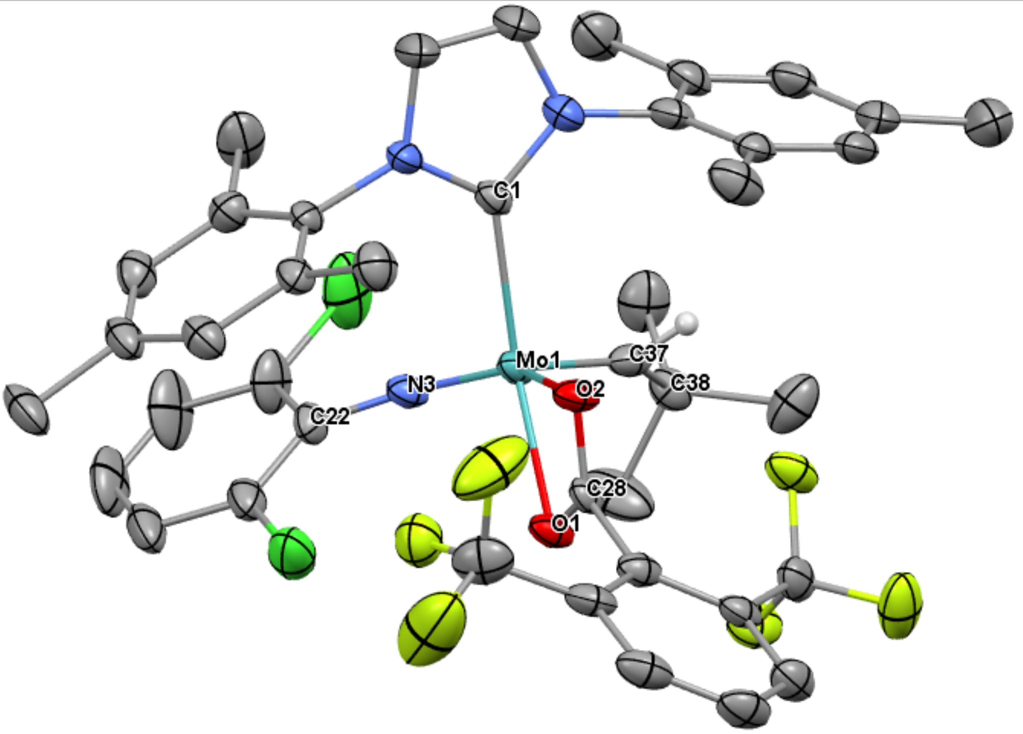

Figure S127. Single crystal X-ray structure of [Mo(N-2,6-Cl_2-C_6H_3)($CHCMe_3$)(IMes)(2,6-$(CF_3)_2$-C_6H_3)][B(Ar^F)^4], **15b**. Selected bond lengths [pm] and angles [°]: Mo-N3 = 172.5, Mo-C37 = 184.1, Mo-O2 = 218.6, Mo-C1 = 218.9, Mo-O1 = 219.1, Mo-C28 = 255.3; N3-Mo-C37 = 103.4, N3-Mo-O2 = 142.33, C37-Mo-O2 = 109.69, N3-Mo-C1 = 101.58, C37-Mo-C1 = 101.41, O2-Mo-C1 = 89.29, N3-Mo-O1 = 100.69, C37-Mo-O1 = 95.22, O2-Mo-O1 = 59.42, C1-Mo-O1 = 148.17, C22-N3-Mo = 165.1, C38-C37-Mo = 145.9. Thermal ellipsoids are set at a 50% probability level. The B(Ar^F)$_4$ anion and hydrogens except for H on C37 were omitted for clarity.

Table S5. Crystal data and structure refinement for [Mo(*N*-2,6-Cl_2-C_6H_3)($CHCMe_3$)(IMes)(2,6-$(CF_3)_2$-C_6H_3)][B(Ar^F)^4].

Empirical formula	C73 H52 B Cl2 F30 Mo N3 O2
Formula weight	1750.83 g/mol
Temperature	130(2) K
Wavelength	0.71073 Å
Crystal system	Triclinic
Space-group	$P\overline{1}$
Cell parameters	a = 9.4743(4) Å, b = 19.9174(8) Å, c = 20.9981(9) Å
	α = 105.326(2) °, γ = 102.824(2) °, β= 98.096(3) °
Cell volume	3641.4(3) Å^3
Z	2
Calc. density	1.597 g/cm^3
Absorption coefficient	0.379 mm^{-1}
F(000)	1756
Crystal size	0.35 x 0.33 x 0.08 mm
Theta range for data collection	1.72 to 25.00 °
Limiting indices	-11<=h<=9, -23<=k<=23, -24<=l<=24
Reflections collected / unique	46897 / 12462 [R(int) = 0.0405]
Completeness to theta = 25.00	97.1%
Absorption correction	Numerical
Max. and min. transmission	0.9978 and 0.7912
Refinement method	Full-matrix least-squares on F^2
Data / restraints / parameters	12462 / 6 / 1018
Goodness-of-fit on F^2	1.047
Final R indices [I>2σ(I)]	R1 = 0.0627, wR2 = 0.1452
R indices (all data)	R1 = 0.0952, wR2 = 0.1603
Largest diff. peak and hole	2.036 and -0.713 e.A^{-3}

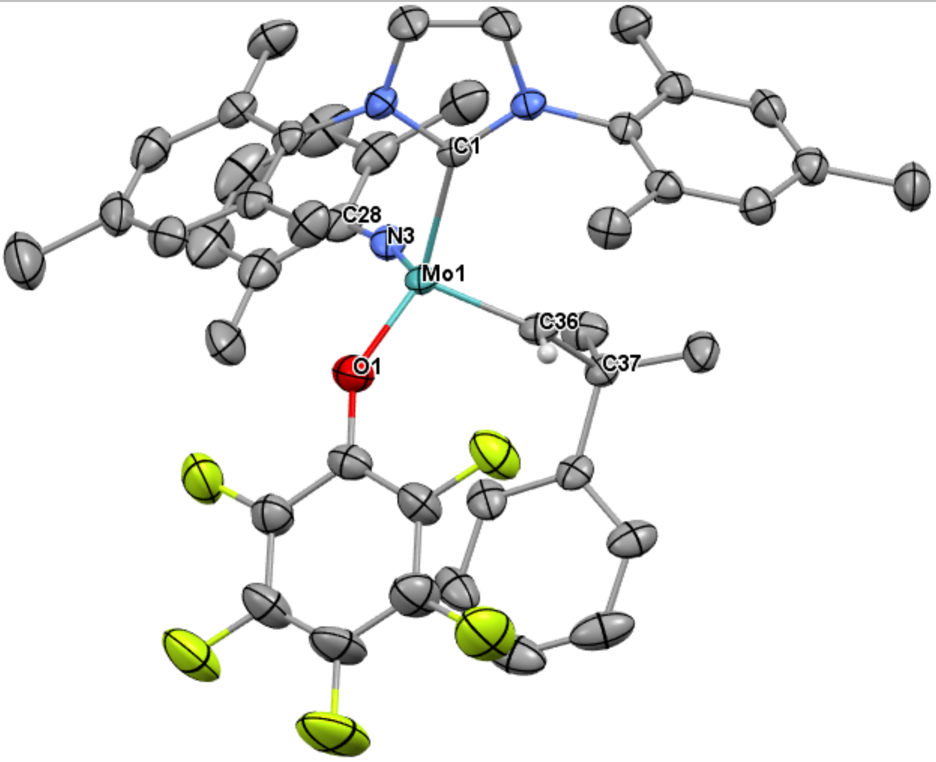

Figure S128. Single crystal X-ray structure of **5d**. Relevant bond lengths [pm] and angles [°]: Mo-N3 = 172.5, Mo-C36 = 187.2, Mo-O1 = 193.9, Mo-C1 = 214.6; N3-Mo-C36 = 103.63, N3-Mo-O1 = 121.42, C36-Mo-O1 = 105.79, N3-Mo-C1 = 101.63, C36-Mo-C1 = 101.46, O1-Mo-C1 = 120.12, Mo-C36-H36 = 96, C37-C36-Mo = 144.8, C28-N3-Mo = 167.1. Thermal ellipsoids are set at a 50% probability level. All hydrogen atoms except for H at C36 and $B(Ar^F)_4$ anion are omitted for clarity. τ_4' = 0.84. Mo adopts a distorted tetrahedral geometry.[20]

Table S6. Crystal data and structure refinement for $[Mo(N\text{-}2,6\text{-}Me_2\text{-}C_6H_3)(CHCMe_2Ph)(IMes)(OC_6F_5)][B(Ar^F)_4]$.

Empirical formula	C77 H57 B F29 Mo N3 O
Formula weight	1698.01 g/mol
Temperature	130(2) K
Wavelength	0.71073 Å
Crystal system	monoclinic
Space-group	$P\,2_1/n$
Cell parameters	a = 19.4445(10) Å, b = 18.7532(10) Å, c = 21.3407(11) Å
	α = γ = 90°, β = 103.348(3)°
Cell volume	7571.6(7) $Å^3$
Z	4
Calc. density	1.490 g/cm^3
Absorption coefficient	0.291 mm^{-1}
F(000)	3424
Crystal size	0.52 x 0.47 x 0.36 mm
Theta range for data collection	1.46 to 28.35 °
Limiting indices	-25<=h<=25, -24<=k<=24, -28<=l<=28
Reflections collected / unique	83780 / 18768 [R(int) = 0.0490]
Completeness to theta = 28.35	99.3%
Absorption correction	Numerical
Max. and min. transmission	0.9873 and 0.8519
Refinement method	Full-matrix least-squares on F^2
Data / restraints / parameters	18768 / 246 / 1106
Goodness-of-fit on F^2	1.042
Final R indices [I>2σ(I)]	R1 = 0.0551, wR2 = 0.1322
R indices (all data)	R1 = 0.1210, wR2 = 0.1575
Largest diff. peak and hole	1.022 and -0.554 e.A^{-3}

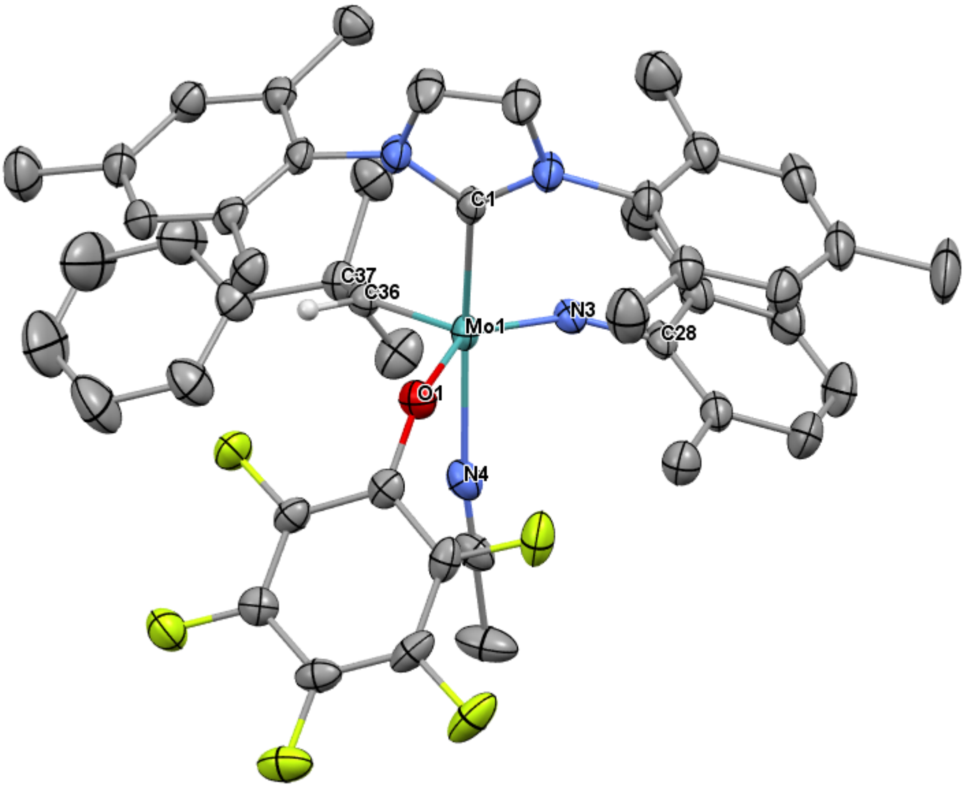

Figure S129. Single crystal X-ray structure of **5d-MeCN**. Relevant bond lengths [pm] and angles [°]: Mo-N3 = 174.3, Mo-C36 = 189.2, Mo-O1 = 200.03, Mo-N4 = 220.0, Mo-C1 = 221.0; N3-Mo1-C36 = 105.46, N3-Mo1-O1 = 140.04, C36-Mo1-O1 = 112.52, N3-Mo1-N4 = 86.05, C36-Mo1-N4 = 90.36, O1-Mo1-N4 = 81.85, N3-Mo1-C1 = 100.51, C36-Mo1-C1 = 101.71, O1-Mo1-C1 = 83.87, N4-Mo1-C1 = 164.04, C28-N3-Mo = 156.5, C37-C36-Mo = 142.6, Mo-C36-H36 = 101. Thermal ellipsoids are set at a 50% probability level. All hydrogen atoms except for H at C36 and $B(Ar^F)_4$ anion are omitted for clarity. The acetonitrile adduct of **5d** adopts a geometry intermediate between trigonal bipyramidal and square pyramidal with τ_5 = 0.4.[20]

Table S7. Crystal data and structure refinement for [Mo(*N*-2,6-Me_2-C_6H_3)($CHCMe_2Ph$)(IMes)(OC_6F_5)(MeCN)][$B(Ar^F)_4$].

Empirical formula	C79 H60 B F29 Mo N4 O
Formula weight	1739.06 g/mol
Temperature	130(2) K
Wavelength	0.71073 Å
Crystal system	monoclinic
Space-group	*P* 2_1/n
Cell parameters	a = 12.7337(7) Å, b = 37.442(2) Å, c = 16.5162(9) Å
	α = γ = 90 °, β = 100.906(3) °
Cell volume	7732.2(7) Å^3
Z	4
Calc. density	1.494 g/cm^3
Absorption coefficient	0.287 mm^{-1}
F(000)	3512
Crystal size	0.30 x 0.23 x 0.13 mm
Theta range for data collection	1.66 to 28.33 °
Limiting indices	-16<=h<=16, -49<=k<=49, -22<=l<=21
Reflections collected / unique	90866 / 19024 [R(int) = 0.0391]
Completeness to theta = 28.33	98.8%
Absorption correction	Numerical
Max. and min. transmission	0.9877 and 0.9142
Refinement method	Full-matrix least-squares on F^2
Data / restraints / parameters	19024 / 172 / 1052
Goodness-of-fit on F^2	1.049
Final R indices [I>2σ(I)]	R1 = 0.0523, wR2 = 0.1189
R indices (all data)	R1 = 0.0751, wR2 = 0.1264
Largest diff. peak and hole	0.982 and -0.802 e.A^{-3}

High Resolution Mass Spectra (ESI)

Acquisition Parameter

Source Type	ESI	Ion Polarity	Positive	Set Nebulizer	0.4 Bar
Focus	Not active	Set Capillary	4500 V	Set Dry Heater	200 °C
Scan Begin	50 m/z	Set End Plate Offset	-500 V	Set Dry Gas	4.0 l/min
Scan End	1500 m/z	Set Collision Cell RF	180.0 Vpp	Set Divert Valve	Waste

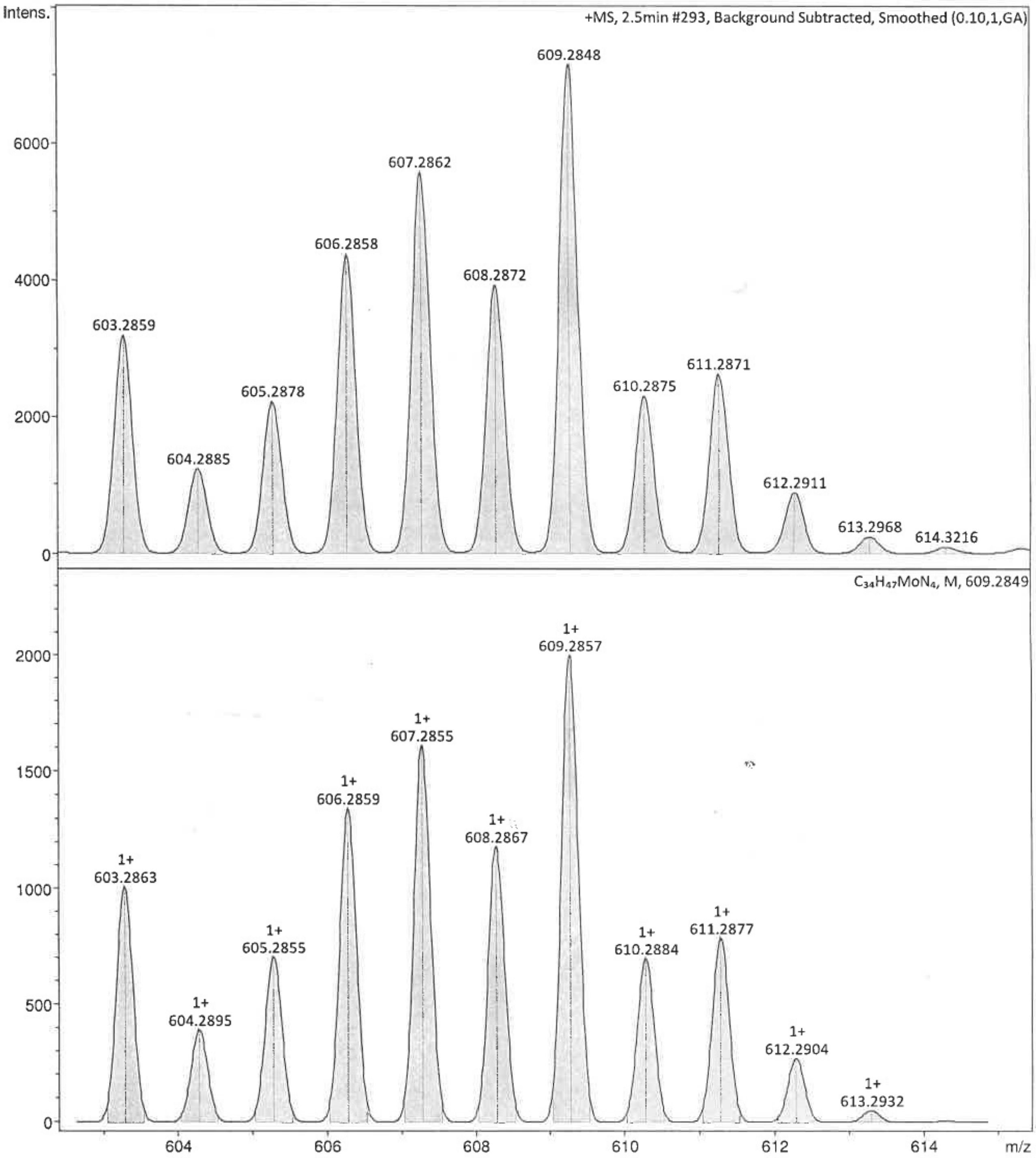

Figure S 130: High resolution mass spectrum of 2b. Found (top) and calculated (bottom) isotope distribution of the molecular ion peak.

References

[1] G. R. Fulmer, A. J. M. Miller, N. H. Sherden, H. E. Gottlieb, A. Nudelman, B. M. Stoltz, J. E. Bercaw, K. I. Goldberg, *Organometallics* **2010**, *29*, 2176-2179.

[2] J. Zhao, D. Wang, B. Autenrieth, M. R. Buchmeiser, *Macromol. Rapid Commun.* **2015**, *36*, 190-194.

[3] J. Yuan, R. R. Schrock, P. Müller, J. C. Axtell, G. E. Dobereiner, *Organometallics* **2012**, *31*, 4650-4653.
[4] A. J. Arduengo, R. Krafczyk, R. Schmutzler, H. A. Craig, J. R. Goerlich, W. J. Marshall, M. Unverzagt, *Tetrahedron* **1999**, *55*, 14523-14534.
[5] Z. Wang, H. Qin, *Green Chem.* **2004**, *6*.
[6] N. A. Yakelis, R. G. Bergman, *Organometallics* **2005**, *24*, 3579-3581.
[7] P. S. Kumar, K. Wurst, M. R. Buchmeiser, *J. Am. Chem. Soc.* **2009**, *131*, 387-395.
[8] I. Krossing, *Chem. Eur. J.* **2001**, *7*, 490-502.
[9] S. Sen, J. Unold, W. Frey, M. R. Buchmeiser, *Angew. Chem. Int. Ed.* **2014**, *53*, 9384-9388.
[10] A. J. Jiang, R. R. Schrock, P. Müller, *Organometallics* **2008**, *27*, 4428- 4438.
[11] M. J. Benedikter, R. Schowner, I. Elser, P. Werner, K. Herz, L. Stöhr, D. A. Imbrich, G. M. Nagy, D. Wang, M. R. Buchmeiser, *Macromolecules* **2019**, *52*, 4059-4066.
[12] I. Elser, M. J. Benedikter, R. Schowner, W. Frey, D. Wang, M. R. Buchmeiser, *Organometallics* **2019**, *38*, 2461-2471.
[13] K. Herz, M. Podewitz, L. Stohr, D. Wang, W. Frey, K. R. Liedl, S. Sen, M. R. Buchmeiser, *J. Am. Chem. Soc.* **2019**, *141*, 8264-8276.
[14] I. Elser, B. R. Kordes, W. Frey, K. Herz, R. Schowner, L. Stohr, H. J. Altmann, M. R. Buchmeiser, *Chem. Eur. J.* **2018**, *24*, 12652-12659.
[15] S. Sen, R. Schowner, D. A. Imbrich, W. Frey, M. R. Buchmeiser, *Chem. Eur. J.* **2015**, *21*, 13778-13787.
[16] M. R. Buchmeiser, S. Sen, C. Lienert, L. Widmann, R. Schowner, K. Herz, P. Hauser, W. Frey, D. Wang, *ChemCatChem* **2016**, *8*, 2710-2723.
[17] H. Jeong, J. M. John, R. R. Schrock, *Organometallics* **2015**, *34*, 5136-5145.
[18] R. R. Schrock, J. Y. Jamieson, S. J. Dolman, S. A. Miller, P. J. Bonitatebus, A. H. Hoveyda, *Organometallics* **2002**, *21*, 409-417.
[19] R. Schowner, W. Frey, M. R. Buchmeiser, *Eur. J. Inorg. Chem.* **2019**, *2019*, 1911-1922.
[20] a) A. W. Addison, T. N. Rao, J. Reedijk, J. van Rijn, G. C. Verschoor, *J. Chem. Soc., Dalton Trans.* **1984**, 1349-1356; b) L. Yang, D. R. Powell, R. P. Houser, *Dalton Trans.* **2007**, 955-964; c) D. Rosiak, A. Okuniewski, J. Chojnacki, *Polyhedron* **2018**, *146*, 35-41.
